# Genome skimming and NMR chemical fingerprinting provide quality assurance biotechnology to validate Sarsaparilla identity and purity

**DOI:** 10.1038/s41598-020-76073-7

**Published:** 2020-11-05

**Authors:** Prasad Kesanakurti, Arunachalam Thirugnanasambandam, Subramanyam Ragupathy, Steven G. Newmaster

**Affiliations:** NHP Research Alliance, College of Biological Sciences, University of Guelph, Guelph, ON Canada

**Keywords:** Metabolomics, PCR-based techniques, Plant sciences, Genomics

## Abstract

Sarsaparilla is a popular natural health product (NHP) that has been reported to be one of the most adulterated botanicals in the marketplace. Several plausible explanations are documented including economically motivated product substitution, unintentional errors due to ambiguous trade name associated with several different taxa, and wild harvesting of incorrect non-commercial plants. Unfortunately, this includes the case of an adulterant species *Decalepis hamiltonii,* a Red listed medicinal plant species by the International Union for Conservation of Nature (IUCN) and declared as a species with high conservation concern by the National Biodiversity Authority of India (NBA). This study provides validated genomic (genome skimming & DNA probes) and metabolomic (NMR chemical fingerprints) biotechnology solutions to prevent adulteration on both raw materials and finished products. This is also the first use of Oxford Nanopore on herbal products enabling the use of genome skimming as a tool for quality assurance within the supply chain of botanical ingredients. The validation of both genomics and metabolomics approach provided quality assurance perspective for both product identity and purity. This research enables manufactures and retailers to verify their supply chain is authentic and that consumers can enjoy safe, healthy products.

## Introduction

Sarsaparilla is a common name that encompasses several species that belong to different genera. Two groups of Sarsaparilla are found in the market namely Indian and North American Sarsaparilla. *Hemidesmus indicus* is known as Indian Sarsaparilla, which belongs to the family Apocynaceae and *Periploca indica* is an accepted synonym for this plant species^1^. Traditionally in Ayurvedic medicine, this plant has been used as anti-spasmodic, memory enhancing, and anti-inflammatory agent. Previous studies identified 2-hydroxy-4-methoxybenzaldehyde and ledol as major ingredients along with 40 other minor constituents^2^. In North America, species of the genus Smilax are commonly named as Sarsaparilla. *Smilax aristolochiifolia*, *S. ornata*, *S. china* are the common species used in commerce. Traditionally, the roots of these plants have been used as a diuretic^3^ and sudorific^4^. The scientific literature has also reported antibacterial, antifungal, anticancer, antidiabetic and antioxidant properties of North American Sarsaparilla^5^. All types of Sarsaparilla ingredients are prone to economically motivated and unintended adulteration throughout the supply chain. Considerable commercial demand for *H. indicus* have drastically reduced the wild populations^6,7^. Consequently, this has stimulated the adulteration with roots of other plants such as *Decalepis hamiltonii* that belong to the same family (Apocynaceae). The roots of authentic Sarsaparilla are typically used in commercial preparations of the Sarsaparilla. The roots of *D. hamiltonii* are often used as substitute for *H. indicus* because they are abundant and easy to harvest; the roots are bulky and loosely attached to the soil, which makes it easier to harvest than *H. indicus* roots, which are very thin, short and firmly attached to the soil costing more resources to harvest^8^. Similarly, it was noticed that *Smilax* spp. in North America are adulterated with root like rhizomes of eastern bracken fern, *Pteridium aquilinum*, which grows in similar habitats. It is difficult to identify the roots of *H. indicus* using their morphology because of the lack of distinguishing characteristics. Therefore, alternative methods such as molecular diagnostic tools are needed to verify the identity of the authentic *H. indicus* roots.

DNA barcoding is one of the molecular methods that can be used for species identification. Standardized gene regions such as rbcL, matK and ITS2 are generally used as barcode markers^9,10^. Using the barcoding approach, 600–800 bp long sequences of candidate barcode regions are amplified in target species using conserved primers. Resulting amplicon is sequenced to identify genetic differences and species are identified. Mishra et al.^8^ examined the efficacy of recommended barcode markers rbcL, matK, trnH-psbA, ITS and ITS2 in differentiating three congeneric species of the genus Decalepis. They also examined the ability of these markers to separate *Decalepis* spp. from *H. indicus*. Their results suggest that combination of rbcL + matK + ITS provided accurate signal in differentiating *Decalepis* spp and a SNP difference at 230 bp in matK was able to distinguish *H. indicus* from *Decalepis* spp. However, using the barcoding approach for species identification in herbal products has some limitations. Generally, it is difficult to obtain ~ 700 bp long amplicons from herbal products due to the sheared nature of DNA present in them^11^. It also involves several post-PCR processing steps, which may take several days, to identify the species ingredients of a product. The resources required to achieve a successful DNA barcoding test are prohibitive to commercial applications.

In order to overcome the limitations of DNA barcoding, we advocate the use of combination of metabolomic and genomic approaches. One of the metabolomic approaches popularly used in pharmaceutical industry is Nuclear Magnetic Resonance (NMR). Due to its efficacy, several studies used this technique in herbal industry for verification of quality assurance. For example, Hachem et al.^12^, demonstrated the ability of ^1^H NMR spectroscopy for the detection, identification and quantification of adulterants in 160 herbal food supplements sold in the market. This study highlighted poor manufacturing practices as evidence for the variability of active pharmaceutical ingredients in capsules of the same box. A similar study was conducted on 150 herbal dietary supplements^13^ demonstrated the utility of the ^1^H NMR spectroscopy in verifying quality in a good manufacturing process (GMP). In this study they also used mass spectrometry as a complementary method for confirming the chemical structures identified in the NMR profiles. The qNMR spectroscopy method provides accurate results with its absolute and relative quantifications, which include a measure of sensitivity with reasonable errors reported to be less than 1% and 0.2%, respectively^14^. Choi et al.^15^ reported that the NMR fingerprinting could efficiently differentiate between transgenic and wild type plants. Hence, NMR tools can be used for differentiating GMO and non-GMO plants, animals and derived by-products. In addition, NMR was also used for assessing the quality of coffee, and distinguishing between superior (Arabica coffee) and inferior (Robusta coffee) cultivars^16^. Likewise, Vogels et al.^17^ studied proton NMR spectroscopy’s ability to determine adulteration in orange juice. Literature states that the qNMR tool has the unique capabilities of both authentication and quantification of commercial botanical ingredients^18^. qNMR is one of the standard-free quantification tools and has capabilities to analyze multiple mixtures without any internal and external standards^19^. Nuclear magnetic resonance (NMR) is a fast and accurate analytical method. Most recently, the phytochemical analysis and metabolomics/chemical profiling by NMR have also been used for the authentication of botanicals and plant extracts^20^. The added benefit of NMR is that it provides a metabolite profile that can be used for identity of closely related species not differentiated by genetic markers. This provides a quick screen for unknown adulterants, overcoming the very difficult task of developing a DNA-based test assay for some extract ingredients used in food and natural health products.

Genome skimming involves shallow sequencing of the target genome to retrieve highly repetitive regions such as ribosomal DNA (rDNA) and plastid DNA. Previous studies used this approach to resolve the phylogeny of tropical trees^21,22^, on herbarium material for plant identification and phylogenomics^23,24^. In this study, we used nanopore sequencing technology for the first time on botanical products to retrieve long stretches of chloroplast genome from *D. hamiltonii* and *H. indicus* to generate more markers for assay development. Using this new sequence information, hydrolytic probe-based assays were designed for quick identification of Sarsaparilla species. Although some barcodes were able to differentiate the specimens of Sarsaparilla group, genome skimming provides several advantages such as, extra sequencing information that can provide more reference sequences for development of multiple assays. We feel the genome skimming approach will overcome issues known to single barcode markers such as low specificity (lack of species differentiation) and sensitivity due to amplification issues in processed samples with degraded DNA due to allele dropout. The development of hydrolytic probe-based assays will enable qPCR assays on portable devices, which can provide quick and accurate response within the supply chain verification.

This goal of this study is to provide a validated genomic (DNA barcoding, genome skimming & DNA probes) and metabolomic (NMR chemical fingerprints) biotechnology solutions to prevent adulteration on both raw materials and finished Sarsaparilla products. More specifically we used both genomic and metabolomic methods to identify samples of known provenance and those in the commercial supply chain. The validation of both genomics and metabolomics approaches provided quality assurance perspective for both product identity and purity.

## Results

### DNA barcoding of Sarsaparilla group

Conventional barcode markers such as rbcL and matK couldn’t amplify 50% of the samples tested in this study. We tested all 24 samples using generic barcoding primers of rbcL and matK that have amplicon lengths of around 500–700 bp. Out of 24 samples tested, four reference samples Pteaq_51470, 75NAT, SR140 and SR281 were successfully amplified by both the markers. However, the amplification rate in the samples obtained from manufacturing and retail units is very low. Samples 461NW, 867NW, BRM 278, BRM 391, 335BI, 336BI, 337BI and Amasar2 were amplified and the remaining 12 samples were not amplified by these markers. Due to such low rate of amplification we did not proceed further with this ineffective method.

### Genome skimming of *Decalepis hamiltonii* and *Hemidesmus indicus* using MinION of Oxford Nanopore Technology

*Decalepis hamiltonii* and *Hemidesmus indicus* genomes were scanned using MinION device to generate reference sequences that can be used to identify variable regions that can tell them apart. Good quality, high molecular weight DNA was obtained from roots of both species. More than 2.1 M reads were obtained after sequencing the DNA library of *D. hamiltonii* on MinION device in 24 h. Filtering was required to remove 376,000 reads that did not pass the quality threshold; the remaining 1.7 M reads had good quality scores. For *H. indicus* we obtained nearly 144,000 reads after running the device for the same length of time. Filtering removed 24,000 reads that did not pass the quality threshold; the remaining 120,000 reads had good quality scores.

### Development of new multi-loci markers for identification of Sarsaparilla samples

Using new sequence information obtained from nanopore sequencing, chloroplast regions other than available barcode markers were targeted for primer and probe design. As per NCBI taxonomy both *D. hamiltonii* and *H. indicus* belong to Apocynaceae. Accordingly, *Cynanchum auriculatum,* which also belong to the same family was used as reference for assembling chloroplast contigs for both species, using the Read Mapper in CLC Genomics Workbench program. Although full length contiguous chloroplast genome was not assembled for both species, contigs long enough (2–54 kb) to identify variable regions were assembled. These contigs were aligned with chloroplast sequences of *Smilax china* and *Pteridium aquilinum* to detect the diagnostic regions characteristic for each species. Six primer sets were designed from these alignments (Table 1). All the six primer sets were tested on DNA samples of *Decalepis hamiltonii*, *Hemidesmus indicus*, *Pteridium aquilinum* and *Smilax* spp. using conventional PCR. Sequences derived from these samples were used to design probes for testing samples collected from various phases of supply chain.

**Table 1:** New chloroplast markers designed from genome skimming data of *D. hamiltonii* and *H. indicus*, and their sequencing success in Sarsaparilla products.

Primer Name	Sequence	Amplicon size (bp)	Samples																							
			Reference samples				Samples from manufacturing units																	Retail samples		
			75NAT	Pteaq_51470	SR140	SR281	461NW	867NW	BRM278	BRM391	44PR	67NW	104NW	223BI	226BI	295NW	341BI	342BI	357BI	379BI	335BI	336BI	337BI	Amasar1	Amasar2	Amasar3
Decha1	5′-GATTTCGCCAAGTCGATTCT-3'	361	+	+	+	+	+	+	+	+	−	−	−	−	−	−	−	−	−	−	+	+	+	+	+	+
	5′-CCAACGGATTACACCTAGCAA-3'																									
Decha2	5′-TCCCTTTTTATCCCTACGAAA-3'	142	−	−	−	−	−	−	−	−	−	−	−	−	−	−	−	−	−	−	−	−	−	−	−	−
	5′-TTTGTTGACCCCATCAGTCA-3'																									
Hemin1	5′-TGACCCGATTTTAGGTGTGG-3'	240	−	+	+	+	+	−	−	+	−	−	−	−	−	−	−	−	−	−	+	+	+	+	+	+
	5′-TGACAATTTCAAACGGACTTTTC-3'																									
Hemin2	5′-GATCCTTGTGAAGCGGAAAG-3'	205	−	−	+	+	−	−	−	−	−	−	−	−	−	−	−	−	−	−	+	+	+	+	+	+
	5′-CCGCACCCAATTTTAAAGAG-3'																									
Hemin3	5′-CGATTTCCGCTCGTTAATACA-3'	173	+	+	+	+	+	+	+	+	−	−	−	−	−	−	−	−	−	−	+	+	+	+	+	+
	5′-GGGATCAAATGGCTGTTCAT-3'																									
Hemin4	5′-TGCGCTATTCATGGTGCTAC-3'	151	+	+	+	+	+	+	+	+	−	−	−	−	−	−	−	−	−	−	−	−	−	−	−	−
	5′-ACCCCAAAGATTTGTGACCA-3'																									

### Sarsaparilla supply chain verification

For supply chain verification, well characterized reference samples of known provenance included Pteaq_51470 (*P. aquilinum*), 75NAT (*S. artistolochiifolia*), SR140 (*D. hamiltonii*) and SR281 (*H. indicus*). They were obtained as part of NHP Research Alliance collection and from OAC/BIO herbarium collection. The following molecular diagnostic methods were utilized on all the same samples in this study of which the results were not congruent.

### Conventional PCR

Primers tested using conventional PCR couldn’t differentiate market samples clearly. Decha 2 primer did not amplify any of the samples tested including references. None of the primers tested were able to amplify the samples 44PR, 67NW, 104NW, 223BI, 226BI, 295BI, 341BI, 342BI, 357BI and 379BI collected from manufacturing units. Primers Decha1, Hemin3 and Hemin4 successfully amplified and yielded sequences for all reference samples tested. Hemin1 amplified three reference samples but didn’t amplify 75NAT whereas Hemin2 amplified only two reference samples, SR140 and SR281. Primers Hemin1 and Hemin3 amplified all three samples purchased online along with other five samples (335BI, 336BI, 337BI, 461NW and BRM391) collected from a manufacturing unit, in addition Hemin3 amplified market samples 461NW and BRM391. Primers Decha1, Hemin2 amplified the market samples 335BI, 336BI, 337BI, Amasar1 Amasar2 and Amasar3 along with reference material mentioned above, in addition Decha1 amplified market samples 461NW, 867NW, BRM278 and BRM391. All together, primers tested were only able to amplify seven (335BI, 336BI, 337BI, 461NW, 867NW, BRM278 and BRM391) out of 17 samples collected from manufacturing units and three samples (Amasar1, Amasar2 and Amasar3) purchased from an online store and four reference samples. However, sequence analysis revealed that variable SNPs were identified only in sequences derived from three primer pairs, Decha1, Hemin1 and Hemin2 but no variable regions were identified in the amplicons produced by the remaining two primer pairs Hemin3 and Hemin4. Decha1 amplicon has only one SNP whereas Hemin1 and Hemin2 amplicons have two and four SNPs respectively. Three variable primers clearly differentiated *H. indicus* from all other samples. Decha1 and Hemin1 couldn’t differentiate *D. hamiltonii* from *P. aquilinum* and as a result, it was difficult to identify market samples 335BI, 336BI, 337BI, Amasar1, Amasar2 and Amasar3 precisely. Hemin2 couldn’t amplify the references *P. aquilinum* and *S. artistolochiifolia,* but simultaneously it clearly differentiated *D. hamiltonii* from *H. indicus* and as a result, all the market samples were identified as *D. hamiltonii*. These results clearly didn’t confirm the identity of market samples unequivocally. To overcome this limitation, we designed hydrolysis probes specific for each of the four species and tested the market samples again.

### Probe-based assays on portable qPCR device

**Figure 1:**
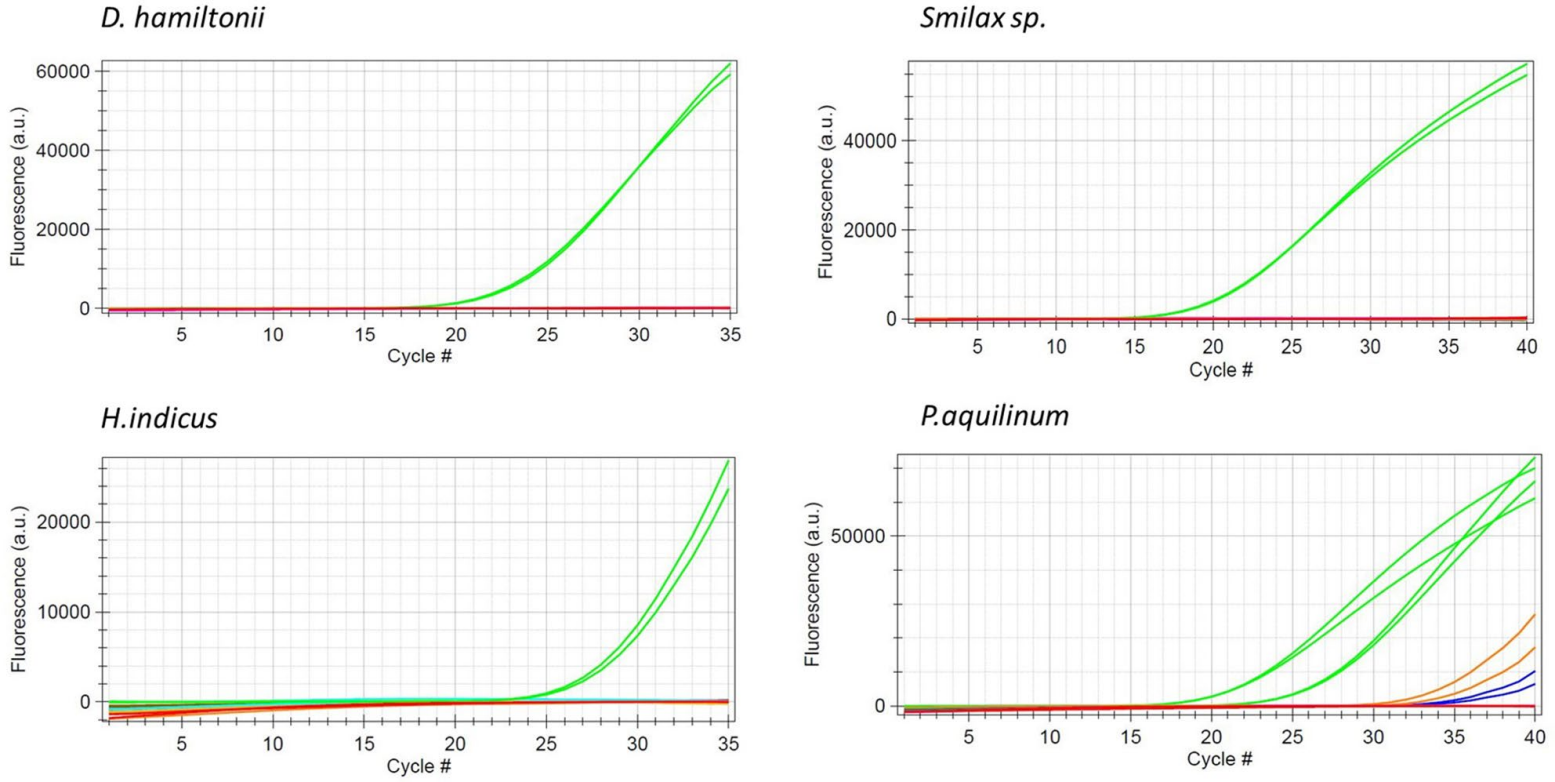
Amplification curves of four probe-based assays showing the amplification of their respective target species and non-amplification of non-target species.

Unlike conventional PCR, probe-based assays designed for each Sarsaparilla species clearly identified market samples. Chloroplast contigs obtained through genome skimming of *D. hamiltonii* and *H. indicus* samples, were used for development of diagnostic assays of these species. Using the new sequence information obtained from MinION, probe-based assays developed specifically for *D. hamiltonii* and *H. indicus* were used for testing the market samples. Similarly, probes were designed for *Smilax* spp.and *P. aquilinum* using the rbcL sequences archived at the NHP Research Alliance sequence reference library. All the four probes were tested on seventeen samples collected from manufacturing units and three samples bought online (Table 2). Originally labelled as *H. indicus*, none of the three samples (335BI, 336BI and 337BI) collected from manufacturing unit were amplified by *H. indicus* probe but instead amplified by *D. hamiltonii*. Similarly, one *H. indicus* sample (Amasar 3) bought online amplified by *P. aquilinum* probe suggesting a clear case of adulteration at these two stages. Also, two other online samples Amasar 1 and Amasar 2, originally labelled as *Smilax ornata* and organic Sarsaparilla root respectively, were amplified by *D. hamiltonii* probe suggesting all three samples bought online (retail) did not confirm with the species listed on their labels. Out of the remaining fourteen samples, eight (44PR, 67NW, 295NW, 341BI, 357BI,379BI, 867NW and BRM278) were amplified by *Smilax* spp. probe and six (104NW, 223BI, 226BI, 342BI, 461BI and BRM391) were amplified by *P. aquilinum* probe appropriately as suggested by their labels. All the positive samples were amplified by their respective probes (Fig. 1).

**Table 2:** Performance of four probe-based assays designed for authentication of Sarsaparilla products.

Sample ID	Commercial description	Probe test results			
		*D. hamiltoniii*	*H. indicus*	*P. aquilinum*	*Smilax* sp.
75NAT	*Smilax aristolochiifolia*	−	−	−	+
Pteaq_51470	*Pteridium aquilinum*	−	−	+	−
SR140	*Decalepis hamiltonii*	+	−	−	−
SR281	*Hemidesmus indicus*	−	+	−	−
104NW	*Pteridium aquilinum*	−	−	+	−
223BI	*Pteridium aquilinum*	−	−	+	−
341BI	*Smilax aristolochiifolia*	−	−	−	+
379BI	Sarsaparilla root	−	−	−	+
335BI	*Hemidesmus indicus*	+	−	−	−
336BI	*Hemidesmus indicus*	+	−	−	−
337BI	*Hemidesmus indicus*	+	−	−	−
Amasar1	*Smilax ornata*	+	−	−	−
Amasar2	Organic Sarsaparilla root	+	−	−	−
Amasar3	*Hemidesmus indicus*	−	−	+	−
44PR	*Smilax officinalis*	−	−	−	+
67NW	*Smilax aristolochiifolia*	−	−	−	+
295NW	*Smilax aristolochiifolia*	−	−	−	+
342BI	*Pteridium aquilinum*	−	−	+	−
226BI	*Pteridium aquilinum*	−	−	+	−
BRM391	*Pteridium aquilinum*	−	−	+	−
461NW	*Pteridium aquilinum*	−	−	+	−
867NW	*Smilax aristolochiifolia*	−	−	−	+
357BI	*Smilax aristolochiifolia*	−	−	−	+
BRM278	*Smilax aristolochiifolia*	−	−	−	+

‘+’ indicates positive amplification and ‘−’ indicates no amplification.

### Chemical fingerprints and clustering

**Figure 2:**
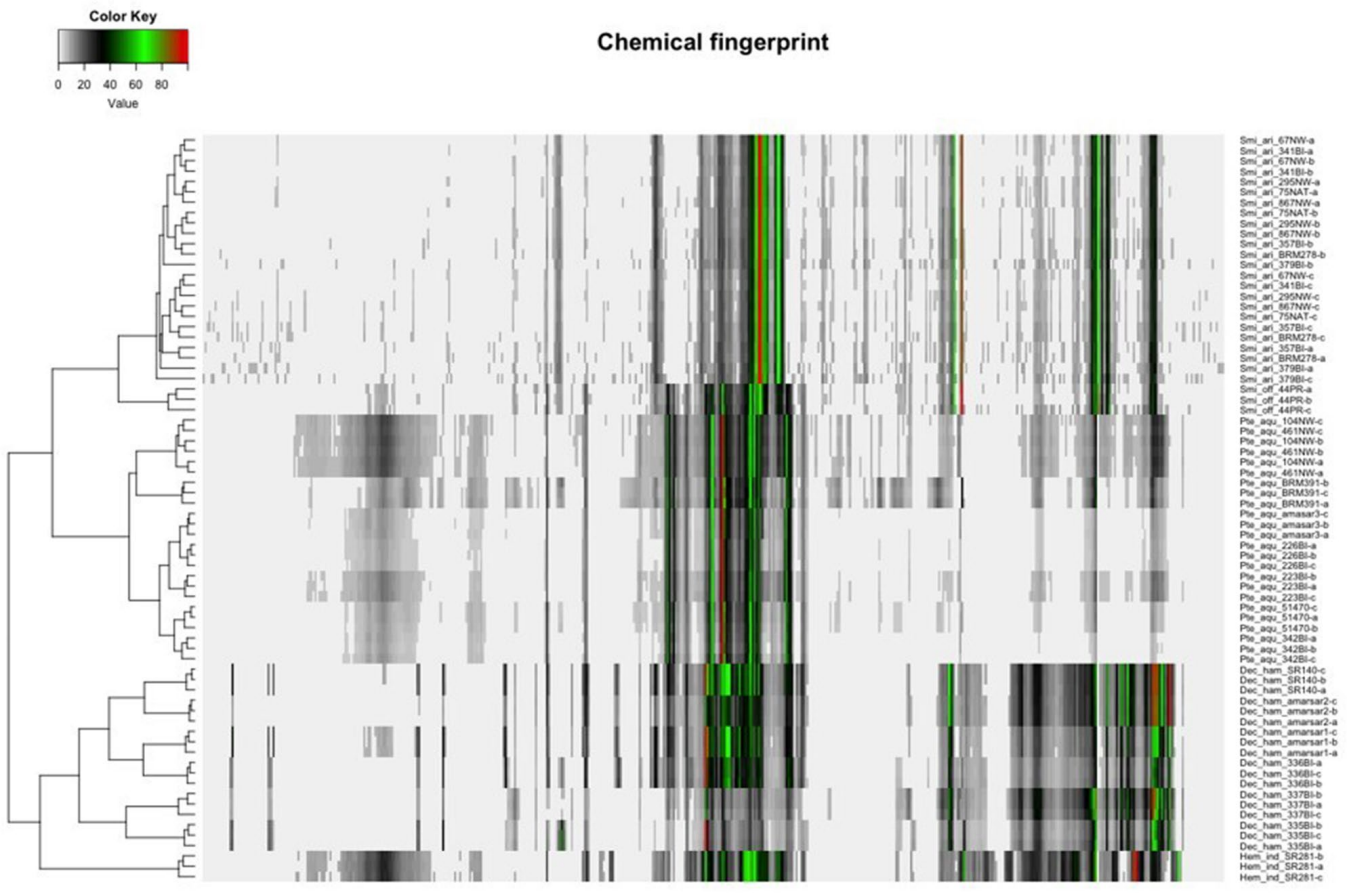
Hierarchically clustered chemical fingerprints.

**Figure 3:**
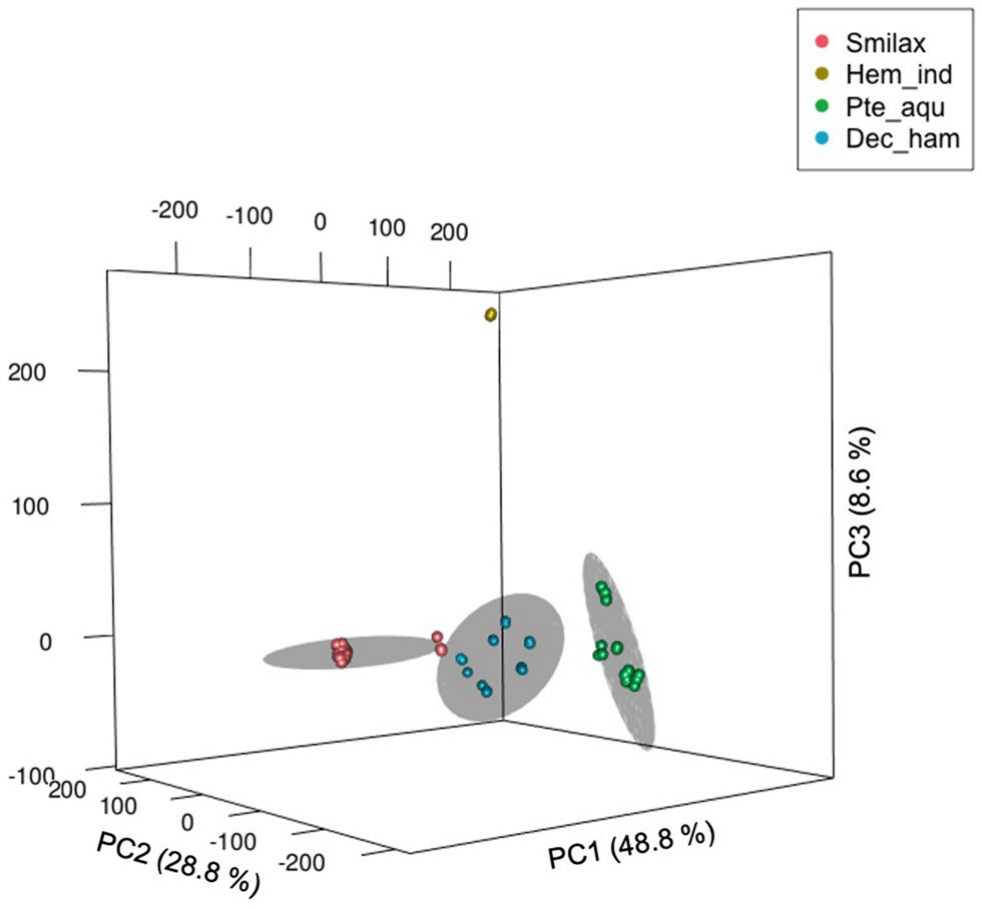
HCPC cluster map with ellipsoids of 95% confidence level for the clear visualization (cluster Hem_ind is with lesser points for ellipsoid).

**Figure 4:**
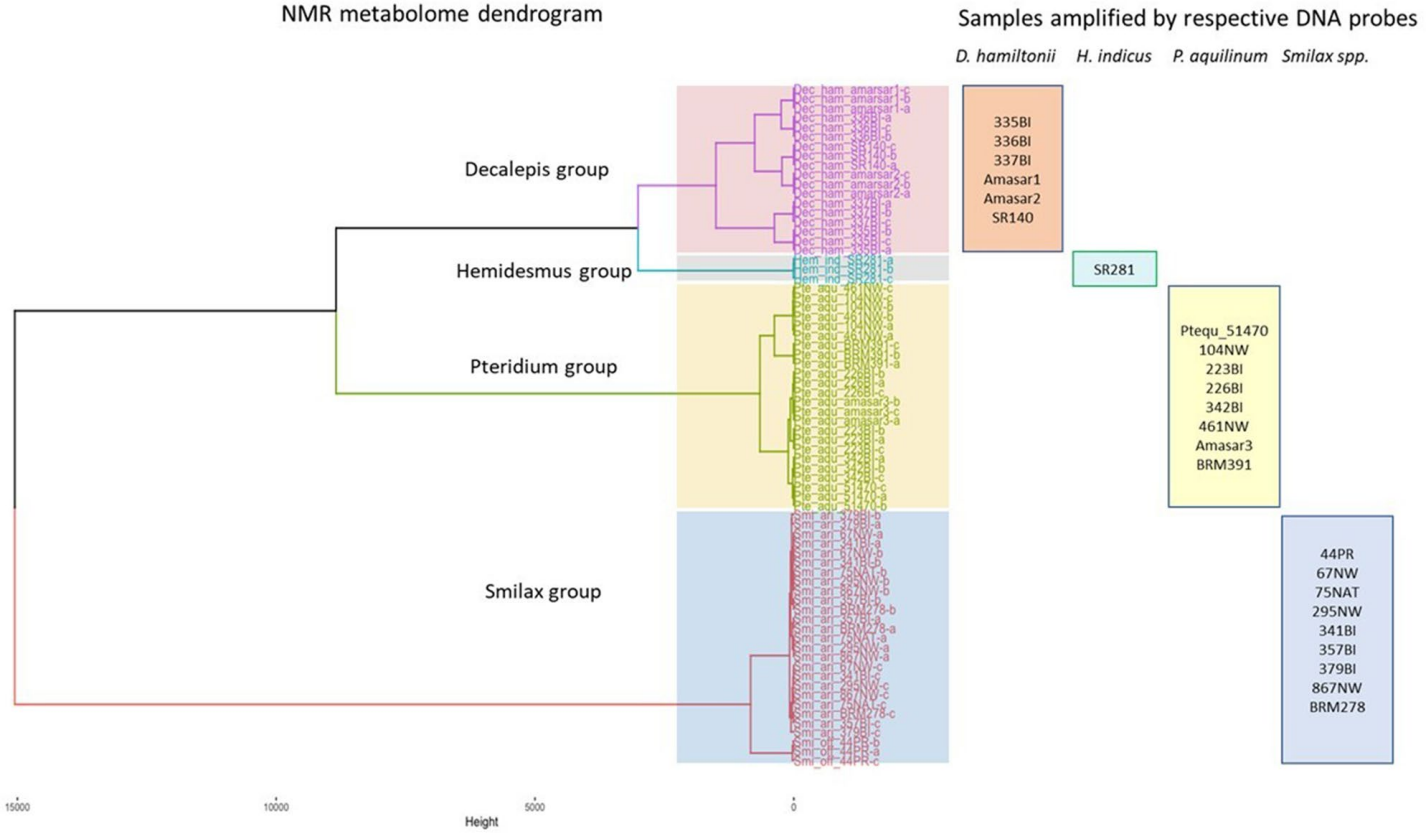
Comparison showing the similar pattern of Sarsaparilla product authentication obtained by both metabolomic and probe-based approach.

In this study, we have included 24 samples belonging to four species of Sarsaparilla group to determine the classification and grouping using chemometric modelling of ^1^H NMR. In trade, Sarsaparilla group belongs to four different taxonomically distant groups. The chemical fingerprint (Fig. 2) shows how the samples are clustered (HCA) based on their metabolite diversity and its intensity. There is a clear metabolite difference between the four different taxa, which separated all the samples in ordination space in accordance to their respective taxonomic classification (Fig. 3). The clusters are mapped in the space by the first three principal components (PC1 to PC3), which explain 86.2% of the overall variance (Fig. 3). Ellipsoids in the cluster (Fig. 3) map are of 95% confidence levels to provide visual observation of separated groups. The area of the cluster Dec_ham (*D. hamiltonii*) is higher than other clusters, this might be possible due to the differences in processing methods of the products. The outlier in the cluster Smilax, is the sample labeled as *S. officinalis* (44PR) all other are *S. aristolochiifolia.* However, all the samples are taxonomically grouped accordingly with the reference materials and aligned with genomic clustering (Fig. 4).

## Discussion

We demonstrated for the first time that the oxford Nanopore can be used for quality assurance of authentic ingredients in the natural health products industry. The MinION provided useful sequence data at a reasonable cost. Genome skimming allows for shallow sequencing of high copy regions of a genome such as chloroplast, mitochondria and ribosomal DNA, which are useful for taxonomic identification. Oxford nanopore’s MinION device is a handheld, portable sequencing device that can be used for genome sequencing in a limited resource-setting^25^. In the current study, we used MinION for genome skimming of *Decalepis hamiltonii* and *Hemidesmus indicus* both of which have limited sequence information available on Genbank. This approach allowed us to generate new sequence information for the Red listed medicinal species *D. hamiltonii* at a fraction (~ 5%) of other NGS sequencing platforms. A previous study used MinION for identifying CITES-listed shark species using genome skimming approach^26^. Capital cost involved in buying a MinION instrument is highly affordable. Currently Oxford Nanopore Technology (ONT) is offering a MinION enhanced package for $4500 USD, which includes one MinION device, 8 flow cells, 2 sequencing kits and 1wash kit. Using this package, it is possible to complete genome skimming of 8 samples that have genome size of around 1 gb with an average of 10 × to 15 × coverage and this would still leave one sequencing kit unused. Conversely, it costs more than $125,000 USD to buy an NGS instrument and few thousand dollars more to buy the reagents. Although low cost is a trade-off for high error rate of the reads obtained with MinION, long contiguous reads we obtained that have length of up to 54 kb are of enough size to develop new markers of 200–400 bp long, when a closely related chloroplast genome is available.

Using a probe-based technology has several advantages compared to traditional primer-based methods for species identification in herbal products. The qPCR method can be validated^27^ and is fit for purpose in identity testing species ingredients. The barcoding approach has several disadvantages such as (1) does not work well on finished products because the DNA fragments are smaller than the barcode regions. (2) Resource intensive (times and costs). (3) Primers designed in conserved regions tend to amplify few to many non-target species depending on the level of sequence conservation. (4) Does not allow proper assay design with proper positive controls. (5) Cannot be properly validated for sensitivity (e.g., LOD/LOQ), and (6) several post-PCR processing steps such as running the gel, sequencing of amplicons and analysis of sequence information are necessary to identify the target species. Conversely, using real-time PCR, which monitors the amplification of target DNA in real-time using fluorescent dye, invalidates the requirement for post-PCR processing and provides quick species identification. This becomes even more useful when hundreds of samples to be tested and provide the authentication results to the industry within a specified time. In addition, having a portable qPCR device on-site further reduces the necessity to ship samples to a specified testing location and provide even quicker results. Therefore, we developed probe-based assays for four species of Sarsaparilla group and tested them successfully on a portable qPCR device.

Understanding the chemical composition of a phytomedicine is vital for providing safe consumer products. This is especially important for products labelled as Sarsaparilla that may contain taxonomic species and adulterants with similar morphological characteristics but with different chemical composition and pharmacological efficacy than original Sarsaparilla group. The botanical industry has been faced with these problems for a long time. Notably, these issues need to be highlighted and addressed to both botanical industries and consumers of natural health products. For example, Srirama et al.^28^ reported that *Phyllanthus debilis* and *Phyllanthus amarus* are chemically different but biologically equivalent. Their study suggested that hepatoprotective property was found in *P.amarus* but not in *P. debilis*. In contrast, *Saraco asoca* which is used in gynecological disorders and its adulterant *Polyalthia longifolia* have similar chemical compositions but different biological effects^29^. In our study, the ‘Sarsaparilla’ is one such botanical entity that poses problems in terms of field identification by localized suppliers. This Sarsaparilla name designates the following botanicals in the industry: *Smilax* spp., *Pteridium aquilinum* (North American Sarsaparilla), *Decalepis hamiltonii* and *Hemidesmus indicus* (South East Indian Sarsaparilla). These botanicals are taxonomically different entities and traded and used as bio-active equivalents/substitutes. Conventional Pharmacognosy techniques based on macro-morphological characters may not be effective in distinguishing the four botanicals traded as “Sarsaparilla” if it is in a processed form such as root fragments or powdered extracts. In this study, we demonstrated that using both genomic and metabolomic approaches provide robust authentication for Sarsaparilla products. Our results present a combined approach that produced very similar results in segregating the four species in Sarsaparilla group (Fig. 4). Either of these tools could be used for the verification of Sarsaparilla species ingredients in raw or finished natural health products.

## Methods

### Procurement of Sarsaparilla samples

**Figure 5:**
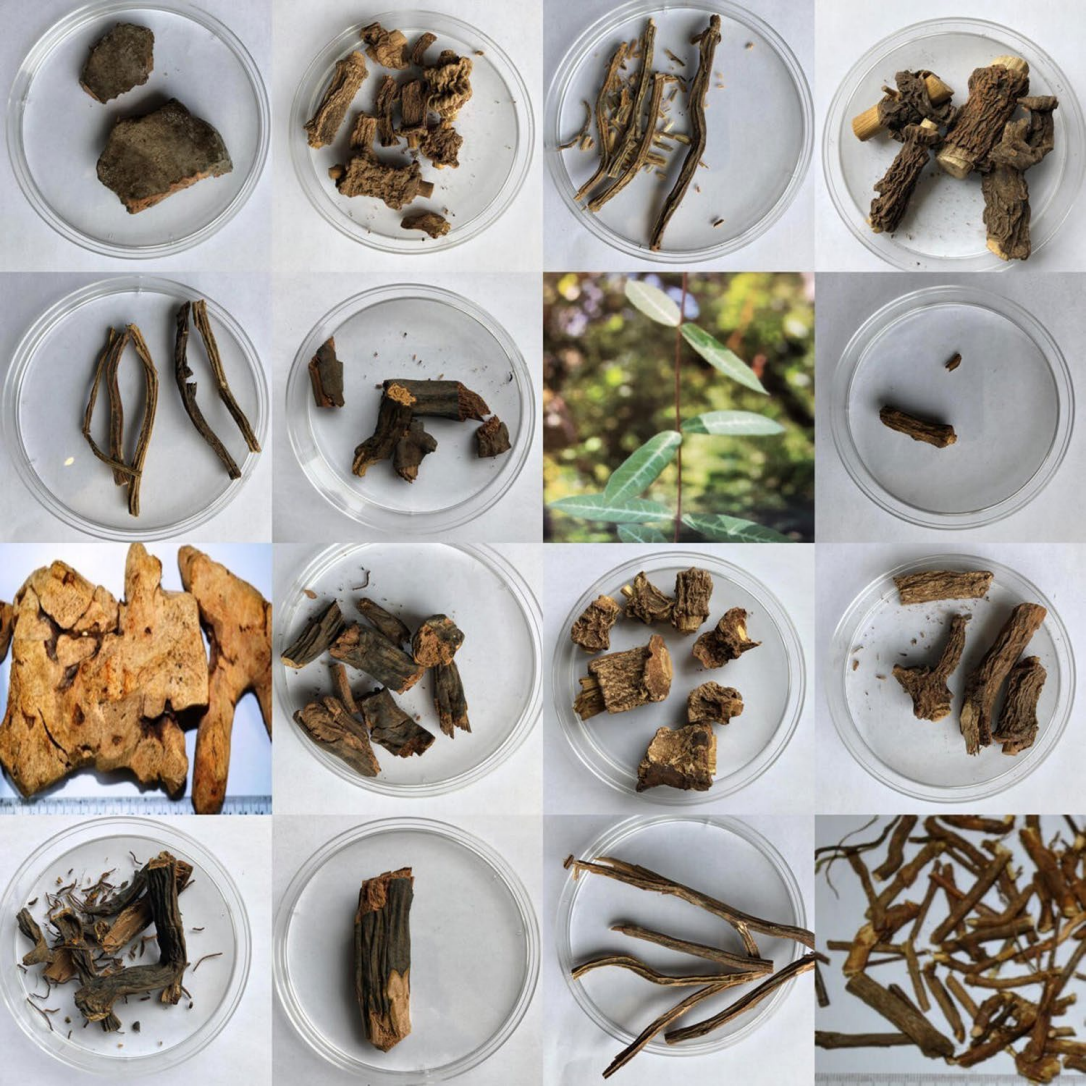
A snapshot of Sarsaparilla root morphology.

A subset of 21 samples were used for this study, out of a larger collection, obtained from different parts of the industry supply chain through the Natural Health Products Research Alliance (NHPRA), University of Guelph, Canada (Fig. 5). In total, 24 samples, which includes market survey samples of all the commercial Sarsaparilla taxa of known provenance were used in this study. The list includes one sample each of *Decalepis hamiltonii* (SR140), *Smilax officinalis* (44PR) and *Smilax ornata* (Amasar1); Five samples of *Hemidesmus indicus* (Amasar3, SR281, 335BI, 336BI and 337BI); Seven samples each of *Smilax aristolochiifolia* (67NW, 75NAT, 295NW, 341BI, 357BI, 867NW and BRM278) and *Pteridium aquilinum* (104NW, 223BI, 226BI, 342BI, 461NW, BRM391 and Pteaq_51470); Two samples were just listed as Sarsaparilla root (379BI and Amasar2). Herbarium vouchers for reference material sequences and NMR profiles were validated by professional taxonomists and archived at the OAC Herbarium, University of Guelph.

### Sample preparation

The standard sample preparation for NMR processing required 50 mg tissues to be homogenized using mortar and pestle in liquid nitrogen and dissolved in 1 ml of deuterated methanol (CD3OD). All the samples were prepared in triplicate. The solvent was chosen for its greater solubility towards diverse chemical compounds. Samples were incubated in the sonicating bath for 30 min at room temperature. Sonicated samples were centrifuged for 5 min at 3000 rpm, and then 600 μl of clear supernatant was collected in a 5 mm Wilmad^®^ NMR tube.

The same samples used above for NMR extractions were also used for DNA extraction. A final volume of 100 µl DNA was extracted from samples using Nucleospin Plant II kit of Macherey–Nagel following the manufacturer’s protocol.

### Spectra acquisition for NMR

To analyse chemical fingerprints of the samples 1H-NMR spectra were acquired using 600 MHz Bruker Avance III NMR equipped with a 5 mm “TXI” room temperature probe. To acquire data, we used Bruker pulse program “zgpr” and the acquisition mode “DQD” including the following parameters as follows: number of complex points, 28,061; dummy scans, 4; number of scans, 64; acquisition time, 2.27 s; delay time, 12.73 s; spectral width, 12,345 Hz; fid resolution, 0.4399 Hz. We wanted to differentiate species using their metabolites and to attain this we used the standard pulse program to keep the experiment simple. The parameters are chosen as good trade-off for the information acquired enough to differentiate species, time duration of acquisition and the expertise.

### Data processing and analysis for NMR

1H-NMR spectra were processed using TopSpin 4.0.7. Phase and baseline were corrected automatically except for the two samples (Pte_aqu_BRM391-b and Dec_ham_337BI-b). These two were having problem with baseline and was corrected manually using fourth-order polynomial algorithm. The corrected samples were well grouped with their replicates. Spectra were calibrated to the residual methanol peak as 3.31 ppm. Processed spectra were bucketed with simple rectangular buckets of positive intensities without scaling (AMIX 4.0.1). The chemical range utilized for bucketing was − 1 to 12 ppm, with a width of 0.01 ppm. While bucketing, the residual solvent signals of water and methanol were removed at the regions 4.75–5.06 and 3.16–3.45 ppm, respectively. After bucketing, each spectrum was normalized by setting below means as 0 and above means were binned from 1 to 100. All the spectra were analysed and clustered by the Hierarchical Clustering Analysis (HCA) and Hierarchical Cluster on Principal Compounds (HCPC) using Euclidian dissimilarity matrix and Ward’s clustering method using the R package FactoMineR^30^. The spectral intensities of chemical compounds were converted to the chemical fingerprints and were hierarchically clustered (HCA).

### DNA barcoding and conventional PCR

Samples were tested using CBOL Plant Working Group^9^ recommended DNA regions rbcL, and matK as well as new primers designed in this study. The selected loci were amplified by polymerase chain reaction (PCR) on a PTC–100 thermocycler (Bio-Rad). DNA was amplified in 20 μL reaction mixtures containing 1 U AmpliTaq Gold Polymerase with GeneAmp 106 PCR buffer II (100 mm Tris–HCl pH 8.3, 500 mm KCl) and 2.5 mm MgCl2 (Applied Biosystems), 0.2 mm dNTPs, 0.1 mm of each primer (0.5 mm for matK), and 20 ng template DNA. Amplified products were sequenced for new markers in both directions with the primers used for amplification, following the protocols of the University of Guelph Genomics facility (www.uoguelph.ca/~genomics). Products from each specimen were cleaned using Sephadex columns and run on an ABI 3730 sequencer (Applied Biosystems). Bidirectional sequence reads were obtained for all the PCR products. Sequences were assembled using Sequencher 4.5 (Gene Codes Corp), and aligned manually using Bioedit version 7.0.9.

### Library preparation and sequencing on the MinIon

DNA quantity was measured using a Qubit spectrophotometer and fragment size of the genomic DNA was measured with Agilent’s TapeStation instrument. Genomic DNA was used for library preparation using 1D Genomic DNA by Ligation (SQK-LSK109) protocol of Oxford Nanopore Technologies. Flow Cell was attached to the MinION device and priming was completed as per the above protocol, the library containing adapter-ligated high molecular weight genomic fragments was loaded on to the flow cell and the sequencing run was run with default settings for 24 h. Throughout the run, raw sequencing reads were collected in minIT in both FASTQ and Fast5 formats while FASTQ files were used for data analysis.

### Bioinformatic analyses

For assembling chloroplast genome, FASTQ files were uploaded into CLC Genomics Workbench software version 12.0.2. Chloroplast sequences were mapped directly to the reference *Cynanchum auriculatumn* chloroplast sequences from Genbank using CLC Workbench Read mapper, other genomic contigs were assembled using CANU by setting the genome size as 15 kb while discarding the reads shorter than 100 bp.

### Primer, probe design and PCR amplification

Chloroplast contigs obtained for *D. hamiltonii* and *H. indicus* were aligned with chloroplast sequences of *Smilax china*, *Pteridium aquilinum* available at the NHP Research Alliance sequence reference library. Variable genomic regions surrounded by conserved nucleotide blocks were selected for primer and probe design. Six primer sets and two probe assays one each for *D. hamiltonii* and *H. indicus* were designed from this alignment. Probes for *Smilax* sp. and *P. aquilinum* were designed from the alignment of rbcL gene sequences available at the NHP Research Alliance sequence reference library.

## Supplementary information

Supplementary Information 1.

Supplementary Information 2.

## Data Availability

All data generated or analysed during this study are included in this published article (and its Supplementary Information files 1).
