# Supplementary material for: Genome skimming and NMR chemical fingerprinting provide quality assurance biotechnology to validate Sarsaparilla identity and purity
Source: Sci Rep. 2020 Nov 5;10:19192. doi: 10.1038/s41598-020-76073-7 (PMC7645426; doi:10.1038/s41598-020-76073-7)
Supplement: Supplementary file 2 — Supplementary Information 2. [file 41598_2020_76073_MOESM2_ESM.docx]

**Genome skimming and NMR Chemical Fingerprinting provide Quality Assurance Biotechnology to Validate Sarsaparilla Identity and Purity**

Prasad Kesanakurti^*^, Arunachalam Thirugnanasambandam, Subramanyam Ragupathy and Steven G Newmaster

NHP Research Alliance, College of Biological Sciences, University of Guelph, Guelph, Ontario, Canada.

^*^Corresponding author contact: Prasad Kesanakurti [kesanakv@uoguelph.ca](mailto:kesanakv@uoguelph.ca)

Real-time PCR conditions:

## Target species

***Decalepis hamiltonii***

Reactions assembly recipe

| SensiFAST Probe No-ROX mix (2x) | 10 µl |
| --- | --- |
| Primer and Probe (10x) | 1 µl |
| DMSO | 0.5 µl |
| DNA | 1 µl |
| H_2_O DEPC | 7.5 µl |
| Final volume: 20 µl | |

## Primer and probe information

| Primer FWD | 5’- GGA GTA CCT ATG TCG ATC ATG C-3’ |
| --- | --- |
| Primer REV | 5’- CTT GTG AAG CGG AAA GGG A-3’ |
| Probe | /56-FAM/TTA TTA GGA /ZEN/GGG CCG TGC AGA TCC /3IABkFQ/ |

## Thermal protocol

95°C 5 min

95°C 10 sec

X35 cycles

65°C 20 sec

## Target species

***Hemidesmus indicus***

Reactions assembly recipe

| SensiFAST Probe No-ROX mix (2x) | 10 µl |
| --- | --- |
| Primer and Probe (10x) | 1 µl |
| DMSO | 0.5 µl |
| DNA | 1 µl |
| H_2_O DEPC | 7.5 µl |
| Final volume: 20 µl | |

## Primer and probe information

| Primer FWD | 5’- GTC GAT CAT GCA CCT GAA TTT AC-3’ |
| --- | --- |
| Primer REV | 5’- CTT GTG AAG CGG AAA GGG A -3’ |
| Probe | /56-FAM/AAA GGA GAG /ZEN/CCG TGC AGA TCC AG/3IABkFQ/ |

## Thermal protocol

95°C 5 min

95°C 10 sec

X35 cycles

65°C 20 sec

## Target species

***Smilax spp.***

Reactions assembly recipe

| SensiFAST Probe No-ROX mix (2x) | 10 µl |
| --- | --- |
| Primer and Probe (10x) | 1 µl |
| DNA | 1 µl |
| H_2_O DEPC | 8 µl |
| Final volume: 20 µl | |

## Primer and probe information

| Primer FWD | 5’- TTT GAA GAA GGC TCG GTT ACT -3’ |
| --- | --- |
| Primer REV | 5’- GGT GGG CCT TGG AAA GT-3’ |
| Probe | /56-FAM/TGG TTT CAA /ZEN/AGC CCT ACG AGC TCT /3IABkFQ/ |

## Thermal protocol

95°C 5 min

95°C 10 sec

X40cycles

65°C 20 sec

## Target species

***Pteridium aquilinum***

Reactions assembly recipe

| SensiFAST Probe No-ROX mix (2x) | 10 µl |
| --- | --- |
| Primer and Probe (10x) | 1 µl |
| DNA | 1 µl |
| H_2_O DEPC | 8 µl |
| Final volume: 20 µl | |

## Primer and probe information

| Primer FWD | 5’- TTA GCA GCC TTC CGA ATG AC -3’ |
| --- | --- |
| Primer REV | 5’- TTG TAG CGA TCA AGA CTG GTA AG -3’ |
| Probe | /56-FAM/CCA TGT ACC /ZEN/CGT GGA GGA TTC TGC /3IABkFQ/ |

## Thermal protocol

95°C 5 min

95°C 10 sec

X40cycles

65°C 20 sec

***D. hamiltonii* and *H. indicus* primers tested as follows:**

Reactions assembly recipe

| 10x Pfu Buffer + MgSO4 | 2.5ul |
| --- | --- |
| DNTPs (2mM) | 1.5ul |
| Trehalose (10%) | 1ul |
| Taq polymerase | 0.1ul |
| Forward primer (1uM) | 2ul |
| Reverse primer (1uM) | 2ul |
| Molecular Biology grade water | 7.9ul |
| DNA template | 3ul |
| Final volume: 20 µl | |

## Thermal protocol

Initial Denaturation: 95^0^C for 5min

Denaturation: 95^0^C for 1min

X40cycles

Annealing: 51^0^C for 30sec

Extension: 72^0^C for 30sec

Final extension: 72^0^C for 5min

Hold: 4^0^C

Sequences amplified by Decha1 marker:

>335BI_Decha1

AGTCGATTCTTAGGAAATGTCGAATCAAACCATTTATCCTTATTTCAACAAAAGAAGCCCGGGCTTCTTCGCCAGAAGAACTTTTTTTGTCTTTGTCCCAATTCAATACTAAACAAGTCCGAACTAATTGAATAGTTGTGTCATAAATTCCTCGAATTGGTTTGCCGTTTCCATTTCCATAAAGGATATAATTGATAACTCGAAGTTGCACAATATCTCTTTCCTGCAACATATCGGGGGGGAAAAGTGTTGCTAAATTTTGACCGTCCGTTATTTCATATGTGACGACAGGTCGAACCAAAACAAAATGCTTTTTTTT

>336BI_Decha1

AGTCGATTCTTAGGAAATGTCGAATCAAACCATTTATCCTTATTTCAACAAAAGAAGCCCGGGCTTCTTCGCCAGAAGAACTTTTTTTGTCTTTGTCCCAATTCAATACTAAACAAGTCCGAACTAATTGAATAGTTGTGTCATAAATTCCTCGAATTGGTTTGCCGTTTCCATTTCCATAAAGGATATAATTGATAACTCGAAGTTGCACAATATCTCTTTCCTGCAACATATCGGGGGGGAAAAGTGTTGCTAAATTTTGACCGTCCGTTATTTCATATGTGACGACAGGTCGAACCAAAACAAAATGCTTTTTTTT

>337BI_Decha1

AGTCGATTCTTAGGAAATGTCGAATCAAACCATTTATCCTTATTTCAACAAAAGAAGCCCGGGCTTCTTCGCCAGAAGAACTTTTTTTGTCTTTGTCCCAATTCAATACTAAACAAGTCCGAACTAATTGAATAGTTGTGTCATAAATTCCTCGAATTGGTTTGCCGTTTCCATTTCCATAAAGGATATAATTGATAACTCGAAGTTGCACAATATCTCTTTCCTGCAACATATCGGGGGGGAAAAGTGTTGCTAAATTTTGACCGTCCGTTATTTCATATGTGACGACAGGTCGAACCAAAACAAAATGCTTTTTTTT

>Amasar1_Decha1

AGTCGATTCTTAGGAAATGTCGAATCAAACCATTTATCCTTATTTCAACAAAAGAAGCCCGGGCTTCTTCGCCAGAAGAACTTTTTTTGTCTTTGTCCCAATTCAATACTAAACAAGTCCGAACTAATTGAATAGTTGTGTCATAAATTCCTCGAATTGGTTTGCCGTTTCCATTTCCATAAAGGATATAATTGATAACTCGAAGTTGCACAATATCTCTTTCCTGCAACATATCGGGGGGGAAAAGTGTTGCTAAATTTTGACCGTCCGTTATTTCATATGTGACGACAGGTCGAACCAAAACAAAATGCTTTTTTTT

>Amasar2_Decha1

AGTCGATTCTTAGGAAATGTCGAATCAAACCATTTATCCTTATTTCAACAAAAGAAGCCCGGGCTTCTTCGCCAGAAGAACTTTTTTTGTCTTTGTCCCAATTCAATACTAAACAAGTCCGAACTAATTGAATAGTTGTGTCATAAATTCCTCGAATTGGTTTGCCGTTTCCATTTCCATAAAGGATATAATTGATAACTCGAAGTTGCACAATATCTCTTTCCTGCAACATATCGGGGGGGAAAAGTGTTGCTAAATTTTGACCGTCCGTTATTTCATATGTGACGACAGGTCGAACCAAAACAAAATGCTTTTTTTT

>Amasar3_Decha1

AGTCGATTCTTAGGAAATGTCGAATCAAACCATTTATCCTTATTTCAACAAAAGAAGCCCGGGCTTCTTCGCCAGAAGAACTTTTTTTGTCTTTGTCCCAATTCAATACTAAACAAGTCCGAACTAATTGAATAGTTGTGTCATAAATTCCTCGAATTGGTTTGCCGTTTCCATTTCCATAAAGGATATAATTGATAACTCGAAGTTGCACAATATCTCTTTCCTGCAACATATCGGGGGGGAAAAGTGTTGCTAAATTTTGACCGTCCGTTATTTCATATGTGACGACAGGTCGAACCAAAACAAAATGCTTTTTTTT

>BRM391_Decha1

AGTCGATTCTTAGGAAATGTCGAATCAAACCATTTATCCTTATTTCAACAAAAGAAGCCCGGGCTTCTTCGCCAGAAGAACTTTTTTTGTCTTTGTCCCAATTCAATACTAAACAAGTCCGAACTAATTGAATAGTTGTGTCATAAATTCCTCGAATTGGTTTGCCGTTTCCATTTCCATAAAGGATATAATTGATAACTCGAAGTTGCACAATATCTCTTTCCTGCAACATATCGGGGGGGAAAAGTGTTGCTAAATTTTGACCGTCCGTTATTTCATATGTGACGACAGGTCGAACCAAAACAAAATGCTTTTTTTT

>Pteaq_51470_Decha1

AGTCGATTCTTAGGAAATGTCGAATCAAACCATTTATCCTTATTTCAACAAAAGAAGCCCGGGCTTCTTCGCCAGAAGAACTTTTTTTGTCTTTGTCCCAATTCAATACTAAACAAGTCCGAACTAATTGAATAGTTGTGTCATAAATTCCTCGAATTGGTTTGCCGTTTCCATTTCCATAAAGGATATAATTGATAACTCGAAGTTGCACAATATCTCTTTCCTGCAACATATCGGGGGGGAAAAGTGTTGCTAAATTTTGACCGTCCGTTATTTCATATGTGACGACAGGTCGAACCAAAACAAAATGCTTTTTTTT

>461NW_Decha1

AGTCGATTCTTAGGAAATGTCGAATCAAACCATTTATCCTTATTTCAACAAAAGAAGCCCGGGCTTCTTCGCCAGAAGAACTTTTTTTGTCTTTGTCCCAATTCAATACTAAACAAGTCCGAACTAATTGAATAGTTGTGTCATAAATTCCTCGAATTGGTTTGCCGTTTCCATTTCCATAAAGGATATAATTGATAACTCGAAGTTGCACAATATCTCTTTCCTGCAACATATCGGGGGGGAAAAGTGTTGCTAAATTTTGACCGTCCGTTATTTCATATGTGACGACAGGTCGAACCAAAACAAAATGCTTTTTTTT

>SR140_Decha1

--TCGATTCTTAGGAAATGTCGAATCAAACCATTTATCCTTATTTCAACAAAAGAAGCCCGGGCTTCTTCGCCAGAAGAACTTTTTTTGTCTTTGTCCCAATTCAATACTAAACAAGTCCGAACTAATTGAATAGTTGTGTCATAAATTCCTCGAATTGGTTTGCCGTTTCCATTTCCATAAAGGATATAATTGATAACTCGAAGTTGCACAATATCTCTTTCCTGCAACATATCGGGGGGGAAAAGTGTTGCTAAATTTTGACCGTCCGTTATTTCATATGTGACGACAGGTCGAACCAAAACAAAATGCTTTTTTTT

>SR281_Decha1

--TCGATTCTTAGGAAATGTCGAATCAAACCATTTATCCTTATTTCAACAAAAGAAGCCCGGGCTTCTTCGCCAGAAGAACTTTTTTTGTCTTTGTCCCAATTCAATACTAAACAAGTCCGAACTAATTGAATAGTTGTGTCATAAATTCCTCGAATTGGTTTGCCGTTTCCATTTCCATAAAGGATATAATTGATAACTCGAAGTTGCACAATATCTCTTTCCTGCAACATATCGGGGGGGAAAAGTGTTGCTAAATTTAGACCGTCCGTTATTTCATATGTGACGACAGGTCGAACCAAAACAAAATGCTTTTTTTT

>75NAT_Decha1

AGTCGATTCTTAGGAAATGTTGAATCAAACCCTTTGTCCTTATTTCAACAAAGGAAGCACAGGCTTCTTGACTAGAAGCATTTTTTTTGTCCTGTTCCCAATTCAATACTAAACAAGTCCGAACTAATTGAATATTTGTATCAGAAATTCTTCGAATTAGTTTA------CCATTTCCATAAAGGATATAATTGACAACGCGGAGTTGCACACTATTCCATTGCTGCAAAAAATCCGGGGGGAAAAGCGCTGCTAAATTTATACCGCCCCTTATTTCATATGTTACGACAGGACGAACCAAAGCAAAAGACTTTTTCTT

>867NW_Decha1

AGTCGATTCTTAGGAAATGTTGAATCAAACCCTTTGTCCTTATTTCAACAAAGGAAGCACAGGCTTCTTGACTAGAAGCATTTTTTTTGTCCTGTTCCCAATTCAATACTAAACAAGTCCGAACTAATTGAATATTTGTATCAGAAATTCTTCGAATTAGTTTA------CCATTTCCATAAAGGATATAATTGACAACGCGGAGTTGCACACTATTCCATTGCTGCAAAAAATCCGGGGGGAAAAGCGCTGCTAAATTTATACCGCCCCTTATTTCATATGTTACGACAGGACGAACCAAAGCAAAAGACTTTTTCTT

>BRM278_Decha1

AGTCGATTCTTAGGAAATGTTGAATCAAACCCTTTGTCCTTATTTCAACAAAGGAAGCACAGGCTTCTTGACTAGAAGCATTTTTTTTGTCCTGTTCCCAATTCAATACTAAACAAGTCCGAACTAATTGAATATTTGTATCAGAAATTCTTCGAATTAGTTTA------CCATTTCCATAAAGGATATAATTGACAACGCGGAGTTGCACACTATTCCATTGCTGCAAAAAATCCGGGGGGAAAAGCGCTGCTAAATTTATACCGCCCCTTATTTCATATGTTACGACAGGACGAACCAAAGCAAAAGACTTTTTCTT

Sequences amplified by Hemin1 marker:

>335BI_Hemin1

CAATTTCAAACGGACTTTTCAAGTAATTAAATATTATTCAATGGATGAAGGTGGGAAATTTTATAATCCCGACCCATGCAGTAACATTATTTTCAATCCATTCAATTTGAATTGGTATTTTCTCCGTCACAATTATTGTGAAGAGACGTCTACAATAATTAGCCTTGGGCAGTTTATTTGTGAAAATGTGTGTATAGCCAAAAACGGACCACACCTAAAATCGGG

>336BI_Hemin1

-AATTTCAAACGGACTTTTCAAGTAATTAAATATTATTCAATGGATGAAGGTGGGAAATTTTATAATCCCGACCCATGCAGTAACATTATTTTCAATCCATTCAATTTGAATTGGTATTTTCTCCGTCACAATTATTGTGAAGAGACGTCTACAATAATTAGCCTTGGGCAGTTTATTTGTGAAAATGTGTGTATAGCCAAAAACGGACCACACCTAAAATCGGG

>337BI_Hemin1

CAATTTCAAACGGACTTTTCAAGTAATTAAATATTATTCAATGGATGAAGGTGGGAAATTT-ATAATCCCGACCCATGCAGTAACATTATTTTCAATCCATTCAATTTGAATTGGTATTTTCTCCGTCACAATTATTGTGAAGAGACGTCTACAATAATTAGCCTTGGGCAGTTTATTTGTGAAAATGTGTGTATAGCCAAAAACGGACCACACCTAAAATCGGG

>Amasar1_Hemin1

CAATTTCAAACGGACTTTTCAAGTAATTAAATATTATTCAATGGATGAAGGTGGGAAATTTTATAATCCCGACCCATGCAGTAACATTATTTTCAATCCATTCAATTTGAATTGGTATTTTCTCCGTCACAATTATTGTGAAGAGACGTCTACAATAATTAGCCTTGGGCAGTTTATTTGTGAAAATGTGTGTATAGCCAAAAACGGACCACACCTAAAATCGGG

>Amasar2_Hemin1

-AATTTCAAACGGACTTTTCAAGTAATTAAATATTATTCAATGGATGAAGGTGGGAAATTTTATAATCCCGACCCATGCAGTAACATTATTTTCAATCCATTCAATTTGAATTGGTATTTTCTCCGTCACAATTATTGTGAAGAGACGTCTACAATAATTAGCCTTGGGCAGTTTATTTGTGAAAATGTGTGTATAGCCAAAAACGGACCACACCTAAAATCGGG

>Amasar3_Hemin1

CAATTTCAAACGGACTTTTCAAGTAATTAAATATTATTCAATGGATGAAGGTGGGAAATTT-ATAATCCCGACCCATGCAGTAACATTATTTTCAATCCATTCAATTTGAATTGGTATTTTCTCCGTCACAATTATTGTGAAGAGACGTCTACAATAATTAGCCTTGGGCAGTTTATTTGTGAAAATGTGTGTATAGCCAAAAACGGACCACACCTAAAATCGGG

>SR140_Hemin1

CAATTTCAAACGGACTTTTCAAGTAATTAAATATTATTCAATGGATGAAGGTGGGAAATTTTATAATCCCGACCCATGCAGTAACATTATTTTCAATCCATTCAATTTGAATTGGTATTTTCTCCGTCACAATTATTGTGAAGAGACGTCTACAATAATTAGCCTTGGGCAGTTTATTTGTGAAAATGTGTGTATAGCCAAAAACGGACCACACCTAAAATCGGG

>SR281_Hemin1

CAATTTCAAACGGACTTTTCAAGTAATTAAATATTATTCAATGGATGAAGGTGGGAAATTTTATAATCCCGACCCATGCAGTAACATTATTTTCAATCCATTCAATTTGAATTGGTATTTTCTCTGTCACAATTATTGTGAAGAGACGTCTACAATAATTAGCCTTGGGCAGTTTATTTGTGAAAATGTGTGTATAGCCAAAAACGTACCACACCTAAAATCGGG

>Pteaq_51470_Hemin1

CAATTTCAAACGGACTTTTCAAGTAATTAAATATTATTCAATGGATGAAGGTGGGAAATTTTATAATCCCGACCCATGCAGTAACATTATTTTCAATCCATTCAATTTGAATTGGTATTTTCTCCGTCACAATTATTGTGAAGAGACGTCTACAATAATTAGCCTTGGGCAGTTTATTTGTGAAAATGTGTGTATAGCCAAAAACGGACCACACCTAAAATCGGG

>461NW_Hemin1

CAATTTCAAACGGACTTTTCAAGTAATTAAATATTATTCAATGGATGAAGGTGGGAAATTTTATAATCCCGACCCATGCAGTAACATTATTTTCAATCCATTCAATTTGAATTGGTATTTTCTCCGTCACAATTATTGTGAAGAGACGTCTACAATAATTAGCCTTGGGCAGTTTATTTGTGAAAATGTGTGTATAGCCAAAAACGGACCACACCTAAAATCGGG

>BRM391_Hemin1

CAATTTCAAACGGACTTTTCAAGTAATTAAATATTATTCAATGGATGAAGGTGGGAAATTTTATAATCCCGACCCATGCAGTAACATTATTTTCAATCCATTCAATTTGAATTGGTATTTTCTCCGTCACAATTATTGTGAAGAGACGTCTACAATAATTAGCCTTGGGCAGTTTATTTGTGAAAATGTGTGTATAGCCAAAAACGGACCACACCTAAAATCGGG

Sequences amplified by Hemin2 marker:

>335BI_Hemin2

CCGCACCCAATTTTAAAGAGAAGGTTCGAAAACATATTTATTCCGACTCAGACGGGGAAATGCACTGGAGTACCTATGTCGATCATGCACCTGAATTTACATATGGTAATGTGCATCTATTACCAAAAACAAGTCATTTATGGATATTATTAGGAGGGCCGTGCAGATCCAGTCCAGTCTCCCTTTCCGCTTCACAAGG

>336BI_Hemin2

CCGCACCCAATTTTAAAGAGAAGGTTCGAAAACATATTTATTCCGACTCAGACGGGGAAATGCACTGGAGTACCTATGTCGATCATGCACCTGAATTTACATATGGTAATGTGCATCTATTACCAAAAACAAGTCATTTATGGATATTATTAGGAGGGCCGTGCAGATCCAGTCCAGTCTCCCTTTCCGCTTCACAAGG

>337BI_Hemin2

-----CCCAATTTTAAAGAGAAGGTTCGAAAACATATTTATTCCGACTCAGACGGGGAAATGCACTGGAGTACCTATGTCGATCATGCACCTGAATTTACATATGGTAATGTGCATCTATTACCAAAAACAAGTCATTTATGGATATTATTAGGAGGGCCGTGCAGATCCAGTCCAGTCTCCCTTTCCGCTTCACAAGG

>Amasar1_Hemin2

CCGCACCCAATTTTAAAGAGAAGGTTCGAAAACATATTTATTCCGACTCAGACGGGGAAATGCACTGGAGTACCTATGTCGATCATGCACCTGAATTTACATATGGTAATGTGCATCTATTACCAAAAACAAGTCATTTATGGATATTATTAGGAGGGCCGTGCAGATCCAGTCCAGTCTCCCTTTCCGCTTCACAAGG

>Amasar2_Hemin2

CCGCACCCAATTTTAAAGAGAAGGTTCGAAAACATATTTATTCCGACTCAGACGGGGAAATGCACTGGAGTACCTATGTCGATCATGCACCTGAATTTACATATGGTAATGTGCATCTATTACCAAAAACAAGTCATTTATGGATATTATTAGGAGGGCCGTGCAGATCCAGTCCAGTCTCCCTTTCCGCTTCACAAGG

>Amasar3_Hemin2

CCGCACCCAATTTTAAAGAGAAGGTTCGAAAACATATTTATTCCGACTCAGACGGGGAAATGCACTGGAGTACCTATGTCGATCATGCACCTGAATTTACATATGGTAATGTGCATCTATTACCAAAAACAAGTCATTTATGGATATTATTAGGAGGGCCGTGCAGATCCAGTCCAGTCTCCCTTTCCGCTTCACAAGG

>SR140_Hemin2

CCGCACCCAATTTTAAAGAGAAGGTTCGAAAACATATTTATTCCGACTCAGACGGGGAAATGCACTGGAGTACCTATGTCGATCATGCACCTGAATTTACATATGGTAATGTGCATCTATTACCAAAAACAAGTCATTTATGGATATTATTAGGAGGGCCGTGCAGATCCAGTCCAGTCTCCCTTTCCGCTTCACAAGG

>SR281_Hemin2

CCGCACCCAATTTTAAAGAGAAGGTTCGAAAACATATTTATTCCGACTCAGACGGGGAAATGCACTGGAGTACCGATGTCGATCATGCACCTGAATTTACATATGGTAATGTGCATCTATTACCAAAAACAAGTCATTTATGGATATTAAAAGGAGAGCCGTGCAGATCCAGTCCAGTCTCCCTTTCCGCTTCACAAGG

Sequences amplified by Hemin3 marker:

>335BI_Hemin3

TCCGCTCGTTAATACATAGAGTCCCATAAGCATATCTTGAGTTGGTACGGAAATAGGATCCCCAATAGCTGGAGACAAAAGNTTCATATGAGAAAACATAAGTAAACGGGCTTCNGCTTGAGCCTCCAAAGATAAAGGCACATGAACAGCCAT

>336BI_Hemin3

TCCGCTCGTTAATACATAGAGTCCCATAAGCATATCTTGAGTTGGTACGGAAATGGGATCCCCAATAGCTGGAGACAAAAGATTCATATGAGAAAACATAAGTAAACGGGCCTCCGCTTGGGCCTCAAAAGATAAAGGTACATGAACAGCCAT

>337BI_Hemin3

TCCGCTCGTTAATACATAGAGTCCCATAAGCATATCTTGAGTTGGTACGGAAATGGGATCCCCAATAGCTGGAGACAAAAGATTCATATGAGAAAACATAAGTAAACGGGCCTCCGCTTGGGCCTCAAAAGATAAAGGTACATGAACAGCCAT

>Amasar1_Hemin3

TCCGCTCGTTAATACATAGAGTCCCATAAGCATATCTTGAGTTGGTACGGAAATGGGATCCCCAATAGCTGGAGACAAAAGATTCATATGAGAAAACATAAGTAAACGGGCCTCCGCTTGGGCCTCAAAAGATAAAGGTACATGAACAGCCAT

>Amasar2_Hemin3

TCCGCTCGTTAATACATAGAGTCCCATAAGCATATCTTGAGTTGGTACGGAAATGGGATCCCCAATAGCTGGAGACAAAAGATTCATATGAGAAAACATAAGTAAACGGGCCTCCGCTTGGGCCTCAAAAGATAAAGGTACATGAACAGCCAT

>Amasar3_Hemin3

TCCGCTCGTTAATACATAGAGTCCNATAAGCATATCTTGAGTTGGTACGGAAATGGGNTCCCCAATAGCTGGAGACAAAAGATTCATATGAGAAAACATAAGTAAACGGGCCTCTGCTTGAGCCTCNAAAGATAAAGGTACATGAACAGCCAT

>Decha_SR140_Hemin3

TCCGCTCGTTAATACATAGAGTCCCATAAGCATATCTTGAGTTGGTACGGAAATGGGATCCCCAATAGCTGGAGACAAAAGATTCATATGAGAAAACATAAGTAAACGGGCCTCCGCTTGGGCCTCAAAAGATAAAGGTACATGAACAGCCAT

>Hemin_SR281_Hemin3

TCCGCTCGTTAATACATAGAGTCCCATAAGCATATCTTGAGTTGGTACGGAAATGGGATCCCCAATAGCTGGAGACAAAAGATTCATATGAGAAAACATAAGTAAACGGGCCTCCGCTTGGGCCTCAAAAGATAAAGGTACATGAACAGCCAT

>Pteaq_51470_Hemin3

TCCGCTCGTTAATACATAGAGTCCAATAAGCATATCTTGAGTTGGTACGGAAATAGGATCCCCAATAGCTGGAGACAAAAGGTTCATATGAGAAAACATAAGTAAACGTGCTTCTGCTTGAGCCTCCAAAGATAAAGGCACATGAACAGCCAT

>BRM391_Hemin3

TCCGCTCGTTAATACATAGAGTCCAATAAGCATATCTTGAGTTGGTACGGAAATAGGATCCCCAATAGCTGGAGACAAAAGGTTCATATGAGAAAACATAAGTAAACGTGCTTCTGCTTGAGCCTCCAAAGATAAAGGCACATGAACAGCCAT

>461NW_Hemin3

TCCGCTCGTTAATACATAGAGTCCAATAAGCATATCTTGAGTTGGTACGGAAATAGGATCCCCAATAGCTGGAGACAAAAGGTTCATATGAGAAAACATAAGTAAACGTGCTTCTGCTTGAGCCTCCAAAGATAAAGGCACATGAACAGCCAT

>75NAT_Hemin3

TCCGCTCGTTAATACATAGAGTCCAATAAGCATATCTTGAGTTGGTACGGAAATGGGATCTCCAATAGCTGGAGACAAGAGATTCATATGAGAAAACATAAGTAAACGAGCCTCCGCTTGAGCTTCCAAAGATAAAGGTACATGAACAGCCAT

>BRM278_Hemin3

TCCGCTCGTTAATACATAGAGTCCAATAAGCATATCTTGAGTTGGTACGGAAATGGGATCTCCAATAGCTGGAGACAAGAGATTCATATGAGAAAACATAAGTAAACGAGCCTCCGCTTGAGCTTCCAAAGATAAAGGTACATGAACAGCCAT

>867NW_Hemin3

TCCGCTCGTTAATACATAGAGTCCAATAAGCATATCTTGAGTTGGTACGGAAATGGGATCTCCAATAGCTGGAGACAAGAGATTCATATGAGAAAACATAAGTAAACGAGCCTCCGCTTGAGCTTCCAAAGATAAAGGTACATGAACAGCCAT

Sequences amplified by Hemin4 marker:

>SR140_Hemin4

TTTCTTNNGCCTGAGTTGGNNTNNA-GCACGGAATGNATTTGCACCGTCGCCGTCTTCAAATAAAGTATTTTCTACGGTAGCACCATGAATAGCNNA---

>SR281_Hemin4

TTTCTTNNGCCTGAGTTGGGTTAAAANCACGGAANNNATTTGCACCGTCGCCGTCTTCAAATAAAGTATTTTCTACGGTAGCACCATGAATAGC------

>Pteaq_51470_Hemin4

TNNCTTCNGCTTGAGTTGGGTTANA-GCACGGAATGNATTTGCACCNTCACCATCTTCAAATAAAGTATTTTCTACAGTAGCACCATGAATAGCGCANAN

>461NW_Hemin4

TNNCTTCNGCTTGAGTTGGGTTANA-GCACGGAATGNATTTGCACCATCNCCATCTTCAAATAAAGTATTTTCTACNGTAGCACCATGAATAGCGCA-AN

>BRM391_Hemin4

TNTCTTCNGCNTGAGTTGGGTTANA-GCACGGAATGNATTTGCACCATCACCATCTTCAAATAAAGTATTTTCTACAGTAGCACCATGAATAGCGCANAN

>75NAT_Hemin4

TCTCTTCGNNNTGGGTTGGGTTAAAAGCACGGAATGNATTTGCACCNTCACCATCTTCGAATAAAGTATTTTCTACGGTAGCACCATGAATAGCGC----

>867NW_Hemin4

TNTCTTCGNNTTGGGTTGGGTTAAAAGCACGGAATGNATTTGCACCATCACCNTCTTCGAATAAAGTATTTTCTACGGTAGCACCATGAATAGCGC----

>BRM278_Hemin4

TCTCTTCGNNTTGGGTTGGGTTAAAAGCACGGAATGNATTTGCACCATCACCATCTTCNAATAAAGTATTTTCTACGGTAGCACCATGAATAGCGC----

*Decalepis hamiltonii* chroloplast genome contigs:

>Decalepis_hamiltonii_chloroplast_contig1

CCCCGATGCGAATCTCTATAGTTGGTGCTAGCAGAAGCCCTTTGTTTGTCTACGAGCTATTTGAATTGGAAAAAAGTCAGCAGAGCAGTCTCCACCAAGGTGAAGTTGAGTATAGGGATGGATTCTGTATGGACGGGATAAGGGGCTGATTGCGCGACCTTCGATTTGAAGCAACTTCCGCGCGACTGCACCTGGTCTTGGATCAAGGCTCTCAGTCGCTCAAGACGTCGATGCGCCACCGAGTCGACCCGAAACTTACCAGCATGGGGAGTTGCGATGGATGCCAAGATGAAGACTTTCCGCCTCCAAGCTTGATTGAAGGACTACTTCCCCATCACTATCAGAGTTGGAAACGAAATGGAATCCCGGATAAGAGTAAGAGCAAGCTTATAACTAGCCTTTCGGGCTATGTCCTTCACTTTAAGTATTTACAGGATTCTACTGAGTATGGGTAGGAGTCAGGGGTTCGAGCCCTTCTCCAACAACTGTTGTAATTATTGGCAAGAGCCAGTGATCGCCAATTCCTTTCCCAAAGGCTCCTCGGACTGGACTAGAGTACGTTCTTTTCGCTTTCCGTCGATCGAAGTAGGTCAAGTCTCAGTCTTGTATTTGAGGTTGGTCGAGTAAAGCACGAGTTCCGGTAAACCCCTACCAGTAAGGGGCAAGCCAGCATCTTGACCGGATTGGAAAGCAGAGGTGGGGGTTGTCAAGTTTGTTTTGGTTTGAATTTGAAGTAGGGTAACTTTGCAGAATCTTTGCTTGCGCCCTATTTGAGACTAAGGCAGGTTTGGAACCCTGTCCTATCACCTTTATCAAATCCCTAGCTCTCTTCCTTAGTGCAACTGTCCTATTCCTACCATGGGAATATCTATCTTTCTTTTTGTGTCATATCTTTAGTTTCGGATATCAAACCGGACGGTATCGTATATGAAGAAAGAGATTGTTGACATTAGTAGACTAATCTATTCATTGGTAGGGCATTTCTTCCTGTGACCGCCTTTCCTACCAAAAACTAACCCAACCAAATCCGGGTTTTAGACCTTCGGGATTTTTTAGGGTTCATACATGCATTGATCCAGTTCTAGAGAACTGCCTGCTCCAGAGCCGGGAGCACCTGAAAGATATCGATTTTTTTCATACAATTTTTGAGATCGAGACTCGAGAAGCGAGTCACAGAGACAAAAAGAGAGGGGGGGCATAGCTATCAGAGGCAACGGGGACAGAAGCAAGGATGGCAGTACCAATTTCAAAGAATTTGGAATATCCCCCATTTCGGGGATGCATAGCCGCCACTATATCATCCAATTCCAGTGCCTGTGAGAGACCCGGTTTACCGAGTAGTTGATTTGATTGATCCAGTTCAATAGCCGCTTCCGCTGAAGCGACAGAACCAACAGCATAGTCAACAGAAGCAGCAGGATCAATGGCTTAAAGTACTGTTTCTTCAACGCTTCTCCTGCGACTGCCTTAGCAGCAGGGTTGAAGGGAAGAAGACAGCAGGTGTGTGGTATAGCTCTTATCTGTTCGCTCCTGTCGACTCAGAACTCTATTTACGCTTAGCCGGGAACAAGATCTTTGTTTCAGCAGAACAGATTGTTGCTAGTGGGACTATAGTTTTCTGTTTGGCGACTCGGAAGTAAGGTAGCTTTAGTGAAGTCTGGCAGAAGTACTAAAGTATGTAGCAAATGTCGGCATATCCGCATCCGGCTCAGGGAAAGGCGCAGGGGTGACTGTATTGCTATTAGCAGATGAAGGTGCGGAGAAAGCAGCAGCTAAGATCTCAGGTCCCACCCTCCATCAGACCGGGCGACAGGCGAGAAATGCTTTCCGTCACTAGTCTTACTAAAAATAATAAAGCATAGGGCTAAGAATTTCTTGTTGAAAGCATCCATCCTGCTCTGAGAACAAGCTTGGCAAGGTCTTCAGTGACTGAGTTGGATGAGAACTAATTTGAGAAAACTGGCAACTTGTGCGATTACGAACAGGTATTCCTTCTAAAGCGACTCACGAATATACTCCACGTCGATTTCTTCCCTAAGCTTAATAACGAGAGGAAGTCAAAAGATTTTGGAAATGAATCCTAACTATTTGAATTCTCCTTTATGAAAGGGGATGTGTGTAAAAGAAACAGTATATTGATAAATTCAGTTTTTCCGAAATCAAAAGAGCGATTAGGTTGAAAAAGAAAAGATTTTTCACCACCTTCTTTCCTTATCCTATAATCTTATCCAAAAATGGAAAAAGAAGAAATAGAGAATCTGTTGATAAGTTTACCTATTTCCGAGGTATCTATTCTTACTAGAATACCCTGTTTCGACTCTATCGCACTATGTATCATTTGCTAACCCCAAAATCGTCTATCTTGGATTCAAATCGAATTTCAAATGGAAGAAATCCAAAGATATTTACAGCTTGATAGATCTCAAGAACCCAGCTTTTTATATCCACTTATCTTTCAGGAGTATATTTATGCACTTGCTCATGACCATAGTTTAAACCGAGCTATTTTGTTGGAAAATCCAGGTTATGACAATAAATCTAGTTTCCTTATTGTGAAACGGTTAATTACTCGAATGTATCAACAGAATCATTTTCTTACTTCTGCTAATGATTCTAGCCAAAATCCATTTTTGGGGCGCAACAAGAATTTGTATTCTAAAATGATATCAGAGGGATTTTCATTTATTGTGGAAATTCCGTTTTCTATGCGATTAATATCTTCTGAAGAACGCAAAGGGATATTCAAATCTTATAATTTACGATCAATTCATTCAATATTTCCTTTCTTAGAGGACAATTTTTCACATTTTTATTTTATGTTAGATATACAAATACCCCATCCTGTTCATCTGGAAATCTTGGTTCAAACCCTTCGCTATTGCGTAAAAGATGCCCCTTCTTTGCACTTATTACGATTCTTTCTACGCGAGTATTGTAATTGCAATAAGATTATTGCTACAAAGAAAACCAGGTTTCATTTTTTAACAAAAGAAATCCAAAAGAAATCAAAGATTATTCTTCTTGTTATATAATTTTTATGTATGTGAATACGAATCCATTTTCGTCTTTCTCCATAACCAATCTTCTCATTTACGACCAACATCCTTTGGGGTCCTTCTTGAACGAATCCATTTCTATGGAAAAATAGAAGATCTTGCCGAAGTACTCACTAAGGATTTTCCGACCAACTTATGCTTGTTCAAATATCCTTTCGTGCATTATGTTAGGTATCAAGGAAGATGCATTCTGCTTTCAAGGGGACGGCTCTTTTGATGAAAAATGGAAATATTACCTTGTCAATTTTTGGCAATGTAATTTTGACCTGTGGTTTCACTCGCGAAGGGCCTATATAAAGCAATTGTACAAAAATTCCCTTGACTTTATGGGTTATCTTTCAATTGTGCGACTAAACCCTTCAACCGTACGGAGTCAAATGCTCGAAAATGCATTTCTAATTAATAATGCTATTAAGAAATTAGATACCCTTGTTCCAATTATTCCAATGATTGGATCATTGGCTAAAGCGAAATTTTGTAACCTATTAGGACACCCCATTAGTAAGCCGGTTCGGACTGATTTATCAGATTCTGATATTATGGACAGATTTGGGCGTATATGCAGAAACCTTTCCCATTATTATAGCGGATCTTCAAAAAAAGAGTTTGTATCGAATAAAGTATATACTTCGACTTTCTTGTGCCAAAACTTTAGCTCGAAAACACAAAAGTACTGTACGGGCTTTTTGAAAAGATTAGGTTCGGAATTTTTGAAGAATTCTTCATGTCGGAAGAAGAAGTGCTTTCGTTGACCTTCCCAAGAGTTTATTCTCTTTTTGGGGGTCTATAGAAGTCGGATTTGGTATTTTGATATTACTTGTATCAACGATCTGGCGAATCAGCAATGATTCCTTCTGAGACTATTTAAATGGAAATTTTCTTAAATGAAAATGATAACAAAGAAAATGATAACTTATTTCTAAAATTCTGAAATGTTGATGTAGCACGTAAGTAGCTTGTAATTCGAATAGGGGTTACATTACCTGACTATTCTTTAAAGTTTTTCTAAGAAAGCAACTGATGTATACATAGGGAAAGCCGTGTGCAACGAAAACTGCAAGCACGGCTTGGGGAGGGGTGTTTACTTAGTTTAACAAGGAAATTATCTACTCCATCCGACTAGTTCCGGGTTCGAATCCCGGGCAACCCACTATCATATCAAAATTGTATGTATAGAAATCCGTATTTGATTTTTTCAATCCATTTCATTTTCAAAAATTCGAATCTAGTTAGATCGATATTGGTTGACACTAGCATATAAGTCATGTTATACTGTTGAATAACAAGTCCTCCATTTTATCTAAATAGAAATAGAGAATGTGTGCGCTTGGGAGTCCCTGATGATTAAATAAATCAAGATTTTACCATGACTGCAATTTTAGAGAGACGCGAAAGCGAAAGCCTGTGGGGTCGCTTCTGTAACTGGATAACTAGCACTGAAAACCGTCTTTACATTGGATGGTTTGGTGTTTTGATGATCCCGACCTTATTGACAACTTCCGTATTTATTATCGCCTTCATTGTTGCCCCTCCAGTAGATATTGATGGTATTCGTGAACCTGTTTCTGGATCTTTACTTTACGGAAACAATATTATTTCTGGTGCCATTATTCCTACTTCTGCAGCTATAGGTTTGCACTTTTACCCAATCTGGGAAGCAGCATCCGTTGATGAATGGTTATATAACGGTGGTCCTTATGAACTAATTGTTCTACACTTCTTACTTGGTGTAGCTTGTTACATGGGTCGTGAGTGGGAGCTTAGTTTCCGTCTGGGTATGCGACCTTGGATTGCTGTTGCATATTCAGCTCCTGTTGCAGCTGCTACCGCTGTTTTCTTGATCTACCCAATCGGTCAAGGAAGTTTTTCTGATGGTATGCCTCTAGGAATCTCTGGTACTTTCAACTTCATGATTGTATTCCAGGCTGAGCACAACATCCTTATGCACCCATTCCACATGTTAGGCGTAGCTGGGGTATTCGGCGGCTCCTTATTCAGTGCTATGCATGGTTCCTTGGTAACTTCTAGTTTGATCAGGAAACCACAGAAAATGAATCTGCTAATGAAGGTTACAGATTCGGTCAAGAGGAAGAAACTTATAATATCGTAGCTGCTCATGGTTATTTTGGCCGATTGATCTTCCAATATGCTAGTTTCAACAACTCTCGTTCGTTACACTTCTTCCTAGCTGCTTGGCCTGTAGTAGGTATCTGGTTCACTTCTTTAGGTATCAGCACTATGGCTTTCAACCTAAATGGTTTCAATTTCAACCAATCTGTTGTTGATAGTCAAGGCCGTGTAATTAATACTTGGGCTGATATCATTAACCGTGCTAACCTTGGTATGGAAGTTATGCATGAACGTAATGCTCATAATTTCCCTCTAGACCTAGCTGCTATAGAAGCTCCATCTATCAATGGCTAAGACCCCGGTCTTAGTGTTAGTGTATAGGAGTTATTGAAAATAAAGGAGCAATACCGCCCTCTTGATAAAACAAGAAGGTGATTATTGCTCCTTTATTTTCTTTTTTTGGTACATTATCAAAGATTCAGATATCTTAGAAATAGAAATAATAAAGAAAATGGTCGAAACGAAATTCTAAATAAAATAGAATTGAAAATGAAGAAAATGAAATAGTAAATATTAAATTAGAATAGGAAATAAATAAATACAAATAGACTGAAGGAGTGAAGGGGCGGATGTAGCCAAGTGGATCAAGGCAGTGGATTGTGAATCCACCATGCGCGGGTTCAATTCCGTCGTTCGCCCATTTGTGTTAAGTTTCTCAATTTTTTTAATAAATGGTTGGCTACAAAAGGATTTTTTAGTGAACGTGTCACAGCTTACTCCTATTTTTTTTCTTTTGTTTTGTAAAGACGAAGAAAAAATTCGATTTTCTCTCTATTTACTACGGCGACGAAGAATCAAATTATCACTATATTTATTCCTTTTCTACTTCTTCTTCCAAGTGCAGGATAACCCCAAGGGGTTGTGGGTTTTTTCTACCAATTGGGGCTCTCCTTCACCACCCCCATGGGGATGGTCTACAGGGTTCATAACTACTCCTCTTACTACAGGACGCTTACCTAGCCAACGCTTAGATCCGGCTCTACCCAAACTTTTCTGGTTCACCCCAACATTCCCCACTTGTCCGACTGTTGCTGAGCAGTTTTGGATATCAAACGGACCTCCCCAGAAGGTAATTTTAATGTGGCCGATTTCCCTCTTTTGCAATCAGTTTCGCTACAGCACCCGCTGCTCTAGCTAATTGTCCACCCTTTCCAAGTGTGATTTCTATGTTATGTATGGCCGTGCCTAAGGGCATATCGGTTGAAGTAGATTCTTCTTTTGATCAATCAAAACCCCTTCCCAAACTGTACAAGCTTCTTACAAAGCATACGGCTTTCTGGATGTAGATGATGATATCTATACAGATGGATCTTATATCGTACAATGACCCATGGGTGGATATATGAATCCAACCGAATGACTCATGTTATGATCTTCTACATCCTAGGTCTTCCTGTTCCGTCATCTGGCTTATGTTCTTCATGTAGCATTCAGACCGAATGACTCTATGAAATTACGTCGATACTTCCACATATTATGGGTAACGTAGGAGACATCTCTATTTTCCCCCGGGAATCTTTAGAATTACCACTGCTTAGCTTTCAATTCGCCTCTGACCATCAAATGAAATGTGAATCACCCGTCCTCCTCTCTTTGAAAGAAGGGGCGCTTCCGGTTCTGTCGGTGCTTGAAACAATTTTGTCTTCTCCATATTACTATATCTCTAGAGTCAATAATTTTATATGAGGAACTACTGAACTCACTCACTTGCTGCCGTTACTCTTCAGTTTTCTGTTGAGGTCTATCCTGTAGAGGTACTCAAATTGGATCAGTGATCGATTTCTAGGTTTCGTCGTAAACCTAATTGGTTACTTCCAATTACGTAAATCAATAGTTCAAACCGCACTCAAAGGTAGGGCATTTCCCATTTTATAGGAACTTCTGTACCAGAAACAATGGTATCTCCAAGTATAGCCCCTCTGGGATGTAAAATATATCTCTTCTCGCCATCCCCATAGTGTATGAGACAAATGTATGCATTTCGATTAGGGTCGTATTCTATGGTTACGATTCTACCATATATGTCTTTTTCATTCCGTCGAAAATCGATTTTACGGTATAGACGCTTATGACCTCCCCTCTATGCCCTGCGGTAATGATTCCTCTGGCATTACGACCTTTACCACAACGATGTTGTCCATAGATCAAATTATTTCGTGGATTGGATTTCACTTGACTGTCTACGGTTCCATTGCGTGTGCTCGGGGTAGAAGTTTTGTATAAATGTATCGCCATGCTATTAAGTATTTTGATTTAAGTTCTTTTCTTTCTAAGAGGTGGAATAGAATAACCCGGTTGAAGCGTAATGATCATACGTCTGTAATGCATTGTATGTCCCATAATAGGTCCCATTCTTCTACCCTTTCCCGGAAGTCGATGACTATTCATAGCTATTACCTTGACACCAAAGAAGAGTTCGACCCAATGCTTTATTTCTGTCCTAGTTGATCCCGATTCGACATTAGAAGTATATTGATTTTTCCCCAATAACCGAATACTTTTGTCTGTAAATACTGCATATTTGATTCCATCCATAAATCGACTTTCTTCCCTATGAGTTCTAGTCTCAATAAGAATGCTAGTTCTTACTGTTCATATATTATGATATGAATATACCACACCAATTCGTTATGTATGGATGATGAGATTCCATTGATACAGAGCCAATTCCAATAGACTTATTGGAGGGTCCCATTGGCGTGCATCCAGTAGGAATTGAACCTACGAATTCGCCAATTATGAGTTGGGCGCTTTAACCATTCAGCCATGGATGCTTAGCGGGGATCCTCGTACATAGTGAATAACCAAATTCCAATTGAAATGAAATCTTTGGGATAAATCAATGCCATTTAGGAGTAATCAATGAAAGGAAATGAATTCAAATCCTGGATTTTCGAATTGAGAGAGATATTGAGAGAGATCAAGAATTCTCACTATTTCTTAGATTCATGGACCCAATTCAATTCAGTGGGATCTTTCATTCACATTTTTTCCACCAAGAACGTTTTATAAAACTCTTTGACCCCGAATTTGGAGTATCCTACTTTCACGTAATTCACAGGGTTCAACAAGCAATCGATATTTCACGATCAAGGGTGTAATACTCTTTGTAGTAGCGGTCCTTATATATCGTATTAACAATCGAAATATGGTCGAAAGAAAAATCTCTATTTGATAGGACTTCTTCCTATACCTATGAATTCCATTGGACCCAGAAATGATACATTGGAAGAATCCGTGGGGTCTTCCAATATCAATAGGTTGATTGTTTCGCTCCTGTATCTTCCAAAAGGAAAAAGATCTCTGAGAGTTGTTTCCTGAATCCGAAAGAGAGTACTTGGGTTCTACCAATAACTAAAAAGTGTAGCATCCCTGAATCTAACTGGGGTTCGCGGTGGTGGAGGAACTGGATCGGAAAAAGAGGGATTCTAGTTGTAAGATATCTAATGAAACCGTCGCTGGAATTGAGATCTTATTCAAAGAGAAAGATCTCAAATATCTGAAGTTTCTTTTGTATATTATATGGATGATCCGACCCGCAAGGACCACGATTGGGAATTGTTTGATCGTCTTTCTCTGAGGAAGAGGCGAAATCCAATCAACTTGAATTCAGGACCGCTATTCGAAATCTTAGTGAAACACTGGATTTCTTATCTCATGTCTGCTTTTCGTGAAAAAATACCAAGTGAAGTGGAGGGTTTCTTCAAACAACAAGGGGCTGGGTCAACTACTCAATCAAATGATATTGAGCATGTTTCCCATCCCTTCTCGAGAAACAAGTGGGCTATTTCTTTGCAAAATAGTGCTCAATTTCATATGTGGCAATTCCGCCAAGATCTCTTCATTAGTTGGGGAAGAATCTGCACGAATCGGATTTTTGAGGAACGTATCGAGAGAGAATTGGATTTGGTTAGACAATGTGTGGTTGGTAAACAGGGATCGGTTTTTAGCAAGGTACGGAATGTATCGTCAAATATTCAATATGATTCCACAAGATCTAGTTTCGTTCAAGTAACGGATTTTAGCCAACTGAAAGAATCTTCTGATCAATCCAGGGATCATTTGGATTCCATTAGTAATGAGGATTCGGAATATCACACATTGGTCAATCAAAGAGAGATTCAACAACTAAAAGAAAGATCGATTCTTTGGGATCCTTCCTTTCTTCAAACGGAACGAACAGAGATAGAATCAGACCGATTCCCGAAATGCCTTTCTGGCTATTCCTCAATGTCCCGGCTATTCACGGAACGTGAGAAGCAGATGATTAATCATCTGCTTCCGGAAGAAATCGAAGAATTTCTTGGGAATCCTACAAGATCCGTTCGTTCTTTTTCTCTGATAGATGGTCAGAACTTCATCTGGGTTCGAATCCTACTGAGAGGTCCACTAGAGATCCAAAATTGTTGAAGAAAGAACAAGATCTTTCTTTTGTCCCTTCCAGGCGATCGGAAAATAAAGAAATGGTTAATATATTCAAGATAATTACGTATTTACAAAATACCGTCTCAATTCATCCTATTTCATCAGATTCGGGATGTGATATGGTTCCGAAGGATGAACCGGATATGAACAGTTCCAATAAGATTTCATTCTTGAACAAAAATCCATTTTTGATTTATTTCATCTATTCCATGACCGGAACAGGGAGGATACAGGTTACACCACGATTTTGAATCAGAAGAGAGATTTCAAGAAATGGCAGATCTATTCACTCTATCAATAACCGAGCCGGATCTGGTGTATCATAAGGGATTTGCCTTTTCTATTGATTCCTACGAATTGGATCAAAACAATTCTTGAATGAGGTATTCAACTCCAGGGATGAATCGAAAAGAAATCTTTATTGGTTCTACCTCCTATTTTTATGAAGAGAATGAATCTTTTCTCGAAGGATCAGAAAAAACGGGTCCGGATCTCCTGCGGAATGATTTGGAAGATCCACAACCAAAAGAGTGGTATTTGCTAGCAACAACATAATGGAGGCAGTCAATCAATACAGATTGATCCGAAATCTGATTCAAATCCCATATAGTACCTATGGGTACATAAGAAATGTATTGAATCGATTCTTTTAATGAATAGATCCGATCGCAACTTCGAATATGGAATTCAAAGGGATCAAATAGGAAAGGATACTCTGAATCATAGAACTATAATGAAATATAGGATCAACCAACATTTATCGAATTTGAAAAGAGTCATAAGAAATGGTTCGATCCCTTATTTTGATTTCTCGAACCGAGAGATCCATGAATCGGAATCCTGATGCATATAGATACAAATGGTCCAATGGGAGCAAGAATTTCCAGGAACATTTGGAACATTTCGTTTCTGAGCAGAAGAGCCGCTTTCAAGTAATGTTTGATCGATTACGTATTAATCAATATTCGATTGATTGGTCTGAGGTTATCGACAAAAAGATTTGTCTAAGCCACTTCGTTTCTTTTTGTCCAAGTCACTTCTTTTTTGTCCAAGTTGCTTTTCTTTTGTCTAACTCACTTCCTTTTTCTGTGTGAGTTTCGGGAATATCCCCATTCATAGGTCCGAGATGATCTACATCTATGAATTGAAAGGTCCGAATGATCAACTCTGCAATCAGTTGTTAGAATCAATAGGTCTTCAAATTGTTCATTTGAAAAAACGGAAACCCTTCTTATTGGATGATCATGATACTTCCCAAAAATCGAAATTCTTGATCAATGGAGGAACACTATCACCATTTTTGTTCAATAAGATACCAAAGTGGATGATTGACTCATTCCATACTAGAAAGAATCGCAGGAAATCCTTTGATAACACGGATTCCTATTTATCAATGATATTCCACGATCAAGACAATTGGCTGAATCCCGTGAAACCATTTCATAGAAGTTCATTGATATCTTCTTTTATAAAGCAAATCGACTTCGATTCTTGAATAATCCACATCACTTCTGCTTCTATTGGAACACAAGATTCCCTTTTCTGTGGAAAAGGCCCGTATCAATAATTATGATTTTACGTATGGACAATTCCTCAATATCTTGTTCATTCGCAACAAAATATTTTCTTTGTGCGTCGGTAAAAAAACATGCTTTTGGGGAGAGATACTATTTCACCAATCGAGTCACAGGTATCTAACATATTCATACCTAACGATTTTCCACAAAGTGGTGACGAAAGGTATAACTTGTACAAATCTTTCCATTTTCCAAGTCGATCCGATCCATTCGTTCGTTACTCGATCGCAGACATTTCTGGAACACCTCTAACAGAGGGACAAATAGTCAATTCTGAAAGAACTTATTGTCAACCTCTTTCAGATATGAATCTATCTGATTCAGAAGGGAAGAACTTGCATCAGTATCTCAATTTCAATTCAAACATGGGTTTGATTCACACTCCATGTTCTGAGAAATATTTACCATCTGAAAAGAGGAAAAACGGAGTCTTTGTCTAAAGAAATGCGTCGAGAAAGGGCAAATGTATAGAACCTTTCAACGAGATAGTGCTTTTCAACTCTCTCAAAATGGAATCTATTCCAAACATATATGCCATGGTTCCTTACTTCGACAGGGTACAAATATCTAAATTTGATATTTTTAGATACTTTTTCAGACCTATTGCCGATACTAAGTAGCAGTCAAAAATTTGTATCCATTTTTCACGATATTATGCATGGATCGGGCATATCATGGCGAATTCTTCAGAAAAATTGGTGTCTTCCACAATGGAATCTGATAAGTGAGATTTCGAGTAAGTGTTTACATAATCTTCTTCTGTCCGAAGAAATGATTCATCGAAATAATGAGTCACCATTGATATCGGCACATCTGAGATCGCCAAATGTTCGGGAGTTCCTCTATTCAATCCTTTTCCTTCTTCTTGTTGCTGGATATCTCGTTCGTACACATCTTCTCTTTGTTTCCCGGGCCTCTAGTGAGTTACAGACAGAGTTCGAAAAGTCAAATCTTTGATGATTCCATCATCTATGATTGAGTTGAGAAAACTTCTGGATAGGTATCCTACATCTGAACCGAATTCTTTCTGGTTAAAGAATCTCTTTCTAGTTGCTCTGGAACAATTAGGAGATTCTCTAGAAGAAATACGGGGTTCTGCTTCTGGCGGCAACATGCTTGGTCCCGCTTATGGGGTCAAATCCATACGCTCTAAGAAGAAATATTTGAATATCAATCTCATCGATATTGTCGATCTCATACCAAATCCCATCAATCGAATCACTTTTTCGAGAAATACGAGACATCTAAGTCATACAAGTAAAGAGATTTATTCATTGATAAGAAAAGAAAAACGTGAACGGGGGTTGGATTGATGATAAAATAGAATCCTGGGTCGCGAACAGTGATTCGATTGGTGATGAAGAAAGAGAATTCTTGGTTCAGTTCTCAGCCTTAACGACAGAAAAGGGATTGATCAAATTCTATGGAGTCTGACTCATAGTGATCATTTATCAAAGAATGACTCTGGTTATCAAATGATTGAACAACCGGGAGCAATTTACTTACGATACTTAGTTGACATTCATAAAAAGTATCTAATGAATTATGAGTTCAATACATCCTGTTTAGCAGAAAGACGGATATTCCTTGCTCATTATCAGACAATCACTTACTCACAAACTTCGTGTGGGACTAATAGTTTTCATTTCCCATCTCATGGAAAACCCTTTTCGCTCCGCTTAGCCCTATCCCCTCTAGGGGTATTTTAGTGATAGGTTCTATAGGAAGTGGACGATCCTATTTGGTCAAATACCTAGCGACAAACTCCTATGTTCCTTTCATTACGGTATTTCTGAACAAGTTCGATTTTCTTATTGATGATAGTGACGATATTGATGATAGTGACGATATTGATGCTAGTGACGATATTGATGCTAGTGACGATATTGATGCTAGTGACGATATTGATCGTGACCTTGATACGGAGCTGGAGCTGCTAACTATGATGAATGCGCTAACTATGGATATGATGCCGGAAATAGACCAAATTTATATCACCCTTCAATTCGAATTAGCAAAAGCAATGTCTCCTTGCATAATATGGATTCCAAACATTCATGATCTGGATGTGAATGAGTCGAATTACTTATCCTCGGTCTATTAGCGAACCATCTCTCCAGGGATTGTGAAAGTAGAAATATTCTTGTTATTGCTTCGACTCATATTCCCCAAAAGTGGATCCTGCTCTAATAGCTCCGAATAAATTCAATACGTGCATTAAGATACGAAGGCTTCTTATTCCACAACAACGAAAGCACTTTTCACTCTTTCATATACTAGGGATTTCACTTGGAAAACAAAATGTTCCATACTAATGGATTCGGGTCCATAACCATGGGTTCCAATGCACGAGATCTTGTAGCACTTACCAATGAGGCCCTATCGAGTAGTATTACACAGAAGAAATCAATTCTAGACACTAATACAATTAGATCCGCCCTTCATAGACAAACTTGGGATTTGCGATCCCAGGTAAGATCAGTTCAGGATCATGGGATCCTTTTCTATCAGATAGGAAGGACTTTAGCACAAAATGTACTTCTAAGTAATTGCCCCATAGATCCTATATCTATCTATATGAAGAAGAAATCGTGTAACGAAGGGGATTCTTATTTGTACAAATGGTACTTGGAACTTGGAACGAGCATGAAGAAATTAACGATACTTCTTTATCTTTTGCGTTGTTCTGCCGGATCGGTCGCTCAAGACCTTTGGTCTCTACCCGGACCCGATGAAAAAATGGGATCGCTTCTTATGGACTCGTTGAGAATGATTCGGATCTAGTTCATGGCCTATTAGAAGTAGAGGGCGCTCTGGTGGGATCTTCGCGGACAGAAAAAGATTGCAGTCAGTTTGATAATGATCGAGTGACATTGCTTCTTCGGCCCGAACCGAGGAATCCCTTAGATATGATGCAAAGCGGATCTTGTTCTATCCTTGATCAGAGATTTCTCTATGAAAAATACGAATCGGAGTTTGAAGGGGGAGGAGAAGGAGCCCTTGACCCGCAACAGATAGAGGAGGATTTATTCAATCACATAGTTTGGGCTCCTAGAATATGGTGCCCTTGGGCCTTTCTATTTGATTGTATCGAAAGGCCCAATGAATTGGGATTTCCCTATTGGGCCGGGTCATTTCAGGGCAAGCGGATCATTTATGATGAAGAGGATGAGCTTCAAGAGAATGATTCGGAGTTCTTGCAGAATGGAAGGGTGCAGTACCAGACACGAGATAGATCTTCCAAAGAACAAGGCTTTTTCCGAATAAGCCAATTCATTTGGGACCCTGCAGATCCACTCTTTTCCTATTCAAAGATCAGCCCCTGGCTCTGTGTTTTCACATCGAGAATTATTTGCAGATGAAGAGATGTCAAAGGGCTTCTTACTTTCCAAACAGATCCTCCTACATCTATATATAAACGCTGGTTTATCAAGAATACGCAAGAAAAGCACTTCGAATTGTTGATTAATCGTCAGAGATGGCTTAGAACCAATAGTTCATTATCTAATGGATCTTTCCGTTCGAATACTCTATCCGAGAGTTATCAGTATTTATCAAATCTGTTCCTATCTAACGGAACGCTATTGGATCAAATGACAAAGACATTGTTGAGAAAAGATGGCTTTTCCCGGATGAAATGAAAATTGGATTCATGTAACGGGAGAAAGATTTCCCATTCCTTAGCCGGAAAGATATGTGGCCATGAAAGAGGGATTAAGTGGATGGGTGCTAGAGTCGTGGAAACGCTTGTTTCTTCCATATTTTGGACCTTAGCTCCATGGAACAATATGTTACTGCTGAAACACGGAAGAATTGAAATCGTAGATCAAAACACTATGTATGGATGGTATGAACTGCCTAAACAAGAATTCTTGAACAGCGAACAACCAGTTCAGATATTCACGACCAAGAAGTACTGGATTCTCTTCTCTTTCGGATAGGCCCTGAAAAGAGAAGGAAGGCTGGAATGCCAACAGGCGTCTATTATTGAATTCACCCGACCCGATAGTACCCATTTTGGGAACGTCCAGGTCCAGTGCCAAAGTCACTGAATGGGTAAGTCGCCAATCCTTGGACTATGTAATGTACTTTATCTGCTGGGTTACGGGCGGGCATTTTACCAGAGGTTTCTAATCTATGATTCCTGTTGAAACATATACTCGGGGGTGGGTGCAGGGCGGACGATTTCAAATTGAAATGAAAAGCAGACTCCCCATTCATTAGATAGAGAAGATCGCCAAGATTTCGTAAGATTTCGTGATCTGCTGCCGAACTTATTCAAGCTCGGATCGAATCGGTATTGATATACCGATTCGATCCGAGCTCGCTTATTGAATTGCTCATTCAATGAGCATTCTCCATATTATGCCTTGAAGAGGACTCGAACCTCCACGCTCTTTAGCACGAGATTTTGAGTCTCGCGTGTCTACCATTTCACCACCAAGGCATCTTGAAAGTGAATCGTATTCCATGAATATGATATCTATCTAGTGTGATGTATGGAATATATGACAAAGGTGGAGTATTTCTATTGATCGGTCATGTCATATAGGCCCGAGTTGGACATCTAATTGCTTCGATTTGAATTATCCGGAGAATGCCTTAGATATATCAAAAGATGGACAATCAAACCTATTTCTCGATTCAATAGAAGCTCAAAGAGGTGAATAGGGTCCCAAATAACGAGAGATGTGTAAAAGCAGGTCTGATTACGCCTATTCCTAATCCTAAATGGAATGACGTAGGGATCCATTTGTATGTAAATAGAGTATCTATTTAAATACGCTCGAATGACCCCTTCTCATAATGAGAATGTATAGAACCCTATTCCGGTCTGGTCCGGTATGGAATGAACTTATAATCATGGAATCGACTCGATCATCAGATTATAAGTTCATAACCCTAGCCCATTCCCATTTTGGGCGGAACAGGTCTACTAATTCTTTGATTCCAGTTAGTAAGAGGGATCTTGAACTAAGAAATAGACCCTAGAAGCTAAAAAGTGTATCCTGAGCAATTGCAATAATCGGATTCATTGATATTCCTGGTATAGTAGATGCTATTACACATACAATCATACTCAATTCGATGGAATTGTTTGATCTTAAAGGAGATCTTCTATAATTTCGCACGTGAGGGTTATTTCTTGGTTTCGTCCAGTCATTAATAACTTGATTATTTTTAGATAATAGTAGATAGAAACAACGCTTGTAAGGAGTCCTATTAAAACCAAGAAATATAGGCCTGCCTGCCATCCACACCAGAATAAATAGAGTTTTCCGAAAAACCTGCTAGTGGGGAAGACCCCTAGGGATAAGAGACATAGGGCTAAAGAGAGAGCCAAAAAGGATCTTTCGTGTATAATCCTGCATAATCTCGAATGTTATCCGTTCCGGTACGTAGACCAAATAATACAATGCAAGCAAAAGTTCCTAGATTCATGGAGATATAGAACAGCATATAAGTTATCATGCTTGCATATCCATCATTTGAGTCTCCAACAATTATTCCAATAATTACATATCCGATTTGACCTATGGACGAATATGCAAGCATACGTTTCATGCTTGTTTGAGTAATAGCAATGAGATTTCCCAATATCATGCTAAGAATAGCTAGGATTTCCAGAAGAAGATGCCATTCGTTTGATGAGAAATAAAAGGAATATCGAAAATTCAGTGGCTAAAGCTGAAGCAGCTACTTTCGAAGTAACAGAAAGAAAAGCAACGACTGGAGTGGGAGAGTCAGAGTCGAAAAGAGGATTCCTCACTTCTTTCTCTCATTCAAAACCGTGCATGAGACTTTCATCTCACACGGCTCCTAAGTGATAAAAGAAAGAAGAACTCATCTTCTTTCTTTTTGATTACCTTCCTCGCGTATGTATAAGACCGAATCCATTCGATTTCTAAAAAGGATTACTAATCCTTAACTTTTCGAGGAATCCTTCATCAGTGGTTGTGAATGACTGATTTTTTCAATCCTTTCGACCTTGGTTCCGTAGGAGCAAAGCAAGTCAGAAAGATTGAGAAATAGAACCATCTGATTTAATTCGTTCTCAATAGCCATGAGATGATCATCTTAGGGTGATCCTTTGTCGACGGATGCTCTTATTACACTCGTAGTCTCTGAAGGATGAGAACCAACTATGTAGCATCTACATCGAGAATTCAAGTATTGTATACGTCATTAGTCCGATCCTTTGTAGGAACTACCCGTAATAAGGAACTTGCAAAATGGATCTGTTTATCATAAAGAGATTCGTCGTTCTTGACCCCGCTTCACCTTAATTGTTATTTGAACAAGTAAAAGTTCTGTCTTGGTCCGAGTGGGGATAGCATTTCTCTTCTGCATGTCCATGGAGTTTTGAAAAATCCAAACATCTCAGAGATAGATAGAGAGGTAGGAATTTCTCGAACGAACCGCACTCCTTCGTATACGTCAGGAGTCCATTGATGAGAAGGGGCTGGGGAAAGCTTGAACCCAATTCCTACAGTAATGAATATGAGCGCAATTGAAATTCCTGGGGAGTTATACATTTGTGTATTGATAAGACCATTCACTATTTCTTGAAGCTCGATCTCTCCCCGGATGAACCATATAGCCAAGAGAAACCATGAACCAGAATAGAAGAGCTTGCCCCACCCATGAGTAAATATTTCATAGTAGCCTCATTAGACCGTACATCTTTCTTGGTATATCCAGATAATAGGTAGGAACATAAACTGAAACATTCTGGAGCTACAAAGATAGTTATGAAATCGTTAGCACCGCATAAAAACATTCCTCCCAGAGTAGCTGTTAATACGAATAAGAGAAACTCTGTTATAGCCATTTCTGTACATTCAATGTACTCTACGGATAGAGGAATACATAGAGTTGAACATAGTAAAATAAGAAATTGAAAGATTTCGTTGAAATTGTTCGTTTGGAAATTTCCCGAAAAGCTAATCATAGGTTCTTCTCTCCATCGGAACAATAGGGCCGTTATGCTCATTACTAAACTTGTTGAAGAGATGAAATATAACCAAGGTATATCTTTTGATCCGAGGTTATATCGATCATCAGAAGAAGAATTAGGCCAAAAATTAGGATACATTCTGGGAAAATCAAACTTCCATCGAAGAGAAGCAAATGAAAGGCTTTCATAAAAATTCTCGTAGAATCGAGAATGAAGTTTTCATTCTGTACATGCCAGATCATGAATTAGTAACTGCATCCAATTTACAAAAAAATCCCAATTGTGTCGAACTTTCCATGGAATATTTACGGAATCTCCATGAATAGGATCAACCCCTATTCCATGGTATTTACATGAGGTTCCTCTTTAAGAAAGTCCCCGAGAGGGCTTAGTTGATCCATGATTTATATTTATGTTTCATCTTTCGTTTGTTTCGAGAAATCTATCGATCAATTCCGATTCTTTCTTTTCTCTTGATTCTTTTCCGATCGAGATGTATAGATCCTGTTCATGGATTAACGAAAATGTGCAAAAGCTCTATTTGCCTCTGCCATTCTATGAGTCTCTTCCTTTTGCGTATGGCATCGCCACTCCCTTTGGCAGCATCCACTAATTCGGAACTTAATTTGAAAGCCATATTTCGACCCGGACGTTTTCGGGATGCCGCTAATAACCAACGAATGGCAAGTGCTTTTCCTTGCGTGGATCCTATTTCAATGGGAACTTGATGAGTCGATCCGCCTACACGTCTTGCTTTTACTGTTATATCGGGAGTTACTCCACGTATTGCTTGACGTAAAACAGATAGTGGATTTGTTTCTGTCTTTTGTTGAATCTTTTCACGGCTCGATAGATAATTTGATAAGCCAATGATTTTTTCCGTGTTTCAGAATACGGTTAACCAACATGTTAACTAATCGATTACGATAAATTGGATCGGATTTGGCTGTTTTTCCTTCTGCAGCACCTCGACGTGACATGAGCGTGAAAGGGGTTGAAGAATCCGTTTTCTTTTATAAGGGCTAAAATCACTTATTTTGGCTTTTTACCCCATATTGTAGGGTGGATCTCGAAAGATATGAAAGATCTCCTCCAAGCCGTACATACGACTTTCATCGAATACGGCTTTCCGCAGAATTCTATATGTATCTATGAGATCGAGTATGGAATTCTGTTTACTCACTTTAAATTGAGTATCCCTTTCCCTTTCCTTCTAGGATGGGAAATCCTGTATTTTACATATCCATACGATTGAGTCCTTAGGTTTCCGAAATAGTGTAAAAAGTGCTTCGAATCATTGCTATTTGACTCGGACCTGTTCTAAAAAGTCGAGGTATTTCGAATTGTTTGTTGACACGGACAAAGTCAGGGAAAACCTCTGAAATTATTTCAATATTGAACCTTGGACATATAAGAGTTCCGAATCGAATCTCTTTAGAAAGAAGATTTTTGTCTCATGGTAGCCTGCTCCAGTCCCCTTACGAAACTTTCGTTATTGGGTTAGCCATACACTTCACATGTTTCTAGCGATTCACATGGCATCATCAAATGATACAAGTCTTGGATAAGAATCTACAACGCACTAGAACGCCCTTGTTGACGATCCTTTACTCCGACAGCATCTAGGGTTCCTCGAACAATGTGATATCTCACACCGGGTAAATCCTTAACCCTTCCCCTCTTACTAAGACTACAGAATGTTCTTGTAAATTATGGCCAATACCGGGTATATAAGCAGTGATTTCAAATCCAGAGGTTAATCGTACTCTGGCAACTTTACGTAAGGCAGAGTTTGGTTTTTGGGGTGATAGTGGAAAAGTTGACAGATAAGCCACCCTTACTGCCACTCTACAGAACCGTACATGAGATTTTCACCTCATACGGCTCCTCGTTCAATTCTTTCGAATTCATTGGATCCTTTTCCGCGTTCGAGAATCCCTCCCTTCTTCCACTCTGTCCCGAAGAGTAACTAGGACCAATTTAGTCACGTTTTCATGTTCCAATTGAACACTTTCCATTTTGATTATTCTCAAAGGAGAAGATTCTTCTCTTTAACAAACATATGGGGATCCAATCACGATCTTATACTAAGAACAAGAGATCTTTGCCCCTCAGTCTTCGAGAATCGGAAAGATCCTTTTAATTTGTTCATTTGGAATCTAGGTTCTTCTACTTCATTTTTATTTAATATTTTTCCTCTCTTTTTTATATCATTCCTTAAGTCCCATAGGTTTGATCCTGTAGAATTTGACCCATTTCTCATTGAACGAAGGGTACGAAATCAATCTGATTGATTTTTCGATCAAAAGTACTATGTGAAATCTTCGGTTTTCCTCTTCCTCTATCCCTATCCCATAGGTATAGGTACAGTGTTTGAATAAATAGAGAGCCCTTTCTTCTGTATGAATCGATATTATTCCATTCCAATTCCTTCCCGATACCTCCCAAGGAAAATCTCGAATTGGATCCCAAATTGACGGGTTAGTGTGAGCTTATCCATGCGGTTATGCACTCTTTGAATAGGAATCCGTTTTCTGAAAGATCCAGGCTTTCATACTTTGGTGGGTCTCCGAGATCCTTTCGATGACCTATGTTGTGTTGAAGGGATATCTATCTAATCCGATGATCGATTGCGTAAAGCCCGCGGTAGCAACGGAGCCGGGGAAAGTATACAGAAAAGACAGCTCCAGCTCTTTTCTATTATATTAGTATTTTCTATTCTATTAGATTAGTATTAGTTAGTGATCCCGGCTTAGTGAGTCCCTTCTTCCGTGATGAACCGTTGGCACCTGTCCTACATTTTGTCTCTGTGGACCGAGGAGAAAAGGGCTCGGCGGGAAGAGGGTGTGCCATGAGAGAAGCAAGGAGGTCAACCTCTTTCAAATATACAACATGGATTCTGGCAATGTAATGTAGTTGGACTCTCATGTCGATCCGAATGAATCATCCTTTCTACGGAGGTCCATCTTTGCCTTCTAGGCAAGAGGATAGCAAGTTACAAATTCTGTCTCGGTAGGACATCTATTTCTATTACTATGAAATTCATAAATGAAGTAGTTTGGGGTTACCATTATCCTTTTGTAGTGACGAATCTTCTATGTGTTCCTAAGAAAAGGAATTTGTCCATTTTCGGGGTCTCAAAGGAGCGTGGAAACACATAAGAACTCTTGAATGGAAATGGAAAGAGATGTAACTCCAGTTCCTTCGGAATCGGCAGTCAATCCTATTTCCGATAGGGGCAGTTGAATCCGATTTTGACCATTATTTTCATATCCGTAATAGTGCGAAAAGAAGGCCCGGCTCCAAGTTGTTCAAGAATAGTGGCGTTGAGTTTCTCGACCCTTTGACTTAGGATTAGTCAGTTCTATTTCTCGATGCGGGCAGGGAAGGGATATAACTCAGCGGTAGAGTGTCACCTTGACGTGGTGGAAGTCATCAGTTCGAGCCTGATTATCCCTAAACCCAATGTGAGTTTTTCTATTTGGCTTTGCTCCCCGCCGCGATTCAATGAGAATGGATAAGAGGCTCGTGGGATTGACGTGAGGGGCAGGGATGGCTATATTTCTGGGAGCGAACTCCGGGCGAATATGAAGCGCATGGATACAAGTTATGCCTTGAAATGAAAGACAATTCCGAATCCGCTTTGTCTACGAACAAGGAAGCTATAAGTAATGCAACTATGAATCTCATGGAGAGTTCGATCCTGGCTCAGGATGAACGCTGGCGGCATGCTTAACACATGCAAGTCGGACGGGAAGTGGTGTTTCCAGTGGCGGACGGGTGAGTAACGCGTAAGAACCTGCCCTTGGGAGGGGAACAACAGCTGGAAACGGCTGCTAATACCCCGTAGGCTGAGGAGCAAAGGAGGAATCCGCCCGAGGAGGGGCTCGCGTCTGATTAGCTAGTTGGTGAGGCAATAGCTTACCAAGGCGATGATCAGTAGCTGGTCCGAGAGGATGATCAGCCACACTGGGACTGAGACACGGCCCAGACTCCTACGGGAGGCAGCAGTGGGGAATTTTCCGCAATGGGCGAAAGCCTGACGGAGCAATGCCGCGTGGAGGTAGAAGGCCCACGGGTCGTGAACTTCTTTTCCCGGAGAAGAAGCAATGACGGTATCTGGGGAATAAGCATCGGCTAACTCTGTGCCAGCAGCCGCGGTAATACAGAGGATGCAAGCGTTATCCGGAATGATTGGGCGTAAAGCGTCTGTAGGTGGCTTTTTAAGTCCGCCGTCAAATCCCAGGGCTCAACCCTGGACAGGCGGTGGAAACTACCAAGCTGGAGTACGGTAGGGGCAGAGGAATTTCCGGTGGAGCGGTGAAATGCGTAGAGATCGGAAAGAACACCAACGGCGAAAGCACTCTGCTGGGCCGACACTGACACTGAGAGACGAAAGCTAGGGGAGCGAATGGGATTAGATACCCCAGTAGTCCTAGCCGTAAACGATGGATACTAGGCGCTGTGCGTATCGACCCGTGCAGTGCTGTAGCTAACGCGTTAAGTATCCCGCCTGGGGAGTACGTTCGCAAGAATGAAACTCAAAGGAATTGACGGGGCCCGCACAAGCGGTGGAGCATGTGGTTTAATTCGATGCAAAGCGAAGAACCTTACCAGGGCTTGACATGCCGCGAACCCTCTTGAAAGAGGGGGTGCCTTCGGGAACGCGGACACAGGTGGTGCATGGCTGTCGTCAGCTCGTGCCGTAAGGTGTTGGGTTAAGTCCCGCAACGAGCGCAACCCCGTGTTTAGTTGCCGTCGTTGAGTTTGGAACCCTGAACAGACTGCCGGTGATAAGCCGGAGGAAGGTGAGGATGACGTCAAGTCATCATGCCCCTTATGCCCTGGGCGACACACGTGCTACAATGGCCGGGACAAAGGGTCGCGATCCCGCGAGGGTGAGCTAACCCCAAAACCCGTCCTCAGTTCGGATTGCAGGCTGCAACTCGCCTGCATGAAGCCGGAATCGCTAGTAATCGCCGGTCAGCCATACGGCGGTGAATCCGTTCCCGGGCCTTGTACACACCGCCCGTCACACTATGGGAGCTGGCCATGCCCGAAGTCGTTACCTTAACCGCAAGGAGGGGATGCCGAAGGCAGGGCTAGTGACTGGAGTGAAGTCGTAACAAGGTAGCCGTACTGGAAGGTGCGGCTGGATCACCTCCTTTTCAGGGAGAGCTAATGCTTGTTGGGTATTTTGGTTTGACACTGCTTCACACCCAAAAGAAGGTAGCTACGTCTGAGTTAAACTTGGAGATGGAAGTCTTCTTTCCTTTCTCGACGGTGAAGTAAGACCAAGCCCATGAGCTTATTATCCTAGGTCGGAACAAGTTGATAGGATCCCTTTTTACGTCCCTGTGCCCCCGTGTGGCGACATGGGGGCGAAAGAAGGAAAGAGAGGGATGGGGTTTCCCTCGCTTTTGGCATAGCGGGCCCCAGTGGGAGGCTCGCACGACGGGCTATTAGCTCAGTGGTAGAGCGCGCCCCTGATAATTGCGTCGTTGTGCCTGGGCTGTGAGGGCTCTCAGCCACATGGATAGTTCAATGTGCTCATCGGCGCCTGACCCTGAGATGTGGATCATCCAAGGCACATTAGCATGGCGTACTCCTCCTGTTCGAACCGGGGTTTGAAACTAAACTTCTCCTCAGGAGGATAGATGGGGCGATTCGGGTGAGATCCAATGTAGATCCAACTTTCGATTCACTCGTGGGATCCGGGCGGTCCGGGGGACCACCGCGGCTCCTCTCTTCTCGAGAATCCATACATCCCTTATCAGTGTATGGACAGCTATCTCTCGAGCACAGGTTTAGGTTCGGCCTCAATGGGAAAATAAAATGGAGCACCTAACAACGCATCCTCACAGACCAAGAACTACGAGATCACCCCTTTCATTCTGGGGTGACGGAGGATCGTACCATTCGAGCCGTTTTTTGTCATGCTTTTCCCGGAGGTCTGGAGAAAGCTGCAATCAATAGGATTTCCTAATCCTCCCTTCCCGAAAGGAAGAGCGTGAAATTCTTTTTCCTTTCCGCAGGGACCAAGAGATTGGATCTAGCCGTAAGAAGAATGCTTGGTATAAATAACTCACTTCTTGGTCTTCGACCCCTCAGTCACTACGAACGCCCCGATCAGTGCAATGGGATGTGTCTATTTATCTATCTCTTGACTCGAAATGGGAGCAGGTTTGAAAAAGGATCTTAGAGTGTCTAGGGTTGGGCCAGGAGGGTCTCTTAACGCCTTCTTTTTCTTCTCATCGGAGTTCTTTCATAAAGACTTGCCAGGGTAAGGAAGAAGGGGGAACAAGCACACTTGGAGAGCGCAGTACAACGGAGAGTTGTATGCTGCGTTCGGGAAGGATGAATCGCTCCCGAAAAGGAATCTATTGATTCTCTCCCAATTGGTTGGACCGTAGGTGCGATGATTTACTTCACGGGCGAGGTCTCTGGTTCAAGTCCAGGATGGCCCAGCTGCGCCAGGGAAAAGAATAGAAGAAGCATCTGACTACTTCATGCATGCTCCGCTTGGCTCGGGGATATAGCTCAGTTGGTAGAGCTCCGCTCTTGCAATTGGGTCGTTGCGATTACGGGTTGGATGTCTAATTGTCCGGGCGGTAATGATAGTATCTTGTACCTGAACCGGTGGCTCACTTTTTCTAAGTAATGGGGAAGAGGACCAAAACATGCCACTGAAAGACTCTACTGAGACAAAGATAAGGCTGTCAAGAACGTAGAGGAGGTAGGATGGGCAGTTGGTCAGATCTAGTATGGATCGTACATGGACGGTAGTTGGAGTCGGCGGCTCTCCCAGGGTTCCCTCATCTGAGATCCCTGGGGAAGAGGATCAAGTTGGCCCTTGCGAACAGCTTGATGCACTATCTCCCTTCAACCCTTTGAGCGAAATGCGGCAAAAGAAAAGGAAGGAAAATCCATGGACCGACCCCATCATCTCCACCCCGTAGGAACTACGAGATCACCCCAAGGACGCCTTCGGCATCCAGGGGTCACGGACCGACCATAGAACCCTGTTCAATAAGTGGAACGCATTAGCTGTCCGCTCTCAGGTTGGGCAGTAAGGGTCGGAGAAGGGCAATCACTCATTCTTAAAACCAGCGTTCTTAAGGCCGACCAAAGAGTCGGGCGGAAAAGGGGAAAGCTCTCCGTTCCTGGTTCTCCTGTAGCTGGATCCTCCGGAACCACAAGAATCCTTAGTTAGAATGGGATTCCAACTCAGCACCTTTTGAGTGAGATTTTGGGAAGAGTTGCTCTTTGGAGAGCACAGTACGATGAAAGTTGTAAGCTGTGTTCGGGGGAGTTATTGTCTATCGTTGGCCTCTATGGTAAAATCAGTCGGGGGACCTGAGAGGCGGTGGTTTACCCTGCGGCGGATGTCAGCGGTTCGAGTCCGCTTATCTCCAACTCGTGAACTTAGCCGATACAAAGCTATATGATAGCACCCAATTTTTCCGATTCGGCGGTTCGATCTATGATTTATCATTCATGGACGTTGATAAGATCCATCCATTTAGCAGCACCTTAGGATGGCATAGCCTTAAAAAAATAAAAGGGAAGGGCGAGGTTCAAACGAGGAAAGGCTTACGGTGGATACCTAGGCACCCAGAGACGAGGAAGGGCGTAGTAATCGACGAAATGCTCCGGGAGTTGAAAATAAGCATAGATCCGGAGATTCCCGAATAGGGCAACCTTTCGAACTGCTGCTGAATCCATGGGCAGGCAAGAGACAACCTGGCGAACTGAAACATCTTAGTAGCCAGAGGAAAAGAAAGCAAAAGCGATTCCCGTAGTAGCGGCGAGCGAAATGGGAGCAGCCTAAACCGTGAAAACGGGGTTGTGGGAGAGCAACACAAGCGTCGTGCTGCTAGGCGAAGCAGCCCGAATGCTGCACCCTAGATGGCGAAAGTCCAGTAGCCGAAAGCATCACTAGCTTATGCTCTGACCCGAGTAGCATGGGGCACGTGGAATCCCGTGTGAATCAGCAAGGACCACCTTGCAAGGCTAAATACTCCTGGGTGACCGATAGCGAAGTAGTACCGTGAGGGAAGGGTGAAAAGAACCCCATCGGGGAGTGAAATAGAACATGAAACCGTAAGCTCCCAAGCAGTGGGAGGAGCCTAGGGCTCTGACCGCGTGCCTGTTGAAGAATGAGCCGGCGACTCATAGGCAGTGGCTTGGTTAAGGGAACCCACCGGAGCCGTAGCGAAAGCGAGTCTTCATAGGGCAATTGTCACTGCTTATGGACCCGAACCTGGGTGATCTATCCATGACCAGGATGAAGCTTGGGTGAAACTAAGTGGAGGTCCGAACCGACTGATGTTGAAGAATCAGCGGATGAGTTGTGGTTAGGGGTGAAATGCCACTCGAACCCAGAGCTAGCTGGTTCTCCCGAAATGCGTTGAGGCGCAGCAGTTGACTGGACATCTAGGGTAAAGCACTGTTTCGGTGCGGGCCGCGAGAGCGGTACCAAATCGAGGCAAACTCTGAATACTAGATATGACCTCAAAATAACGGGGGTCAAGGTCGGCCAGTGAGACGATGGGGATAAGCTTCATCGTCGAGAGGGAAACAGCCCGGATCACCAGCTAAGGCCCCTAAATGACCGCTCAGTGATAAAGGAGGTAGGGGTGCAGAGACAGCCAGGAGGTTTGCCTAGAAGCAGCCACCCTTGAAAGAGTGCGTAATAGCTCACTGATCGAGCGCTCTTGCGCCGAAGATGAACGGGGCTAAGCGATCTGCCGAAGCTGTGGGATGTAAAAATACATCATCGGTAGGGGAGCGTTCCGCCTTAGAGAGAAGCCTCCGCGCGAGCAGTGGTGGACGAAGCGGAAGCGAGAATGTCGGCTTGAGTAACGCAAACATTGGTGAGAATCCAATGCCCCGAAAACCTAAGGGTTCCTCCGCAAGGTTCGTCCACGGAGGGTGAGTCAGGGCCTAAGATCAGGCCGAAAGGCGTAGTCGATGGACAACAGGTGAATATTCCTGTACTACCGTTTGTTGGTCCCGAGGGACGGAGGAGGCTAGGTTAGCCGAAAGATGGTTATCGGTTCAAGAACGTAAGGTGCCCCTGCTTTTCAGGGTAAGAAGGGGTAGAGAAAATGCCTCGAGCCAATGTTCGAGTACCAGGCGCTACGGCGCTGAAGTAACCCATGCCATACTCCCAGGAAAAGCTCGAAGGACTGTAAGCAAAGGGTACCTGTACCCGAAACCGACACAGGTGGGTAGGTAGAGAATACCTAGGGGCGCGAGACAACTCTCTCTAAGGAACTCGGCAAAATAGCCCCGTAACTTCGGGAGAAGGGTGCCTCCTCACAAAGGGGTCGCAGTGACCAGGCCCGGGCGACTGTTTACCAAAAACACAGGTCTCCGCAAAGTCGTAAGACCATGTATGGGGGCTGACGCCTGCCCAGTGCCGGAAGGTCAAGGAAGTTGGTGACCTGATGACAGGGGAGCCGGCGACCGAAGCCCCGGTGAACGGCGGCCGTAACTATAACGGTCCTAAGGTAGCGAAATTCCTTGTCGGGTAAGTTCCGACCCGCACGAAAGGCGTAACGATCTGGGCACTGTCTCGGAGAGAGGCTCGGTGAAATAGACATGTCTGTGAAGATGCGGACTACCTGCACCTGGACAGAAAGACCCTATGAAGCTTCACTGTTCCCTGGGATTGGCTTTGGGCCTTTCCTGCGCAGCTTAGGTGGAAGGCGAAGAAGGCCCGGGGGCCTGAGCCATCAGTGAGATACCACTCTGGAAGAGCTAGAATTCTAACCTTGTGTCAGGACCTACGGGCCAAGGGACAGTCTCAGGTAGACAGTTTCTATGGGGCGTAGGCCTCCCAAAAGGTAACGGAGGCGTGCAAAGGTTTCCTCGGGCCGGACGGAGATTGGCCCTCGAGTGCAAAGGCAGAAGGGAGCTTGACTGCAAGACCCACCCGTCGAGCAGGGACGAAAGTCGGCCTTAGTGATCCGACGGTGCCGAGTGGAAGGGCCGTCGCTCAACGGATAAAAGTTACTCTAGGGATAACAGGCTGATCTTCCCCAAGAGCTCACATCGACGGAAGGTTTGGCACCTCGATGTCGGCTCTTCGCCACCTGGGGCTGTAGTATGTTCCAAGGGTTGGGCTGTTCGCCCATTAAAGCGGTACGTGAGCTGGGTTCAGAACGTCGTGAGACAGTTCGGTCCATATCCGGTGTGGGCGTTAGAGCATTGAGAGGACCTTTCCCTAGTACGAGAGGACCGGGAAGGACGCACCTCTGGTGTACCAGTTATCGTGCCCACGGTAAACGCTGGGTAGCCAAGTGCGGAGCGGATAACTGCTGAAAGCATCTAAGTAGTAAGCCCACCCCAAGATGAGTGCTCTCCTATTCCGACTTCCCCAGAGCCTCCGGTAGCACAGCCGGGATAGCGACGGGTTCTCTGCCCTGCGGGATGGAGCGACAGAAGTTTTGAGAATTCAAGAGAAGGTCACGGCGAGACGAGCCGTTTATCATTACGATAGGTGTCAAGTGGAAGTGCAGTGATGTATGCAGCTGAGGCATCCTAACAGACCGGTAGACTTGAACCTTGTTCCTACATGACCTGATCAATTCGATCAGGCACTCGCCATCTATTTTCATTGTTCAAATCTTTGACAACACGAAAAACATTGTTCAACTCTTTGACAACATGAAAAACCAAAAGCTCTGCCCTTCCATCCCAGAGGAGAGCAGAGCTTTTGGTGTCCCCTCCAGTCAAGAATTGGGGCCTCATAATCACTAGCCAACATGCTTTTCTCTCATGCCTTTCTTCGTTCATGGTTCGATATTCTGGTGTCCTAGGCGTAGAGGAACCACACCAATCCATCCCGAACTTGGTGGTTAAACTCTACTGCGGTGACGATACTGTAGGGAGGTCCTGCGGAAAAATAGCTCGACGCCAGGATGATAAAAAGCTTAACACCTCTCATTCTTATTACTTTTCAATATGAAAACGAAAAATGAAAAATCAAAAGGTCGTCTTATTCAAAACCCCAATTATGACATCCCTTCTCTCCCACTTCACACCTCGGAACGCACCTCTTATAGAGATAAACGCGCCTTCACATCTTCTTAACCCGAAATTGAAATGGCTGGGGAGAGGAAAGAGTCCTTTTTGGGGTACTCCCGGGAACAGATCCGCTGGAGACGAGGTGGGGCCTGTAGCTCAGAGGATTAGAGCACGTGGCTACGAACCACGGTGTCGGGGGTTCGAATCCTCCTCGCCCACAACCGGCCCAAAGGGAAGTACCTTTCCCTCTGGGAGTAGAATGATGGGGATAGCGGACCAAAAAAGCTATGGAACTTGGGTGTGGGTCTTTTGTCGAAATGGAATGGCTTTTCTTTTTTATCTATTTATTGTGGATTTATTGTAGATGGTGGAATCATTACACATAGTATGCCCCGTCCGCGTATTTTTTGTTTTACGCTCCGTAAATCTTCCTCAGCCAGGCTTGGGCAGAATAGCAGAGAAAGTACAAGTATTAGTAGCATAACAAAAGCCCTCCCCGTCATTAATATCTTGCGGCAATTGTGACCTCTCAGGAGAATCGATGACTGCATCTTTGATGCAGTGCTAGTACTAGTAATTCTTTAATTCTTAATTGGCTAGTTGGAAATAGCCCCAGGGCTATGGAACAAAGGATTATCCCAGACCTACACCGAGGTATTGACGGTGATTTTCAAATCTCGCAGAATGTGATACGATGGGATAGAATGCAAAGACAGGGAACGGGTTACCTACTCTTAACGGTCAAAGCGAGCCTTTATTCTGAATTCTTTAATTCAGAATGAATCAAATCTCCCAAGTAGGATTCGAACCTACGACCAATCGGTTAACAGCCGACCGCTCTACCACTGAGCTACTGAGGAACAACGGGAGATTAGATCTCATAGAGTTCAATTCCTGTTCTCAACCCATGACCAATATGAGCTAGAAGCTTCCTTCGTAACCCCGGAACTTCTTCGTAGTGGCTCCCTCCATGCCTCATTTCAGAGGAACCTCAAAGTGGCTCTATTTCATTATATTCCATCCATATCCCAATTCCATTCATTTAATATCCCTTTGGTGTCATTGACATAACAGATGTCGTTTCTAGTCTATCTCTTTCTATTTCTTTTCTATATATGGAAAGTTCAAAAATCATCATATAATAATCCAGAAATTGCAATAGAAAAGAAATAGGGAGGTTTGTGATGATTTTCAATCTTTTCTACTAGGTAATCTAGTATCCTTATGCATGAAGATAATCAATTCGGTCGTTGTGGTCGGACTCTATTATGGATTTCTGACCACATTCTCCATAGGGCCCTCTTATCTCTTCCTTCTCCGAGCTCAGGTTATGGAAGAAGGAACCGAGAAGAAGGTATCAGCAACAACTGGTTTTATTACAGGACAGCTCATGATGTTCATATCGATCTATTATGCGCCTCTGCATCTAGCATTGGGTAGACCTCATACAATAACTGTCCTAGCTCTACCATATCTTTTGTTTCATTTCTTCTGGAACAATCACAAACACTTTTTGATTATGGATCTACTACCCGAAACTTAATGCGTAATCTCAGCATTCAATGTGTATTCCTGAATAATCTCATTTTTCAATTATTCAACTATTTCCTTTTACCAAGTTCAATGTTAGCCAGATTAGTCAACATTTATATGTTTCGATGCAACAACAAGATGTTATTTGTAACAAGTAGTTTTGTTGGTTGGTTAATTGGTCACATTTTATTCATGAAATGGCTTGGATTGGTATTAGTCTGGATACGGCAAAATCATTCTATTAGATCGAATAAGTACATTCGATCTAATAAGTACCTTGTGTCAGAATTGAGAAATTCTATGGCTCGGATCTTTAGTATTCTCTTATTTATTACCTGTGTCTACTATTTAGGCAGAATACCGTCACCCATTCTTACTAAGAAACTGAAAGAAACCTCAAAAACGGAAGAAAGGGTGGAAAGTGAGGAAGAAAGAGATGTAGAAATAGAAACAACTTCCGAAATGAAGGGGATTAAACAGGAACAAGAGGGATCCACCGAAGAAGATCCTTCTTCTTCCCTTTTTCGGAAGAAAGGGAGGATCCGGACAAAATCGATGAAACGGAAGAAATCCGAGTGAATGGAAAGGAAAAACAAAGGATGAATTCGACAGTAATAAATAGTAATAAATTAAATAGTAATAAATATCTTAAGTAAAATATCTTAAGTATAAGTAATATTAAGTAATAATTCTAATAATTACAAATAATTACATAAGTAATATATATTATATTAAATATTAAGTAATAAAAGTAATAATAAAAATGAATAAACAAATCAATACATACGATAAATAGAAGAAAAGATAAGAAGAGATGCGCCTGCCCCTACATATTTGATAGCTTCTCCTATAAAGAAACTAAAAAACAACCCCATTTGTAATTCCATCAATTATTCGTCGATCAAAAAATGAGTTAATTCAGCCAATCCTCTAGTTCCCCAGTAAAGAATCTTGTATAAAAAGAATCTATATAAGCACGATTATATGACCAACTATAAATGCCATTTATAATTTTTCCCAAAGAATTCCCTTAGGACCTCTTTTAACAGCTGAATTAATTAAATCCAAATTTAGTAAAGAGGAATAAGGAGATTTATATAAAAAGACGCTATAAATATTCCTAAAAACCTATACTGACTGAAAAATTGCATCTTTCAAAATTCATACCAATCCGTCGAATTAGTGAACTTTTGGTGCAAAAGATTTATAGACGGAGATAACCATTTAGATAATATATCAACATTGACTCCCTCTTGATTAAAAGGAATTCCTAGAAACCCAACGAATAGAGTAAATAGGCCCAATACAAGTAGAGGAAATAACATAGTATTGTCTGATTCATAAGGATAGGAACCAAGTTTTTATTCTCAAAATGAGCAATAGTAATATAGGGTCGTGTCAAATTTCTACGATTATCATCAATTCGATATGTTCTTTTTGAAAAAGGAACAAGTTTTATTATCAGTCATTCCTAACAAACGAACATTTTGGTTAATTCTTTTGAAATTCCCTTACCCCATAGAGATATTGAATAGAAAGGTATTTTTTGTTTGCCACTATAATTTTGAAAAGAAACATTTAAATGTCCCTCAAAGGTAAGTAAATAGATCCGAAACATATAAAATGCGGTTAATCCGGCAGTAACCCAGGCTATTATTGCGAAAATCGTCGAATACAACCAAGTATCGTTAAGAATTTCATCTTTGGACCAAAAAACAAGCCAAAGGCGGAATACCACAAAGAGAGTGTACCTAATAAAAAGCATTTTTGGTAATTGGTACATGCTTTAAACCTCCCATAAGAATCATATTCTGACTCTTATAGGAGAATAGCCAACAATTCCTTCCATTGAATGAATAACGGATCCGGATCCTAAAAATAACAATGCTTTGGAATAGGCATGAGTAATCAAATGAAATAAAGCACTTCGATAAGACCCCATACCGAGGGCTAACATCATATAACCCAATTGAGACATTGTCGAATAGGCTAAACCTCTCTTAATGTCTTTTTGAGCAAGAGCTAAAGTAGCTCCTAATAACACTGTTATTATTGCTATCAAGGAGATTAAATTCATTATGGAAGGTATAATTATGAAAAGAGGAAGAAGCCGAGCTACAAGAAATTCCTGCCGCTACCATAGTAGCAGCATGTATAAGAGCGGAAATCGGAGTAGGCCCTTCCATAGCATCGGGCAACCATACATGAAGGGAAATTGTGCAGATTTAGCAACGGCACCAGCAAATAACAGAACAGCACACAAAGTAACAAATAAAAATTGACCTCATTATTAGAAATTAAGTTATTGAATATTTCGAATAAATCCTGAAATTCAAAACTACCCGTTATCCAATAAAAACCTAAAATTCCTAATAATAAACCAAAATCCCTACACGATTTGTTACAAAAGCCTTTTGGCAAGCATTTGCTGCAAGAGGTCGTGTAAAAATCCTATTAATAAATAGGAACACATTCCAACCAATTCCCAAAAATATAAATTTGTATCAAATTCGAACTAGTAACTAATCCTAACATGGAAGTACTGAAAAACTCATATAAGCAAAAATCTCAAATATGCTTGATCATGAGCCATATAATTATCACTATAAATAAGAACCATAATTCCAACGGTAGTGATTAATATTGACATAATAGAAGTAAGTGGATCTATCAAATAGCCGAATTCTAAAGAAAAATCGTTAGTAATGATCCAAGACCATACATATTGATAGCTCGAATTGCTATTTATTTGCTGAATAGACAGATTCATTGAAAAATCATGACTATACTTAACAATAAAACACTATGAAAAGACCACATGCGGCGAAAAGTTTTTGTTGCCGTCGGAAAAAGAAGCCCCACCCCTATTAATATAGGAACGGGAAGTGGGATGAAAGGTATGAGCCGCCCGTATTGATATGTCTGTTGCATAAAAAGCTTGTTGAATTATGAATTTCCTAATTTATTGTTTCCGATTGACCGGATCTTGCCTCTTTGAAAGGAGTCAGTAAAAGTCAAGATATCGACTAAGTTAAACTAATTTAAATAGAAATTTAGAAGCTTTTCTTCTTACTTTACTTATTCTTAACTTATTATTTTATTTTTATAAATTTTAAAAATCCTTCAAATATTCAATTCAAATCAAGAAGTTAGATTTGGTCAAATGATATAAAACAGAGTAATTCCTAATTTTTCTCAATTTTAAAATGAAACCTACCCCGTTTAACCGGTTAATAAAAAGAAAATGAAAATGGAAGGTTGGATTCTTTTTTTTATTATTAATATTTAATTCGACTTCTATGGTGCAGGAGATTCTATAGATATAGACTTTAATGAGAAAGAATATCAATAAAGATACTAATCAATCGAATGAATAAAAATGATTTTACAGCTATTCTATAGAATAAAAAAGTTTGAAAGAAAAAAATCACATATCTTCTCGTCTTTCTCTATTTCTAGTATTTCGAAAACGCGAAATATTATTTTTATTTTATTGATGCGATTTTCATATATCTTCCCCCAGCTTTCTTTTTTCTGAAAAGAAGAATAAATAGAATAAAATAAAAGTTTAAAGAAGGATTTGTTTTGAACAATAGATGTCTTTCACATCCAACTAGAACAATAAGTAATCCTTTTTATCTTAAATGGCCGTTCCAAAAACGTATTTCTACATCAAAAGCGTATTCGAAAAATATTTGGAAAAGGAAGGGATATTGGACAGCGTTAAAAGCTTTTCGTTAGGGAAATCTCTTTCTACAGGAAATTCAAAAGTTTTTTGTGCAACAAACAAATAAGTAATTAAAATTTAAATAATCTGAATCGACTCGAAAAATGAAATTGACCCATTTCCTTTTGCTACAAAATTATGAATTTCCACTATCTATTGAGTTAAGCTAATCAATATAAAATGGGACTCAGCTGCCTCTTTTATATTTAAAAAAACAATTTAGCTTTTTAGTGAATTCTAAAATACTTTCTTTGTTGACTAACACATAAATACAAGGTTATCCTTCTGTTTTTGGTAGATTTTATCATTTACAAGATGGGGAGGTTTTTCCCCATCGAACTATTTGTTAGATTTACGATAATAAAATTTATTATTTTTCTCCTCTTTTCTCTATCTATAAAATATAAAAAGGGCTAATCTTTAAATTTGAATTTCATTTTATTGAAAAAAGTAATAGATTCGATTTAACGTTACTTAATTTATTTTATATTCTATGAATTACTATATAGTATATATTCCCATATATTTCCTGTTATCAACCCAATTAAATCAAGACTTTAGTGAGAATATTCTAGTGGGAATATTCAGAATATTCTGTATGAATAATGTGTCAATTTTCAGTACCTTCGCCTTTCTATTTTTATCTTCATCGAAAAAAATGCATTGGTAAATTTCTGAAATTCAATAAATGAGAAAAAGTTCATTAAAAATGAAAACCAAAATATTTTTAGGGTAGAACTTTCTAGTTACGAGAGTTCAATTCTAGGAGAACAGAAAATACACGAAAAGCCCCAAGACGCTAAGAAAAATAATGAACTTTCAAATCCCTATTATTTTGTATTATTTTAATTTTGTTTGAGTAATTTAAAAGTTTTCTGAAAAAAAATAAAAATATTCCACTGAATTGGCTTCTTCAATCTCGACGATTGTTCATTCATAAGCTATTATAAGTTCAAGACAAGCCGCTATGGTGAAATTGGTAGACACGCTGCTCTTAGGAAGCAGTGCTAGAGCATCTCGGTTCGAGTCCGAGTGGCGGCATGTCATCTTCTAAAAAGAGAAATAGATCCTATAATGAATTCAACTCCCGATTTCCATTTAAGAGACTCTTCTTTTTATGATATTTTCAACCTTAGAACATATATTAACTCATATTTCCCTTTCCATTGTTTCAATCGTAATTACAATTCGTTTAATAACCTTTTTGCGTAGATGAAATCGTACAACTGTACGATTCATCCGAAAAGGGTCTGATAGTTAGTTTTTTCTGTATAACAGGATTATTAGTTATGCGTTGGATTTATTCGGGTCATTTTCCACTAAGTGATTTATATGAATCATTAATTTTCCTTTCATGGGCTTTCTCCCTTATTCATATAGTTCCGTATTTCAAACAAAATCAAAATTATTTAAGCACAATAACCGGTTCAAGTGCTATTTTTACTCAGGGCTTTGCTACTTCCGGACTTTTAACTCAAATACACCAATCTTCAATATTAGTACCCGCCCTTCAATCCGAGTGGTTAATAATGCACGTAACTATGATGATATTGGGCTATGCGTCCCTTTATGTGGATCATTGTTATCAGTAGCCCTTGTAGTCATTATATTTCGAAAAACAGAAAGATTCTTTGTAAAAGTACTCTTATTAAAAGAGTCGTTTTTGTTGGTGAAATTGAATACATGAATAAAAGAAACCATCTTTTACAAACTACTTCCTTTTTTCGGGTAAGAATTATTACAGATCTCAATTAATTCAACAATTGGATTATTGGAGTTATCGGATTATTAGTCTAGGTTTTTTTTTTAACCATAGGTATTCTGTCAGGAGCAGTGTGGGCTAACGAAGCATGGGGATCCTATTGGAATTGGGATCCAAAAGAAACTTGGGCATTTATTACTTGGATCGTATTCGCGATTTATTTACATACTCGAACAAATATAAACCCGCAAAGTGCAAATTCGGCAATTGTAGCGTCTATAGGCTTTCTTATAATTTGGATATGCTATTTTGGGGTTAATCTATTAGGACTAGGGCTACATAGTTATGGTTCGTTTACATTAACATCTAATTAAATTCAAGAAAGTATCTTACGAACACAACACATTTACATTACAGTACATATAAAAACAGTACATATAAAATTTTACGCGAATCGGCACGAACCGTGCGAATCAATACAATATTTGATTTATGTGGTTCGTGCCCATATGGGGTTCAAATTCATTTTTTAGCTTCACTTATAAATATAAAATTAAAATTTGCGAGAAGAAAAAGTTCTCATTTTTCATTCTACAACTTTTTCATTGTAAAGTGAATGGTTTTAAAATCATAATCAATAGCAAAACTATTTAGAAAAAAATTAGATAGGATAACTTCAACCTTGTCAACCGATAGTGACAGAACGAAATCTGGGTACATACCAATACCTAGTACGGGTAAAAAGAGAGAAATAGAAAGAAATAACTCTCGCGGTCCAGAATCAAAAAATAAGAGTTTGGAACGTTAAAAAGCTTATATCCATAGAACATCTGACGTGACATAGATAATGAATAAATAGGAGTTAATATCATTCCAATTGCCATTACAAAATAATTAATATTTTTGGCATTAAAAATATTTGTGACTGGTTATTATTCCAAAAATACTATCAATTCAGCAACAAAGCCACTCATACCCGGTAATGCAAGGGAAGCCATCGCAAAGCTACTAAACATCGTGAATATTTTTGGCATTGTGATAGCTATTCCGCCCATTTCGTCAAGATAAAGAAGGCGTGTTCTATCATAAGTTGTCCCTGCCAAGAAAAAAGTGCAGCACCAATAAATCCATGAGAGATTATTTGTAAAGAGCTCCATTTAGTCCTGTATCAGTTATAGAACCAATTCCGATAATTAGGAAACCCATATGAGATACAGAAGAATAGGCTATTCTTTTTTAAATTCCGTTGGCCAAGAGATGTTGAAGCTGCATAAATGATTTGGATTGCGCCTACTATCACTAACCAAGGGAAAATAGATAATGGGCATGAGATAATAATTCTATATTGATCCGAGCCAGTCCATACGCTCCCATTTTTAATAAGATTCCGGCTAGAAGCATACAAGTACTGTAATGTGCTTCTCCGTGGGTATCTGGTAACCATGTATGTAGGGGTATAATCGGCGATTTGACAGCAAAAGCAATAAAAACCCAATATAAAATATTATTTCTAAGACCGCAGGATACGACTGATTAGCTGATGTTTCAAAATTTAATGTTGGTTCATTAGAACCATATAAACCTATACCCAGAACTCCCAGTAGGAGAAAAATGGAGCCCCTGCCGTGTACAAAATAAATTTTGTAGCCGAGTAGAGACGTTTCTTTCCCCCACATGGATAGAAGTAGATAAACGGGAATTAATTCTAACTCCCACATGAGAAAAAAAGTAAAAGGTTACGAGAAGAAAATGATCCTATTTGACCACTGTACATTGCTAACATCAGGAAATGAAATAATCGAGAATCTCGAGTAACAGGCCAAGCCGATAAAGTAGCTAAGGTCGTGATGAATCCCGTTAGTAAAATGGGTCCTATAGAAAGTCCATCTATTCCCAATTTCCAATGGAAATCAAAAAGCGGATCCATTTATAATCCTCCAATAGTTGGATTAATGGATCGTCTGGTTGGAAATGATAACAGAATGTATAGACCGTTAGAAGGAGTTCTAAAATACATATACATATTGTATACCAGCGAACGACCTTATTTCCCCTATGGGGAGAAAGAAAATTAAGGAACCCGCGGATATTGGCAAAACTACAATTATTGTTAACCAGGGAAAAAATTCGTGGTAAAGACAAGATACGTTTGGACCAGAAAAACCGTGCTCAAAAATAAAAATATATTGAGCACGGGTCTTGTCGGTAAAAAAAAAAAAGAAAATGGATTCAAGTAGAGTTTATTGGAACATATCAATAAGCTAGACCCATACTGCGAGTTGTTTCAGCCCCTAAATAAACCCGAACACTCAAGAAATCCGTTGGGCAGGCGGATTCACATCTTTACAACCAACGCAGTCTTCCGTTCTTGGAGCCGAAGCTATTTGTTTAGCTTTACATCCGTCCCAAGGTATCATTTCTAATACATCGGTCGGGCAGGCTCGGACACATTGAGTACATCCTATACACGTATCATAAATCTTTACTGAATGTGACATCGGATCTATACATTTTTAAACGTCATAAATTTTGACCCAGTAAGTTTATAAACTAATCCTATATTTAGATACCAGGTAAATCAACAAGTTATCAGAACTTTTCCTTCTGGACTGCTTTATTATAAAAGGAAGGGCCAAAATACTTTGATTTCTTATGTTTTTTTTTTGTTTTTTTTTGCAGAGAAGATTATACCTTAAATTTGAATTTCTTTAGGAATATTCGAATTCCTATGATTAATACTACTTATTCAACAAATTCGATTGGTTAATACGAGTAGATTTTCGATTACGATAAATTGATGAAACAATAGCCGGCCCAATGGCTGCTTCAGCGGCTGCAATGGCTATAACAAAATGGAGAAAATATCTCCCTTAATTGACGATTATCAAAAAATCAGAAAACGTTACAAAATTGATATTAACTGCATTCAATATAAGTTCAAGACACATAAGGGCTCTAACCATATTTCGACTTGTGATCAATCCATAGATACCGATAGAAAATAAATAGGCACTCAAAACAAGTACATGTTCGAGCATCATTAAGCAACTCCTTAGCAATCTCATTCATTTCAATCTAAATAACAATTCAATTGGTTTGGTTGAATCACATATAACAAACATGGATGGAATATTTTAATTGCTGATTTTTTATTGACGAGCTACAGCAATTGCACCTATTAAAGCAACTAAAAGGATTATTGAAATGAGTTCAAATGGAAGAAAAAATCCGTTGATAAATGAATTCCAATTTGTTGACTATTGCTTATCAAATCTTGTTCTAGAACCTGGTTTGATCTTGTAGTCCAAATAATCCCGTACCATGACGTATCTGGAATAGTAGTAATTAGTGAAATAAAAAGACTTGTACAAACCACTGAAGTAACCCCATCCCCAACAGTCCAAAGGCGAGAATCTTTGTAATATTCTGAACCACTCATGAACATCACAGCAAAAGAATCAAAACATTTACAGCCCCTACGTAAATAAGTAGTTGCGCAGCTACAAAATAAGAACTCAATAGAATATAGAATAAGGATATACAAACAAGAACCAATCCTAGCGAAAAGGCAGAATAAATTGGATTGGGAAGTAATACCACTCCTAGACCTCCTAAGATCAGACCCGATCCCAGAAAGACTAAAAGAAAATCATGCATTGGCCCAGGTAAATCCATTTAAAAAAAAATCGAAATATTTCATGATTTTATTGACCTGACCAGGAAAAGAAGTAACTCCATTTTTTATGTTTATGATACTTCTTAACTTAAACTTAATAACTTAATAAAATTAAATAAATATTAAATAAATGAAAATTGAATAGGTGCGGGCGCGTTGATGTATATAGTGTTATTGGAGCCCTATCATTCAATATCTGGAAGAAGAGTTTGAAAATATATATTACTCTAATATAAATATAGAAATAGATTTTGAATCCAAATTAAATGACAAGTTTAAACAACTTTTGGATTGATCGAGGAAAGCCTTATTTTCTAGATCCAAACTAAACCTTAATTTCATTTATTTTCATTTTTTTATTATTTAATTTAAATAATTAATTTTTAATTGAATTATTATTATTAATTGGGGATTTTTGAATTGCGAGGTTTAACTATTTTTTATTTGAGGTTAATTCGAAATTGTGCGAATTGTGTAATCATCAATTACTGACGTTGGTAACCGACCCAAAGCAATTTGATTATAATTCAATTCGTGACGATCATAACTCGAAAGTTCGTATTCTTCAGTCATTGATAAACAATTTGTTGGACAATACTCAACGCAATTACCACAAAATATACAGATTCCAAAATCAATACTGTAATTAAACAATCGTTTCTTTCGAATATCACTTTCCAATTTCCAATCTACAACGGGTAGATCTATAGGACATACACGAACACATACTTCACAAGCAATGCATTTATCAAATTCAAAGTGGATTCGTCCTCGGAAACGTTCCGACGTGATTAGTTTTTCGTAGGGATATTGAATAGTTATAGGTAAACGATTCGCGTGAGACAGGGTAATCGTGAAACCTTGACCGATGTATCGGGCAGCTCGTATTGTTTGTTGACCATAATTCGTGAATTCAGTTACCATGGGGAACATATCGTGAATGTGTATAAAGAATCAAATTGTAGGCTTGTTTCTTTCTCTTGTTTGCGATAAGTGATGAATATAGAATATTGTAATTCTTTACAGTGAAAGAAGTTGGGACGAAGTTGTTAATAATAGATTACCTAGAGAAATGGGTAAAAGAAATTTCCATCCAAGATTTAAGAGTTGGTCTATTCTCAACCTTGGTAAAGTCCATCTTGTTGCAATAGGAATGAACAAGAACAAATAAGTTTTAGCTAATGTAATAAAGATGCTGATTATTGTTCCAAAACTTTACCTACTTTATTAAAATTAATATTTATGTCAAAATGTTAGGAACGAATAGGTATGGAATAGAAAGATTCCAACCTCCTAAGTAAAGAACTGTTACAAATAATGAAGAAACTAGTAGATTCAGATATGAAGCAACGTAAAATAAACCAAATTTTATACCTGAATATTCGGTTTGATAACCTGCCACTAATTCTTCTTCTGCTTCTGGTAAATCAAAAGGTAATCTCACACTCGGCTAGAGAAGAAATTAGGAAAATGAGAAACCCTATAGGTTGGCGCCACAAATTCCACCCCCAAAACCGTATTTTGACTGCGCTTCAACTATATCAACTGTACTTGAACTGTTAGATAATCATAGTCGATTAGAACATCGCTATTCTTATCACTATTACAGAACCGTACATGAGATTTTCATCTCATACGGCTCCTCAAAGGTCACAAATAAATCTAAGGACATTTAATTATTATTTATCTTTATCTTGATATTTGATTTTGTAGGATAGAGTCAAAACTTATCCTAGTTCCCCAAATTAGACCAACGGAATTCTGTTTGCTATATTTTATATTAAAATGAAATATATATATTAATATTATAATTATAAAAATTATAAAATAAAAAGGGTGCTTCTGAATTAATCTCATCTTTTAATTTTAGGAATGTTATTTTTGCTTGTTGATCAATAACTTAACTTAATCCTTGAATAAAAACTTTTGTAACAACATCATAGAGGTATTTCAACCTATTATTTTCAATTACGAAAAAGAATTCGACGTTCCATTAATTTATGAATCATGCTAAGAGTTCTCTTTCTAACTAACGAAAAGATATAGGAAAGGGAGATCTTTTTTTTTTTTATTTTATTGTTATTCTATTTTATTTCGTTCCTATTCTCCTTTCTCATAAAGGGATATTAATCAAAATAAAAGATTACTTCGTTCTTGATAGTTATTTACTTAATCGGTGGATAGGGGCATACTCTAGGCCGGAATCGTGGGTAGTACTTCTTGATCATTTCTACTAACTTAAAGTCCCAATTCGAATTCCATTTATGTACAGAAATACCCTCTTGAATAATTTAGAGAATATCCATTACTAATCCTTTGTGTATTTTGGTCTTTCTAACCATCCACTCAATTTTGTTCAACCTCCCGTTTAAACCCGCTTCAAGTGATGATAACTAAGCAACCAATCTTGGGGTAAGCGGTCTCTACTGCTTATATTTACTTTTAATTAATAATTAATTCTTGTACATAGGAAATGAGACTTAATCTTTTACTGCAAATTTGTAAGCCGTTTTCTTTCACTCATATAACTATCTGCTTTAGTTCATCAACCCAAATGATGACTAAAAAGATGAAAAACATTTATTTAACCCTCTTCCCAGAAATTCGAAAGAAAAGAACTAGGAACTTTTTGTATTTCACCTAATTAGACGTCCCCGAATCACACGTAGAGATATTGATAATACACATAAAGTTAATGGTATTTCATAACTAATTGATTGAGCAGCAGCGTAGACCACCTAAAAAGGAATATTTATTATTTGATCCATATCCCGACATAAGAAGTCCAACAGGAGCAATGCTTGAAATAGCGATCCATAAAAAACACCAATACCAAGATCAGATAGAATAAGGTGGTATCCAAAAGGAATTACTGAATAACTTAGTAAAATCGATATGACTGCGACGGATGGTCCGATACTAAATAAACGACCATCTCCTCTAGATGGAAGAAGGTTCTCTTTGAAAAGTAGTTTTGTACCATCTGCTAGAGCTTGAAGAATTCCTAAGGGACCGGCGTATTCAGGCCCAATACGTTGTTGTATTCCTGCAGATATTTCTCTTTCTAACCAAACAATTACGAGTACGCCTATTGTGATTCCTAATACAAGAGTCAAAATCGGAACAAGTACCCATATAATCCCATAGACCTCTTTTAAGGATTCTAATCTGGAAAAAGAATTGATAGCTTGTATTTCGGTTGTATCAATTATCATTTTAACGATCAACTTCTCCCATAATGATATCTATGCTACCTAATATCGTCATAATATCAGCCAATTTCATTCTTTTAACTAACTGAGGAAGAATTTGCAAATTGATAAAACCCGGTGGACGAATTTTCCATCTCCAAGGAAAAACACTCTGATCCCCTATCAGAAAAATTCCCAATTCCTTTTGGGGCTTCAACTCTCACATAAAGTTCTTGTTTCGCCAATTCAAAGGTTGGAGAGGGTTTTTACTAATAAATCGATATTCAAAATTATTCCATTCGGAATCTTTTACTCTATCAAAACGTCGTATTTCTAAATTCTCGTAGGGCCCTCCTGGAATTCCTTCCAGAGCCTGTTGTATAATTTTTATGGATTCTGTCATTTCGCCGATTCGTACTAAATAACGAGCTAATGAATCCCCTTCTTTTGCCATTGAACTTCCCAATCAAATTCATCGTAACACTCATAATGATCAACTTTACGAAGATCCCATTGGATTCCGGAAGCCCGTAGCATTGGTCCCGATAAACCCCAATTTAGTGCTTCTTCTCCGCGAATAATGCCCACGCCTTCAACTCGTTCTAAAAAATTGGATTCCGTGTAATAAGCTTTTTATATTCAGCAACCCCAGTTAAAAAGTAATCACAAAAATCCAAACATTTATCTATCCAGCCATACGGTAGATCAGCAGCTACTCCTCCGATACGAAAATAGTTATGCATCATTCGCATACCAGTAGCAGCTTCGAATAGGTCATATATCAATTCCCTTTCTCGAAAAATATAGAAGAAAGGGGTCTGTGCACCAATATCTGCCATAAAGGGCCAAGCCATAACAAATGGGAAGCTATACGACTCAACTCCAACATAATGACTCTGATATAACTAGCTCTTTGGGGTACTTGAATATTTCCTAATTGTTCGGGCCCGTTTACAGTTATTGCTTCTGTGAACATAGTAGCTAAATAATCCCAACGTGTTACATAGGGCAAATATTGTATAATTGTTCGATTTTCTGCAATTTTTCCATCCCCTGTGTAAATAACCTAATATAGGTTCACAGTCAATAACATCTTCACCATCTAGAGTAAGAATGAGTCGAAGAACACCATGCATTGATGGGTGCTGAGGCCCCATATTGACTATCATAAGGTCTTTTCTTGTAGCTGGTACAGTCATAAGTTTTTACCCATTCATTCTTCCATGAATTCCTGAAAGTGAAAAGAAGTTTATCCAAATACCAAATAAAAAATAAAAGAATACCTAAAACGATTCTTCCAATTAACGAGTTTTTGTCTCTCGAATACTCAACTGACCAATTAATTCTTTATAACGTACTCTATTTTTTGCCAAATAAGCCAACAGCCGTTGACGTTTTCCTAAAATTTTTCGCAAACCTCTCTGAGATAAATAGTCTTTTTGTGCACTTCCAAATGTGAAGTAAGTCTCCGTATCTTATTGGTAAAACTGAATACTTGAAATTCAACCGACCCCTTCTTTCTTTTTCAGAAATAACTGAAATGAATGCATTTTTACCATAAAAGATTTTCCTCTTCCACCTTTACAGATATGGATTTTACTGATGAGTAATAATAATGCTAGTAATTTTAATGTGTTATATACAATAATCTGGATTTCTTGATAATTTGAATTTCTTTATGAAATCCTAATTTTATCAACTCATATTAATCCATCCAGGTTTCAATTTCATTTTATTCAGAAAAGAAAGAAGGGGGTTCGTTTTGCATGGCATAATTTAGACCTAAAATGAAGAAGATTCTGTTAATTGATTTGTAGGTCTAATTGATACCTAAGCGGCTTTAGTCTTCGTATCATATATTAGAATGGCATGGAAGACGGTGCGTGTGAATCTCTTTACTTTCATATACCCCGTAGGGTATAGCATATATAGGAAAAATGGACTATCAACGACTTTTCAACCGGGGATACGGATGTATCCTTAACATACTGAAACGACTGCCGTTATTTGTATCAAACCAATAGCGATTCATACAAGCTAAATCTTCTAATCGATAATTAGGCCAAAGAAAAATTTTAATTGAATTAATTCATTTTTTCTTTATCAGGATGGTTCCCTTTATCCAAAGATTGACTGCAGTTGTTCTCAGTACAAAATATTGGATTTTTCTTCCTATTCTTTGAATTGAAAGAAATTAGAATTCTCAACTCTCTACGATGTCTAGGCGATAAAATGGTTTCGGGAACAAGCAAATCAAAACCATTCTTATCAACATGGGGTTGTTCTCTATATCCTTGATTTGTTTGGTTCTTACTCTTATGAACCAACGAAATACCTAAGGTTTGATACATAATAAATTGTCCATCATTTTTACAGACAGACGCGTGGGTTCGATAATCAGGACCCCCTTTTGATCAATTCTGCAAGACTTAAATTCTTCTGAATCAGCATTATATCCAAACTAATTTCTCTTCTTTGAATCGAGGATATAGTAATTTTTCGTGGATTTATCAGCCTAAGCAGGAGACAGTATATTTTTATATTATTGATCATTCTTTCATTCAAAACATCGCCCCATCTCAATTGAAAAGTAAATAGCGTTTTAGGAATAAATCTAGTTCTGCTCCTGTATTACTTTTATTTTTTTCTTTCTTGCATAAGGATCTTCAATATCTTTTCGAGGTTTGTTGAAGGACCAGATTCAAGATTCCCTTGTTTTTGTACATTTGATCTAAGATCTCTTTTCCATTCTTTTGTTGATTCTTTATTTTTTATTTGAAACACATTCCCTTTTGGTTTCCTTCGATGTTTTTCTTTTCACTAAGAGCTTTTTCGTTTAGATTCAAATTAAAAGAAGGACTTCACTTGGTATAACCCACGGTTTTATTTTATATAGATTATAAGGTAGAACAAATTCGGGGAAGAACCAAAGTCCTAGGGTTGAGATGGGACGATTTAGCATTTCTTCATTCATTCCCATCCAAAAAACCTTTTGGGAATTTGGTAAATTGATTTGGAGCTTTGGCGTAAGCGTAAGATAAACAAGGCCTTTATTATCAATTTTATCAATTATTTGATAATTGTTAGTATCAATCTTAGTATTTTGATTACTCGTGGTATCGATTTTGATACAGGACTCAATAGTGACTTTTTGTCTAAGATCAAAATGGAGAATTTTCCAATCAAAATATTTTCTATTGGGATTTTTTCCATATATCTAATATCGGCATAATTATTAATAGGATACCCTCCGTATATCAAAAAATTCTGTTTATGTGTGTTGGAATTATAAGAATTTTTTCGTTCTTATTTAATTGTACTTGAAAGCTTGATTTAGAAATAGATGAGTCCCTCTTATTTTCATAATTCAAATTAATACATTTATACGATAAAAGATCATATCTAGAGTTTTTTGAAAATTCTCTTTTTATTCAATAAGTAATATATTTCCGAATTCCTAGAATTCCCTTTTTTGACTGAATTTTTCATATGAATCCCAAGTGGGATTTTTATTTTTATGACGTAGATTCACTCTATTTCTCCACTTTTGTGGTATTAATCCAGACCATTTAATAGGAGATAAATCGTATTGATAATGCCCTTTAACCAGTTTTTCCATTCATTCATTTCAGAATTCGTAAGTTTCTTATGACTTAATTTAGAATGAAGTATTCCTTGTGTTTCAAAGGAATCCATTATTTCAGCTTTAATAAAAAAGACATTCCGTGATATTGAAAAACAGATCTTAATTTATACAAGTTACTAACTTGGATTTGTGATAATTTGTAAAATACATATGCTTGTGACAAATATGATAAATCACAAAAACTATGTAAATTTTTTCTATTTCTAATACTATTAATTGATCTTTTAGAGTTGAAATAGTTGAAATAAAATGAATTGTATTTTTATTTTCTCTATTAATAAGCCTTTCTTGATTTGCTTCATTAATGGGTTTATCCGTCATTTTTTTATCGATTCAAGAAGAAATTGTGTATTGAGTCTGGGAATATTAACAATAGATAAAAATATATCTATGTATATTCTTTCGAGGAAAATTTTTATAAAGCAATCTAATTGAGAGATTAATCGGACATTTCTTCTTTTAATAGTTGCCAAATATTTGTTGTCGATTCGAACCTTTTGGCATTATAACTTTTTCAGTAGGACTAATATATACTCCTGATGGGGTTATTTTTGTTTTTCTTTTGTAATTCTTTCTATTTGATTTCGAATTGTGCTTGTTCTACCAGTTAGATCCTTCTTTTTGTCAGTGAAGAATTTGTCCAATTTTGAGATTGAAGTAGACTAAATGATTCATGAATTATTTGATTGCTGATTCTAGATTCTTTTCGTTTTTAATCTCATTCGATTCAGATACTTTTCTTAAGCTAAATAAGATAATTGGGTTGAATTTAAAAGTCCTTTTTTATTATTTTTCTAAATAAAAACTTTCGATAATGGATTTTTGTTTCTTTTGAAACTAATTTTGTTTTTCCTTTTAAAACTCTTAGAGCTAATATCTTAAATTTGCCAATTTTTGTTTGTAGTTCCTTAAAATGGGCTTAAAAAGGAATATCGTTTTCTGGGAGAACCAAAAGGAAGTTCAGTTTCCATTCCCCAAACTGTTAAAAACAAAATCATCTTTTGTTTTTCTTTTTGATTAGATTTCTATCAGAGTGTCGTAATTTAGATTTGTGCCAAGGTTTCAGGTAGAAAGGAAATAGGATTTTTATCTGAATACCATCTGTCAACCAATTTTTCGGAAATTCTTTTCCGATAATTGGACACCATTATAGGTACATTTAACATGCATTTCTTGATTCAATTCCTGTATATCCTCAGACCATTCGGGAAATTGCAATAATAACATACGTCCAATATTTTTGGTTATTATCAATGAAGGCAATACAATATATTTTCTAAGAATCGATTGAGTTATTAACATAAATCCCTTATTACTTGCGCCAATGGAATGTTATCCCATGCCTCCGCTATCTCTATCCGCGTTTGCTCCTTTCTTATACTGTCCTCTTTTTGTCTTCTTTTTTGTTTCTTCGTCTATATACTCCACAATTTCGGATTCTACCCCTTTCCTTTCCACTTTCTAAAAATCACTTTAACGATTTCTGATATATCAAACGAGAAGTGGTGGGTGCTTTTGATTTTATCCAAAAAAGGGAGAGCGTGCGTTTGCTTGAAACAGTTCCCAAATTACAATTTTACGCCTTTGAGCGCGCATAGAACCTTTAATTATTCCTCGCCGAAAGTCCGATTGTTGTGAATAGCGTAGCAAAGCCACTTCGTTTCTCGGATCTGTAGGATTACTACCACCAGTATTAGAGTCAGTATTCTCGTTGTTAGGAGTAAAATCACTACATGTTTGGCTTTTCTTGATCGAATTTGATGGTCGATTGGCACGTTTTCTTGATATTCTCCTAATTGTTGTTCTAACTCGGTGATTAATTTGTATGGCCATCGAGGGACTTTTTACTGATTTCTTTTATTCCAATAGATTCTTTAATAGATTCCTTTGGAATTTTTGACTATTAGTATGGGTTTTAATGACATTCAATAAAAATTGGAAAAATCTTTCCTTTTTTTATTCAATTTTAGTTGGCTATCAAATTTGCCAGTTAAAGGTAAAAATTTTCCTGTTCTATTAAAAATTGTTTTTATCAAATGTATCCGTTTTCCGCTCAAATTCTTGGTAATCGGGATCTGGAAAAGGATAGCGTAAATCCGATTTATACCAAAGGTTTCTCTTAAATTTTCTATCGACGTTTTATAAGGCGAAACCTTTTTTCATGGTTAAGCGATAGGCCCCATTTAAGAAAGGATCATAGGCTTTAGGAAAATATTCGTTTTACTATCATCATTACACAATCGAGTTCTTGTTTCTAGTATATCTATAAAAAGCGATTTCTTGTCTAGAGCTTCAATTCTATTTCTAAATTCCTTGCTTGTCTTATTCCTTTTCTGTTGTTGGAATAAATCCAACGGTTGGCAAATTCGTTATCATTAGAGGGGTTTTATCTATCGTAGACCACGATACCTTTCTTTTAGTATTTCCTCAAAATTGATAAACTTGGTGGATAGGTAAAAGAAATTCTTTGTTTTCCATCACTTTGGCATGTGTCAAAGAAATATTGTGACATTTCATTTCGTACAGCCCTTTCAAATTGATTATTCTTTATGTAGCGAAGTGGACGATTCCATCGGTTATAATCGAAAATAAGAGTCACAAGAGATTTTTCAAGGCAGAAGAGTTTCCCTTTTTATTCGAATTTTCTTGATTCGGATTCCTATTTAGATAAGAATCCTCATAAACGGCGCTTTTGTTATAGCCTGTCTCTGGAAAGTGAAGGTCGAATTCATCCTTTGTTTTTTCCTTTCCATTCACTCGGATTTCTTCCGTTTCATCGATTTTGTCCGGATCCTCCCTTTCTTCCGAAAAAGGGAAGAAGAAGGATCTTCTTCGGTGGATCCTCTTGTTCCTGTTTAATCCCCTTCATTTCGGAAGTTGTTTCTATTTCTACATCTCTTTCTTCCTCACTTTCCACCCTTTCTTCCGTTTTTGAGGTTTCTTTCAGTTTCTTAGTAAGAATGGGTGACGGTATTCTGCCTAAATAGTAGACACAGGTAATAAATAAGAGAATACTAAAGATCCGAGCCATAGAATTTCTCAATTCTGACACAAGGTACTTATTAGATCGAATGTACTTATTCGATCTAATAGAATGATTTTGCCGTATCCAGACTAATACCAATCCAAGCCATTTCATGAATAAATGTGACCAATTAACCAACCAACAAAACTACTTGTTACAAATAACATCTTGTTGTTGCATCGAAACATATAAATGTTGACTAATCTGGCTAACATTGAACTTGGTAAAAGGAAATAGTTGAATAATTGAAAAATGAGATTATTCAGGAATACACATTGAATGCTGAGATTACGCATTAAGTTTCGGGTAGTAGATCCATAATCAAAAAGTGTTTGTGATTGTTCCAGAAGAAATGAAACAAAAGATATGGTAGAGCTAGGACAGTTATTGTATGAGGTCTACCCAATGCTAGATGCAGAGGCGCATAATAGATCGATATGAACATCATGAGCTGTCCTGTAATAAAACCAGTTGTTGCTGATACCTTCTTCTCGGTTCCTTCTTCCATAACCTGAGCTCGGAGAAGGAAGAGATAAGAGGGCCCTATGGAGAATGTGGTCAGAAATCCATAATAGAGTCCGACCACAACGACCGAATTGATTATCTTCATGCATAAGGATACTAGATTACCTAGTAGAAAAGATTGAAAAATCATCACAAACCTCCCTATTTCTTTTCTATTGCAATTTCTGGATTATTATATGATGATTTTGAACTTTCCATATATAGAAAGAAATAGAAAGAGATAGACTAGAAACGACATCTGTTATGTCAATGACACCAAAGGGATATTAAATGAATGGAATTGGGATATGGATGGAATATAATGAAATAGAGCCACTTTGAGGTTCCCTCTGAAATGAGGCATGGAGGGAGCCACTACGAAGAAGTTCCGGGGTTACGAAGGAAGCTTCTAGCTCATATTGGTCATGGGTTGAGAACAGGAATTGAACTCTATGAGATCTAATCTCCGTTGTTCCTCAGTAGCTCAGTGGTAGAGCGGTCGGCTGTTAACCGATTGGTCGTAGGTTCGAATCCTACTTGGGGAGATTTGATTCATTCTGAATTAAAGAATTCAGAATAAAGGGGCTCGCTTTGACCGTTAAGAGTAGGTAACCCGTTCCCTGTCTTTGCATTCTATCCCATCGTATCACATTCTGCGAGATTTGAAAATCACCGTCAATACCTCGGTGTAGGTCTGGGATAATCCTTTGTTCCATAGCCCTGGGGCTATTTCCAACTAGCCAATTAAGAATTAAAGAATTACTAGTACTAGCACTGCATCAAAGATGCAGTCATCGATTCTCCGAGAGGTCACAATTGCCGCAAGATATTAATGACGGGAGGGCTTTTTGTTATGCTACTAATACTTGTACTTTCTCTGCTATTCTGCCCAAGCCTGGCTGAGGAAGATTTACGGGCGTAAAACAAAAAATACGCGGACAGGGCATACTATGTGTAATGATTCCACCATCTACAATAAATCCACAATAAATAGATAAAAAAAAAGCCATTCCATTTCGACAAAAGACCCACACCCAAGTTCCATAGCTTTTTTGGTCCGCTATCCCCATCATTCTACTCCCAGAGAAAGGTACTTCCCTTTGGGCCGGTTGTGGGCGAGGAGGATTCGAACCCCCGACACCGTGGTTCGTAGCCACGTGCTCTAATCCTCTGAGCTACAGGCCCCACCTCGTCTCCAGCGGATCTGTTCCCGGAGTACCCCCAAAAAGGACTCTTTCCTCTCCCCAGCCATTTCAATTTCGGGTTAAGAAGATGTGAAGGCGCGTTTATCTCTATAAGAGGTGCGTTCCGAGGTGTGAAGTGGGAGAGAAGGGATGTCATAATTGGGGTTTTGAATAAGACGACCTTTTGATTTTTCATTTTTCGTTTTCATATTGAAAAGTAATAAGAATGAGAGGTGTTAAGCTTTTTATCATCCTGGCGTCGAGCTATTTTTCCGCAGGACCTCCCTACAGTATCGTCACCGCAGTAGAGTTTAACCACCAAGTTCGGGATGGATTGGTGTGGTTCCTCTACGCCTAGGACACCAGAATATCGAACCATGAACGAAGAAAGGCATGAGAGAAAAGCATGTTGGCTAGTGATTATGAGGCCCCAATTCTTGACTGGAGGGGACACCAAAGCTCTGCTCTCCCTCTGGGATGGAAGGGCAGAGCTTTTGGTTTTTCATGTTGTCAAAGAGTTGAACAATGTTTTTCGTGTTGTCAAAGATTTGAACAATGAAAATAGATGGCGAGTGCCTGATCGAATTGATCAGGTCATGTAGGAACAAGGTTCAAGTCTACCGGTCTGTTAGGATGCCTCAGCTGCATACATCACTGCACTTCCACTTGACACCTATCGTAATGATAAACGGCTCGTCTCGCCGTGACCTTCTCTTGAATTCTCAAAACTTCTGTCGCTCCATCCCGCAGGGCAGAGAACCCGTCGCTATCCCGGCTGTGCTACCGGAGGCTCTGGGGAAGTCGGAATAGGAGAGCACTCATCTTGGGGTGGGCTTACTACTTAGATGCTTTCAGCAGTTATCCGCTCCGCACTTGGCTACCCAGCGTTTACCGTGGGCACGATAACTGGTACACCAGAGGTGCGTCCTTCCCGGTCCTCTCGTACTAGGAAAGGTCCTCTCAATGCTCTAACGCCCACACCGGATATGGACCGAACTGTCTCACGACGTTCTGAACCCAGCTCACGTACCGCTTTAATGGGCGAACAGCCCAACCCTTGGAACATACTACAGCCCCAGGTGGCGAAGAGCCGACATCGAGGTGCCAAACCTTCCCGTCGATGTGAGCTCTTGGGGAAGATCAGCCTGTTATCCCTAGAGTAACTTTTATCCGTTGAGCGACGGCCCTTCCACTCGGCACCGTCGGATCACTAAGGCCGACTTTCGTCCCTGCTCGACGGGTGGGTCTTGCAGTCAAGCTCCCTTCTGCCTTTGCACTCGAGGGCCAATCTCCGTCCGGCCCGAGGAAACCTTTGCACGCCTCCGTTACCTTTTGGGAGGCCTACGCCCCATAGAAACTGTCTACCTGAGACTGTCCCTTGGCCCGTAGGTCCTGACACAAGGTTAGAATTCTAGCTCTTCCAGAGTGGTATCTCACTGATGGCTCAGGCCCCCAGGCCTCTTCTTCCACCTAAGCTGCGCAGGAAAGGCCCAAAGCCAATCCCAGGGAACAGTGAAGCTTCATAGGGTCTTTCTGTCCAGGTGCAGGTAGTCCGCATCTTCACAGACATGTCTATTTCACCGAGCCTCTCTCGAGACAGTGCCCAGATCGTTACGCCTTTCGTGCGGGTCGGAACTTACCCGACAAGGAATTTCGCTACCTTAGGACCGTTATAGTTACGGCCGCCGTTCACCGGGCTTCGGTCGCCGGCTCCCCTGGTCACCAACTTCCTTGACCTTCCGGCACTGGGCAGGCGTCAGCCCCATACATGGTCTTACGACTTTGCGGAGACCTGTGTTTTTGGTAAACAGTCGCCCGGGCCTGGTCACTGCGACCCCCTTTGTGAGGAGGCACCTTCTCCCGAAGTTGGGGCTATTTTGCCGAGTTCCTTAGAGAGAGTTGTCTCGCGCCCCTAGGTATTCTCTACCTACCCACCTGTGTCGGTTTCGGGTACAGGTACCCTTTGCTTACAGTCCTTCGAGCTTTTCCTGGGAGTATGGCATGGGTTACTTCAGCGCCGTAGCGCCTGGTACTCGAACATTGGCTCGAGGCATTTTCTCTACCCCTTCTTACCCTGAAAAGCAGGGGCACCTTACGTTCTTGAACCGATAACCATCTTTCGGCTAACCTAGCCTCCTCCGTCCCTCGGGACCAACAAACGGTAGTACAGGAATATTCACCTGTTGTCCATCGACTACGCCTTTCGGCCTGATCTTAGGCCCTGACTCACCCTCCGTGGACGAACGCGGAGGAACCCTTAGGTTTTCGGGGCATTGGATTCTCACCAATGTTTGCGTTACTCAAGCCGACATTCTCGCTTCCGCTTCGTCCACCACTGCTCGCGCAGGCTTCTCTCTAAGGCGGAACGCTCCCCTACCGATGATGTATTTTTACATCCCACAGCTTCGGCAGATCGCTTAGCCCTGTTCATCTTCGGCGCAAGAGCGCTCGATCAGTGAGCTATTACGCACTCTTTCAAGGGTGGCTGCTTCTAGGCAAACCTCCTGGCTGTCTCTGCACCCCTACCTCCTTTATCACTGAGCGGTCATTTAGGGGCCTTAGCTGGTGATCCGGGCTGTTTCCCTCTCGACGATGAAGCTTATCCCCATCGTCTCACTGGCCGACCTTGACCCCCGTTATTTTGAGGTCATATCTAGTATTCAGAGTTTGCCTCGATTTGGTACCGCTCTCGCGGCCCGCACCGAAACAGTGCTTTACCCTAGATGTCCAGTCAACTGCTGCGCCTCAACGCATTTCGGGGAGAACCAGCTAGCTCTGGGTTCAGTGGCATTTCACCCCTAACCACAACTCATCCGCTGATTCTTCAACATCAGTCGGTTCGGACCTCCACTTAGTTTCACCCAAGCTTCATCCTGGTCATGGATAGATCACCCAGGTTCGGGTCCATAAGCAGTGACAATTGCCCTATGAAGACTCGCTTTCGCTACGGCTCCGGTGGGTTCCCTTAACAA

>Decalepis_hamiltonii_chloroplast_contig2

TCGATATCTTATATCTTATTAGTAAATCTATATCTTATTAGATTCGATAGTAAATATTTTTATTTTCGTTTTATTTTAGATAGTTTTTAGATAGTTAAATTATTTAAATTTGTCATTTTTGAATTTGAATTCAAATGACATTTGAAATTCTTTTTATTACACTTCTATATTTTCTATATTATTTGATTCCATATCATTAGGAATGATTAGTTCGAACTGATGAGACATTCCTCTGCTTTCATTCCATTCATAAAGTAGTAAGGGCGGAAATTAAGACAACAAAAAATCGACCGTTCAAGTATTCAAAATTGCGTGGGAAGGGCGACAGGTAGATATATATATAGGATATCTATCAATCTATATTGAATTACGGATCCAGAAAGGATAAAATCCTATTTGATTGGACCAAATAGAAATAGGGTCTCCTATCGAAGATAGGTAAGAAATCATATAGGAAAATGCTTCTTCAAGATAGGAATCGGTATCTAATGAATTCAAGGGTTCCAGTAGAAAGAAAAGGGGAACGACATCATAACGCAATGAAATCCTAATCTTAACACAAAAGGAAGGGGATATGGCGAAATCGGTAGACGCTACGGACTTAATTGGATTGAGCCTTGGTAAGGAAACCTACTAAGTGATGACTTTCAAATTCAGAGAAACCCCGGAATTAAGAAAAAGGGCAATCCTGAGCCAAATCCTATTTTACACAAACAAAGGTTCAGAAAACGAAAACAAGGATAGGTGCAGAGACTCGACGGAAGATGTTCTAACAAATGGAGTTGGCCGCGTTGGTAGAGAAATCCTTCCATCGAAAATTCAGAAAGGATGAAGGATAAACACATATACATACGTATTGAATACTATATCAAATGATTATGCCGACCCGAATGAATCTGTATTTTTCTATAAAAAACGGAAGAATTGGTGTGAGTCGATTCTTTCTTCAATGTGGAATCGAATATTCATTGATCAAATGATTCACTCCATAGTCTGTAGATCCACTTTTCAAGAACGGATTAATCGGACGAGAATAAAGATAGAGTCCCGTTCTACATGTCAATGCTGGCAACAATGAAATTTATAGTAAGAGGAAAATCCGTCGACTTAAAAAATCGTGAGGGTTCAAGTCCCTCTATCCCCAAAAAGCCTATTTGCCTCCCCAACTATTTATCCATTCTATCCCCCTTTCGCCAGTGTCCTTATACACTCGCCCTATTCTATTCTTTTGAAATTGATCTGGGCGGAAATGTCTTATTATATCTTATATCTAAGATATACATCTTTGATCAAGAAATCCCCATTTGAATGATTTACAATCGATATCATTACTCATACTGAAACTGAAAAAGTCGTTTTTAAGATCCAAGAAATTCCAGTACCTAGATAAAACTTTTTAATCTTCTTTCGCCCTTTTAATTGAGAGACCCCGCCATCTAATAAAATGAGGATGCTACGTTGGGACATAGTCGGGATAGCTCAGCTGGTAGAGCAGAGGACTGAAAATCCTCGTGTCACCAGTTCAAATCTGGTTCCTGGCACATGATTAATTTGGATGAGTTTCTCTTCCATAAATTAATTGATATGAATCGACATCCCTATTCATTTTGAGTTTGGATCATAACACATACTTTTTTTATCCATCTCGCCGAGATATATCCCATCTATTTTATTTTATAGATGGGTAGAATTCTTTAGATACTTTATCTAAAAGAATTCGATTCCATTTCATCTTTTTATCTTTATGTCCAGCCCGTAAGTCAAAAGAATGATAATACTTCATATATATTCAAAAGACGCGTTCAAAAGGTTAAATTAGTTGGTTGAGATACTCAAAAGTCGAATCTAGAGAAATTGAAATAGACAAGATTCTGTTCAGATATAAATAAATAGAATCTGGTTCGATTTGTTTATTTCTACCTACATAACTTTCTAGAACCATCTAAGTAATATGCGTGGTACAAAGTCAAACTTCATGATACAGAACTCCTTTGGTTCATCCTATTGGTTTGGCTCAGCTGAAATAGATATCTTACAACCCAAATAAATGGGAGGCGAGAATTCCAACGAATCCCTAATTTTTTTTGTTGTTATTGTTAGCCTATCGAGAATTTAAACAAGACTTCCATTATTCTTGTTAATCTAGAAGAGAACGTACAAGCTTTGAATATCTGAAATTGTATAAGTGGAATTTTTTTCTTATCATTCAATGAGCATCTTGTATTTCATAAAAATTGGGGCAATATAATCCTTACGTAAGGGCCATCCTATCCAACTTTCGGGCATTAAGATACGTTTCAAGCGTGGATGATTGTCATAAGAGATTCCCAACATATCATAAGATTCTCGTTCTTGAAAATCTACACTTTTCCAAACCCAGAAGACGGATGGAATCCTAGGATTCATCCTTGAGGCAAATACTTTTATGCATACCTCTTCCGGTTGATCCACACCATACTCTATTCTCGTAAGATGATACACACTAGCTAACAGCCCTCCGGGTGCTACATCATAGGCGCATTGGGAGCGTAGATAATTGTAACCATATACATATAAAATGACAGCAATGGAATGCCAATCCTCGGGCTTTACTTGTAAAGTTTCTATTCCTTGGTAATCAAAGCCTAAAGATCTATGAATTAGCCCACGCTTGGCTAGCCAAGCAGACAAACGACCCTGCATCTTTTTATCTCTCCCGCATTTTTTGTATAAGTTTTTCATATTTACGATAAAATTTATGAAGATTGTCCTACTCCTTGTTAAAGAAATCCTGTCTAAGTGACTAATTCATGGGAAGATACTGAACTTTTATATTTGAAAAAGTTTCAGTAGGTATCTCTGAAGTAGAAGTCGATGGCGGTTGATAAAGGAATCCTTGATCATAATTTCCAGTATGAATACTGCGTCCAACGCGAAACTTGTGATTGGTAGTAAAACACCGATTCGCTTGTTGAGACCTAATTCTATCTTCATAGATTTCTCGAGATATTTTTTACGAAGTTTTGTTATAGCATCTATAACTGCTTCCGGTTTAGGTGGGCAGCCCGGCAGATAGACATCCACAGGAATTAGCTTATCAACTCCCCGAACAGTACTATAAGAATCAGTACTGAACATACCTCCGGTAATTGTACATGCTCCCATAGCAATAACATATTTTGGTTCAGGCATTTGCTCATATAATCTTACTAGGAAGGGGCCATTTTCATTGTTACTGTTCCGGCTGTTAAAATTAGATCTGCCTGTCTAGGACTCGATCTTGGGACTAGTCCATAACGATCAAAGTCGAATCGTGACCCTATTAGGGAAGCAAATTCAATGAAGCAACAACTGGTACCATAGAGAAGCGGCCATAAACTAGAGAGTCTTGACCAATTTGAAAGATCATTTAATGTAGTTGAAATAACTGAATTTTGGATCGTTCGATTAAGTAAAGGAAACTCAATAGAATTCATAACTGTCTCAATCTTATTTTTCTTTGTATTGTCCGAATATTCAGGGGTTAAGACCATTCCAATGCCCCTTTCGCCATGCATAAACTAAACCAACAATTAAGATAAGCACAAAATTAAAGCTTCTATAAATACGGATACACCCAATACATCGAAACTCATTGCCCAGGGATAAAGAAAACCGTTTCAACATCAAAACAACAAAAACTAGAGCAAACATATAATAACGGATTCGAAATTGTAACCAAGCATCGCCCATTGGTTCTATACCCGATTCATAGCTAGAAAGTTTCTCTGGTCCTTTGTTAATCGGGGCTAAAACTCCGGAAATAAAAAAGCCAAAATAGGAATAAGACTTGATATTATTAGAAATGCCCAAAAATATCATATTCGTAAAGCAGAAACATAGACGCACTCCCATGAACGTGGAAAATCTACCGGATTGCTCGATTCGAATTGTCAAGTCATCCATAACTGTTTAGTCAAAATCAAAACAAGAATTCATTTTGATCGAATTGACTAGTTTCTTTACTTATCTCATTTCAAGACGAAATCGTATTTCCATGTGAATTATATTTAATTCGATTTTATAACTTTAGATCTATATCTTACTCTTATACAAATATACAAATTCTCTTATTTTCACCTATAATTCTCTTTAAATAAAGGGAATTCAAAATAAATATGGAATTCCCTTAGAATTTTGAACTTCTTTTTTTATTAATTAAATTAATTAAAATGAATTTTAATTATATATTTATTTATATTTTTGAAATTGAATTGATAATTTATTTTTATTTATTGAAATTCAATTCTTTTCTTTATAGATTTCTATTTTATGAATAGAATCGGCAGGTCCTTTCTAGATCCATTGAGGTTTTTAACAGGGTGGCTCGTGTCACGATTTTGACTTCAAAAATTCACCAACATTGTGTATACTAAAGAAAGGGAGTATCAAGAACTCCTAGAATATGAAACTCGCCCCTAAGATTAATGACAAAAGGTTGCTTTGTTTATCCGCGATTGGAAAAATATCAATCGGATCCATTGAAACGCCTTTTTTTGATTTCAGGTTTATGTTCTATTTTAAAAGCTTGCCGTGAGTAAGCTTATGAAAAAAATTTTGATTTCGATTAACCAACCCGACAGTTCCAAGCAACAAACAATAATGAAAAAATTATACAATTGAATTTTCATTAATCATTTATATTTATTCTACTTTTTATTTTTATATTATATTATAGTATAGTTATATTATATTATAGTATTAGGGCTATACGGACTCGAACCGTAGACCTTCTCGGTAAAACAGATCAAACTTTTTATTATCAAAATGATCTGAACTGTTTCAAAGACCCAACATGCATTTTTTTGCATTGGGCTCTTTCATTAACTGATCAAAATATCAGCTAGTCTGCCATATTTTTTCTTGATATAAAAAGATAAGTAGATGGCTCCATGTGCTCTGATTCATTATTTGGGATTCTGATTCAAGAGCACTACCAAAGTGTTTCAAGGGTAGGGTTATCTTGACGTAGGTCTGCCTCTGGCCTAGATCAACCTAAGTTAAATGAAGTCTCTATCGTTCTGATTACAAAATGAAATTATGAAACTTCATACACCTTAAAGTTCATAGGATGAAAAGAGATTTTTGAGGTCCTTAGACTCATTATGCCTAGCGTTGAATAGACTGGATATTCACCTTATCAATATCTCAAATCCATGATAGGGTCTATTTGGCACCTAAATAGGCACCTCAATCGGACCGAATCCTTTGCCAGGCTATTGTTCCCCAAAGTTATGGGGTAAGGCATCGATTAAGATCACAATTTTGTGATTGGATTGTATGATGGATCCCTGAAAAACATTGGCGCGTGTAAACGAGGTGCTCTACCAACTGAGCTATAGCCCTTATTGCTTGTGATACATATTTTATCATGTAGAGAATTTCTTGTCAAGATAAATTAACATCATAAATGTTTGATCTGTTTGAATGATATTGCTTAAATTAATATTGCTTAAGATTAGTATTGCTTATTAAGATTAGTATTGCTTATAAGTAATATGATATTTATAATCCATCCACGGGGTGAGTACCGTTTGGTTCTCTTTGAGATGAGAAATGACCTACTTAACTCAGCGGTTAGAGTATTGCTTTCATACGGCGAGAGTCATTGGTTCAAATCAATAGTAGGTAGAACTTATTAGATACCGTCGACTCCAGTATCTAATAAGTTTTTGCCCCACCCTTTTTATTTTATTGATTTTGTTTGCTATTTCATTTTTTTTCAATTGGATTTTGCTTGATTGTATTTAATTCTATTCACTCGACAGAATCCAATCAAAATGGGGTTCGAAACAGAACTTCTTTTGATTATGTGAACGTACCAACTAGTTATGTTATGAAATCAAACCGCATTGATAGCCTCTACCCGTGTCCTAGCTCTGCGGAGAGCTAGATTTGCCTCAATTGTTTGTCTCTTTCCTTCCGCTTTTCTCAAATTAGCTTCCGCGATTTCAAGAGTTTGCCGGGCTTCTTGTGGATCAATGTCACTACCCTTCTCGGCATCATTTACTAAAACAGTGATCTCATTATTGCCTATTCTAGCAAAACCACCCATCAGAGCCATCGTTAACCATTGGTCGTTAAGGCGTATTTTCAAAATCCCTATATCTACGGCTGTGGCAATAGGGGCATGGTCGGGTAATACGCCAATTTGACCATTATTAGTAGATAAAATAATTTCTTTTACTTCTGAATCCCAAACAATTCGATTAGGGGTTAGTACACAAAGATGTAAGGTCATTTCTTCTCCATTTCTAAGTTCATAGCCTTTGCGGTAGCTTCATCGATATTACCTACCAAATAAAAGGCCTGTTCAGGAAGACCATCTAATTCTCCGGAAAGGATCAATTGAAATCCTCTAATTGTTTCTGCTAGACCAACATATTTCCCCGGAGAACCGGTAAATACTTCTGCTACGAAAAGGGTTGTGATAAGAAACGCTCAATTTTTCGCGCCCGCGCTACGGTTAAACGATCTTCTTCGGATAATTCGTCTAACCCAAGGATAGCTATAATATCCTGAAGTTCTTTGTAACGTTGTAAAGTTTGCTTAACTCTTTGCGCAGTTTCATAATGTTCCTCACCAACGATCCCAGGTTGAAGCATGGTTGATGTTGAATCTAAAGGATCTACTGCTGGGTAGATACCTTTGGCAGCTAATCCTCTCGATAGTACGGTAGTAGCATCTAAATGTGCAAATGTCGTAGCAGGAGCGGGGTCGGTTAAATCGTCTGCAGGTACATAAACTGCTTGAATAGAAGTTATGGACCCCTCTTTGGTAGAAGTAATTCTTTCTTGTAAAGAACCCATTTCGGTACTCAAGGTGGGCTGATAGCCCACAGCGGAAGGCATTCTGCCCAATAAGGCCGAGACTTCAGATCCTGCTTGAACGAAACGGAAAATATTGTCGATAAATAAAAGTACGTCTTGTTCATTAACATCTCGGAAATATTCCGCCATAGTTAGGGCAGTCAAGCCAACTCTCATACGAGCTCCCGGCGGTTCATTCATCTGGCCGTAAACCAGAGCCACTTTTGATTCTGCAATATTTTCTTCATTAATCACTCCCGATTCCTTCATTTCCATGTAAAGATCATTTCCTTCACGAGTACGTTCACCCACCCCAAATACGGATACGCCCCCATGAGCTTTGGCAATATTGTTAATCAATTCCATAATGAGTACTGTTTTACCCACCCCAGCTCCTCCGAAGAGTCCTATTTTTCCTCCACGGCGATAAGGGGCTAAAAGATCTACTACTTTAATTCCCGTTTCAAAAATAGATAATTTTGTATCTAACTGTACAAAGGCGGGCGCAGATCTATGAATAGGAGATGTTGTACGAGCATCTACAGGCCCTAAATTATCAACAGGCTCCCCAAGCACGTTGAAAATTCGTCCCAGAGTCGCCCCCTACCGGAACGCTTAAAGCGGCTCCCGTGTCAATCACTTCCATTCCTCTCGTTAGACCATCTGTAGCACTCATAGCTACAGCCCGAACTCGATTATTCCCTAATAATTGCTGTACCTCACAAGTCACGTTAATTGGTTGACCAAGAGTATCTCGACCTTTAACTACCAGAGCGTTATAAATATTAGGCATCTTACCCGGCGAAAAGGCTACATCTAGTACCGGACCGATGATTTGGACGATACGCCCCAGGTTGTTTTTTCAAGCGTGGAAACCCCAGAACCAGAAGTAGTAGGATTGATTCTCATAATAATAAAGAAAATATGTCAAATTTTGTTGCGAAAATTATCGAATTCAAAACAAATATCCGATAGCACGTCGATCGGTTAATTCAATAAGAAATAAGAAATGGGAATTAGCACTCCATTTCGTTGGTACCATTCAATCGAATCCAATCCACTCCTTTCCTTATTCAAAGAGTAGATTTGAAAATCCAACCAACCTATTTTTACAATATCAAGTGGATGAATAAGAATCTTGAGAAAGTCTTTCATTTGTCTATAATGATAGACAATCCCATTTATATTATTTAATATATGGAATTCGAACCTGAACTCTATTTCCATTACGATTCATTATTTCTAGTCCACTGGCTTTTTTGTATTTCGGCATATCGATTTACGCCTAGCCTATTTCTTTTTTTTTCGTTTTATACCTTTTCATAGACGAATTCCGTACATTTCCACATCTAGGATTTACATATACAACATATACCGCTGTCAAGGGGAATTTCTTATTAGTTAGTTCTTTCTATTCCAAAAGGGTAAAAAAGAAAAATTGGGTTGCGCTATATATATGAAAGAGTATACAATAATGATGTATTTGGCAAATCGCATGGTCTAATAATCAAATATTCAGATTAGTTGATAATATTAGTATTAGTTGGGAATTTTGTGAAAGATTTCTATGAAAGGGTTCATTAACATGTCGAGTAGACCTTGTTGTTGTGAGAATTCTTAATTCATGAGTTGTAGGGAGGGATTTATGTCACCACAAACAGAGACTAAAGCAAGTGTTGGATTCAAAGCCGGTGTTAAAGAGTACAAATTGACTTATTATACTCCTGAATACGAAACTAAAGATACTGATATCTTGGCAGCATTCCGAGTAACTCCTCAACCCGGAGTTCCACCCGAAGAAGCAGGGGCCGCGGTAGCTGCCGAATCTTCTACTGGTACATGGACAACTGTGTGGACCGATGGACTTACCAGCCTTGATCGTTACAAAGGGCGATGCTACCACATTGAGCCCGTTCCTGGAGAAGAAGATCAATATATTGCTTATGTAGCTTACCCTTAGACCTTTTGAAGAAGGTTCTGTTACTAACATGCTTACTTCCATTGTAGGTAATGTATTTGGGTTCAAAGCCCTACGCGCTCTACGTCTGGAAGATTTGCGAATCCCTACGGCTTATATTAAAACCTTTCAAGGCCCGCCTCATGGCATCCAGGTTGAGAGAGATAAATTGAACAAATATGGTCGTCCCCTGTTGGGATGTACTATTAAACCAAAATTGGGGTTATCAGCTAAAAACTACGGTAGGGCGGTTTATGAATGTCTTCGTGGTGGACTTGATTTTACCAAAGATGATGAAAACGTGAACTCCCAACCGTTTATGCGTTGGAGAGATCGTTTCTTGTTTTGTGCCGAAGCAATTTTTAAAGCACAGGCTGAAACCGGCGAAATCAAGGGGCATTACTTGAATGCTACTGCAGGTACATGCGAAGAAATGTACAAAAGAGCTATATTTGCTAGAGAATTGGGAGCTCCTATCGTAATGCATGACTACTTAACAGGGGATTCACTGCAAATACGAGCTTGGCTCATTATTGCCGAGATAATGGCCTACTTCTTCACATCCACCGTGCAATGCACGCAGTTATTGATAGACAGAAGAATCATGGTATGCACTTCCGCGTACTAGCTAAAGCGTTACGTATGTCTGGTGGAGATCATATTCACGCGGGTACCGTAGTAGGTAAACTTGAAGGGAAAGAGACATCACTTTGGGCTTTGTTGATTTACTGCGTGATGATTTTATTGAAAAGATCGAAGTCGCGGTATTTATTTCACTCAAGATTGGGTCTCTCTACCTGGTGTTCTGCCGGTGGCTTCAGGGGTATTCACGTTTGGCATATGCCCGCTCTGACCGAGATCTTTGGTGATGATGCTGTACTACAGTTCGGTGGAGGAACTTTAGGACACCCTTGGGGTAATGCGCCAGGTGCCGTAGCGAATCGAGTAGCTCTAGAAGCATGTGTACAAGCTCGTAATGAAGGACGCGATCTTGCTACTGAGGGTAATGAAATTATCCGTGAGGCTAGCAAATGGAGTCCTGAACTGGCTGCTGCTTGTGAGGTATGGAAGGAGATCAGATTTAATTTTAAAGCAGTGGATACTTTGTAATTACCTTTTTTGTTATCTTAGTTGAATTGCAATTAAACTCGGCCCAATCTTTTCCTAAAAGGATTGAGCCGAATACAAAGATTCTTTTTTTTAATACATACTTATCTAGATATAGAAGATTTGAAATACAAAATCTAAGACTCAAATCTTTCTATTGTTGTCTTGGATCCACAATTAATCTACGGATCCTTGGGATTGGTCTATTCTTTTATATCCCTGAATCAAGCCAAGTATCACAATTCTTTCTACCCATCCTGTATATTGTCCTTTTCTTTCCATATTGGTGGAATAGAACCTTAAATTATTACGTTCTTAGGCGAAATTTTACGAAAAAATGATTTCTAGGATAGAACAAATATTTCTTTTTTCGATGCGAATTTGATACGACATAAGAAGAAGAGTGCTCTTTATCATTGTATTTATAATGACAAGAGGTTCCCTCATATCATATTCATATTCATATCATATTCATATATAGTGAAATATTACTCCGGATTTCCAAAAAGATAATTTTTTCAATACTCACACCTATTACCTTTTTCTTATTAGTTAATAAACAATCCTAGCGATTGGGTTTCTATATTTAGTCTAATAGGAAAGAAGAAATTCAAATAAAGAATTTTTGATCGAATGACTATTCATCTATTGTATTTTCATGCAAATAGGGGCAAGAAAACTCTATGGAAAGATGGTGGTTTAATTCGATGTTGTTTAAGAAGGAGTTAGAACGCGGGTGCGGGCTAAATAAATCAATGGACAGTCTTGGTCCTATTGAAAATACCAGTGAAATTGACGATTCGAATAGAAAAGATACAACTAAAAATATTCAGAGTTGGGGGTCCTGACGATTCTAGTTACAGTAATGTTGATCGTTTATTCGGCGTCAAAGACATTCGGAATTTCATCCCCGATGAAACTTTTTGGTTAAGGATAGTAATGGAGACAGTTATTCCATATATTTTGATATTGAAAAGCAAATTTTTGAGATTGATAACAATCATTCGTTTCTGAGTGAACTAGAAAGTTCTTTTTCTAGTTATCGGAATTCTAGTTATCTGCATAATGGATCTACGAGTGAAGATACCGACTATAATCGTTACATGTACGATACTCAATATAGTTGGAATAATCATATTAATAGTTGCATTGATAGTTATCTTCAGTCTCAAATCTGTATCGAAACTTCCATGGTAAGTGATAGTGATAATTGCAGTGATAGTTACATTTATAGGTCCATTTGTGGTGAAAGTCGAAATAATGGTGAAAGGATGGGTTCGGATATACAAACCCACGCGCAAGATAGTGATTTAACTATAGGAGAAAGTTCTAATGATACTGATGTAACTCAAAAATATAGGCATTTGTGGGTTCAATGCGAAAATTGTTATGGATTAAATTATAAGAAATTTTGAAATCAAAATGAGTATTTGTGAACACTGTGGATATCATTTGAAAATGAGCAGTTCAGATAGAATAGAACTTTCGATCGATCCGGACACTTGGCATCCTATGGACGAAGACATGGTCTCTCTAGATCCCATTGAATTTCATTCAGAGGAGGAACCTTATAAAGATCGTATTGATTCTTATCAAAAAAGACGGGATTACCTGAGGCTGTTCAAACAGGCATAGGCCAACTAAACGGCATTCCCGTAGCAGTCGGGGTTATGGATTTTCAGTTTATGGGGGCAGTATGGGATCTGTAGTCGGGAAAAAATCACCCGTTTGATTGAATACGCTACCAATAAATTGCTACCTCTTATTATAGTGTGTGCTTCCGGGGGCGCGCATGCAAGAAGGAAGTTTGAGCTTGATGCAAATGGCTAAAATATCGTCTGCTTTATATGATTATCAATCAAAAAAAGTTATTTTATGTATCAATCCTTACATCTCCTACTACTGGTGGGGTGACGGCTAGTTTTGGTATGTTGGGTGATATCATCATTGCCGAACCCGATGCTTACATTGCGTTTGCGGGTAAAGAGTAATTGAACAAACATTGAATAAAACAGTACCCGAAGGTTCGCAAACGGCTGAATATTTATTCGAGAAGGGATTATTCGACCTAATCGTACCACGTAATCTTTTAAAAGTGTTCTGAATGAGTTATTTAAGTTCCACGCTTTCTTGCCTTTGACTAAAAATTCAATCAAAACCAAATACCAAATAGAGCGTTAAGTTCAATTATTTGTAGCAAACGAGTAGTTAGTTTATTCGGAATTAAAGGAAAAAAATAGGAATTTTGTTTGGTGACCTAAGATCTAATTGTAGAAAGAAGCAAAAGCTGCGGATAACCCTTTTACCTATTTACCTATATTTCTGATTACTAATCAAGAAGTCTCTATCAAAAAAGAGTGAATTCTTCCTTTCATGAAATTAGGCAAACAAAACGAATTTCTTCTTCTGATCTTACGTATATAATTCAAATAGAAAAAATAGAAAGTTTTGTGGTTTTCTCGATTTCCCGAAATCCGGTTTAGCTAAAAATACCTCCGGGTTCGAATTCTAACGAATCCTTCGATAATCTGGAAGAAACTCTTTCTTTAGTAAAAAGAATAAAAGAAAAGAAATCAAGAAAAATAATAAAGTTGATTATCGTACATATCTTTCATGTAGAAAGATGAATAAGTTCATTTATTTAGCTCTACATTCCTTGCACTTATTCTATACCCTCACTTAGATATATAGATACTTATATCTATACTAAGAATTGAATTAATAATTCAATAATACTGAAAATTCTTACTTAATTATAATTATTATAAGATATCTTTATTAAAAATAATAATAACAGGTACGAACAATAAATCGAGGTACCCATTCTATGACAACTTTTAGCTTTCCCTCTATTTTTGTGCCTTTAGTAGGCCTAGTATTTCCGGCAATTGCAATGTCTTCTTTATTTCTTTTGTTCAAAAAACAAGATTGTTTAGGCCCGCTGGACCCCATCTCTGCTATTTTTTTTCAAAACTTAGACTTGCATCATAACTAACACAGATATCTATTTAGCGAAATATGATATAACATGTGATTTCTGCCGAACATAAAAGAAAGGACTCTTAAGAAACATAATAATATATGGGTAAATGAATTCTAGCCGTTTTCAAATCGACCAGGATCGCTGGATGACTGAAATAAAAGCCCTCGGATCTATATGTATATATATGTATAGTAGGATCATATGAAGGGTATGTTATTATTTTAGTTTAGATTACATCTAAGTAATTTGATGAATTACTCCTAAGGGTTGACATCAAAGTAGTGCTAGTTGATGAGAGTTACTTGGGAAACAAAAAGGTAAAGTCAAATTAATTTGAGGGTTTCTCTCAATTCCAATAAAATACAATCGGATCAAGTATGAGTTGGCGATCAGAACATATATGGATAGAACTTATAACGGAGTCTCGAAAAATAAGTAATTTCTGCTGGGCCTTTATCCTTTTTTAGGTTCATTAGGATTCTTATTGGTTGGAACTTCCAGCTATCTTGGTAGAAATTTGATATCTTTTTTCCGTCTCAGCAAATAATTTTTTTCCACAAGGTATCGTGATGTCTTTCTACGGAATCGCGGGTCTCTTTATTAGTTCCTATTTGTGGTGCACAATTTCCTGGAATGTAGGCAGCGGTTATGATCGATTCGATAGAAAGGAAGGCATAGTGTGCATTTTTCGGTGGGGATTCCTGGAAAAACCGTCGCATATTACTCCGATTCCTTATAAAAGATATTCAGTCCATTAGAATAGAAGTTAAAGAGGGTATTTACGCCCGTCGTGTCCTTTATATGGACATCCGAGGCCAGGGGCCATTCCCTTAACTCGTACTGATGAGAATTTGACTCCACGAGAAATTGAACAAAAGCTGCTGAATTGGCCTATTTCTTGCGTGTACCAATTGAAGTATTTTGAAAAAAAATGGGAAATGGGTACTTTGAGGGAAAAATGCAAGAATCCTCTTTTTCTATAACATAACCTAAGTGAAGTTTCATCAGAAGGGTCATTCGAGCCAAAGCGGGCGGAATACCTACAAAGCGAAATTCTTTATTTTTGTCACTCGACGTTTTATTTTCTCCTTTCTTTTGTGTTCCTTAATAACCAACACAAAAATAACAATTAGATTTCTTAATAATGATAACTAGCCAATTTCTGTCTTGTTTTGCTACTTTGAGTATCGCAATATCTTTCCCTCAATTATTCTACTATTCCTGTTTGTGGGCAATCAGTGAATTTTTTTTGAAATATTGGATATTGTGGATTCTTTCGTCTCAAAATTGGATTAATATTCATTACTTAAAGTGGTTCTTTCAGTCATTCATTGAAAGGAGACTGTTGTTTCGAATTTGCCCAATTGAGATATCGGGAAACCCTATTTTTTTAGTATTTCTTCATTCGAAATGGATTGATTATTAGTCGATTTCTCTAGTCTTTCGAGATTGAAAGACTAACCACAAAATCACAAATAAAATAGATTCATAGGTTCCATACCTTGTATAGAACTCATGCGTGAAAGAAGGATTCGATCCCATAGAGTCGACGAATGAGGTGGGTTGATTAACAATTCACAGATGAAAAAATGGCAAAAAGCATCCACTCCTCTTGTATCTATAGTATTTTTGCCTTGGTGGCTTTCTCTCATTTAAAAAAGTCTGGAATCTTGGGTTACTAATTGGTGGAATGCTGGGCAATCCGAAATTTTTTGAATGATTTTCAAGAAAGGGGTATTCTAGAAAATTCATAGAATTAGAGGAACTCCTCGTCTTGGACGAAATGATCGAAGAATACTCGGGACACGTCTACACAAGCTTCCTATAGGAATCCAAAAGAAACGATCCAATTAATCAAGATACACAACGAAGATCGTATCCATACGATTTTTCACTTCTCAATAAATATAATCTGTTTTGTTATTCTCAGTGGTTATTCTATTTTGGGTAATGAAGAACTTGTTATTCTTAACTCTTGGGCCCAGGAATTCTTATATAACCTAAGCGACACAGTCAAAGCTTTTTGTATTCTTTTATTAACCGATTTATGTATCGGATTCCATTCACCCCACGGGTGGGAATTAATGATCGGCTCTGTCTACAAAGATTTTGGATTTGTTCAGAATGATCAAATCATATCGGGTCTTGTTTCCACTTTTCCAGTCATTCTTGATACAGTTTTAAAATATTGGATTTTCCGTTATTTAAATCGTGTATCCCCGTCACTTGTAGTTATTTATCATTCAATGAATGACTGATAAAAGATCCACTCATATTAATCTAATCCAATTCGAAGGTTTGCTACTTTGTAGTTGTACATAAACAAAGCGTTGAAAATTTATGCTTTCTTTTTACCCATCTTCAGGATTCATCCTCCTATATTATTCCAGTAACCAGTCAAATTATTATTATTCCAGTAACTAACAGAATCGTGGATAGGGAACTATACTAGCGACTTACCCCACCTATTGTAGAAATTTTGGTATCAACGATTGAACCATGGAAACTAGAAATACTTTTTCTTGGATAAAGGAACAGATTACTCGATCTATTTCCGTATCGCTCATGATATATATCATAACTCGGACGGCCGTTTCAAATGCATATCCCATTTTTGCACAGCAGGGTTATGAAAATCCACGAGAAGCGACGGGCGTATTGTATGTGCCAATTGTCATTTAGCTAACAAGCCCGTGGATATTGAGGTACCGCAAGCGGTACTTCCTGATACTGTATTTGAAGCAGTTGTTCGAATTCCATATGATATGCAACTGAAACAAGTTCTTGCTAATGGTAAAAGGGGGCTTGAATGTAGGGGCTGTTCTTATTTTACCGGAAGGTTTTGAATTAGCCCCCGATCGTATTTCTCCCGAGATGAAGGAAAAGATAGGAAATTTGTCTTTTCAGAGCTATCGCCCTAATAAAAAAATATTCTTGTGATAGGTCCTGTCCCCGGTCAGAAATATAGTGAAATTACCTTTCCTATTCTTTCCCCTGACCCCGATACTAAGAAAGATGTTAACTTCTTAAAATATCCTATATACGTAGGCGGGAACAGGGAAGGGTCAGATTTATCCCGACGGTAGCAAGAGTAACAATACAGTTTATAATGCTACAGCAGCGGGTATAGTAAGTAAAATCATACGAAAAGAAAAGAAGGGGGATATGAAATAACCATAACAGATACGGATGGACGTCAAGTGGTTGATATTATCCCCCAGGCCCAGAACTTCTTGTTTCAGAGGGGAATCCGTGAAATTTGATCAACCATTAACGAGTAATCCTAATGTGGGCGGATTTGGTCAGGGGATGCGGAAATAGTACTTCAAGATCCATTACGTGTCCAAGGCCTTTTGTTCTTCTTGGCATCTGTTATTTTGGCACAAATCTTTTGGTTCTTAAAAAAACAGTTCGAGAAGGTTCAATTGGCTGAAATGAATTTCTAGACTTGCGGATTTATTGACATCGGGTTTGTAAAAGGGATTATTCGTCTTCCCCGCCTTCGGCACAAGAAAGGAATTTGCTACACCCCTTCTTGTGCCGAAGAGTAATGATTCTTGATCCTCTTTTCAAATATTCCTAGTCTTTTTTTTTCCAGATTTACCAGAACCTTTATGATTTTTACGAAACATTCTAAGTATAAGAAGTAGTGGACAAATAAAAAACAAGAGGGACATTCATTAAATAGAACTTATTGAATGAACTTATAAAAAATTTAATTTTGATGAGATACTAAAATAAAGAGAGTCAAAATGAAAATCAAATAAATAGAGTAAAGTTCTATTAGTATAGAAAGTATTTACCCGATATCTAATCTACAAAATATAGATTCTATCATCCAAGAAATTGAGCGATTCCTTATTCCTTCTTTCTTCTAACTTTGAAATGAAAGTACGGATAAATACTCTCAAGGAGGAGGTGTTCTGAATCAATCAATGGAGTTCGTTTTTCAAAAGCATCACCAGAGAGTGGAGTTTTTGAAACGGAAGAAAAGGGATTTCTCATTTAGACGAAAAAGAGTATGATCCTAAGAACTCAACGGGCCCTTCCCCTTGAATCAGACAAACAAAGAAGGAAATCCTGTTGAGTTCTTATGCTTTCATGTCTACAACTCAATTCATTCGAGTACTAGAGGATGAACCCAATCCGGAATATGAACCATAAAAGAAAACACCTATTAAACCGATCACAAGAATACCTGTTACAGTACCTATTATCCAAAGAGGAATCCTTCCAGTAGTATCGGCCATTTACCCCACTTCCCTCCACATTTCATCAAGTGTTCGTGCTAGAGACATAAACAGTCATGGATAATTATGAGATGAGACCCTTCCGAATGCGCTAAGAGAATGCCTAGAATTCTTATTTATTCTCTTTCGTTTTCTTAATTGAAGAAATAATTGGAAAATAAAACAGCAAGTACAAAATGAGTAATAACCCCAGTATAAACTGGTACGATTCAATTCAACATTTTGTTCGTTCGGGTTTGATTGTGTCATAGCTCTATAATTCGGATGTAGGTTTATCGTTGGATGAACTGCATTGCTGATATTGATCCCAAAAAAGACGGTAGGTACAGCTAGGCCGTGAACAGCCAACCATCGTACTGTAAAATTGGATAGGTTCGATCTATAGTCATTAGGGCCTCCTAAAAGATCTACTAAATTCATCAAGTTGTTCCAAAGGATCAAAACGGCCAGTTATTAATGGAATTCCCTGTCGGCTCTCTGTGAAATACTCGTTTGGCCGAGGACTTCCAAATACATCATAAGCTAAACCGGTGCTGACAAATAACCAACCCGCAATGAATAGGGAAGGTATAGTAATGCTATGAATGACCCAGTATCGAATACTGGTAATAATATCAGCAAAAGAACGTTCTCCTGTGCTTCCAGACATGCTGAGCTCCACATATACTTGTACAGTCAAAGGGATCGATTCCGTAAAAGATGAGATCAGTAAATGACAATTCACCGAAAGCAAATCTTTGTTGGATCATCAATATGGTACCGAGGGCGTCTTTAGAGTATACCGAATCAGTATAGCTATCCTTCTTCTGACACAGCAACGCAATTTTTTTTTAATCAGCATCGAAAATGAGTGCTAAATAATTCCTTTTTTCCTTTACAGCTTCTTTATTGATGTAAAAGGATGCTCCATTCAATAGAGAATTCGTCCAATGCAAGAGATTATCATATTCCCACAGATGCGAGATCTAGAAATTTACCCTTCAGAGTTGTAGAAGTAATTTTTGTTGGAATCTTTGTTTTTTATTTATTTTAAAAATTGGAATCGTCCAGTAACAAATAAGTGTATTAGATCGGATTCTATCAAAGAAAGAGAAGAAAGAATAAAATAATTGGAATCAATAGTTGTGATGCATTCTTGTACTCGACCGAGAACCAAAGGCCCGGCTACAGGGGTGAGCGAGGTTTTGGAATATGGAAAGATAAATGTGCAACCTATAAACGATAATAAATCAAAATTATTAGTTTTAGGATAAAAAATATTTTTTGAGTGATCGTGTAATAACTTTTTCTTCTCATTCAGTCAAGATATTATGCGAATGAACCTATTGCTGACTCTAATGAGTTAAATTGAAAGTAAAGTAAAAGTTTTATAAGCTTACTCTTCGCTCTAAAATCGACAATAGATTCGATAGAATTCAAAAATAGACGAAATGGTCAAAAATGAAGTTACGCGGCCCGGCTCTCATTACTTATTATTTTATTAGTTTATCTAATTTTATTAGTTTTACTCAATGAATTGAATCGGTTGATTGAAATCGCAAGATGGATAGATATTCCAGATGATGAATCGATTTCATTTTATATACCTGTTACTTTTTCTTTGTTAGTGCCGTCTATAATGATAGATGAATAACAAACTTTCAATTGAGCTTCTTCTTTCAATTGGTATTTTGTGTATCCTCTTATTTTGAAAAAAAAATGGAAACTTAGGTAAGTGCTTTATAAACATATGTATAAAAAAGATATTTCATTTAGATCCTTTATGCTTACTATAACTAGTTATTTTGGTTTTCTACTAGCGGCTTTAACTATAACCTCCGCTCTATTTATTGGTTTGAGCAAGATACGACTTATTTAAAATGAATATTTGAATAAACCTTTCCGCGAGATTACATGTATTCTATAGTTACTTACAGCCTCAATTGTCAATTCTTGGTCATTGTCATTGAGATTCATGTCAATTCGGATTAATATTTAGGTATAGATATTACCTCTTTTTTTCTTTCAAACAAATTGAAATGATTGAAGTTTTTCTATTTGGAATCGTCTTAGGTCTAATTCCTATTACTTTGGCTGGATTATTCGTAACTGCATACTTACAATACAGACGTGGTGATCAGTTGGACCTTTGATTAACATCTCTTTTTATCGACCTCCTCCTTTTTTATTTAATCCACAGGAGGTCAAATTCCTATTGCTGTGCAAAAGTTACTGAATCAATTTCAGTCTAATTTGATCTAAGAATAAAAAATCACGCTCTGTAGGATTTGAACCTACGACATTGGGTTTTGGAGACCCACGTTCTACCGAACTGAACTAAGAGCGCTTTCTTATATGAATAGATAAGACTGTAAAGAAAAGGATTACCCCAATCCATTTTATTTTGGGCGCATAGTATCATAAAATGAAAGACTATGCCCAATTAAAATCGATCTCAGCCGATCCTCGTTACTGCTCAAAGGAGCGGTAATAGGTAGGGATGACAGGATTTGAACCCGTGACATTTTGTACCCAAAACAAACGCGCTACCAAGCTGCGCCACATCCCTTCCGTTGCTCTACAGTGTCATTGTACAGAATTCCTGTCTTGTTTTCCACATCGTTATTTCCTCCGTTGATATACACAATTTTCTGCCCATTTCGTCTTTTTTGTCTCATTTAACATATAATAATTTAACATATAATAATAGTAAAAGACTTGTATACAAAAATAAATGAGTATTTTTCGCAAATGCTCGGTAAGGGGAGATGCTTTTTCTGTTTTAAGAAAAGGTAAAATCCTATTTACTTTTACTGTACTGGTTTACTGTACTGGATCATTGTACAATTTAATATATTAATAATAGAGGAATTTTATGGATTACGTGTACAACTATAAGTGGTCCTTAACTACCGATTTTTGTTTTACAATAAAAAGGAGGGTTTTCAATGCGAGATTTAAAACATATCTCTCCGTGGCACCAGTACTAAGTACGCTATGGTTCGGGGCTTTAGCAGGTCTATTGATAGAGATTAATCGTTTTTTCCGGATGCGTTGACATTCCCTTTTTTCATTCTAGTTATTGAGATGCGCTAGTTATTGAGATGGGAAGGAATGAAGAAGGTTAAAGATACAATCAAATATCTGTGACTAATCCCCTCTCCTCTTTTCTCTTTTTCCCTTTTTCTATTTTTAAAATAAGGGAGGAAAGAGAAAAATAAGAGTGGATTCAACATATGCGAGGCTCGGGTTCAATATTCAATAAATGAATATTATTAATAAATAATATTCATAATAAATAATAGAGGAATGGGGAGTAGAATAGAAAATGTGGGTCTAAGGAAAGAGTATTATACAAGATATTTAAATGAAAAACGAAATACTGTACTTCGATTTGAAATAGAGTTTCGAAATCTTTGTATTACTTATTATTTTTATTTTATTTACTATGACTTTAATTTACTAGGTACTTTGATCTTTCTTTTAGAATTCGATTTCAAGTTAGTAAATTCTATTTGTTTTCCTTCTTTTCTTCTTCTTCGTTTTGTAGAAGAGTTGAATAAATCAAAAATCTAAAGGAGGTTCATGGCCAAGGGTAAAGATGTCCGAGTAACGGTGATTTTGGAATGTACCAGTTGTGTCCGAAACGGGGTTAATGGGGTATCAAGAGGCATTTCCAGATACATTACTCAAAAGAACCGTCACAATACGCCTAATCGATTAGAATTGAGAAAATTCTGTCCGCATTGTTACAAATATACAATTCATGGGGAGATAAAGAAATAGGGCGAACCGAGTACCTGTATGTTACCCTTTCAAGCAAGGGTAAAAATGACATTATATTATATATAATATATTTAAATAGAAAATAAACAAATCCTATATCCTATTTGGGGTGACTTTCAAAATTAGAATTAAGAAATAGGATTTTCGAGATAAAGATAAGGAATAAACTAAAACAAACTATGGATAAATCCAAACGACCCTTTCTTAAATCCAAGCGATCTTTTCGTAGGCGTTTGCCCCGATTCAATCGGGGATCGAATTGATTATAGAAATATGAGTTTAATTAGTCGATTTATTAGTGAACAAGGAAAAATATTATCTAGACGAGTGAATAGATTGACCTTGAAACAACAACGATTAATTACTATTGCTATAAAACAAGCTCGTATTTTATCTTTGTTACCCTTTCTCAATAATGAGAAACAATTTGAAAGAAATGAGTCGACCGCTAGAACTACTGGTCTTAGAACCAAAAATAACTAGGCTTACTTTGGAATCATAATTTTAATCCGAACTCAAAGGCAGGTTGGTATTTTTCCGAGAATCCAGATTTGATTATCGGGTCGTAAGAAAAAAGAATTGGAGAAAGCTTTTTCTACTGAGCGTGTTCATTCGTTTTGACTATTTTAGCATATTTTTTATCCCAGTAATTTCTACTCTACCTTCCCGGAGTTCATTCTCCGGGAAACTCCGTTTAAATTATTCCGACGGACTTTTTATAACCCACTTCTTTTATTATCTCATTGGAAATCATATAAAGACAATCCCTATTTAATATAGCAATTTGTGCAAGTATTTTACGATTAAGAAGCAAGCGTCTCTTGTACAGATCGTGTATTAATCTACTATAACTATAGGATACCCTCCTTCGCGAATTACTGCGTTTATCCGAGTGATCCACAAACGACGAAAATTTATCTTTTGACTGCCCTGATCCCGATGAGCCGAAACCAAAGCTCTTATTTTCTGTTGAGTAATAGTTCGAGTAAGTCTTGAATGGGCCCCTCGAAAGCTTGATGCAAATAAACGAATTTTTGTTCTACGTCTACGAGCTATATATCCCCGTCTAATTCTAGTCATTGAATAGATGAAACTTTCACGAATAACTAATTTATTTCTTTTCTTTCAGTTATTCTTTTACCTTTTCCTAATCTATTAATAACAAAACGGATTTTTCCAATGTATAAAATAAAAATTCCAATGGCTTTGGCTACTATAACCTTCCTGACCGCGATTTTTTCTTTTTTTAGGCATTTCAATGCGAAATAAGAAATTTATTGTGTTATAGGTGTCAAAAATAGAAAATATAAATGAATAAAGAAATAGCGGGTTCCTTCGTTTCTATGGTGACTTCCTAAACGGTGAGGTCTTCTCTATACACCGGAGCCTTTACTTCATTTAATCAACGTTATTGGTAACTTGTATAGTTCACCCTTTTGGCTCTACCCATGAATTATCCAGCAATAGGCCTTTCACAATGAGATCTACCTATACAGTAACGGTATTTAATTATGAAAGTTAGCTGGGTAGCTGACCCTCTTAGTCCGTTCTTGCCAGAGTAGGAGCTTAATCTTTATGCTCCTTTAAGATTTCCTCCGCTTAATGGCTAACCTTTTGCTACCAATGGAGAATTGCTTCTCATCTTAAATTGAGGTGATTGGATTTGCACCAACGGAAACCATAAAATTTATACACAATGTAGGAATGTGATAACTTTGATTATTTTTATATGGTTATATAGTGAATGGAATCCTTCTTACATTCTATACTATTCACCGGTACTGATCATCGATACTGGAAAGTTTTTCTTGCTTTTGTACCAGTTCATGGTATGATCTAAACGAGTCGCACATACACCCGAGTACATGTTCCTCGACGTTGAGGGCATCCCCGAAGAGCAGGGGATTTCGTGACATTTCTGATTGGCTGTCTTGTATTTCTAATAAGTTGTTTAATGGTTGGCATACTGAATTGTATACATAATGGGCTGGTTTAGATTGATCCTAACCGGATAATTATGAATTACTTCCATTTATTAGATTTATTAGAATAGTAAAATCATAGATAAAATCTCAAATCACGGATTTGTGTGAAATCCGTCTTATTTTCATTCAACCGCTACAAGATCAACAATTCCATAAGCTTGTGCTTCTGTTGCTGACATAAAAACATCTCTTTCCATGTCTTCGGATACAACCCATAAGGGTTTGCCTGTTCGTTGTACATAAACCCTTGTGAGGGTTTCACGCAGTTTCAGCAATTCTTCCGCTTCCAGGATAAATTCTCCCGCTTGTGCCTCATAAAACGAACTAGCGGGTTGATGGATCATTACCCTGATATAACATAATAAAAGTTCCTCTATCTCGCATGATGAAGCGAAGAAAAAGAAAGATAAAGACTAATATAATAGGAAAAAGATAGAATTGAACAACCGTACGGGCATCTTTGGTGCATTGCATATGCATACGGCTTTACAATAAAATTGACCCTTACCTTCCAAGAAAGAGAAAAGTAAAATATACCAGACCCTGGTGGGTTAAATGATCAAATTGCCATCCTTCCTTTTGGAATAGTTAAAACTACTATGATGGCTCCGTTGCCTTATATATTAATATATTCATTTTTTTTTTTGTTTGTGATTCAGCAATCCCAAAGTTTCTTTTTGAACCAATCAAAGAAAAATCTTGTTTTTTCGCACTCCTTGCATAATATAAATATTTTTAAGAGCCTTCCGGCGTGAACAAAAAAAGGTTTGTGACGCTGAGCCGGGCTCCCGATAAATAAGAGAAAATCGGAAATACCCTTTCTCCCATACTACTCTCTCGATACATAATCTAATGTTTTGAAAAACAATGCAAAATTTATCATATCGAATTCGAAGTGCCATGCTATTATTACTCGATATATATTCATATTTCATAGGGCGAAGGCATAGTCTTCTTTTTCTCTTAAATCATTGGCGCCAAGCGTGAGGAATGCTAGACGTTTGGTAATTTCTCCTCCGACCAGGACAAAAGATCCCATTGAAGCGGCTAACCCCATGCATACTGTATGGACATCTGGTCGTACAAATTGCATAGTATCATAAATCGCTACTCAGGTATTACCCAACCGCCGGGAGAGTTTATAAACAAATACAGATCCTTGTTATCATCTTCAATACTGAGATATATCATAAGACCGATAAGTTGATTTGAGATCTCGCTATCAACTGCTTGTCCTAAAAAAGTAATCTTTCTCGATAAAGTCGGTTGATTAGGGTACAATTGTAGCCCCTAGGAACCGTACACGCGTCTTTTGATGCATACGGTTCAAAAAATTGTGAAAACAAAAAATCAATGTATAGATTCCAGTCCTCTTTCTTTTAGATTCTTTATGACTTCTAACGAGAGGTTTTGTCTTCTTCAATAAAGACGGGTTTTACTTTCTTTTGACTTTATACTTTTCTAACTATTATATATAATATATAAGATATATAATAATAATATGTTAATATTAAAAATTAATATATAATAAAAATCAAATATAAAATATATAATAACTAAACTTATTGAATTAACTTCTCATTGATGTATTTTTCATCGAGATTCAATCCTAATCACGATGGTATTTTCTTGTTCCTGAGTGGGTCTCTTTCATCTTTTAGGTTTATGCTCTACTCCGGGTAAATTCGCCCGATTTGAATTTGCACATATAGGACAAGCACTCCCGCATTACCATTTCTTTTGTTATGACTTTCTACTTTTCAATTCACTTCTATCGAGTTTGTTCATTAACAGTTTAAGATCAACTCGGCTGAATCATTTCTAATAGAACTCATAATCATAGATATATTACCAATTCTGTTGGGTTTTTCTAAACGGAGCCTGGATACTTCATTTTTTTAGTCCAACCAAGACAACCATAAATTATTCTAATTGATAATAGTAATATGAATCCTCCCAAAATGGATCTAATTGTACTTCACGCTCCAAACTTTTGATGATTCAATGAATCTTTATTGGGCGAAACAGAGGATATCTCGATTGTGGGAGATAACGAAATCCCATATGACCCAATATATCTGACAAGTCGCACTATACGTCAACCCAAGATGCATCTTCCTCTCCAGGACTTCGGAAAGGTACTTTTGGAACACCAATAGGCATTAATTGAAAGAAAAAACTAAGTACTATATTTTACTTTGATGTGGAAACGTAACGATATGATTTTTTGTCTTTAGAATATTGGCTTTTATCGTATTTATTTTATCCATAGATTCGAAAAGGTCATAAAGAAAAACAGAATGAATAAAGTCAAATTATTACGAATAGGGCGATGAATAAATATGTACGCATTCGCTCATAGAAAATGGTATTAGCCCCGTTGCATATTGATACTTATCGAGTATAGAATAAATCTGCTTCTCTTTGTTTCTACGAATAGAATTGTTCCATTATTTTATTACCAACAGAATAGAACAAATATTAACCCCTGCTCTGAAATAATTCACCGAAAGGGAGGTCCATAGGATAGTCATAGTAGAGTCTTTTCCAATGCAATAAAGTTACGTAGTGTTTATTTATCTTTGATAAAGGGGTATTTCCATGGGTTTACCTTGGTATCGTGTTCATACCGTTGTATTGAATGATCCCGGCCGGTTACTTTCTGTTCATATAATGCATACAGCTCTGGTTGCTGGTTGGGCAGGTTCGATGGCTCTGTATGAATTAGCAGTTTTGATCCTTCTGACCCTGTTCTTGATCCAATGTGGAGACAGGGTATGTTCGTTATACCCTTCATGACTCGTTTAGGAATAACCAATTCATGGGGCGGTTGGAGTATCACAGGAGGGACTGTAACGAATCCGGGTATTTGGAGTTACGAAGGTGTAGCTGGGGCACATATTGTGTTTTCTGGGTTATGCTTTTGGCAGCTATCTGGCATTGGGTGTATTGGGATCTAGAAATTTTTAGTGATGAACGTACAGGAAAACCTTCTTTGGATTTGCCTAAGATCTTTGGAATTCATTTATTTCTTTCAGGGGTGGCTTGCTTTGGTTTGGTGCATTTCATGTAACAGGCTTGTATGGTCCTGGAATATGGGTGTCCGATCCTTATGGACTAACAGGAAAAGTACAACCTGTAAATCCAGCATGGGGCGTGGAAGGTTTTGATCCTTTTGTTCCGGGGAATAGCCTCTCATCATATTGCAGCAGGGACGTTGGGCATATTAGCGGGTCTATTCCATCTTAGTGTCCGCCCCCACAACGCCTATACAAGGGATTGCGGATGGGAAATATTGAAACCGTCCTTTCTAGTAGTATCGCTGCTGTCTTTTTGCAGCTTTTGTTGTTGCCGGAACTATGTGGTATGGTTCAGCAACTACTCCGATCGAATTATTTGGGCCCACTCGTTATCAATGGGATCAGGGATACTTCCAGCAAGAGATATATCGAAGAGTTAGTGCCGGGCTAGCAGAAAATCAAAGTTTATCAGAAGCCTGGTCTAAAATTCCTGAAAAATTAGCTTTTATGATTACATCGGAAATAATCCGGCGAAAGGGATTATTCAGGGCGGGCTCGATGGATAACGGGGATGGAATAGCAGTTGGATGGTTAGGACACCCCATCTTTAGAGATAAAGAAGGACGTGAACTTTTGTACGTCGTATGCCTACCTTTTTGAAACATTTCCAGTCGTTTTGGTAGACGGCGACGGAATTGTTAGAGCGGATGTTCCTTTTCGAAGGGCAGAATCGAAGTATAGTGTTGAACAAGTAGGTGTAACTGTTGAGTTCTACGGTGGCGAACTCAACGGAGTGAGTTATAGTGATCCTGCTACTGTGAAAAATATGCTAGACGCGCTCAATTGGGTGAAATTTTTGAATTAGATCGTGCTACTTTGAAATCCGATGGTGTTTTCGTAGCAGCCCAAGGGGTTGGTTTACTTTTGGACATGCTTCATTTGCTTTGCTCTTCTTCTTCGGACACATTTGGCATGGTGCTAGAACCTTGTTCAGAGATGTTTTGCCGGGATTGACCCGGATTTGGATGCTCAAGTAGAATTTGGAACATTCCAAAAGTTGGAGATCCGACTACAAGAAGACAGGCAGTCTGATACAACGCCGCTTTGTTATCTTTTGTCTCTATTCTCGTTTTAGAATTTGTTATAGGGGACCAGCTCGCAGATAAAGAAAGAACAAAAGTGATGATTCAGATCAAGATTTCTCTGGTCCCCATAAAAGAAAATCAAAATAAGCAAACAGGTATGGAAGCTATAATTGTAAATCGCGATCGAATCTATGGAAGCACTGGTTTATACATTCCTTTTAGTCTCGACTCTAGGAATAATTTTTTCGCTATCTTCTTTCGAGAACCGCCTAAAGTTCCAACTAAAAGGTTGAAATGATTTTCATTATCTCAATTGAAGTAACGAGCCTCCCAATATTAATATCAGGAGGCTCGTTACTTCAACTAATCCCCATGTTCCTCAAACGGATCTCTTAGTTGTTGAGAAGGTTGCCCAAAAGCGGTATATAAGGCGTACCCAGTAAAACTTACAAGTAAACCAGATATAAAGATGGCTACTAGGGTTGCTGTTTCCATTCTTATATAATTTCAAGACCCCAATGGATCTATGATAAGATCGTTTATTTACGATGGAATGGTATACAAAGTCAACAGATCTCAATGAATACAATAGGATTTATGGCTACACAAACTGTTGAGAACAGTTCTAGATCTGGCCCAAGACGAACTACTGTAGGGAGTTTATTAAAACCATTGAATTCGGAATATGGTAAAGTAGCCCCTGGGTGGGAACAACTCCTTTGATGGGTGTCGCAATGGCTCTATTTGCGGTATTTCTATCTATTATTTTGGAAATTTATAATTCTTCCGTTTTATTGGATGGAATTTCAATGAATTAGATCTATAAGAATCGCAAAGTTCTACAAAATAAATCATTTAGAGCTCGGGTTTCGAGCCCATTCTATGGAGGTCGATCGCGGAATTTCTTTGTTTCTGTATTTCCGGAATATGAGTGTGTGACTTGTTATAATTGATCCTAGTGATATTACAGAAAAGGTTCTGTCATCTTGATAAAGATGGTTCTACTTCGTCGGATATTTATTCTAGTATCTGGAACACGAAATAGATTAAGAAATATTTGAACTATGATTCATACTTAATCTTCAGACCTCGTATCCGGACTCCAAAAATTTTCAAAGAATTCGAATTTTTAAATCGAAAGATTTTTCTTCTATTTCGATTTTGACCAAAAGAAAAATATTTCTTTGAATTTTTAGTCAGTCTATATATTCATGGAATAAGTGATGGTCCAATGGTTCTTACTCAGGGAATCCTTGACTTAACTTAGTATTTTTTATTGAATCATCGTGGTTCTAGTATGAATCTGAGGTTTTAATCAATTCATATTCATAGGGTTCTTAACAAGAGAATTCCTATCAATACAATAAGAAATAATAAAAGCCATATTATTATACATAAAAACAAATAAATAGGAAAGAGAGGAATCAAGAGGCCTGTGCGGATCAACATAAAGACGGCTGAGCCAACTTGAGATTTTGGTATTATCGCCACAAACAAAAAGAGCTTTGGGATTTTCTTCTTTCGTACCTTCAGAAAAGATTGAATCAAAGAAAAAGTTTCAAACTTTCCATTACAGATTCAATTAGTATTTGGGTGTTTTTGCTTGAGCTGTACGAGATGAAAGTCTCATATACGGTTCTCGGAGGAGTTCCGCCTATCTCAATAAAGTTTATGATTGGTTCGAAGAACGTCTCGAGATTCAGGCGATTGCGGATGATATAACTAGTAAATACGTTCCTCCCCACGTCAATATATTTTATTGTTTAGGGAATTACGCTTACTTGTTTTTAGTACAAGTAGCTACGGATTTGCTATGACTTTTACTATCGTCCGACTGTTACTGAAGCTTTTGCTTCCGTTCAATACATAATGACTGAAGCTAACTTTGGTTGGTTAATCCGATCAGTTCATAGATGGTCGGCAAGTATGATGGTCCTAATGATGATTCTGCATGTATTTCGTGTGTATCTTACCGGTGGATTTAAAAACCTCGCGAATTGACTTGGGTTACAGGCGTGGTTCTAGCTGTATTGACCGCGTCTTTTGGCGTAACTGGTTATTCCTTACCTTGGGACCAAATTGGTTATTGGGCAGTGAAAATTGTAACAGGTGTACCTGAAGCTATTCCTGTAATAGGATCCCCTTTGGTAGAATTATTGCGTGGAAGTGCTAGTGTGGGACAATCCACCTTGACTCGTTTTTATAGTTTACACACTTTTGTATTGCCCCTTCTTACTGCTGTATTTATGTTAATGCACTTTCCAATGATACGTAAACAGGGCATTTCTGGTCCTTTATAGATATAGAGAAGATATTTCATCGATATTTGGAATCAATCATTTATCACTTGGGAAGGAATAATAGTATTTCATTGCTAGAAATATGGATTATTAAAAAAATAAGACATGTATTTGGATATTTCCTTCAACTATTCCTGTCAAATAAATAATATTAGGGAGTTGAGGGAATTTTCTGAAGAGAAAATGGATTATGGGAGTGTGTGACTTGAACTATTGATTAATCTGTGTAGCTATATGCCTGCCACATTGGGATTGGAATTTACAAACCAAATGTGTCTTTATTCCAACCGCCGTAAGCCCTATACAGAGGATAGGCTGGGTTGCTTGAAGAGAATTGTTTCTATGATCAGATCCGAATCATGTTGTACATGAGCAGGCTCCGTAAAATCCGGTATAATAAATGAAATTGAAATAGATAAAAGATAATATATAGGTTATTTATTTCACTTACTTAAGATTTAATAGTATAGAAATGCATTCATTTCCTCTGCATTGACATGATCATAATACTCATACTATTGGAGTGAAACGGGGGTCTAAAGAAGAACAGGGGCTATATTAGTAACAAGTAAACCTTTGTGTGTACCTCAAAATCTTGGGGAATAAATACCAATCGTAAGGTCTGAGACGACCCAGAAAGCACTTGATCATGTCATGATCAACTTTGTAAGCCTACTTGGGTATTGAGTAGCTACTTAGAAGAACTGAACTCTTTGGAATCGATATTTGCAACTCCAGAAAAGAATTCAGTAAAATTATTCTTACATTTAATCCATGCATATATGAATATATAGGTAAGAAATAGATTTTATCTGGATTCGTTTGGTTCTTTGATTCTTGCTCGAGCCGGATGATGAAAATTATCATGTCCGGTTCCTTCGGGGATGGATCGATAAGAATTCACCTATCCCAATAACAAAAAACCTGACTTGAATGATCCTGTATTAAGAGCTAAATTGGCTAAAGGTATGGGTCATAATTATTACGGAGAGCCTGCATGGCCCAACGATCTTTTATATATTTTTCCAGTAGTAATTCTAGGTACTATTGCATGTAACGTAGGCCTAGCGGTTCTAGAACCATCAATGATTGGTGAACCGGCGGATCCGTTTGCAACTCCGTTGGAAATATTACCGGAATGGTATTTCTTTCCCGTATTTCAAATACTTCGTACAGTACCCAATAAATTATTAGGTGTTCTTTTAATGGTTTCAGTACCTGCAGGATTATTAACGGTACCCTTTTTAGAAAATGTTAATAAATTTCAAAATCCATTTCGTCGTCCGGTAGCGACAACCGTCTTTTAATTGGTACCGCAGTCGCCCTTTGGTTGGGTATTGGTGCAACATTACCTATTGATAAATCCCTAACTTTAGGTCTTTTTAATTCAATTGTGAAATAACACGACGTGTGTATCTAGGAATAATCGCTTCTAAAGTGAATTACCCCTAGATACATCTATTCAATCAAATTCTGAATCTATCTAGAATATATGAATTGTACTAAAGATTCAAAATAGATTTTCATTTTAAAAGAAAAAAAAGACGAAGTAAATTCAATAGATTTCAAACTTATTTTTCGGTAAAAAATTGCCAAATGTTTTTCTAGAACGACCAATATCTGTTTTACATCTTCTATGCGAAAATGTTCAATTTTCATAAGGTCTTCTTGACTGTTATTCAAAAGGTCCAATAGTGTATATATATTGGACCTTTTGAGGCAATTATAGATCCTGGGAGGTAATTCTAATTGGTCAATAAAAATCGATTTCAATGCTATTTCTTTTTTCTTTCTTATGAGTTTATTCAATTTATCGTGAAAGGTAAAAGGGAATAAAGGAACCTCGTGTTGATTGTTCTCTAAATGTGAGTTTTCTTCTTCCATATGTAAAAGGGAATAAACAAATCAATCAAATTCCGGGATGCTTCGTGAAGTGCTTCTTTCGGGGTTAAACTTCCGTTTGTCCATATTTCGAGAAAAAGTATCTCTTGTTTTTCATTCCCATTCCCATAAGAATGAATACTATGATTTGCATTTCGAACAGGCATGAATACAGCATCGATAGGATAACTTCCATCTTGAACGTTCTTTGGCGTTTGTATAAGATATCCGCGATTTCTCTCGATTTGTAATCCAATACAAAAATTGATCGGTTCCGTCAAGCTAGCTATATGTTGTGTATTATCAACTATTTCTACATAAGGTGGTAAGACGATATCTTGAGCAGTTACATATCCAGGCCCCTGACACAAATAGACGCGTCACAAGTTCCATATAGATTACTTCTCAATACAATTTCTTTCAAATTCATTAAGATTTCATGTACCGATTCTTGAATACCCTTTATGGTAGAATATTCATGCGGTACTTTATCAAATTTTACACGTGTGATACATGTTCCTTCTATTTCTCCAAGCAAAGCTCTTCGCATCGCAATACCTATTGTGTCGGCTTGACCTTTCAGAAGTGGAGACAGAATAAAACGCCCATAATAAAGACGTTTACTCTCTGTTCTTGATTCAACACATTTCCACTGTAGTGTCCGAGTAGATACTGTTATTTTCTCTCGAACCATAGTAATATAATTTCATTAGATCATTGAATCATTTATTTCTCTTGTTTCTCTTGAAAGTTCTTTAATGTGCATTTCTACACACGTCTTTTTTCGGAGGTCTACAGCCATTATGTGGCATAGGGGTTACATCCCGTACAAAAGTTAATAATATACCACTTCTACGAATAGCTCGGAGAGCCGCGTCTCCCGAGACCGGGGCCTTTATCATGACCTCTGCTCGTTGCATACCTTGATCCACTACTGTACGAATAGCATTGCCTGCTGCGGTTTGAGCAGCAAATGGTGTCCCTCTTCTCGTACCCTTGAATCCACAAGTACCGGCGGAGGACCAAGAAACCACCCTACCCCGTACATCTGTAACGGTGACAATGGTATTATTGAAACTTGCTTGAACATGAATAACTCCCTTTGGTATTCTACGTGCACTCTTACGTGACCCAATACGTCCATTCCTGCGCGAACCAATTCTCGGTATAGCTTTTGCCATATTTTATCATCTCGAAAATATGAGTTAGAGATATATGGATATATCCATTTCATGTTAAAACAGATTCCTTATTTATGTATCGTTTCATTTAGTGGATGTGATTATCCCTGCCTTTGTTTATGTCTCGGATTGGAACAAATTACTATAATTCGCCCCCGCCTGCGGATTAGTCGACATTTTTCACAAATTTTACGAACAGAAGCTCTGATTTTCATATTTGTCATTCCTTATCTTAAATTGTAATTTTCTTCTTGGAAGAAAAAGTTTCTTGAGATTTTGCATCTCGAATCGTATTCTCACGAAAGGGGTGTTCAAACCACTTAATCCTTGGAATCCTTGTTGCGGAGTCGATAAATTATACGTCCTTTGGTTGAATCATACCGACTTACCTCAACCTTGACTCTATCTCCCGGTAGTATCCGTATAAAACTACGTCGGATCTTTCCTGAAACATAACCTAGAATCAGATCTTCATTATCTAAACGAACCCGGAACATGCCATTGGGAAGCGATTCGGTAATTAAACCCTCATGAATCCATTTTTGTTCTTTCATTCCAGGTAAAGTCCCTTGAAGTATCAACTAATGAAGGAGGAACAATATTAGACAACTCGTCCCTTTTCTTTTTATTTTACAATTTAAAATTGTAAGTTTTGTGTCCAATTTGTAGATTACCATATATAACACAAAATTTCTCCGCCGATGCCTTCTAGCCGAGCCTCTCGGTCTGTCATTATACCCCGAGAAGTAGAAATAATTACAATTCCCATCCCGCCTAAAATTCGAGGAATTCGTCGATAATTAGAATAGATTCGTAGACCGGGTCGACTGATCCGTTTTAAATTTACAAGATTTCTACAGGGCCTTTTCCTATTCCTTCTATGTCGCAGCGTTAAAACCAAAAATCTTTGTTGTTTTCTCGATGTTTTCTCACGTTTTCGATAAAACCCTCTCTTAAAAGTATTTTGACAATATTTTCGGTAATATTAGTAGCTGTTATTCGAACCACTCTTTTTTATCCATATCAGCATTTCGTATAGAGGTTATTACCTCAGCAATAGTGTCCCTACCCATGATGAACTAAAATTGTTGGTGACTCAAAATTTGATATAATCAACATGCTTATTTCTTTTTTTTGTTTGTTTATGAATTTGAGTAATTAATTTGAGTAATTAAAGGTATATGCGTGAGATACAATCTATTTCTCAATCCATTTCTTTCAACTGTTCCACTATAAAATAGAACGATCCCTTTTATAATACTTCGGGAGCTAATGAAACTATTTTAGTAAAATTAAATTGTCTTAATTCCCGGGCGATCGCGCCAAAATTCGAGTTCCTTTTGGATTTCCTTCTTGATCAATAACAACTGCAGCATTGTCATCATATCGGATTATGATACCGTTATCACGTTTGAGTTCTTTACAGGTACGCACAATTACAGCTCTGACCACTTCTGATTTTCTAGGGGCATATTTGGTACTGCTTCTTTGATCACAGCAACAATAACGTCACCAATATGAGCATATCGACGATTGCTAGCTCCTATGATTCGAATACACATCAGTTCTCGAGCCCCGCTGTTATCTGCTACATTTAAATGGGTCTGAGGTTGAATCATATCATTTTGTAATTTGTTCTTTAATGCAAAGGACAAAAAAAGAAATATTATTTGTCTAAAAGAAAAAAAAGAAATAAGCCGTTTTTTCACACCCAAGACCCATTTCTGTTAGTTCTACCCTTTTATCCCGAAATAATGAATTGAGTCCGTATAGGCATTTTGGATGCTGCTATTGAAATAGCCCTTCTAGCTATATTTTCTGTGACTCCGCCCATTTCATAAAGTATTCGACCTGGTTTAACAACAGCTACCCAATATTCCGGGGATCCTTTCCCCGAACCCATACGTGTTTCTGCGGGCCTTAGTGTAACTGGTTTGTCTGGAAATATACGTACCCATAGTTTTCCACCACGACGCGCATTTCGTGTCATTGCTCGTCGACCGGCTTCTATTTGTCTAGATGTGATCCAAGCGGGTTCGAGTGCCTGAAGAGCATATTTACCGAAACAAATACGATTCCCTCGATACGATATTCCTTCATTCTTCCTCTATGTTGTTTACGGAATCTGGTTCTTTTAGGGTTATAGTTGATGGTTGGTTATGAATTCCATCTCTACTGCAGAACCGGACGTGAGAGTTTCTTCTCATCCGGCTCCTCGCGAATAAAAAATTGAAAAATAAAAGATTTCGAATCTTAATTAATTAACTAATTAAGATATACATGTATTTAAATTGAATCGTGGAATTTTTGAGATTTCATCTAATCTAATATATTAGTAATCTATAGATCTTGTTTAAATAGACAATTAAAGATTTTATTAAAGATTTAAGTTTCTATTTTCGAAATCTTATTGTTATTTTGAAATATAAATAGAACAAATTTTTGTGTTTTTATCGTCCAAATTTATCAATTCAAAAAAAAAGAAAGTTTTCGCGGGCGAATATTTACTCTTCTATTTCAATTTTCGGCTTGTCTCCTGACTTCTTACAATAGATGAATTGATCTCCGGTTCATTCCGCCATCCCGACCAGTGAATCATTAAGATTCCTTTTTTTGACTAAAATCTTTTGCATTCACAGCTTCCATCGTTCCCATCGCTTCTTACTTAATGCTTAGGTCAAAATTTTACAACGGAGCTCATAATGAAATTTGTTCTGGAGTCAATCTTCTTAGTCTTTATTGGCTCGAAGCTCTTGATTTTTTGTTTTGTTCTATAATCTATAAGAAGAGTCATTTAATTATGTTATGGAAGAATCAGTATTGATGCTTTATTACACTGCCTTTTATGAGATGACTTATAGACCTTACAGATTGGAATTCTATATCATTGAAATTTCTTTTCTCTCTTTCTCTCATCCTTCCATTTATATCCACATCTTTCTTCTCTATTTTGCTTTACAGCTTAAAATCGGATTGATTTTTTTTTTGCAGAAACACAAAATTTCAGTTGCTACAAAGATATGACCGATATATCATATCTTCACTGGTTCTTTAGATCGAGATAATGTGAAGTAATGAGTTGGTTATGTTAGTTTTATTAGTTCATACTATGGTGTTGGGCTGGTCTCCTAACCCTAAAACCAACGAGTCACACACTAAGCATAGCAATTTTCTCAAAATTAGTGTCAATCGAATTTTTATTCAACCTTATAGAATTAATAGAATTAATAATGAATTCATAGTTTTGAGCAAAAGAATGAGGGTTTCTCTTTTATTTATTTATTTTTTATTTTATGTTAAAAGAGAGATTTTTATCCCTGGTCTTTATTATTCCTCGTCTATAAATATCCAAATTTTGATACCTAGTCTATAAATATCCAAATTTTGATACCTAATACACCATAGATAGTTCGAACTGTATAGGAACAATAATCAATTTTAGCTCGAATGGTTTGTAGGGGAACCCTACCCTCTCGAATCCATTCGACACGTGCAATTTCTTTTCCGTCAATACGACCTGCAATTTGTACTTGAATTCCTTTTGTATCTGCTTGTTCAGTTAATTCAATAGCTTTTTCATTGCTTTTCGAAATGAAACTCTATTCTTTAATTGTCCGGCTATAAATTCTGCAAGAATATTAGGGTTCCCGTAAGGTTTTGGAATTCTTTTGATAGCAATATTAAGTTTTCGGTTCATACAATTAAACTCTTTTGTAGAGTCGTCTGTAATTCTTCGACCCCTCGCGGTCGACTTTCTAGTAAGAATTTTGGGAATCCCATAAAGATTATGACCTGGATCAGATCGATTCTTTTTGAATCTCTATACGTGCAATTCCCTCTAACGCAGAAGAAATTTTCATATTCTTTTGTACATAATTCTTGATACAATCTCTTATTTTTGATCTTCTTGTAAACCCTCCAAATAATTTTTGGTTGTGCAAACCAAAGAGAATGATGACCTTGGGTTGTACCAAGTCTGAAACCAAGTGGATTTATTTTTGTCCCATATTCCCCACTACTATACATATCAGGATACACCATAGCTGTATGCTTTTTTTCGATCTAGTTTTTTTAACGAATCTATCTCTACATATTTATCATCTAAAGATATATCTTTCATTACAATAGTTATATGACAGGTAGGTTTTTTATCGGATAACTACGACCTCGAGCCCTAGGTTTGAATTTCTTGATGGTAGTACCCTTGTTGACTTCGGCTTTACTAATGACTAAATTGGCTTCGTTGGAAGACATATTGTAACTAGCATTTGCTGCTGCAGAATAAACCAATTTAAAAATGGGATAACATGCTCGATAGGGCATAAGTTCGAGTATCATAAGTGTTTCTTCATAGGAACGTCCGCGAATTTGGTCAATTACTCTTCGTGCTTTGTCAGCGGACATAGAGATATGTTGACCTAAAGCATATACTTCTTTTTTTTCTGTCTTTTTATTTTTTAGCATAAGGTGCCTCCTACTACTGAATCATCATAAGCATCGTTATATTTATGAAATTAACGACGAGATCTATTATCGCTTTTGCATGTCCTCGGAAATTTAAAGTAGGTGCAAATTCTCCCAATTTGTGGCCGACCATACGATCTGTTATATAAATAGGCAGATGCTCCTTTCCATTGTGGATAGCAATGGTATGCCCGATCATTGTGGGGATAATGGTAGATGCTCGGGACCAAGTTACGATTATTTTTTTCTGCTTTTGTGTTAAGTTTCTCAATTTTTTTAATAAATGGTTGGCTACAAAAGGATTTTTTTAGTGAACGTGTCACAGCTTACTCCTATTTTTTTCTTTTGTTTTGTAAAGACGAAGAAAAAATTCGATTTTCTCCTATTTACTACGGCGACGAAGAATCAAATTATCACTATATTTATTCCTTTTCTACTTCTTCTTCCAAGTGCAGGATAACCCCAAGGGGTTGTGGGTTTTTACCAATTGGGGCTCTCCCTTCACCACCCCCATGGGGATGGTCTACAGGGTTCATAACTACTCCTCTTACTACAGGACGCTTACCTAGCCAACGCTTAGATCCGGCTCTACCCAAACTTTTCTGGTTCACCCCAACATTCCCCACTTGTCCGACTGTTGCTGAGCAGTTTTGGATATCAAACGGACCTCCCAGAAGGTAATTTTAATGTGGCCGATTTCCCTCTTTTGCAATCAGTTTCGCTACAGCACCCGCTGCTCTAGCTAATTGTCCACCCTTTCCAAGTGTGATTTCTATGTTATGTATGGCCGTGCCTAAGGGCATATCGGTTGAAGTAGATTCTTCTTTTGATCAATCAAAACCCTTCCCAAACTGTACAAGCTTCTTACAAAGCATACGGCTTTCTGGATGTAGATGATGATATCTATACAGATGGATCTTATATCGTACAATGACCCATGGGTGGATATATGAATCCAACCGAATGACTCATGTTATGATCTTCTACATCCTAGGTCTTCCCGTTCCGTCATCTGGCTTATGTTCTTCATGTAGCATTCAGACCGAATGACTCTATGAAATTACGTCGATACTTCCACATATTATGGGTAACGTAGGAGACATCTCTATTTTCCCCAGGAATCTTTAGAATTACCACTGCTTAGCTTTCAATTCGCCTCTGACCATCAAATGAAATGTGAATCACCCGTCCTCCTCTCTTTGAAAGAAGGGGCGCTTCCGGTTCTGTCGGTGCTTGAAACAATTTTGTCTTCTCCATATTACTATATCTCTAGAGTCAATAATTTTATATGAGGAACTACTGAACTCACTCACTTGCTGCCGTTACTCTTCAGTTTTCTGTTGAGGTCTATCCTGTAGAGGTACTCAAATTGGATCAGTGATCGATTTCTAGGTTTCGTCGTAAACCTAATTGGTTACTTCAATTACGTAAATCAATAGTTCAAACCGCACTCAAAGGTAGGGCATTTCCCATTTTATAGGAACTTCTGTACCAGAAACAATGGTATCTCCAAGTATAGCCCCTCTGGGATGTAAAATATATCTCTTCTCGCCATCCCCATAGTGTATGAGACAAATGTATGCATTTCGATTAGGGTCGTATTCTATGGTTACGATTCTACCATATATGTCTTTTCATTCCGTCGAAAATCGATTTTACGGTATAGACGCTTATGACCTCCCCTCTATGCCCTGCGGTAATGATTCCTCTGGCATTACGACCTTTACCACAACGATGTTGTCCATAGATCAAATTATTTCGTGGATTGGATTTCACTTGACTGTCTACGGTTCCATTGCGTGTGCTCGGGGTAGAAGTTTTGTATAAATGTATCGCCATGCTATTAAGTATTTTGATTTAAGTTCTTTTCTTTCTAAGAGGTGGAATAGAATAACCCGGTTGAAGCGTAATGATCATACGTCTGTAATGCATTGTATGTCCCATAATAGGTCCCATTCTTCTACCCTTTCCCGGAAGTCGATGACTATTCATAGCTATTACCTTGACACCAAAGAAGAGTTCGACCCAATGCTTTATTTCTGTCCTAGTTGATCCCGATTCGACATTAGAAGTATATTGATTTTTCCCCAATAACCGAATACTTTTGTCTGTAAATACTGCATATTTGATTCCATCCATAAATCGACTTTCTTCCCTATGAGTTCTAGTCTCAATAAGAATGCTAGTTCTTACTGTTCATATATTATGATATGAATATACCACACCAATTCGTTATGTATGGATGATGAGATTCCATTGATACAGAGCCAATTCCAATAGACTTATTGGAGGGTCCCATTGGCGTGCATCCAGTAGGAATTGAACCTACGAATTCGCCAATTATGAGTTGGGCGCTTTAACCATTCAGCCATGGATGCTTAGCGGGATCCTCGTACATAGTGAATAACCAAATTCCAATTGAAATGAAATCTTTGGGATAAATCAATGCCATTTAGGAGTAATCAATGAAAGGAAATGAATTCAAATCCTGGATTTTCGAATTGAGAGATATTGAGAGAGATCAAGAATTCTCACTATTTCTTAGATTCATGGACCCAATTCAATTCAGTGATC

>Decalepis_hamiltonii_chloroplast_contig3

AAATAATATTGTTTCCGTAAAGTAAAGATCCAGAAACAGGTTCACGAATACCATCAATATCTACTGGAGGGCAACAATGAAGGCGATAATAAATACGGAAGTTGCGGTCAATAAGGTCGGGATCATCAAAACACCAAACCATCCAATGTAAAGACGGTTTTCAGTGCTAGTTATCCAGTTACAGAAGCGACCCCACAGGCTTTCGCTTTCGCGTCTCTCTAAAATTGCAGTCATGGTAAAATCTTGATTTATTTAATCATCAGGGACTCCCAAGCGCACACATTCTCTATTTCTATTTAGATAAAATGGAGGACTTGTTATTCAACAGTATAACATGACTTATATGCTAGTGTCAACCAATATCGATCTAACTAGATTCGAATTTTTGAAAATGAAATGGATTGAAAAAATCAAATACGGATTTCTATACATACAATTTTGATATGATAGTGGGTTGCCCGGGATTCGAACCGGAACTAGTCGGATGGAGTAGATAATTTCCTTGTTAAACTAAGTAAACACCCCTCCCAAGCCGTGCTTGCAGTTTTCGTTGCACACGGCTTTCCCTATGTATACATCAGTTGCTTTCTTAGAAAAACTTTAAAGAATAGTCAGGTAATGTAACCCCTATTCGAATTACAAGCTACTTACGTGCTACATCAACATTTCAGAATTTTAGAAATAAGACTTTTGTTATCATTTTCTTTGTTATCATTTTCATTTAAGAAAATTTCCATTTAAATAGTCTCAGAAGGAATCATTGCTGATTCGCCAGATCGTTGATACAAGTAATATCAAAATACCAAATCCGACTTCTATAGACCCCCAAAAAGAGAATAAACTCTTGGGAAGGTCAACGAAAGCACTTCTTCTTCCGACATGAAGAATTCTTCCAAAAATTCGAACCTAATCTTTTCAAAAAGCCCGTACAGTACTTTTGTGTTTTCGAGCTAAAGTTTTGGCACAAGAAAGTCGAAGTATATACTTTATTCGATACAAACTCTTTTTTTGAAGATCCGCTATAATAATGGGAAAGGTTTCTGCATATACGCCCAAATCTGTCCATAATATCAGAATCTGATAAATCAGTCCGAACCGGCTTACTAATGGGGTGTCCTAATAGGTTACAAAATTTCGCTTTAGCCAATGATCCAATCATTGGAATAATTGGAACAAGGGTATCTAATTTCTTAATAGCATTATTAATTAGAAATGCATTTTCGAGCATTTGACTCCGTACGGTTGAAGGGTTTAGTCGCACAATTGAAAGATAACCCATAAAGTCAAGGGAATTTTGTACAATTGCTTTATATAGGCCCTTCGCGAGTGAAACCACAGGTCAAAATTACATTGCCAAAATTGACAAGGTAATATTTCCATTTTTTCATCAAAAGAGCCGTCCCCTTGAAAGCAGAATGCATCTTCCTTGATACCTAACATAATGCACGAAAGGATATTTGAACAAGCATAAGTTGGTCGGAAAATCCTTAGTGAGTACTTCGGCAAGATCTTCTATTTTTCCATAGAAATGGATTCGTTCAAGAAGGACCCCAAAGGATGTTGGTCGTAAATGAGAAGATTGGTTATGGAGAAAGACGAAAATGGATTCATTCACATACAAAAATTATATAACAAGAAGAATAATCTTTGATTTCTTTTTGATTTCTTTTGTTAAAAATGAAACCTGGTTTTCTTTGTAGCAATAATCTTATTGCAATTACAATACTCGCGTAGAAAGAATCGTAATAAGTGCAAAGAAGGGGCATCTTTTACGCAATAGCGAAGGGTTTGAACCAAGATTTCCAGATGAACAGGATGGGGTATTTGTATATCTAACATAAAATAAAAATGTGAAAAATTGTCCTCTAAGAAAGGAAATATTGAATGAATTGATCGTAAATTATAAGATTTGAATATCCCTTTGCGTTCTTCAGAAGATATTAATCGCATAGAAAACGGAATTTCCACAATGAAAATCCCTCTGATATCATTTTAGAATACAAATTCTTGTTGCGCCCCAAAATGGATTTTGGCTAGAATCATTAGCAGAAGTAAGAAAATGATTCTGTTGATACATTCGAGTAATTAACCGTTTCACAATAAGGAAACTAGATTTATTGTCATAACCTGGATTTTCCAACAAAATAGCTCGGTTTAAACTATGGTCATGAGCAAGTGCATAAATATACTCCTGAAAGATAAGTGGATATAAAAAGCTGGGTTCTTGAGATCTATCAAGCTGTAAATATCTTTGGATTTCTTCCATTTGAAATTCGATTTGAATCCAAGATAGACGATTTTGGGGTTAGCAAATGATACATAGTGCGATAGAGTCGAAACAGGGTATTCTAGTAAGAATAGATACCTCGGAAATAGGTAAACTTATCAACAGATTCTCTATTTCTTTTTTTTCCATTTTTGGATAAGATTATAGGATAAGGAAAGAAGGTGGTGAAAATCTTTTATTTTTTCAACCTAATCGCTCTTTTGATTTCGGAAAAACTTAATTTATCAATATACTGTTTTTTTACACACATCTCCCTTTCATAAAGGAGAATTCAAATAGTTAGGATTCATTGAAAAATGGAGAATCCCCCATGGGGAGCAACCCTTCCCGGATCGGCACTAATATTTTTTAACGTCTAATTAGATCAGGAAATAATTCAAATTCAGAACGAAAGCTCGTTGCTTTGTCTTTTAGTTAATTGAAGCCGCAGGGCCCTATCCATTTATTCATTCGACCCAACTTTATTTTGTTCCATTCCAAGACTTCAAACCGGTTTTTGTACCGACCAGGTAAGAATGAAATATTGTCAGAACTCTCCGTCAATACGACATGCTATTTTTCCATTCATTCCCTTTCAGGATCAGTCGCGGTCTTCCAAACTTTACCGATGGTATGGACGAATCCTTTGCTTCATCCAAGTGTGTAAAAGATCCTAGCCGCACTTAAAAGCCGAGTACTCTACCGTTGAGTTAGCAACCCGAAAAGAAGAAACCAACTATAAACTATAGAAGATAATCTATCGAATATAAAAATATATGTATTATGTATAATAAAAAGAAGTGCATAAATACAATCGGAATGAAAAAAAAATTAAACAAAGAAATTAGACGAAGCAATTAAAGCATTGAGATAACAAAAAAAATGGATTTTCTAATTAATTCGGAACATAAAAAATAAATAGATCAGATGAAAATATAAGAAATTGTCAGATAAAATTAGATAAAGGAAAACAAATAAAATCAAATGAAAATAGAGATTCCAAATTTATGGTCGGATCCTCTTGTTTTGTTATCCCTTTTCAATCAACCAAAAAGCTCGTGTATCAAAGAACCCGTCGTTTGAATGAAGTAAAGTAAAAACCAAACAAACCAAATAACTAGATCTAACTTCACTTATCACGATGGATTATATTTGTTCGATACACTGTTGTCAATATAAATGTTACGAAAAGAATAGAATAAGAAAGAATAGAAAAAAGAAATTCAATTGAATTTCTTTTTTCTATTCTTTCTTAGTTAAATTTCAAATCAAAATAAAAACCATCCAGTTGTCGAGAATGTCTAAATTTTCTAGGATCAAGAAAATAAGGTTTTATCGTTATAGAACACAAGATTCCACGGAAACAATGATAAATAGATAGGAATAGAGCAGGAAGGGGAGAAATAAAAGTAGAGAAAGGTTATCCAAAGTTATATACAAACCACTACCCCTTTTTGTATTTCCTTAATTGAATTTCGTTTGATTAAGATCAAGTTCCTTAAAACCCCACCCTTTTAAAATATCCTGAACAGTGCCTGTAGGTTGAGCCCCCTTTCAAGGAAATAGAGAATAGCGGGAACATTTAAATAAGTTTGATTCTTTATCGGATCATAAAACCTACTTTCTGAAGATCTTTTCCTTCTCTTCGAGATCGAACATCAATTGCAACGATTCGATAGACGACTCATTGAGATAGATGTAGATAAACAATACACCCCCTAGAAACGTATAGGAAGTTTTCTCCTCGTACGGCTCGAGAAAAAGAATTGGATTTGAAGTTTTATCTATGGTATGGATTCGAATAAATTACATAAATGACAAAAGGTTCTATAAAATCATCAAATCAATTAGACTAGGGTTTAAGTCTTTTTTCTTCTTCTTTCCTAAAATGAAAAAGAAACCATTCGTACTCATAACTCAAGTTAGATAACTCTCAAATAATTCAAAAGAGAATCTTTCATTTATTGAGTGGTCTTTACCCCCTTTTTGCTTTTTGTTTGTCTCGGTTAAAATCAATTTGGATCCTTAAGTCTGGCCCAGTTAGTGAGACGATTAAAACGGTGTTTCCTTGTTCTGGGATCCTTTATCTTTGTTTTAAATCATTGGGTTTAGGCATTACTTCGGTGCTTCTTAATCCTTTCAAAATGGCAGCAACATACCCCTTTTGGGGATTTCCTTCTATCAAAGAATCCTACGGACAATTGATTCCCGCGCGATACACTTTGGATCGAAGTGGGTTTGATTAATTCCAACAAATTTTCCTTTTGAATTGAAAACTTGCTCGAATGGGATCCCTTCGATTTCTATATCGAAGATATATTCACGAAGTTGCTCCAATTTATTGATTTGCATTAACCCTAGATTCTTATCCTGAGAAATGAATTAATACTTTCTACTCGAGCTCCATCGTGGACTATTTAGGTTCCCAACGGATGTCGAAGCCAAGAGCACCTTCATTCCTATAGAAATCGAAAATGGTGGATATAAGAAAGAATCCACAATGGATCATGTCCTTCAAGTCGCACGTTGCTTTCTACCACATCGTTTCAAACGAAGTTTTACCATAACATTTCTCTAATTTGGAACCAGTATGGAATTGATTCAATTATGGAATCATGAATAGTCATTGGTTCGTAGACATCATAATCTATACCCTTTCTTCTAGAATATAGAATAGAGATTCTATATATAGATATAGCTATAGATCGGTAAAGAATATAGCTATAAATCGGTAAAGAGGTTTCTGAAGAAGTTTTAGCAAGTCCTCTTAATTAAAAGAATTTCAATTTAATATTAATAATAGATTAACTCTAAATCTAAATAGATTGGGGTTAAATATTTCAATTTCCAAATTTTAAATCGAGGGATCAAAACCCTTTTTCACAAGAATAGAAGGATACATATACGTTAAATATTAAACATTTCCTCATTTTTTATGTATTTTGTTTAAGCTGTGTAGTTCAGCAAGATTTCGTTAATCGAATCTTACCATACATAATAGAATAGAAAGTGAATAAAAGATAATAAAAGATAGAAAACGCTCCCTTCTTAACTGTTACATAAATTTACAATTATTTATTAGTTATTAGGGCCAAACACGAAATACTTAAAAGCGAGATTCAAAGAAAATACATCGGGCGATATATAATTACATATATAATTTGATTTTGTTAATGCAATTCAGACAGACACAGAAATTTTAATACCTTCGGTTAATGTATTCGCCCCCGTGTACTACGGGGCTAATGGGGCATAAGAGAAATCAAAATGGGAATTATGATCTGGTATGCGGACTGGCTAGGTACAAACCCCAACAAGTCCAAATTAAGTTGCTCCTATTTTTATTTTAGTCTAACCCTTGGGAGAATTAAGAATTAATCCTATTGACTTTGAATGAATTTTATTAGTACCAAAATAGTTAGAATTAAGAAATCTATATAAAAATTCATAAAAAAGTTAGTTTACGGATGGGGAATAATAGATAGGATCCTTAAAAATTCAAATATTGATAAAATAGAAAATCAATATGTGACGGAATGTTTTTCTAAATAACTAACTTCCAGAAACAAGATGGGATTGGACATAGGATGAAAAGATCCCTTTTTGAATTCAAACTCTGTTCCCAATTTACCCAGATCAGAAATAGAGTCAATTACATCTACGTGTCATTTGAACTCGATTCAATTCAGTTTGTGTAAAAAGATATGATTCATGCATAAAAGATAAAAATCAGAAACAAGAAAGAAATTCCAGCTAACATTAGACTTTACCCATTCACCAAAATCTTTATTCTATTCCAACATAATGAATATGCTCTGGGACGGAAGGATTCGAACCTCCGAATGGCGGGACCAAAACCCGTTGCCTTACCACTTGGCCACGCCCCATTTAGATTTCTATTGGATACTACTAAAGTATAATATGGGTATTCATTATTTGTCAACTCTAATCTAATTTATTCTATAGATATTTAGATCGTTACTAGGATTTTCTTAAATATAGAATTAAACTTAATTTATTGATCATTAGATATAATTCAATTAAGATATTGTATGAAAGTATGATTTCCTCTATTCTCTTTTGAGAATTGGAGGATTTTGATTGGGTGAGTTCAAAAGAAAAGAAGGATTTTTTGTCTACCTAAATTTTCCCCTTTTTCCTTATATCAATAACTCAATCAAAATGCAATTATCTCCAAGAACAAAATGTCTGTTATGCTTAATATCTTTAGTTTGATCTGTATCTGTCTTAATTCTGCCTTTTATTCAAGTAGTTTTTCTTCGGAAAATTGCCCGAGGCCTATGCTTTTTGAACCCAATCGTAGATGTTATGCCAGTCATACCCGTGTTCTTTTTCTCTTAGCTTTTGTTTGGCAAGCTGCTGTAAGTTTTCGATAAGATCCTTAATCTTAAATATTATTTATATTTAAATTTAATAATTTCTTACTAATTTCCTAGAAAGTTCATGATTTTTCTAGAAAGAGACCCGGTTCTTGATATCCAAATAGGATATGTGGTAGAAAACGGAGAATCTATTCTCTTTTTTTAATTATCTTGGAGATTGTGTAATGCTTACTCTCAAACTCTTCGTTTACACAGTAGTGATATTTTTGTTTCTCTCTTCATCTTTGGATTCCTATCTAATGATCCTGGACGTAATCCTGGGCGTGAAGAATAGGGGTTTTCTTTTCTATTTTCTTAGGATTTTAAGAACAAAGGATTTGCCAAATTTGAAAAAAAAAAAATCATCAAAGGAAGCGGAAGAGAGGATTCGAACCCTCGGTACGAATAACTCGTACAACGGATTAGCAATCCGCCGCTTTAGTCCACTCAGCCATCTCTCCCAATTGAAAATTGGGTTAGATTACATAGAATAAGGAGTATTTCTTTCTCTATTATATAGATATGTTAGATATGTACAATTTTTATCAGTAATTTCTTTTCGATAAATAAAGGAAAAGGCCCGAAAGTGCCAACAATATAAAAGACCCTTTCGACTTAAGGTTATTTTGTTCGAAAGACCCTTCTTATTGACACGGCCTGGCCTGGTCAGTACCTGTACCTAGCCGGGCCTTTTTTGTTCCAACTAATTAACTAATTATAGAAAAATAATGATGATTTTTCATTTATCTGATTTGAAAACAAAAATGCTTAGGATTATATATTATTATTCTATTTAGTATTATATTATTATATAAAGTAAATAGGAAATATTTAAGCACGAACAAAAAAGCAGAAGGACTACTTCCATCCGCGAAAACGAAAAAAGGGGTCAATAGTCTAACAATACTCTAAATTTGAAAAATTTTATGATTTTTGATGATCCTATCTTTATTATATTATTATACCTCATTTGCCGGTTCGACAAAAGGTCCAGTTGTATACAATAATCGCATTGTAGCGGGTATAGTTTAGTGGTAAAAGTGTGATTCGTTTTTAACCCTTTGATAGTTAAAGGGTCTTTGTTTTCGGTTTGATTCGTATTCCGACCAAAAACTTTATTTGGAAAAAAAAGATTTAATCCTTTACCTCTCAATCGCGGATTCGAGAAAAATATACATTCTCGTGATTTGTATCCAAGGATCACTTAGAAAGTGAGAAATTGGATTATGAAATTACGAAACATAATTTTGAATTGGATTAATACTTCCAATTGAATAAGTATGAGTAAAGGATCCATGGTGAAGATAAAAAGTAGGTTTCTAATCGTAACTAAATCTTCAATTTTTGCTTTACAAATAAAAAATTGAATTAAAATAGCTATTAAACGATGACTCTGGTTTACTAGAGACATCGACCTGTTGTTTTAGCTCGGTGGAAACAAAATCCCTTTCCTCAGGACCGTCTCAAATAAAAATAGAGAACGAAGTAACTAGAAAGATTGTTAAAATTACTCTCTTCTAGAGGGATCATCTAGAAAGCGATTAGTTTTGTCTGTAGTCAGACAAAAGTTGACATAGATGTTATGGGTAGAATTTTTTTGTAATTTTGTTCACATCCATATCCATAAAGGAGCCGAATGAAATCAAAGTTTCATGTTCGGTTTTGAATTAGAGACGTTCAAAATGCTGAATCGACGTCGACTATAACCCCTAGCCTTCCAAGCTAACGATGCGGGTTCGATTCCCGCTACCCGCTTTCTATTCTTTTATTCGAAATTGAAATTTTATATATTCTAATATTCTAGACTTAATGCATCATTTAATATACAGTTTCAAAAATTATCTCACATACAATCCGATTTTTTTTTCAGTGAAGAGGTGGGGAAAGTCAAAATACGAAAAATCGGAATGAAAAGCGTCCATTGTCTAATGGATAGGACAGAGGTCTTCTAAACCTTTGGTATAGGTTCAAATCCTATTGGACGCAAATTTTTCCATATATATATTTTTTAGATTTTGATATCACGAAAGACTTTTCGAGTAACTTGAATTTGAGACCCTTAATTACATTACCTCTTTAAGATAAAAGAAAGTAAGTGATCCATTTTTTATGCTTATTCCTGAAGTAGAAAACGGTCCATTTGTTCCTGAATAGCTTCTTTCAAAAGGGCTTCCGCTTCCTCGGTAAACGTCTTGGTAGAAGAGATGATTTCTTGGAACTGAGGTTTGTTAGTTTTACGTAAGTCCGTAACTCAACAAGAAATTTCCTTACCTGTCCAATTTCTAATGAATCAAGATAACCGTTTGTTCCGGTATAAATAGTCATTACCTGTTCTTCCACCGCGAGAGGAGCTGATTGCGATTGTTTAAGCAATTCACGCAATCGTTGACCTCTTGCCAATTGATTCTGAGTCGCTTTATCGAGATCAGAAGAAAATTGTGCAAAGGCTTCTAATTCTGCGAATTGCGCCAGTTCCAGCTTTAATTTACCAGCTACTTGTTTCATGGCTTTAATTTGAGCTGCGGACCCCACCCTGGAAACAGAGATACCCACATTAATAGCGGGCCTAATTCCAGCATTGAATAGGTCGGCGGATAAGAATATTTGGCCATCCGTAATGGAAATTACATTAGTAGGAATATAAGCCGAAACATCTCCCGATTGAGTTTCAACTATTGGTAAGGCGGTCATACTTCCTTCACCTAAACTAGAACTTGATTTAGCGGCTCTTTCCAAAAGGCGTGAATGTAAATAAAAACATCCCTGGATAAGCTTCGCGCCCAGGCGGTCTTCGTAATAGAAGAGACATTTGACGATAAGCTTGTGCTTGTTTTGAGGGATCATCATAAATGATTGAAGTGTGTCGTTCACGATACATAAAATATTCAGCCAGAGCTGCTCCTGTATAAGGGGCGAGATATTGTAATGTAGCAGGGGAATCCGCCGTTTCGGCTACCACAATAGTGTATTCCATCGCCCCCTTTCCTGTAAAACAGTTACTACCTGAGCCACAGAAGATGCTTTTTGACCTATAGCTACATAAACACATATTACATTTTGACCTTGTTGGTTGAGAATCGTATCCGTGGCTACTGCTGTTTTACCGGTCTGTCTGTCCCCAATAATTAATTCTCGCTGGCCCCGCCCTATAGGAATCATCGAATCAATAGCAATAAGTCCGGTTTGAAGAGGCTCGTATACGGAACGCCGCGAAATAATACCTGGGGCAGGAGATTCAATTAATCGAGATTCAGAAGCTGAAATTTCGCCTCTACCATCAATAGGTTTAGCTAGGGCGTTTATAACACGGCCTAAGTAGGCCTCACTCACCGGAATCTGAGCAATTCTTCCTGTTGCTTTTACCGAACTTCCTTCTTGTATCATCAAACCATCACCCATTAATACAACACCAACATTATTTGATTCTAAATTCAGAGCAATACCTACCGTTCCTTCTTCAAATTCAACCAATTCGCCCGCCATTACTTCATCAAGACCATGAATACGAGCAATGCCATCGCCTACTTGAAGTACGCTACCAGTATTTACAATCTTTACTTCTCTATTATATTGTTCAATCCGTTCACGAATAATATTACTAATTTCGTCAGCTCGAATGGTTACCATGAGTATTTCTTAATTTTTTTGAAAGAAAAAAAAAAAATAATGCCTACAGTAGAAAAGACTAATCAGTTATTTCTTTCATTGACCCTAACATGCCAATATTGGCACTAATAGTACGTAAATGTAACTCGTTATTTAAACAACTATTCAGAGTTCCTAGGGCTCCCCGTAAGGCTTGTTGGAAAACCCGCTGTCGGACTTGATTAATCGCCCTTTGTTGTTCAAATTGAATAGTTTCATTTTGTAATTTTCTAATTGTTCTAAAGTCTTAGAAGTTGAATTAATTAAATTCAACTTTTCTCGCTCTATCTCAGAGTATCCATTTACTCGAAACTGATCTGCTTCAATTTCCACTTTCCGTAAGCGAGCCCGGGCTTTTCCAGCTGTTCAATGGCGCCTCCACGGAGTTCTTCTGAGTTTCGAATAGTATTCAAGATTCTCTGTTTTCGATTATCTAATAAATCGCTTAATGAAAGTGGATTATCTTTCCATTTATTCCAAAGCTTCCATAATCCCTTCCCGAACCAAACATGAATCTTTCGACTCATTTGGCTCTCCTACTCAATTACTTAAGAAAAATTCTCATATCTTTTATGAATGTAATGAGCCTATCCTCTCTTCTTTGTTTGTTCATATTTTCCTATCCAAAAAATATATAAACTAATGCCAAATCAAAATATTCAGAGGACTCTTCTGACAAAAATATGTAATTGTCAACAAAGTTGTTTCTTTTTTTTCTTCAAATCCAAAAACTCGTCTTACTTATACATAGGTCGTCGATTCAGCATTGGATAAAAGGGGAAATACCCATTTTACAATAACAATAAGGGTTCAAAACTATTTTATGAATAGGAGTGTTCTCTATCCCTAAAATATTCATTTTGAACCATCTCTATATTACCAATATTAACATAGAGGTTGAAAGAGTACCGCGCTGCGTCTAGACTTCAAACAGTTTGCTTTAACCATATTCATATTAATGGCCACACATTGTTGGTTGATAGAGAATCAAAGTAAATTTACCAATGAATCACGAAATGCTATGGTTCTTATCCATAATCTCTTAATTTATTCCGAAGTCATTCGTGAGATCGTGCACCTTTCTTTTCTAGTTATAACGAAAAGGTACAGCTGGTTGGATCCAGCATATTCTTGAAATAAACAACCCGCACACACTCCCTTTCCAAAAAGATCAATACACCAAGCACTACACTTAGATTTATTAGATTTGTTGATAAAATATCGGTATTAAACCCGAAACTCCCGGCGGATGGCCAATGGCCCAAAGAAACGAAAGAATCGGTTACATTTTTCATATGATCTCCTATAGACTAAATAGATAGACTAAAAGAATAGAGTTCTTTTGTATCACTTCGCCCCTCTTTTTATTTATTTCCTATTTTAAAAAGTATTCGATTTATGTGAACTATCCCCTTGCGTCAATCCTTAGTTTCCTTGGACTCCGAGGGGAAGGATGAAAGGGAGTGGGTATACTAATCCCTCATCCACAAATCAACCCTTCCCACAGGTTATTTTGTCAACGAATAAGTAATTGTAGGAGTAAAATCTTAATAGAATTCGAAAAGGAAACAATAAGTCCAAGGCTATAAAATAGGGATTTTTCATATTAAACAAAAGGATTCGCAAACAAAAGCGCTAATGCTACAACCAGTCCATAGATTGTTAAAGCTTCCATAAAAGCTAGACTAAGCAATAGAGTACCTCGTATTTTTCCTCTGCCTCAGGCTGTCTCATGATACCCTCTACAGCTTGACCCGCAGCAGTCCCTTGACCAACTCCGGGTCCAATAGAAGCAAGCCCCACGGCCAACCCAGCAGCAATAACGGAAGCGGCAGAAATCAGTGGATTCATGATAAGTTCCTCGTACCAAAAAAGAAATGGTTAATGATACAATCAACCACCAAATTATGACTTAATAATTCTGTCACTAGGATTCGAAGTAAATAAGAACTTCGAGTTGAAGTAATAGAAGTAATATAGAATATAATAGTATTAGTGAATCATCCGAACTACTTTTGAGTTTCTGTTTCTATCCACAGAGTCTTTGTGAATCCATACGACTTTCGATCTTCCATTTCTTGGTTCCGAACTCTTATTTTCGTCCACCCTTTTCTTAATTTCATCTGTTTATTTATTCAATTCACAGTCACAAGGAGACGGAAAGGGAAAGGCTTCCATTGGTATTAGAATCCCTGCCAAAATTCATAGGAGTGGGGTGGCAAATAAAATATATCGTTAGTTCATATAGAATCAGTCAATATCTAATATCACATATACATGTCTTTCTTCCATAACGTAAACCAAGCACTCATCTTAGATTCCATCGGATTCGAGAATCAATTATCGAAATATCTACAAGAGTTTACTTATTTATAGCCATTCAATTACATATACCTACCCTCTCCAAACCAACCCATTTTTTATGAATTACGTTTTTGTCTAAATTCCTTTTTTTTTCTCTTACTTTTCTTTATCATTATTCCAATGTATCCAATCTAAATGGACAAATTGGTTAGTCCAAATCATTGCATAGAAATATTATTATTTGATTGATCTAAGTTCATACAATTTGTTATTTATTATATTATCGTTTGGTCAATCGTTGAATAAAACAACTGAAATGGGAATCCTTCGCCCTTTTTTATTTATTATTTAGTTCATTTTTATTTTATTTAATATTATTATTAATCACATTTCTTTTATTTAAATTTCATATTTTTGTTATTTAAATTAAATATTACTATTTAATCACACGTCCTAAAGATATACTAAAGGCACTCGTATTGATATTGAATATTCAAATAATAAAAGAATTTGAATTGAACTATTGAATAATGAGATAGAAATCGAATATTCGTTGAGGAGAGGTATGATATTAAGTGAACGAAAGGGAACTTTAGGAAAAAGATCTTTTCGATCTCTTTCCTTTTTACACCTATCTAATATCGTAATTTTTTGCTATTACTCTATATCGTATATGGCCTAGTTGCATATTCCTAAGTAAATTTTATTGAACCAAAGACCTCTTAGTTTGAAAAAACAATTTCAATGGTGGCCCTCCATGGATTCGCCGATATAAGCCGCGGCTAAAGTTGCAAAAATAAGAGCTTGAATACCACTTGTAAATAATCCAAGGAACATAACTGGTATAGGAACCACTGAAGGTACTAAAGAAACAAGAACAACAACGACTAATTCATCAGCTAAGATATTCCCGAAAAGCCGAAAACTTAGCGATAGGGGTTTTGTGAAATCTTCTAAGATGTTAATGGGTAAAAGGATTGGAGTTGGTTGAATATATTTCCCGAAATAACCTAATCCTTTTTTGTAAGACCCGCATAGAAATAGGCCACTGATGTGAGTAAAGCCAAAGCAACAGTAGTATTTATATCATTCGTGGGTGCGGCTAACTCCCCATGAGGTAATTGTATGATTTTCCAAGGTAAAAGAGCTCCTGACCAATTAGAAACAAAAATAAATAAAAACATAGTTCCAATAAAAGGAACCCAAGGACCATATTCTTCTCCAATTTGAGTTTTACTAACATCTCGAATGAATTCAAGAACATATTCGAAGAAATTCTGACCCCCACTCGGAATGGTTTGCGGGTTCCGAACAGCTACGGCGGCCGAACCTAATAAGATAGCAATTACAACCCAAGAAGTAATAAGTACTTGGCCGTGGACTTGGAAGCCCCTATTTGCCAATAGAAATGCTGGCCTACTTCCACATCGGATACATCATATAACCCTTTTAGTGTGTTTGCTAGAACATTCATATTGCCCTCTGAAAAAATCGAACTTTGAATTTGATTCAACCATCTCTTTGCCCACTTGAATCGGATATTTTGAATACCAACTAATAGTACAATTTTGAATAATAACTAATCACATGGTCTGCCCAGTTTTTTATCTCTTTTTGATTTTTATGAAATTCAGAAACAGTAACAGAGTCCACAAATCTATATCTATAGTATTTTCGAAGTAAATTCTTTATCTTATTATTAATCAAGGATTTCGTATATAGCTAGAACGACCCTCACAAATTGCAAACACTAATTTGTTAAGAATTAATCGGATTGAAGATATAGCGTCATCATTAGCTGGAATCGAAATATCTGCTAAATCAGGGTCACAATTTGTATCGATTAAACAAATTGTTGGAATTCCCAAAGTGATACACTCTCGTAGCGCCGTATATTCTTCGTGCTGATCGACGATGATTACAATATCAGGTAACCCTGTCATATATTTAATTCCGCCCAGATATGTTTGCAAACGAGATAATTGTCTTTTCAACATAGCTGCATCTCTTTTGGAAGACGGTTGAGTCTCCCTGTTTTTGTTCCATTCTCAAGTCCCTGAACTTATGAAGTCTCGTTTCTGTAGTGGACCAATTCGTTAACATGCCGCCGAGCCACTTTTTATTAACATAATGACACCGGGCCTTTATTGCAGCCCATGCTACTGAATCAGCTGCTTTATTTTTGGTACCAACAATTAAGAATTGTTTTCCTCTACTTGCTGCATCAAAACCAAATCACAAGCTTCTGATAAAAACGAGCAGTTCGAGTAAGATTTGTAATATGAATACCCTTACGCCTTGCAGAGATATAAGGTCCCATTTTAGGATTCCATTTACTAGTACCATGGCCAAAATGAACTCCTGCCCCCAGCATTTCTTCTAAATTTATGTTCCAATATCTTCTTGTCATTTCTCCCCCACCCCCTTACCTTTAAAAAAAGAGAGGAAGAGCCCTGAACTGAAATAAAAATTGTTCCAAGGGAACCTTATGCTCTACCGTAGATTGGCTATAGATACACAACCCAAGCCATTATTCTTTTCTATTCATTAGGCTTTTATTACTAAATCAAATGACTGCACCAAATCAAAGAAAGTAGTGAAAGGAATGACTCCTTCTTAGTAATCAGTAAATACTATAAATGATTGTTCTGATGTATCGTGGAAATTCTCTCTTTCAAAGGGAGGAATCAACTAATTTTCTCTGGTAAAACAAAATATCTCTCATTTTCACCTCGAATAGATTCTTTTTTGGTTTCCAAAGGACTCTTGTTATGTTGTTTTGAAGGGTGCACTAATCCGGTCCCGACAGGTATCATACCCCCAAAACTACGTTCTCTTTCAGACCTTTCAACCAATCGATACGCCCCCGGAGAGCTGCCTTTGCTAAAACCCGAGCAGTTTCTTGAAAACTTGCTTCAGATATGAAACTTTGGGTATTGAGAGATGCTTTTGTTATTCCCAATAAGATGGCTCGGTAACAGATCGCTTCTTCCAAAGCGCGTCCCATTCGTTCTGCTCGCAACAATCCAATTAGTTCTCCGGGTGAAAAAACATTAGACATTCCGTCTTCTGAAACCAACACTTTTGATGTTATTTGACGTACAATAATTTCTATATGCCTATTATGAATCTGCACCCCTGGGATCGATAAACCTTTTGGATCTTATTGACCAAAGAGATACGACTTTGCACTATAGTTAGCTCAGCACTAATGAAGAATCCCCAAGGAATTCCAAGAATTCTTGTTATACGTTCGTTCCAACCTTCAATCCTCTTTTCTAGATTCATCGATATTGAATCAATCGAGCGCACTTCTAACACTTGTTCCACTTTTGGAAGACCCTGCGTTATGTCCCCAGATCTCGACTTTTCATATATAAATGTAACTAATGTATCTCCTTGATAAAGGATTTCCCCATAATGGCCATGAACGGTTGCTCCTGGGTGGCCAAATAAGGCTTAGCTGATCGTATTACTACGGAATCGGCTTGAACAAAGATAACTTGACCCGATTTTAGGTGTGGTCCGTTTTGGCTACACACATTTTCACAAATAAACTGCCCAAGGCTAATTATTGTAGACGTCTCTTCACAATAATTGTGACGGAGAAAATACCAATTCAAATTGAATGGATTGAAAATAATGTTACTGCATGGGTCGGGATTATAAAATTTCCCACCTTCATCCATTGAATAATATTTAATTACTTGAAAAGTCCGTTTGAAATTGTCAAATTGCAAATAGTTAGTTAACAAGATTTGATTGTGAGTTATTAAGTGAGAAAATAAATAAAAATTCTCAATTGGGGGACTGATCCTAAAGGGCCCAACGAATTCCTAATTGGAATTGGGGATCCTTTTGATGAATTTGATAAATTGATTCTTTTGTTCCATTGTGATATTTTAGATCGTTGAATGGACCCATCCGAGAACAGTTGGACGATAACAAAATTATCAATGGTTGGAATTCCTTATTTCTATTCAACAACGTATGAATAGTTCCTTGATTGGGGTTAAGGAATTGTTGAATTCTTGCCTTTGAATATGAATAGGAATATATGGAGGAAAACGGATTGATATTGGTGTAATCTGATACATTATCAGAGAGCAATCCTGAACCTGAGGGCTCATTCCTTTTTCCGATATAGGAAATCAGGGATTTCGCCAAGTCGATTCTTAGGAAATGTCGAATCAAACCATTTATCCTTATTTCAACAAAAGAAGCCCGGGCTTCTTCGCCAGAAGAACTTTTTTGTCTTTGTCCCAATTCAATACTAAACAAGTCCGAACTAATTGAATAGTTGTGTCATAAATTCCTCGAATTGGTTTGCCGTTTCCATTTCCATAAAGGATATAATTGATAACTCGAAGTTGCACAATATCTCTTTCCTGCAACATATCGGGGGAAAAGTGTTGCTAAATTTTGACCGTCCGTTATTTCATATGTGACGACAGGTCGAACCAAAACAAAATGCTTTTTTTGCTAGGTGTAATCCGTTGGACATAGATCCAATTTTTCCATTTTGAAATTCCTTGGAATTTCCTTTTCCCTTCCTGGGGTATCAAAACGCCGCTATGCCGGGATATCGTATCTATTTCTCCAGGAAAATGGATATCTCCAGAAAATATTTTAAGTTCGATTTTTTTTTCTCTCCACGGACCAACCCCCTACCCGGCTTCTTAGATTTAAAGTGATTTGTGTATCTACCCCAATGATACTATTGTTCCGTACCATTATGGAAGAAGATCCAGGTAAGATATGAACTTCCTCGGGAATGAAAAAAATCGATCTACTTTCATTTGGTATTTTGGCCTAAATTCCTTGACTCCTCGATACTCAATCGAATCTTCCTTTTAAGGATTGAATGCATTTCTATAGTCCCATATTTAGTAATTCCCGAACTCTTTCTTCTATACCGAGGATCGTCGAAATAAGAAAGAATACTATTTCTACGGAAAATACCATTTATGGGGAGTTCAATCGAGATACCCGAAGGGACGTTAGTTCGTTCTCGCAGGATTGGAGTGGAATAATAAATCTATTTCTTCGCCTCTTTGACAATAAATCTGAATTCTCGCGAAGAATGGCCGGATATATGAGATTACAATGAGCAGTACATATGACTTGATTAAGGTGTGAATAATCAGGAATGCTATCTTTTTTTACCATAAAAATCCGAACTAAAGAATTTGTGTCTCTTAGTTACCGAGAGGTTAGAAGTATATCTTTGCTTGACAGAAAGAGAATGCGCGCTCGTTTGATCTTGATCCTTGTGAAGCGAAAGGGAGACTGGACTGGATCTGCACGGCCCTCCTAATAATATCCATAAATGACTTGTTTTTGGTAATAGATGCACATTACCATATGTAAATTCAGGTGCATGATCGACATAGGTACTCCAGTGCATTTCCCCGTCTGAGTCGGAATAAATATGTTTTCGAACCTTCTCTTTAAAATTCAAAGTGGGCGTTCCCGCGCGAATCTCAGCAATCACTTGCTCTGATTCTACATATTGATCATTTTGAACTAAAAGAAAACTTTTGGATGGAATATTCACATTATGTATAATATCTTCACTCTCAATAGTTACATACAAGTTTATAGAACATAGAAAGGCAGGATGTCCATGACGTGTACGTGTCGGATGAACGAAATCCTCATTAAATTTGATTTTTCCATTAGAGGGCGCTCTCACATGTTCTGCAGTACCTCCCGTGAATACTCCGCCGGTATGAAAAGTTCTTAATGTTAATTGAGTACCCGGTTCTCCAATCGATTGACCTGCAATAATACCTACAGCCTCCCCAGCTCAACCAGGTCGCCATGAGTGGGACTCCGCCCATAGCATAATCGACAGATCCAAGATGTACTCCTACAGGTAAAGGGAGTTCGAATAGATATTGGTTGTACTCGGAAGGTTATGAATCGATTTACAAGTTCAATCCCGATGTCTGTATTTCTAGTGGCAATACAACGCGGACCCATATATATATCATCCGCTAATACACGACCAATTAATGTTTGAATAAAGATCCTTTCCGGCATTATCCCATTTCGAGGACTCACAGAAATACTCCGGGGGTGCCACAGTCTGTTCGGCGTACAACAATGTGTTGAACTACTTCAACAAGTCTGCGCGTGAGATATCCAGCATCTGATGTTCGTACAGCAGTATCCACAACCCCTTTACGGGCTCCGTAGCAAGAAATGATATATTCTGTTAAAGAGAGCCCTTCGCGTAAATTGCTTTGAATGGGTAAATCAATCATTTGTCCTTGAGGATCCGACATTAAGCCTCTCATACCTACTAATTGATGTACCTGAGATGCATTTCCTCTAGCTCCCGAAAAGACATTATATGAACTGGATTAAAAGGGTCAGTCATCCTAAAATTAGGATTCATTTCTTGTCGCAAATATTCACTTGTAGCATACCATATTTCAATGGATTGGCGTAATTTTTCTACTGCGTGTACATTCCCATAATGGTGGTGTTTTTCTAAAATCAAACTTTGTTGTTCAGCATCTTGAACTAGCCACCCTTTCGAAGGTATTGTTAAAAGATCATCAATTCCTAATGAAATGGATGTAGCGGTAGCTTGTTGGAAACCCAGGGTTTTTACTTGATCCAGGATGTGTGATGTATATGCCATTCCGAAGTGATCTATTAATCGACTAATAAGTCGTTTCATGGCAGTTCCGTCTATCGCTTTATTGTGAAAGACCAGATTAGCCCGTTCTGCCATAAGTACCTCCATATTCTGCTGAGTAGGATTCGACAAACAATGGGTTTGGGTCCGTGATTGGAAAACTTCCTTTTCTTGATCTCGATTAGCGTAGAAATTCGGGAACTATAATCCCGGAGAGACCAGAATTCCTACCGGTATTATACAATTACTTAGGTATCGGCATGAGAAAATCCCTGTAAGGCTTCGTCGATTTCTCGATAAAGAGAAATATGACCAACCGTGGTTCGAATGTATATAAAAAATTTCTTTTTATACTTCTTACTATTAGATAGTGTCCATAAATTTCATAGTAAGTTCCTAAAGATTCATAGTGAACTTCGATGGGAGTTTCTCTTGAAGCAATAAGGCGTTGATCTAGTCGCCAACGGATCCACAAAGGGCTATCTAAATTGATTCGTTTTTGCCGATAAGCCCCAATTGCATCATAGGAATTGCAAAAAAGGTTCTTTCGTATAATCATAGTTATTATTATCACTTCTTTGATTTTGATAGTTTCCGCGATTACCTGGATTATATCTATTTACACAAATACCTCGACGATTTCCGCTCGTTAATACATAGAGTCCCATAAGCATATCTTGAGTTGGTACGGAAATGGGATCCCCAATAGCTGGAGACAAAAGATTCATATGAGAAAACATAAGTAAACGGGCCTCCGCTTGGGCCTCAAAAGATAAAGGTACATGAACAGCCATTTGATCCCCATCAAAATCTGCATTGAATCCCTTACAAACTAATGGATGTAAACAAATAGCACGCCCCTCCACTAAAATGGGCTGGAATGCCTGTATGCCTAATCTATGCAGAGTGGGCGCTCTATTCAACAATACGGGATGTCCCTCCATAACTTCCTGAAGGATTTCCCATACAATGGGTTCTTTTTCCCTAATTTTACTCTTAGCAACTCCTATGTTCGAAGCAAGATGTTGTCTAATTAGACCACGAATTACAAATGTCTGGAAAAGCTCTATTGCTATTTCTCGGGGCAATCCACATCGATGTAATGAAAGTGAAGGACCCACAACAATAACGGAACGCCCTGAATAATCGACCCGTTTGCCAAGCAGAGTCTCACGAAATCTTCCCTCTTTGCCTTCAATTACATCTGAAAATGACTTGTAAACTTTATTATGACCATCCTTCATTGGTTGGCCCCGAATTCCATTATCAAGAAGCGTATCCACGGCTTCTTGTACTAATTTTCCTGACACATTAGTAATTCTCCCGGCGTAGATCTACTTGTTGTTAATAGATCGGTAAGAGTATTGTTCCGATAAATAACTCTTCTATAGAGTTCATTAATATCCGAGCTCATTAGTTTACCCCCGTCTATCTGAATGATCGGTCTCAACTCCGGGGAAGAACTGGTAATAGAGACAAAACCATCCATTCTGGTTCTATATTTGTTCGAATAAAATGCTTAGCCAATTCCATACGTCTAACCACAAAATCCTTTCTTCTTCCAACTTTTCGATCTTCCCATTCATTCCCTGTGAGACCTTCTTCCCTAATTCTTTCCACTCTACTAATGAATTTTCTAGAATAATTCGTAAATCTAGATCGGCTAATTGTTCTCGGATAGCACCCGCACCAGTAGATATTTCTCGATTGCGAAATGTATCGAAGCCTTGGGTAGTAAAAAAGCGGGATGCTGTATTTCCAAGATTGGATTTCATATTCGAATGAACCTCGTAATCGTAAGAAAGTGGGTTTTTAGTTATTGGCCTAGCAAAAGAAAAATTGGGATAGGATCCTATAGGATCTCCCCTTCAAAACCGGACGTGAAAATTTTCCTTCATCCGGCTCAAGTAAGTACACCAAATAAAGAGAGGGGTTCTCACGTTCAAATTCTAGAAAACCCAAACAAAACAAAAGATCTACTCCTTACTCAAGTTCCCAGTGAAGACCAAGCAAGATTTCATGGATTCCGTCTTTCTTTTATTTCTATTTTCTTAATTTTTATTTTTACAACATAAATGAAAAATTGTAAAATTCTTGAGTAGTCTACTTCTCTTCGAATGATGAATCCCTTAAAAGAATTAAGGAGTACTTTGTAATTCATAAGAGATTTCCTTGTCTATGTATTGTTCCATTAGATCTTTTAGGCCCCGACTTCACCTCGACGGTTATCCCACGATGCCCTTAAAGTCTATACGCGATGAATAGACTCCTATAACCATATGACATATTTGCATTTGCTTACTTGAAATTTCTTTCCAAAGGGAAATAGTGAATTCCGCAAAACAAAAAGTCTTTTTACGAGGTACAAATAGAAATTTCTATTTCTATTTATTTGTTGTTACGAAATCGACCATAGATAAATTCCCTTTTTATTTATTTGGGAGTATTGATTACACCCTATTCTGAGCTTCATGTTACTCGTCCCAAGCGACATGTCAGGTCCAGGGCATCCCAATTTGATTAGGATGACAGTTTCTCATTCCGAATCTGTAAAATCAGAATTTCGATCAAATCACACATCGCAGTATACCAGCCCTTCTAATTCTTTAAGGGATTTATCTAAAAGATTCGCAATATAACTAGGAAGACGTTTTAAATACCACACATGGGTTACTGGGCATGCGAGTTTGATGTAGCCCATTTGATACCTTCGTATCCGAGAATCAACAAATTCGACTCCGCATTGTTCACAAAATTTCGGGCCTTCCTTTTCATTTCCGATTACTCGATAATTTCCACAAGCACAAATTCCACTTTTATAGGTCCGAAAATTCTTTCACAAAATAATCCATCCTTTTCTGGTTTATTGGTTTTGTAATGAAAAGTATAGGGCTTTGTCACCTCTCCAACTATCTCTCCATTAGGCAGGATTTTAGTGGCCCAGGCGCTTATTTGTTGAGGAGAAACTGATCCAATTCGGAGTTGTTGATGTTTATACGAATCGATCATCGAAGAAAAATTCTGGCGCGTTTCCATTAAGCTTCCTTTCTATTAATCTCGAAGTTCTTTTCAGATACAAGGAAATGATTCAGTTCCAGAGCTAAAGATCGTAGTTCTCGAACGAGTAATCGAAAAGATTCTGGAGCATCCTCGGGATTCGGTATTGTTCCTCCAACGATCGTAGTCCCAAGTACTTCCTGGCGAGCTCTAATATGATCAGATTTATAAGTAAGCATTTCTTGTAAAATATGAGCAACACCAAATCCTTCTAGAGCCCAAACCTCCATTTCTCCTACCCGTTGTCCCCTTGCTTTGCCCTTCCTCTAAGGGGTTGTTGTGTAACAAGCGCATAATGTCCACTGGAACGTCCGTGGATTTTATCATCAACTTGATGAATTAATTTTAAGATATAAGACTTTCCTATTAGAACGGGTTGTTCAAAAGGATTCCCCGTCCTTCCATCAAATATTCGGCTTTTCCTGGATACTCGGGTTCAAATACCCACGGATTCGCGGTTTGCTTACTAGCTTGATATAATTCAGAAAACACTAGTTTTCTCGAAGCTTCTTGTTCATATCTCTCATCAAAAGGCGCTATTCGATAATGTCTGTCTAGCAGACCCCCGCTAACAAGTGAACATTCAAATATCTGTCCTACATTCATTCGTGAAGGTACTCTAGCGGGTTGAAGACCATATCAACAGGTCTTCCGTCTTGCAAATAAGGCATATCTTGTCTAGGCAAAATTTTGGAAATGATACCTTTATTTCCGTGTCTTCCGGCTACTTTATCGCCTACTTTGATTTCGCGTTTCTGTGAAATATATACACGAATCGTTTCTGGATTATAACTAGAACCCCCTTTTCTGGATCCACCTCACATCAATAACCCGACCCCTACCGCCTATAGGTAGTTTTAGACAAGTTTCTTTTGAAGTAGATACCTGAATGCCAAGTATGGCTCGTAACAATCTATCTTCCGGGGCATATGATGATTCTTTTACCATCTGGGGTGTTAATTTACCTACTAAAATATCGCCTGTCTCTACCCAAGATCCCAGCATCACAATTCCTTTTTGTCTAAATTGCGGAGTAAATGGGCTTCTAAATGCGGTATTTCGTTAGTGACCCTTTCGGGGCCTTGGCTTGTCAGATGAGTTTGAATTTCATATTTCCGTATGTGAAAAGAAGTATAAATATCTTCATATACCAAACGCTCGCTAATGAGTACTGCATCCTCAAAATTGTAACCTTCCCATGGCATATAAGCTACTAATACGTTTTTCCCCAAAGAAAGTTCACCACCAACCGTAGCAGCGCCGTCGGCTACAATTTGTCCCTTTTAATGTATTTACCCCGTTGAACCCGCGGTTTTGGTGCATACAAGTATTTTTGTTCGAACGTTGATACAGAACTAATGGAATGCTTAGAGTATCTCCATTAACTGATAAAAGGATCTTGTCGGTATCAGTATACACGACCTTTCCCTCGCGTTCGGCTATCGAAAGAGCCCCTGAATCTAGAGCTGCTTGTCGTTCCAACCCAGTTCCAACAATGCATTTCTCGGACCGGGAAAGAGGAACTGCTTGACGTTGCATATTCGAACTCATTAAAGCTCGATTCGCATCATTATGTTCGATAAAAGGAATGAGGAAGCTCCAATAGAAAAATATTGGAAGGAAAAATACTTCGAAGATGAATCTGTTCCCATGCAATAGTCAGAAATTCTTGCCGGTATCGAGCCGGAACACGCTGTTCTTCCTGAATATCCTGATTCAAGGCCAAAGAATTTCCCGCCGCTACCCTATAGTATTCATCTCTACCTGGTGATAAATAAAGCATCTGTACTCCTGTGGATCTCTCAGAAATTTCATAAAATGGACTTTCTAGAGACCCCCAATGACCAATCCTCGCATGAATTGACAAGGATCCAATAAGTCCAACATTGATGCCTTCAGATGTGTCGATTGGACAAATACGCCCATAATGACTAGGATGGATATCTCGTATCCGAAAACTAGCAGTTCGCCCTGTTACTCCCCAGGGCCCAAATAACTCAATTTTCTCCCATGAACTATTTGTGTCAACGGATTAGTTCGATCCAAAACTTGAGATAATGGGTGTAAACCGAAAAAGATTCATAAGTAGTTGTTAATGGAGTTGAAGTTACCAAATTTTGCGGGGTCGGTATCAATTTATGCCGAATTGCTCCACCTATAGTTCCTCGAACCACATTTTCTAAACGAACCAGAGCCAATCCAAATTGATCTTGCAAAAGATCCGCTACAGAACGAATACGTTTATTTTTCAAATGATTCATATCGTCAAGTGTACCCATTCCAAATTTCAGCCCAATCAAAAGATCGGCGGCTGCCAATATATCTCGTGGTAACAAAAATGTATTGTTCTGGGGTATATCAAGGTTCAGTCTTCGGTTCATATTTCGTCGACCAATCCTTCCTAATTCACATCTTTGTTGAAAGAATTTCTTTTGTAATTCCTTACATAAGGATTCAGAAAATACCGGATCTCCACCTACACAAGCAAATTGTTGATAAAACTCCAAAATGGCATTTTCTTTTGACCCAATTTTTTCTCTCCTTATCATTCAGAAAAGACAAAAAATTTCAGGATAGCAAACATTCTCTAGAATTTCTCTTAGATTCGAACCCATAGCTGATGATAGAACTAGAATAGATATTTTTGTTTCCTACTCACACGAGCCCATATCCTTGCTTTTCTATCAATCTCTAATTCTGATCTTCCTCCCCAATCTGATATTATGGTGCCGGTATAGACCGAAATTCCGCTATGGTCCAACTCTGACCGGTAATAAATACCGGGACTTTGCAATATTTGATTGATCACAATTCTATATAGTCCATTTACTATAAAAGTTCCTAGGGAATTCATTAGAGGAATGTTTCCAATAAAAACCGTTTGTTCTTGCATGTCCCTACAGGTTTTCCAAATTAATCCCGCGGATACATATAATTCAGACGAATATGTGAGTAATTCATACACAGCATCTCTTTCCTTTATCGAGGGTTCTACTAATTGATATGTTTCTACAAATAATTGAAATTCGATTTCTTGATCTGTATCTTCGATTTTGGAAACTTATAAAGTTCTTCCGCCAAACCCTGATCAATGAACCTACAAAATCCTTCAAATTGGATCTGATTAAATCCAGGTATTGTAGACATTCCCTCATTTCCATCCTCGAGCATTTTGAATTTCCCATTTATCAAAAAATCCCACTATTGGTTCATTCTTTACCGAATTTTATAGATGATCTAGCAACGATGGGATTTAGAGTCTGTTTACTGAATCACATGAAATTTTACCCCACTCCATATCCATATATGGAGTGGAATGTATGAAACACATATAAACGGATAAATAACGAGGGTTTTCCACTTCAATTTCCATTTTTGAAATTGAAATTTACAAATGGAAAGGAATTGATAAAACATTTTTATAAAAAGAATTCTGCCACTTAGACTTATTAAGTTAAGTTATAGGATTTTGTAGAGAATATAAAAACAAAAAAATGATTCTATTTCTACTATTATAATGGTATTACATATTCCAATCCGCTTGAATACCCGAAAAATTTGGCGTTTGATCTTTTCGATGAGAGAAAGACATAAAAATAAGAAACAACAATCGATTTTTAGCACTTAACACACCCCTTTATGTTATGGATTTCATTATCCAAAAGGATTCATTCGCAGAGAAGAGAGAAATCTTTACTGATTTTTGAGTAGAATATGATTGTGAGGTATATAATAGAAATACACGAAGGGTTGTATTTAGTAAACATGTATGCAGGTATTAATATCCGTCTTTTCTTTTTCTTTTATTGTGTTGTTTATTCTATTGCCGTTCTATTTGTGGCAACACGGGTTGCGCTCTATTATCTATTAAAAGAGAATTTCGATTTTCTATTCAATTCAAAATGGAAATATGAAAATTTTCTGAGAATGACTTATAGGCAACATATATAATAAAATAGTGTGACAATTTCTGCTCTGGGGTTTACATAGACTCATAATTGTTGTTATAATGGAAATTGAGAAGGATACTGATGTATCAATTTGTATTTTCTCATGTCATTAGGAAAAGAAAATCCAATTTGGAGATTCAAATCGTTCATGAATTCGCAGTCAGCAGTCAATAGTTAATGGTTCAAATTTGTCATAAATTTTGACTTTTGCGAATGAAAATCCACATTTGATTTTTCAATAGAAAGTTGGCTATTTTGAGATGGGTGGATCCAATCCAATCAAAAGTGGGAAGGATTTGTTTTAGGATTAGGAAAGAAAAGGATTAGAAGTTAAGAAAACCGGAACTCAAGGTGCAAATCCAAAGTTCCGTACTGTAGGAAAAATTTCATAGATTTTCTATCATTTTGGCGGCATGGCCGAGTGGTAAGGCAGGGGACTGCAAATCCTTTTCCCCAGTTCAAATCCGGGTGTCGCCTGATCAACAAAACAAAAGACTCGAAATATCTTCGTCTGTTCTACTGATATAACTCGCCGAAGTATTTCCCAGCAGAAGCGGACCGTTGCCATTTGTTTGATTCGAAGCATCCGGCTGGGGCAGGGGTTTTCTGTAAATCCTCGTATCTAGGATTTAAGGAAATATTCTATATTCTAATGGTAGAGGGGTTATGAAGACTTCGAGATTCCTTCTACTCCATAAGCTACCAATCCAGCATTATATAGAAAGGAAAGAGTGTATAGTCAATAGAGTCAAACCCGGTATTCTACGATCGGGAATTATTAAGAGTATGAAAGGGGCGAAGCGGCTTCAGATAAGGAATAGAAAGAAATGCAGAATATAGGTTGCTTAGGGCCCGGCCCGAGAGGCCGCTCAAGAGCTAACTCGGATAAGCTACCTTAATTAAGAAACTGTCTGTCAGTGATCATCGACTATGGGAAGGAGAACCATTCCTATTTAAGAGATTTTGCGGTGAGGCCAACCATCTTTTCATGATTCGGCGCATTTAGCATCGTAGAATAGAGAATTTGTAATAGTTACCGCCCCTTGGGTCCACTACTAAGAATGGATCCTTCTGAAGCCATACCAAAAGGTCATACTATCTAGCATAAAAGGGAAGAAATGAAAGGATTCGTAGGAATTGAATCAATAGTAAAGATATCCCACGAGGAGGATTCAATTCATCTTTCAACAGCATTTCCGACATTCCACTTTCGGAATAAACGCAGGACAGACAGATATTGAATTAACGGAAAGGAAATGAATTAACTTCAAAAATGAAGGAGCAGGAAATTCAAGTTCAATTTCTGCCAGTGTAGGCTTGCTTCGCTCTATCTCCCGTAAGCTCGTGACGCTTAACGCCCAGTCATTACGATGGGACTCGTTGCGTTGGTGACTGCTCTCATTTATCAGTGACTAAACAACTGAAACCTACTAACCTCTAGTGACTAACCGCAACCATTTACGAGCCTCCTATCCGCCGGAAAAAGTTTTTATCAAACCAAGCTGGTGATTCAAGCGCAGCGCATTGCATGCCTCCACTGAACCTCTCCCTCTATCGCTGCCTATCTAAGCTAAAAAAGGCTTTCAAAGGGCCTCCTCGTTCACAGGAAAAGCGAATTCAGTCACCCCAGGGTCTCAATCCTAAGATAGGGAGAAAGGCAAGTATGCCATTACTCTCAGCTGCTCACTCTTGAGAAAATCTTTTCCGGGGTGAAGGAATCACCAGTCTTTTCTGAATAAGCGGTCTTGCCGAATACAGTTACCTCAAAGGTAGTGATCAGTGGAGGAAGAATCAGCTCTTGGTAGTTTGGGCATGGAAAAGGCCTAACTTTGATTTTCTTGTTCACAATCCCTTTCAAGTTTGAGGGAAGACGGTGCTGGTTTGAATGCAAGGCCGTTAAGGGCTAGGAGCATACAGGGGAAAGGTTGGGCAACTCACTTGCCTCCGCCTGGTGGAATTCCGCACCACGTAAAAGCCTTCGTCTAGTGACGGCTTAGCTACTTGTATTAATTGACAAAGTTCTTTCTTAAATAGAAAATTTCGTACATAAGACAAGCAAATGAAAGAAGAATCTCAAGCCTAATAACCCCTTAAGCTGCAAGACAAGAACGTACCGTACCCTCTTAGCTTTTCTAGAGGAAGGGGCTTTGATAGTAGTTCGAATATCAAGCTGGGAAGTCTTTTAATAGGAATGAAAGAAGGTTCATCTTAATAATTCTTGACTTCATTTGGATCCCATCTATCTTCTTCAATGAAAATGAAAGTAAGAGTCGCTAAAGCTGTAACCAGCAAGCAAAAAGTTGTGGTGTGTATTGGGATTAATAGTTCTTACGAGAAGATAATCCAGCCCTAGATTCTGCTTAATACTTTGTTGGATTTGAAACTACAGCCTCAGCCCTTGCTCCATTAGCTGGTTCCTCTGATCACCATCTGTTTCGACTCGTTCGGACCTAGTGATGAGCTTGACTGGTGCTCTAAGGCGAACGCGGTCTGATCTGGCTCAACGTCTAGCTCCAGTTTGAGTTTGCCCTCTCTTAAGCAAAAAGCCTTGTTTCCGGGTCGTCACCTTCTTTCAAAGCTTGCTAAGCGTTGGAGTTCTACCTGAATGACTTTCTTTCCAAAGCAAGAAAATCACTTCCAACTAGCCGCATTCTCCTCTATCTGTAGCGAAGGAGGCTTCTTATTGCCAAGATTTCCTTCTTTAACTGAATGTGTGTGTTAACTTTCAATAGAAAGGGTACTTTCCAGCTGATCTGTAACGGATCAAAGCGACCGTTAGTCGGGCTGATCTGTGATTGTTGGTCAAGGCGACCGAGGACACATACCTCCTCCATATTCCTTAAATGGGCAAGACTCGGGTACTGCATGCGAGAAGTCAAAAGTTTACTGAACAACTACAACTACTTAGATCTTGATTGATTTGGATGCAATGGAATTTTAGCATTACTCTAAAAAAGCCATCCCATGAGTATACATGACCCCACAACCTGTATAAAGCGTACATCTCTGTTCCGAGGTAAAGCTTTTATAAGCAAAGTCAAAAGCTGGAATCCGAGAAGACAAGACCCCTAAGAGCGTCTTGTAAACCAATTATTCGATCTGGAGATCTCAGCAAGGCTGCCGGAGACATCACTAAACCCAGGGATGTCTCTTGCAAAAATTCGTAGAGATTTTCCCCAGTCCAGCCTACCCGACTGATATAATCTAGAATCCACTCACCGCCCTTGGGGAAGGGATTTCGCCTCCAAATAGGTCTTGGCCTTGTCTTGTGTAGCACGAGATATTGAGAAGTGGAGTCCAATCATCACCTCAGTGGGGCTCGCTATGCCACTTTCATTTTCTTTGCTAAAATGTCTTCCTAGCTCCAGTAATGGAGAGTACTGCCCCAAAACGCTCGTATCAAACGCGCAGGGGCCTACTAGTAAAAAAAGAAAGCATGACATCGACGTATCGGCAACCGATAAATATAACAACAGGGGTCTTCCCGGCGTTGGTGGGACCAGGTTCATCAGGTA

>Decalepis_hamiltonii_chloroplast_contig4

TATCATAAGGATTTGCCTTTTCTATTGATTCCTACGAATTGGATCAAAACAATTCTTGAATGAGGTATTCAACTCCAGGGATGAATCGAAAAGAAATCTTTATTGGTTCTACCTCCTATTTTTATGAAGAGAATGAATCTTTTCTCGAAGGATCAGAAAAACGGGTCCGGATCTCCTGCGGAATGATTTGGAAGATCCACAACCAAAAGAGTGGTATTTGCTAGCAACAACATAATGGAGGCAGTCAATCAATACAGATTGATCCGAAATCTGATTCAAATCCCATATAGTACCTATGGGTACATAAGAAATGTATTGAATCGATTCTTTTAATGAATAGATCCGATCGCAACTTCGAATATGGAATTCAAAGGGATCAAATAGGAAAGGATACTCTGAATCATAGAACTATAATGAAATATAGGATCAACCAACATTTATCGAATTTGAAAAGAGTCATAAGAAATGGTTCGATCCTTATTTTGATTTCTCGAACCGAGATCCATGAATCGGAATCCTGATGCATATAGATACAAATGGTCCAATGGGAGCAAGAATTTCCAGGAACATTTGGAACATTTCGTTTCTGAGCAGAAGAGCCGCTTTCAAGTAATGTTTGATCGATTACGTATTAATCAATATTCGATTGATTGGTCTGAGGTTATCGACAAAAGATTTGTCTAAGCCACTTCGTTTCTTTTGTCCAAGTCACTTCTTTTTTGTCCAAGTTGCTTTTCTTTTGTCTAACTCACTTCCTTTTTCTGTGTGAGTTTCGGGAATATCCCCATTCATAGGTCCGAGATGATCTACATCTATGAATTGAAAGGTCCGAATGATCAACTCTGCAATCAGTTGTTAGAATCAATAGGTCTTCAAATTGTTCATTTGAAAAACGGAAACCCTTCTTATTGGATGATCATGATACTTCCCAAAATCGAAATTCTTGATCAATGGAGGAACACTATCACCATTTTTGTTCAATAAGATACCAAAGTGGATGATTGACTCATTCCATACTAGAAAGAATCGCAGGAAATCCTTTGATAACACGGATTCCTATTTATCAATGATATTCCACGATCAAGACAATTGGCTGAATCCCGTGAAACCATTTCATAGAAGTTCATTGATATCTTCTTTTATAAAGCAAATCGACTTCGATTCTTGAATAATCCACATCACTTCTGCTTCTATTGGAACACAAGATTCTTTTCTGTGGAAAAGGCCCGTATCAATAATTATGATTTTACGTATGGACAATTCCTCAATATCTTGTTCATTCGCAACAAAATATTTTCTTTGTGCGTCGGTAAAAAAACATGCTTTTGGGGAGAGATACTATTTCACCAATCGAGTCACAGGTATCTAACATATTCATACCTAACGATTTTCCACAAAGTGGTGACGAAAGGTATAACTTGTACAAATCTTTCCATTTTCCAAGTCGATCCGATCCATTCGTTCGTTACTCGATCGCAGACATTTCTGGAACACCTCTAACAGAGGGACAAATAGTCAATTCTGAAAGAACTTATTGTCAACCTCTTTCAGATATGAATCTATCTGATTCAGAAGGGAAGAACTTGCATCAGTATCTCAATTTCAATTCAAACATGGGTTTGATTCACACTCCATGTTCTGAGAAATATTTACCATCTGAAAAGAGGAAAAACGGAGTCTTTGTCTAAAGAAATGCGTCGAGAAAGGGCAAATGTATAGAACCTTTCAACGAGATAGTGCTTTTTCAACTCTCTCAAAATGGAATCTATTCCAAACATATATGCCATGGTTCCTTACTTCGACAGGGTACAAATATCTAAATTTGATATTTTTAGATACTTTTTCAGACCTATTGCCGATACTAAGTAGCAGTCAAAAATTTGTATCCATTTTTCACGATATTATGCATGGATCGGGCATATCATGGCGAATTCTTCAGAAAAATTGGTGTCTTCCACAATGGAATCTGATAAGTGAGATTTCGAGTAAGTGTTTACATAATCTTCTTCTGTCCGAAGAAATGATTCATCGAAATAATGAGTCACCATTGATATCGGCACATCTGAGATCGCCAAATGTTCGGGAGTTCCTCTATTCAATCCTTTTCCTTCTTCTTGTTGCTGGATATCTCGTTCGTACACATCTTCTCTTTGTTTCCCGGGCCTCTAGTGAGTTACAGACAGAGTTCGAAAAGTCAAATCTTTGATGATTCCATCATCTATGATTGAGTTGAGAAAACTTCTGGATAGGTATCCTACATCTGAACCGAATTCTTTCTGGTTAAAGAATCTCTTTCTAGTTGCTCTGGAACAATTAGGAGATTCTCTAGAAGAAATACGGGGTTCTGCTTCTGGCGGCAACATGCTTGGTCCCGCTTATGGGGTCAAATCCATACGCTCTAAGAAGAAATATTTGAATATCAATCTCATCGATATTGTCGATCTCATACCAAATCCCATCAATCGAATCACTTTTTCGAGAAATACGAGACATCTAAGTCATACAAGTAAAGAGATTTATTCATTGATAAGAAAAAGAAAAACGTGAACGGGGGTTGGATTGATGATAAAATAGAATCCTGGGTCGCGAACAGTGATTCGATTGGTGATGAAGAAAGAGAATTCTTGGTTCAGTTCTCAGCCTTAACGACAGAAAAGGGATTGATCAAATTCTATGGAGTCTGACTCATAGTGATCATTTATCAAAGAATGACTCTGGTTATCAAATGATTGAACAACCGGGAGCAATTTACTTACGATACTTAGTTGACATTCATAAAAAGTATCTAATGAATTATGAGTTCAATACATCCTGTTTAGCAGAAAGACGGATATTCCTTGCTCATTATCAGACAATCACTTACTCACAAACTTCGTGTGGGACTAATAGTTTTCATTTCCCATCTCATGGAAAACCCTTTTCGCTCCGCTTAGCCCTATCCCCTCTAGGGGTATTTTAGTGATAGGTTCTATAGGAAGTGGACGATCCTATTTGGTCAAATACCTAGCGACAAACTCCTATGTTCCTTTCATTACGGTATTTCTGAACAAGTTCGATGATATTATTGATGCTAGTGACGATATTGATCGTGACCTTGATACGGAGCTGGAGCTGCTAACTATGATGAATGCGCTAACTATGGATATGATGCCGGAAATAGACCAAATTTATATCACCCTTCAATTCGAATTAGCAAAAGCAATGTCTCCTTGCATAATATGGATTCCAAACATTCATGATCTGGATGTGAATGAGTCGAATTACTTATCCTCGGTCTATTAGCGAACCATCTCTCCAGGGATTGTGAAAGTAGAAATATTCTTGTTATTGCTTCGACTCATATTCCCCAAAAGTGGATCCTGCTCTAATAGCTCCGAATAAATTCAATACGTGCATTAAGATACGAAGGCTTCTTATTCCACAACAACGAAAGCACTTTTTCACTCTTTCATATACTAGGGATTTCACTTGAAAACAAAATGTTCCATACTAATGGATTCGGGTCCATAACCATGGGTTCCAATGCACGAGATCTTGTAGCACTTACCAATGAGGCCCTATCGAGTAGTATTACACAGAAGAAATCAATTCTAGACACTAATACAATTAGATCCGCCCTTCATAGACAAACTTGGGATTTGCGATCCCAGGTAAGATCGGTTCAGGATCATGGGATCCTTTTCTATCAGATAGGAAGGACTTTAGCACAAAATGTACTTCTAAGTAATTGCCCCATAGATCCTATATCTATCTATATGAAGAAGAAATCGTGTAACGAAGGGGATTCTTATTTGTACAAATGGTACTTGGAACTTGGAACGAGCATGAAGAAATTAACGATACTTCTTTATCTTTTGCGTTGTTCTGCCGGATCGGTCGCTCAAGACCTTTGGTCTCTACCCGGACCCGATGAAAAAATGGGATCGCTTCTTATGGACTCGTTGAGAATGATTCGGATCTAGTTCATGGCCTATTAGAAGTAGAGGGCGCTCTGGTGGGATCTTCGCGGACAGAAAAAGATTGCAGTCAGTTTGATAATGATCGAGTGACATTGCTTCTTCGGCCCGAACCGAGGAATCCCTTAGATATGATGCAAAGCGGATCTTGTTCTATCCTTGATCAGAGATTTCTATGAAAATACGAATCGGAGTTTGAAGGGGGAGGGAGAAGGAGCCCTTGACCCGCAACAGATAGAGGAGGATTTATTCAATCACATAGTTTGGGCTCCTAGAATATGGTGCCCTTGGGCCTTTCTATTTGATTGTATCGAAAGGCCCAATGAATTGGGATTTCCTATTGGGCCGGGTCATTTCGGGGCAAGCGGATCATTTATGATGAAGAGGATGAGCTTCAAGAGAATGATTCGGAGTTCTTGCAGAATGGAAGGGTGCAGTACCAGACACGAGATAGATCTTCCAAAGAACAAGGCTTTTTCCGAATAAGCCAATTCATTTGGGACCCTGCAGATCCACTCTTTTCCTATTCAAAGATCAGCCCCTGGCTCTGTGTTTTCACATCGAGAATTATTTGCAGATGAAGAGATGTCAAAGGGCTTCTTACTTTCCAAACAGATCCTCCTACATCTATATATAAACGCTGGTTTATCAAGAATACGCAAGAAAAGCACTTCGAATTGTTGATTAATCGTCAGAGATGGCTTAGAACCAATAGTTCATTATCTAATGGATCTTTCCGTTCGAATACTCTATCCGAGAGTTATCAGTATTTATCAAATCTGTTCCTATCTAACGGAACGCTATTGGATCAAATGACAAAGACATTGTTGAGAAAAAGATGGCTTTTCCCGGATGAAATGAAAATTGGATTCATGTAACGGGAGAAAGATTTCCATTCCTTAGCCGGAAAGATATGTGGCCATGAAAGAGGGATTAAGTGGATGGGTGCTAGAGTCGTGGAAACGCTTGTTTCTTCCATATTTTGGACCTTAGCTCCATGGAACAATATGTTACTGCTGAAACACGGAAGAATTGAAATCGTAGATCAAAACACTATGTATGGATGGTATGAACTGCCTAAACAAGAATTCTTGAACAGCGAACAACCAGTTCAGATATTCACGACCAAGAAGTACTGGATTCTCTTCTCTTCGGATAGGCCCTGAAAAGAGAAGGAAGGCTGGAATGCCAACAGGCGTCTATTATTGAATTCACCCGCCCCGAAAGAGCAACATACTTGTAGCGAGAGAAGGGCTACGGAATCGAATCGAAAATTGGATACAGATTTGGAGAATTCGCGCGTCTTCAATAAGACTGACAGATTGATCTGAGGGTGGTGATGCAGCTACGCTCACTTTTAGAATATTCTATAAGTGATAATCGCCTCGCACTCCTTTATCAGGCTTGCCATCTATATGGAATCCTCAGCTACAGAGTCTTATTACATTCCCTATATCTAACTCTAAAGGTCTGTCAAAAGGTAAAGGTCTCGATATCGGCCTTCTTTTGATATTTGAATTAGGTAAAGCAAGGGATTAGTCCTTACAGGTATCTAACTTCTCTAGCTCCTTGGGGTGACCAAGAAAGGGATGAAAGAAACTAAATAAGAAACTAGGCCCTAGCGATTACTTGTAGCGAGAAAAGGGCGAAACAAAGGCTACTTCGGGAATGGGAAATGTCTGTTCTTCGGTCTTCCTTCTCTTCCCCTTTCGAAGACGGCTATAGTGCAATCCCCGGAGGGAGGAACTAAAGGCTCTTACTAAACCGGGGATCGCTGAGGGGTGCCATGGGTCGCCCTACTCTTAGCCCTTTATATTCACTATAACGAGGATAAGAGGGTCTCCTTATCATGGATCCTACGGCGCAGGACATAAGGAGTATCGGCATGCCAGGCGAAGAAGGTGGCTCCATACCATACAAATCCCTTCTTTATGACTTTTTAGAGCTTGGAATTAAGAACAAGAAAAGCAAGAACGATTTCGCGGTCGTAAGGTCGATTAGCCCGCTCGGAAGCCATTAAAATGGCTTCCTCTCTGCAACCGATATAGAAAGCTTTTTGGTTGTAGAGTCGAGAGCTACAGGCGATTAGCTGCAAGCGAGAAAGAGATTGAGTTAGTCGCTTTCCCGTGGTTCGTGTTCGTGGAATTGAAGCGTAGCTTCCGAGAGCTGCAAACGATATAGTTGTTGCTTTCGGGCCGATAGCTGCAGACGTATGGCTTTTGGGCGAGGGCGAATCACGTTCCTTTTAGGCGAGAGAGAAAGAGAAGTCCAAAACAAATCGGAATAGGGATAGAGGGAAATGAGGCGACGAAGAAAGCCAATTGGCTTTGTCCAACTGCCTCCTGTTCAATCGAACCGGACAGATGTTTTGATCAGACCGAGGAATGAAGGCAATATCTTATCGAGGCAATCGTCTTCTTTTCTTGCATACGAATTGATGAGAATCATCTTCATGTCGTTATTTGGGATAGAGAGTGATTGTTCGACTATAGTTTGAAATGAATTTTTGTTGTTCCCAGTGAAGGCTCGCTTCTAGCGGGGCTCTCGGCCTTTTTCGAGAAAACCTATGAGTAAGCTTCCAGAAAGAAGTGATTGCCCGGGGTGGCGATCTTAATAAGTGTGTTTTCACTTCTCTGAAAAGACTGGCACAAGGATCGAGCGAATTCCTTTATGGTTATGGGTATTCTAATATATAGAATAGAATCTAAAAGAAAGAAAGGAAGGCAGCAAGAACTCTTCTAGAACCAACGAGTGGAATGAAATGAAGCGAGCATAAAGGAAGTGAAATAAATCTCGTCTAGAAATCTCGTTGGGGCTTCAATTTCTTCTTATTAGTATTCGACAACTTTCAGCTCTACAGATGATTGACCGGGCCGAACTCGATTTCAGCCTTCTTCCCTTGAAGACGATTGGCTGGCTTTCAACGGCCTACAATTCAATGGGGACTCTTTGACGCATCTGATCCCGCTTCAACGTTAAGGATAAGGCGATCTGCCAACGTGGATGGCAAAATAGAAGAAGAAGAGGGACTTTGCCAGGAAAAAATCTATTAACTTCCCTTGACTTGCCGCCCTCGGCTCTAATAAGTGGAGGGAACCCCATAGTGGGGAAGGTGGATCAGTAGACTGACTCTTCTCCTTATTATTCTTTATTCTAGGAAAGGTTGTTAGAAAACTGGGCCTTACTCTTCTTCTTCTACTAGAGGCTTACGGATTCCTATCCTATCCCTATGGCATCCCTTTGATAGGCCAACATGTTGCTTCTAGCCGAGCTTAGATACGCGGGTCAAGCCTTCCAACAAAGGAACTCAAAGCCAGTGCCAACTGGGCTGACTTCAAAGACTAGTGTTTTCCAGCTCCCTACTCTCCCGGATTTTGGATTCCCTAGACGGATGCGTTTTCGTTTGAAGGCAATTCTGGAACGGGCAGAGTCCCAACGTCAAAGTCAAGACCTATGCTATACGCGCTTCCTATCTTTGCTCTTCGATTCCCTTTCGTTTCTGACTAGGTGATGAGGCCTTAACTTAAGTTAAGGGGAGCTTCTTACATACTTATTGGCTAGGCTTCCTCTTATACTGCTAAAAAAAATCATTATCATATATATAGAGAGAAACAGAAAGAAGAAAGAAAGGATACCACAATGATAAAAGATTTGAAGAAGATAGATCCGATCCAACGAGTGGAATGAAATGAAGCGAGCATAAAGAAGGAAGTAAGCTGAACTTCAAGTAAACTAAAGCAAAGGGCCCTTACCCACTCCCCTTTTCCCCATGGTTAGGGGCCTCGCCTTTTGACTTGATATGTCAAAGAAGACTACAACCGATAGTTGATCACCTTTTGCTTAACTTAAAACGCTCATATCATATTTATGATAAAGGAAGCACGCTCTTTTAGCTAAAAACCATGAAAGCCGTAGGGTGGTCACATACCAAACAAACCCAAAAGAATCAAAGAAAAGCCTATGGACATAAAGATTCAAACAAAAAGCAAAAGGAACCATATCTTACTGACTGAATGAGGAAGAGCTCTTTATGCGGTCTCACTTTACCACCATCTGAAATGCGCGAAACTACGATTGGTGGAAGCCAGTCCATGAAGATTCTCCCTTTTCTTACGTGAAATTCCCTCTTTCGGTAAGCATCCAGTATCTCAGCAAATGAACATTCCTCTAAGCTTATCCTGTAGCTTATACGTCGTTTGAAAGCTGCTTCAAGGATCCAACGAATAGCTAAGGTTTGTTGACGATCCTGGCTACAATCCCAGGGACATCATAAATAGTACCAGCTATTCCTACTTTTCGACTTCGCAGATGGGCTTTATATTCTCTAGGGCGTTAACCATAAGTTTGATTACATCGCGTTCAGTTCGAGCTAGGCGATGAAAAGTTTGATAAACAATAGCACGAATTCTTGTTTTTACCTTCTTTCATGCGAAAGTTGACCAACTTCTTGATCAATTGTTTTTGTTCACGATCCAAGCCCCCATATAGCAACAAATTTCCGAGCAATTGGATATAGCTTTCGATGACGAGGCCGAATAATTTATATTACGCAATCCTTACTTTCCATCTTTATCAGCCAGCTCCCCTGTTTCCATCTTTCCTTTGACCAACGGGTCCTCAAACCAACCTGTCCTCCCCACCGCGCTTATGGCCAATACCCAATTATGATCTGATCATCGTTCTAAAAGAGATCAACATTTTGTATTGCTCTCATAATCCGGCGATAGGGAACACTCTCGCGTGCCGCGTTCCATGATCTGAGTGACGTACGAGCTATAGGTACGTGGATTCAACAGCTCCCCTTGCGGAGAATGGAGAATGCTCCGAATATGTCTATATTTTGAAAGAATGGAATGGGAATTCAACCCATCATCTACCAATGCCGCCTCCACATATCGTTCCACTATAACTTGGGCGTCGATAATGGACACCATACGTCCTAGGTCCCCATTCCCCCGAAATGGAGGAGCAAGTACCGGTTATAAAGGAGGCTCCTTCTCGTCTCATCCGAGATAAGAAGAGGTTGGGCCAATTCGGGTATAACAACCGCTTGAGGTACCGGCGGAGCAACCGGTTCTTCCGGCAATTGTACCGGGGGCTAGGCGGGGGCGTGAAAGCATTGCCCGCATCGCAATAGGCGATGTAAGGGTTTGGTTGAAGATCGATGCAGTTGATAAGACAAAGTAATACAAACAATGAAATCATACTCTTAGTCACATCAAAACGGTGCCAATAGAAATAGAAACGGAATCTCCATAAAGAAAGACAGGAAGAAACAAGGAAGGATACAAGGAAACATTTAATATCGTGATGTAAGTCTATTATTCCTTGCATCATAGGTGTTGCTGCGTCTTGAAATCCTAATTGCCATGATTCCGCTGCATCACAAGGAAAGGGGTCTCCACCGGAGCAAGTGTCAGAAATAGCCATTCTAAAACAATCATTTGCTTTGGTTCATTTTCTTTTCTTTATCCGCTCCCCAAAACAAAAAAGAGAGACTCTTGAGAACTATAGTGAGTTGGTTGTTGACCAACGGAAAGCATGGGATACTTTCGAAATTGCAAATTATTTCTTTCGTTTGATTTGCATATGTTAAGCATAGTAGCATGGATTGGAGCTTCAACCTTACTTAGATGGAAGAGAAAGTAAGCAAGTTCCTACGATTTTGCTTTCTCTTATCTTAAGATGGATGAAAAGAGCGCTCCTAAATGAATCCTTTCTCTTCTATACGAATACCACCAGACGCCATTCTTTAGAAGGCGTCGGCGTTTCGGTTTCCCTATCTAACCTTCTCGCGGAACACTTACTAGATTCTACATCTCTTTCAAGAGACGACGAGATCTGTTGACTTCACTCCGCCTACCCGCGCCGCCAGCAGCACAAAAGAGCGGCGTACGAGCGGAACAAGACCAATATCTGAATAGATAGAGCAGACCGATAAAGGAGGAGAATTCCACAACATAGCCTTTTACCGAAGCAGGGCTTTCAAAGGAATTGTCAGTGGTAGACTGAAGACAAAAGCCTGCCTTTCGGAGACTGAAGAGCATAGATTGAGATTTAGATATGAAATTGATAGCTCAATGAAGAGATCACTCCGCCATGCAAGCCTAATCAACAAATCTCATAAGTAAAGGCCTGTTCGCATCGCAACTAATAGAAAAAAACTACTACTAGACTAGACTAGTAGTGGAGTGCTCCTTGTTGTTCGGATCTTGACCGGATCCGAGTTTCCCAAGCTCTATGCTGTTGGGGAATTCTGCAATAGTCTTACCGCCTTCTTGATTGACTATATTTGAGTCTTTGGAGTACTTTGGGATTATATTCCGCGCCGAGGATTTGTGCTTGTGGGCCGGGGTGAATATTGCAGACCAGCGGATCTGGTGGTCGACAATCGTTCGGACTTGGTAAAAGTTGTCGCGGCACCTGTAGTAGGACAGAGGACTTATCGCGATGCCCGCGGACCAATTTACGATGTCTCCGTCGCTGACGTTCGTCAAGCAGGCCACGTGGATTGGCCAGGGTCTTCTTCGGCTAATGAGACCTCGATCTCGAAGCCTTCGGACTATCTTTTGATAGGCGCCTCTATTTGTATGGGGAATTCGCTGCTGATTGATTTCGC

>Decalepis_hamiltonii_chloroplast_contig5

GCAGGAACCTTTTCGAAATATGCCTTTGCTTGACACTTAAGAGTCAGAATTGGTAGTTTGATAACTTGGCCTGGCCTGCGCTATAGGGTATGAGGTCAGTACCACACCGTGGGCTTGAAAGCCTTCCATTCATATAAAGACCTCTAGCCTTTAGTTAAAGGGGAACTGCAAGAGACCTAGACGATGAAGTTGCTTTGGGTGCTTTTCTAACGGCAATTGCCCTTTCCAACGAAATAGCCTTCTCAGGAGATAAATAAGGATCAAGATTTGAATCTTCTTACCTATGCTTCGTATGCCAATGCCAAATCACCATCGGCCCTTACTAGATGCTTCCACTGAAAGCCAGAACATTCGCATAGAGATGAGGAACAGCAAGCGGAGTGGCGAGCTTTAAAGGTTACCCTGAGTTGAGGCTCCTCCCATCAGGAGATCGAGTGGCAAAACATATATGTAAAGATGGACTTCCTATGGATGCGAGAAGATGTTAAAGCATGTGCCCCGGATGCGGATATGCCGATGGTTAGACGGCTAGCGAACATCCTTTCTAAGAAGTTGGTTTCATGAGGCTAACTCCTAGCGTCCCCGTCGCTGCGTTAGGGAACTGAAGACTCACGAGTGAGTCAGCAAGATCAAGAGTTATCAAGATGTGCTCGCCGCTTAACGCCCTTGCTTAAGGAATGAACATAGCGGATCATTCCCCAATAAGAAATACAGCATAAATCTCTTAAGGTCGGAATCTTGATGCCTGCTGCTTGACAACACTAGCCTTGCTGAGTAGTTATACACCTAGCAGCGTACAAGAGACGTCTGGTTGTCTATGAGAGAAGGGCAAAAAGAATGAAGAGCTATTCTTTTAAAAGCTAACCATGCGTTCACAGTCGACTCAACAAGACGGGTCACTCCCTACCCAAAGCCAAGCCAAATACCTATTCTCACCCTCGAGTCAGGAGTTGTTGCCCCAGAGAGCTGCCTTCGCTAGCCTAGTTAGCCTTTCTAGGGTGCAATAAAAGTACCTCCAACTTTTTGCCTTTCGGCCTGATCTTAGGCCCTGACTCACCCTCCGTGGACGAACCTTGCGGAGGAACCCTTAGGTTTTCGGGGCATTGGATTCTCACCAATGTTTGCGTTACTCAAGCCGACATTCTCGCTTCCGCTTCGTCCACCACTGCTCGCGCGGAGGCTTCTCTCTAAGGCGGAACGCTCCCCTACCGATGATGTATTTTTACATCCCACAGCTTCGGCAGATCGCTTAGCCCGTTCATCTTCGGCGCAAGAGCGCTCGATCAGTGAGCTATTACGCACTCTTTCAAGGGTGGCTGCTTCTAGGCAAACCTCCTGGCTGTCTCTGCACCCCTACCTCCTTTATCACTGAGCGGTCATTTAGGGGCCTTAGCTGGTGATCCGGGCTGTTCCCTCTCGACGATGAAGCTTATCCCCCATCGTCTCACTGGCCGACCTTGACCCCCGTTATTTTGAGGTCATATCTAGTATTCAGAGTTTGCCTCGATTTGGTACCGCTCTCGCGGCCCGCACCGAAACAGTGCTTTACCCTAGATGTCCAGTCAACTGCTGCGCCTCAGCGCATTTCGGGGAGAACCAGCTAGCTCTGGGTTCGAGTGGCATTTCACCCCTAACCACAACTCATCCGCTGATTCTTCAACATCAGTCGGTTCGGACCTCCATAGTTTCACCCAAGCTTCATCCTGGTCATGGATAGATCACCCGGGTTCGGGTCCATAAGCAGTGACAATTGCCCTATGAAGACTCGCTTTCGCTACGGCTCCGGTGGGTTCCCTTAACCAAGCCACTGCCTATGAGTCGCCGGCTCATTCTTCAACAGGCACGCGGTCAGAGGCCTAGGCTCCTCCCACTGCTTGGGAGCTTACGGTTTCATGTTCTATTTCACTCCCGATGGGAGTTCTTTTCACCCTTCCCTCACGGTACTACTTCGCTATCGGTCACCCAGGAGTATTTAGCCTTGCAAGGTGGTCCTTGCTGATTCACACAGGATTCCACGTGCCCCATGCTACTCGGGTCAGAGCATAAGCTAGTGCTTTCGGCTACTGGACTTTCGCCATCTAGGGTGCAGCATTCGGGCCGCTTCGCCTAGCAGCACGACGCTTGTGTTGCTCTCCCACAACCCGTTTTCACGGTTTAGGCTGCTCCCATTTCGCTCGCCGCTACTACGGGAATCGCTTTTGCTTTCTTTTCCTCTGGCTACTA

>Decalepis_hamiltonii_chloroplast_contig6

ACTTAGGGGCTTCGAGATCCTATCGATTCAGGTGCGCAGCTGATCGAAGCTCTTTGTATGAATGAGGATTGGAGGATCACTCTTCCCACAGATGTGAAGATGCAAAGCCTTTTATCCCCCATCAAGGAAATAGCCGATCCTTAACCCGTACCCCTTGATTCGCAGCATAGGTGGAAGGCTTGCACTTATATACAAGGACCTCATTATCTTTCCCTCCTTTTCTTGTTCGAGCTAGAACACAAACTAGAACATAAGAACTCGAATCAGGAACGAGGGCTAACTTCTTCATCTTCTTAAGACGACCTTACCTAGAGCAAGCGGTATGGCAGTACAATAGAGAGTTCGATCAGAAAAAGGAAGAAAAAAAGTGGAATGAACTTATGAACTCTCAATCTTTGTCCTTCTTGTGCCACTGAAGATCGATTAGATGGATCTAGCTTAGTTCGACTGTAAGGAGCCTCTTTCTTTACTCATGCCGATTGAATGGCTCTAGAAAAATGGCTATGGAACATGGGTAGCATAGAATGATTGCACAACTGTCATAAGGGAGTTGCTTTCTAGAAGAAAGAGCAAGAAAGAGAGTCTCCCGTAGCTCTTCTAGTTCATCTAGAGTCCCGGACAGTAAAGAAGGGAGTACCACTAGCAAGATAAAAAGAGGGTGCCAGAGAGTTGGCATCAAGGAATGGGAGAATCAATCCAATCGTTTTAAGGGCACAAAGACAAGCATGACTCACGTAGATCCAGAATGGAAGGGCTGGTCACTCTCTAATTAAGCACCTCTTGCGCCTTTAAGATTCCTCACAGAGCGATTGAAGAAGCTCGACCTAAGGGAAGGAGTGTAATAAAAGGTTTTCCTTACTTTCAGCGAGAAGGGGAGATTCGGAGAGGTTGAGAGGGCGGAGTCCCCAGGCATGGTTGTTGAGAAGAGACCTTAGTATCCTGATTTACAGAATCCACAGGAAGCTCCTTGCTCTCTCCTCAAAGGCATTCAAAATCAAAGTAGGGTCGGATCACTTTTTACACATATGGAGCCCAACCCCCTTCTCTAATGCGCATATTGAGACTGAGCTTGCTTGCATCTGATCTAAGCCTATCGCCTGTCTCCAACTGCCTTTCTATTGCGCCTGCCGTTAACTCCTGTTTACCTTCCTTTCGGAGAGTTCCTTACTCATAAGTAAGATAAGGATAGGTTGGCGAGACCTCGTAACAATTGCAATGACTAGTTGTGGGACTCTCCTCCTCTATTCTCTCTATGTCATCCTTCTGATATGAAATTTCGAGAAAGCAGGCAAGGAAGGTAAATGGCTATACTACAGAATCAGAGCAGAGGCGACCAACGTGTGAAGCCACTTGACCGATGAGGTGCAGGAGTTAGGACTCTGACTATAAAAGATACACGAGCCCATCTATTAGAAACGCTCTTTATTGAGTAGCCAGAATAGTTTGTAGCGAGAAGCGTTCCTCATAGGCCAGCCAGGAGCTACAAGCAACTAGAGCGCAGCGATAAGAGCGGGGACACTCATTAGATTAGTGCTCCCTTCGATCTATCATGGATTAGAGGGAGAAGCCACCTCTATTCCTACTAGCTTGAGCTCTAACCTTCCTTACTTCTATCTTATTGGAAGAGATTGTGACCAGCTATCTACGCTATTTGAAGATCTTTGTCAGAGACGGATTCTTGACCAAAATCAGTGACTAGTACCTTTCCGGGATGGACGGCGATAGGGATTGTTAACAGCTGTATTTATTTAATAAAACAAAAACCAACCAAACAAAGCAGTCCCTCGGTTTACAGCCAGTAGCCAGTCGGATCGTTATATACACAATTCTCGGTCCAGTCGAATGCGAGCCCAGCTAGCTAAACCAGTTCCAGAGGTCATGAGTGAATCTCGATCTATGGCCTATTTGATTGAATATGTCAACAGATTGTCACACCGTATGCCCTATTATTCATCGATGAGTCCCCAGTTGACTGTAATCGAGGAGGTTGAATGGTTTGCACAATCCACTGTCAAAGTAGTTGTTCGGCCAATAGTAGGAGTGAATGAAACTCCGGATTCGCGTCAGTCTGGATCGATCGAGCTAATTGCCCCTCACAGCCCTTTCTTGGTTTCAGGAACAAGGTCCTCCTATTCATTGAATGGGTGAAAATCCAACAATAGTAGTTCCGTCTTCCTCATTAACTATGCTTCCCAGTGAGCTAGTTTTGCTTTCGGTTAAGTGGTTAGCCAGCCAAAGGGAGTTTAGGATTGGTCGGAGGTAGATGGAAATAACTAGTAGTTACCCTAGTTGCATAGTAGTTCAGGCCGCGTCGGGTTCTCTCTCGTAGGGATAAAGAACATAGTAAAGGGGAAGGCTCGTCGTCGAAAACAAGAGCTGAGACAAGAAGTACGGATGGATGGTTCATTTCATGTCTAGTCAGGTCTCGAGAGCGAGTGGAAGCTAGATCTGGAATGTCAAGAGCCAGAGAACGACATGCAAGTAAGTAGGGCAGTCGTCGGAGCTGTAGTCATTGTCAGTAGCAGAGGCTAAAGCCGCTGGCAATGCAGTCTAAGACATTGGTGCATAACGGGAAGGCAGGTTAAGTCAAGTGTCAAGTCAGGGTTAGTGCATTAAGGTTTTTTTCTCCTGTAGCTGGTCGATCATCTTGCTTTCCCCTTTGAAGAAGCGTCGAAGACGCCCTCCCCTGAAATGCTCTAAGCTGGACGTCAAGTGATTGATGGCATTCAGCCCGACTCCATTCCCTGAGGGCGGAACTCTTCATCTCAAGTCTATGGCTATCAGTCCCTTGCTTCTACTTTAAGAGACCTGCTTGCCTAATCGCCCTGACTCCACCCCTAGAAAGGTGGGGTAGTCTTAGTTTTCACTTCCACTTCTCTTGAATTGACCTAGCCTGTTGTATTGCTAGTGGACTCTTTTTCTGAGTGATGAAAGATAGTTGAAGATTGTGTCTGTCGGTGATATGTGAGTAGAGCGGATAGCCTTGAGCAGGGCTAATAGAGTTCTAACAATCATCGATCGCGTAAGCCGAGCTGGGCCTGGAGGCTGTCAATGGCTGTTCTGTCCCTCGCATGTGGCATGTTAGTAACTTACTAGCTTTTACTTATTCATTTCTGTGTCGACTTTTATGGTGACGTAGGCAATCTTATGCTCTTCTTTCTCAAAAATACACATACAATTTTATCAATTGGAATTGTTGCCATGTTCACTTAGTTTATTATGCTATCTGAACCTATATGTACTATTGAGCTTTGCTCGTGCAGAAATGATCGATGAAAACGCTTGAGCCCAGCTCGCCTTTTTAGAGACTTTTCTCTCCCATTTCGGGCTAGACCTAATCTTCAACTTCAGGCTAAGCCTACATAACATATTCAACCACGGATTAAGTCGACATAACATCCTCACGTTCAGTCAGGTTTTTCCAATTGGGCGGGTAGAAGTAGGGCTTTATTGGCCTTCGGCTGCGGTAGTTACAGGATCATCGGATAGCTATGAAAGGCATAACGGAAGAAAGGACCGAGCTGATTCTATAGCTGCCTATTCTGTAACAGTGGATTCTAGCACAACAACTTCTTCTTTGATTCAATTGCTGCTCCTTCTATGCCATCACTCCCATTATTAGGATTCGTACGCGGATACGATCACACCAAGAAAAGACTAGGACCGCTTAGGAATGAAGTTCGCTTTCCTCGGATACGAGAGCAGGAAGAGCCGGCTTGACTTTCTCTGTGGTAGCTAGGCCTGGTAGCATGGTTAACGGTAGCATGTTTGCTAGTCTTTTCGAAAGAAGTAGCTAGACGTGCTAGCATGCTAAGTTAATGGTAGCGGAGGAAGAATACATGGTCTAAGAAGAGGCTTAATCAACAGAATAGAATCCCTGCATGGCTGTTTCCTGTGTTTGAATGCCCTGTTAGAGAGGTAGATGCTAACCTTTTCAAAGAAAGGGTCGGTCTTGACTTGATGCCGAGAAGGAGCAGCTATTCAATCAGCTTTGGCACTTGAGGAAGGGGAACAAGCTCTTCGGAACGGATACCCATCTCTTTGAATCAAACCTTTCCAGGATGCTCGCTTCCAAGCATTTAGTGCCATGTTTTGTTACCTGACTACTGTTCTCTGACCTCTAAAGGAAAAGAAGCATACTTGAAGGGCCCATAGAGAATAGCCCCTACGGACACCATCTATCAATCGTTCCATCTCCGCTTTCCCCAAAGGAAGAATCTGGCTTATCCTATTATCTTTATGCCAACTGTATTGAACTCAATCATCACATTATGGTTACACCACACCATTCGAAAGACGAACATCAAGTAAAGGACTACAACATTTTTAGATTGGCAGGATGTTGTATAAAGCATTATTGTAGAGGTTCCCCAAAAAATAGATAAGGGATCTTGCCCTATAACCTAGTGAATAGGGAAGAACAGATCCCATATGTCTAGTTAAAAGCCTTTCTTTCCTTTGCGGCCTCACAAAGCCCTTCCTGCTTTCTATACGCAATACTTCATCTAGCCCCGGTTCTGTTCCCCCTCGTAGACAAGACAATTGCTAGCCTGAAAGTTGCTTGATACTAAGTCTATCTTCATTCCGTCATCTCTCCAGAAGAGCCACCGCTCCTACGAATACAATGGGGAGTAGAGCACAGAAGCATATTATGTTGTGTTGATACTCCTGTGTAGGCTGGCTTTTGAGGTTGATGCCCATATCTCTATCGTCTTCTTGGTGTAAGAGCCTATTGCTATTGTCAGTCAATCTGTCCAAGCTTTCCAAATTGTTCTATTCCGGCTGTCTCTTTCTCCGTCTTGTATGGGGGTCTAAGGTCTTTCCTTTATTTATATACATATACGAAAATACCAAATTAAGAGATTTACACGACATAAAAATCAAAATAGATCTCTTCTCGAAAAGGTAAGCCCTCTAAGTTGGTTCATCCCCTGCTATAGGAAGATTAGCGAACAGGCGAACCACGAGCCTGCACTTGATCTAAAGCAACGATCCGAGCTATCCCAGGAGTGAAAAGAAAGAGTAGAAAACCGAAGCTATCTCCTATAACAGCGAAAGTTGGAGTAGCAGGATTCGCATATATACCAGTGTATGTGAGAGCAAGATTGGATTTTTGCTTTCTTTTGAATTATTCACACCTATAACTAGAAAAGGGCTACTGGCCAGCTATACCAGGAGTCTTGGTATTCGGATAATTGATTTGGGTGATCCACGACTGCAGACAGGATCAAGTCAGAGACCTATTCTAGGTAGTCAATGAAATGAGCTACCCATTGAAGAGCAACAACCGATGTCCCCGCCAGCCTGTAGCTAAGAGTAGCTTCTCTACCGCACCTCAAAAGCGGGTATAACCTTACTAAAAGCTGGGGCTTCAGCCGCTAGCAGCAACTTTGTCCCGAAATACAATCTCAATTCCAATCTTTCTTTCGAAGCTCACCTTCCGGACACTAGTAAGGAAAGGTCAGGTTCAGCACGTAAAGCAAACCGAACCGAAAGAAGAGACTCTTTGGAAAGTTAGTCGCCCCTATCACATCACCTCGCTTGCATTTACATCTGATGAAATAGCTCAAACTGCTGATTCTCGGGAAATGCTTCTTTTCGGTTCCCAGTTCACGACTCTAGCTGGTAGCGCTTCCCTGCCTTTGTGGGGACAGGAATGAAAGACTCAAGTAGAGGAAAGATGGAACTCAAACTCCAATATCTCTTCTAGGGAAAAACAGAAGTACATCCAATGTTCTCCGTTGGCGGCATGGCACCTTGAAATTTCGTAAAGACCTGTTCGAGAACTTTCTAACGATTGGAATCGACGTGTGGCGGGAGGAGATTAAGAAGCAAAGGCAAAACCCAATTTTGGAGTGCGATTGAACAAAGGTCGAGCTTCCGTTGGTCGCCTCTTCTTTCTCTCTCGAAAGGAAGGGCAAGAGCAAGAAAGTTAGAAAAGTCAAGGTTAGAGAGCAACCTATGCAGTGTGAGATATAGTAGAAGTTGTAGTTGAAGTGCTTCTCACCGGAAGTTATGTTACATCGCCTTCCATTCCTTCTACAGGGGACTCGTTCTTAGCTAGATCTTTAGTAAGGGGGATATTCTAATGAATAGTGCTCTATCTAGGCATGTTAGCTTAATTTTAGGTTTTAGGATAGCAGATTCGTTTTCACGCCTTTACTTTCCCCGGGTAATAAAGATAGTATCAGCACCTGTTATTGCAGTAGTCGTCCCTTCCAGCCTTATTATTTCCCCAGATCCAGTTACAATTTCAGTTTCTTTTGGAAATAGGATTCCTTCCGCATTCTCCTTCAATAGCTAAGGCCTTTTAGAAGCCTGTGCTAAGGTAAGGCTCTCCAGAAGGAGAAAGCTACGGAAAATGAAAGAGAAGGAAAAGGCTCTCGTCAAGGGCATGAGTAAAGTGCTATCCTCCGATTCGTAGTCTCATTTCAGTTTGAAAGAAGGGTTTCCTGCCTTAACTTCAAAGAAATCCCTTTGTTGTAAAGTAAGGCACCTTTCATTTTAATATAGGCTTCAGGTATCAGATGTGCGATGAAACTATTAAGCGTAGTCTCATTGGATAATGAAAAGAAGGGCTTTCAGCACTAACGAATAGGAAACACTAACAATGAGAATAGAAAGAAGGAAGTCAAAATAAGGGCTTTGTTCGAAGGTTTAGAGGCTCAAATGGGTGTTTCCACATGTTGGTGGTCAAACTCTCAAGAGATTGAGCTCTGAGACAGCTTCATCTTATAGGGTGAGAATAGTTGATAGTGATGGTTCGTTGATTGAGGTTTAGTCAGGTCAGTAGTTCCATCGACAGAGGACTGAAGTCCAGTGGTGAGAATGCGAGAAGGAGGGTGAGAAGAAAGAACTTCCGATAATGAAAGAAAGGCCTTTTAGCAGGCAACTGATCACCAAGCAGCTTAGGCTCCAGTAACACTGTAATGAAAGGTTTCTTTAAGAACCGCTCCTTGGGCAAGTTGAAAATGCCAAAAATTCCGATGGAGCAGAAGGCTTAACGCCCTGGTCGCGACTAGCTCAACTGTCGGTGGGACAGCTCCCATTTGAGCAGTGGTGTCCAGCGAAGAACCGATTCCGTCTGGAGTAGTGGGTTTAGGAGGAGAAGAAGATCTTGATTCGGCAAGTCATTTTATTGAATGTGAAAAGCTAGTATCGGATCCAGGAACAGATGGTTTTATCACCCCGGTAGTACGATGGATGTTTATTCCTTTCTCTGGGCATCATTCCTTCTGAGGAGGGATGAGCGGATGGATCAACAAAAAATTTGCAGATAGATCATAGGGGAAGGCGGGCAATCTAACCATGTCAGGCGGCGGGCGGTCAAGCCCTTCCTCATTTCTGTCTCACTCGACATCCCAAGCTTACTTTCGAGATGAGTATCAAATGTGGGCACCTCGACCGAAGTGGGTAGGGAATCACGACTGGTATCGAATCAAAAGGGATGAGGAGTTTTTGGAAGTTTGAGAGATAGTGAGGAAGAATCTAGGTTGGCTTAGGCTATCCTTCGATTGAATTCAGTGATGGATGGGAAGGAAAGAGATCGTAACACTTCATCGGTGCATTCTTATCTACCATTTGCTGACACGGCTTCTCCGGAATGAAAACTCTACTGCTAATGGATTTCGAAGAAAAGTCGATCGAATAGCTGCCCAATCAATAGTAGGCCTAACAGGAACTACTCAGAGAAATGAAACTAACTAGTCTATTGATTCACTTACAAACAGGTACTTACCTAACAGAAGAGAACAAAAACAAGTAGTCATTTTTCCTTAGTGACCGAGGAACTTCAGTCCCTATAGAGGACAGGGCAGACAGTTAGACTTTTTAGTTAGAATACAAGGCCCCTTCTGAATGATCAGGCCGAACTAGATTTTGATATTTCGTTTTTACCGGGTCCTTAAATAGATTCTTCTCCTTCGGACGAAGTCGCAGTTGAAGGTAATAAGATCTTAAACCCAGGGATTCCATTACTACTCAGCTCTGATCCATTCCTTCCAACTAAGGACAAGAAAACGCGTTAGAATAGCCTATATAGGCCAACTGAACGAAATAGATCTCTCTTACTATTCCGGAGATTAGTCATAGCATCCACTTGTGCTAACTCCTTGCCAATCTCTTCAAGCGGGAGGCAAGGTTAGTTACGTAGTTGACTTTCCCGGAGAGAAGGAAGTATTAAAGCATCAAGTCTCATCTTTCCCTGCAGAATCCGTATATCCTCTGATTTAGCTGCAAGTTTCTCTTTAGTGTGCGAAGTCCGTCCTTGGAATACGCAAGATGGGCTAGTGTATATGGAAGTATGACAGGCGGTCTATCTAGTTACGTAGGTTAGTGCGTAGTTAGCGTAGAGCCACAGAGGCTTATCAGATGTTGTAAGGGATTGCCGAAAGAAAAAGGAATGGAATCGGGGAAGACAGAGCCTAGAGATCAGTTCTCTGATCAAGGAGAAAATTCTCTTCTACTAGCTGCTAAAGGCTGCTCATTATATGTAATTTAATTCCTCACTTACAACCCGGACGCAGACAGGAACAAGGTAAAAGACATTCTATCTTAGTTACGGAAAGCCGGAGATCTTTCTCTTCAAGCTCTAACTCCTCTATCTCTATTTCCGCTAGACGATGCTTGCAGATCCGCATCCCGTAATAAAATGAAAACGAAGTCAGATCCTATTGATCTCTTACCGGCAGTGGGATCTTCTTCTATAGTCTCTTCCCGGGAGTTGGATTAGAGGCTCTTCTAAGTCTGAATCGGAAACTGCTTTGACTCCCTCCGAGAAGAGAAGATAATACCTGGGTATCCTCTAACTCTTGCTTTGGAGTTCAGATTTGATCTGTCCTATCGAGGGAAGTTCCTCTCTGGCCTACCTTATCTCTTTCAGCAGGCGGGGAATAGAGGAACGCACTAAGAGGACACAGATTTCCCAGCTTCGGAGCATTCAAAGACAGAGCATTTGATTTCCTTCTTAACTCAATCTTTATGCGCTGAGCACAACTACGCACTCTGAAATAGCTGCTAAAAGCAACCTCGCCTGCAGTCTTTACTTAGAGGGCATTACCTAAGCGTCAATCTAATACGAAAAGCAAGTTATGGCCCTTCCTCTTACTATTGATATCATTGATATCTGAGTGCAGGAGCCCATACTAGATTAGACTTCTAACTCTTGTTCAATCCCGTCCTTGAGATTCCTCTTGAATTGACTAATTCAAGCATTCTAAGCAGCAGGGGATAATCCTATTTATCTTGCTATTGTTAGAGGCCCTGCTTCGTCAAGTAAGGAAGCTCAAAGCAAGCTATTTATCAGCTACTTCGTTTGAAATGCCCATGTTTGCCCTATTCTTTCTAATGCAATACCGTCCGACTAAAAGGAAGAAGCATGCCTGGGCAGGAACTATTCCCATCTTCTCACCCACCAGAACAAGGATTGAGAGGTAGCAAATCAAAGATATGGCCTCAGCATGAGAAGCTTGAAAGTCAAGTCCGGCAGGAGCCTTACGGAGACAGGAGTTAAGTCCTCGTTCGGAGGCGGGTTAGGACATTGAAATAACATAGATAAGAGCGGGAGATCCGTATAATATCTTCTTATCCATCGCGGGAAGTGAGCCTACGGATCTCTTCTATTTTAATCAAATCCCGTGCTGCTACAGAAGAGAGTTGAAAGAAAGCATCCTCAGTCTATTGTTGTGCAAGCCAAATGAGAAGTAATCTCTTGTTGCCCGTCTAATACAAATTGATATTCCCGGGCGGAGTAAACTCTTGATATTGACTTACCGGGGATATAGTTAGATAAGCCATTAATGCATCTGTTAAGCCAAGTAAAGTCCTAATAGAAGGGCTTACAGTGTCCTACCCTAACTGCAAGAACAAAAGCAGCAAGTCTTAATGCGCCTTCGTGATAAGAAAGTTCAGACTGACTGCTTTCCAATCTTCAAAAGACAATCCCGCCCGGCAGCTCTGATCTTACAATTCCTATATAGTTGAATGCAGGTACCGGGATAAGAAGAAAACCAATATGACGATTTCTAGCTAAAGAGCTAAGCCCTCATCAGATTAACCCGTAACGAATAAGAAAGAAAAGAGTTAGAGGTTAGGAGCTCTGCATGCCCTCCGATCTTTAGGCTATGACAGCCTATTCACCCGAGTTAACTCAGTCTAAGACACGCCCGAAAGGATTACATTAGCTTCCTTTCTTTCTCCTAAAGCCATAAATTCAGTTAACTCGACCTGTGCCTTACTTAGACTTCGATCCGGCGGGGATTCCAACTATTGAGATTGCAATCCCTGCATCCGGAGAATCAAATGCCTTATCATGGCCTAACGCAGGCAATAGAAGGAAATATACCGATTTCGATCATCCCAGGGATTCCTCCATTGAATCTCTTCGCGTCTTTTCGAGGACTGATAAAGACTGACAGCTCGAGGAACAAGAAAGTGCGTAGTTATTCTTCACGTAGTTAAACGCTCTTCCTGCGGGAAGTCAGATTAATGAAACTATCTAATTTATCCTCCTACACCCTGCTTCTTCAGCGAAGTCAAATAAGGTTAGAAAGTCTAGTTGGCTTAGAAGTTCCCGCGCGGGGATTAGTAATTTCTTTAGTAGTTTCACTTCATCTTTTAGAAGCTAGGAGATCTTTAGTGTATTCTCTGCAAACCTCACCACTTCGTCAGCAGGTGATAACTGCCATCTTTTAGGTTTTTAAACCATCAGGACAGAATGCTTTTAGCGGCAAATAGAACGAAGCGGACAATATAACGCAATCTATCAATTGTTGAGCTAAACCAGCCTTTTCCCCTCAAGGGGCTTCTTTCCCAGTTTTCCAAAAGAAGATTTCCAACTAACTGGATTTAGGGATTTCTTGCTTTCCGGGGCTTTTCTGCCCTGATCTTCCTGACTGAACTCCTATAGACGAAAGAGAAGCTGTAGGCCTGACTGAGCTAATTCAGTAAGCTATTCAAGCTCCTAGACCTCCGTCTGCCTAGTCCTCAATAGATGACTTCCGCTTTAGGACTTCTCGGAGATTAATCGATCTATCTTAGTTACGACCGGAGACAGATCGGGACGAGCTGCATCCTATTAAGATTTAGATGTTTTAGTTAGGGCGGAAAGCTACCTATCTTCTTCTGTCCTTATCTTCAATGCTAAATCTGAGATCGATTCCTGAAGTTACTTTGAAACATCAACATCATCAAATACGGCTACTGAAACTGCTAAATCAACAGATTCTGCTACTGAATCAGCTACTTCCGAATCCCTGTGATGAACTGAAACTGAGTCTGATTCCACTTCTTTCCTTTCCTCCGTCTTCTTTACGGTGAGGCGCGGTTAGATCATTGCAGTGAATCGGAAGATAAACCGCTTAGAGCACTTGACCATGGTCAGGTCAGTAGGACATACCGGAATCTTTGCAGCTACAGGAATTGATTTCATTCCGACTGTTAACTCTCACGGGAGTTAGCAGCGAATAGGAATCAAACAAGAAACCAACCACCGTGTATCACGTAACTTGGAGTCTATCATCTAGAGTCCAAGTTGAGAAAGATTGAGTGACATCTGAGGAAGCTGTCTTAAGTCCAGTGACAGGAGTACTCGTGAAATGGTGGCAATCAAAAGCCGCTTTCGTTACCGTAGCTGCTGAGACCAGTGCGGGGCCCCGAAAGGTGCTCTTTGTTTGCCGTTGCTAAGCGGAAAGACCAATAGTGGCGGCCGATATTCGTGTAACCGGTGAGACTTAAGCCATTTCTTCTCACCTTTCCAAATCATTAGTTGCCCGCCTACACACGGTGAAATGGGTGGTTTGCAGTTAAGAGGTACTGCGGAGGGGCTTGAGGACTAGCTTTCTCATACCCAAAGCTAAAGGGAAAAGGATTCTATTTTCTATTGGACTGAGATCTTGTAGGGGCTGGCATGGGTCGGAGAACCACGTTTCAAGGGCCCCATACCAACTGCCTGACCTCATACAGATATAAGGCCTCTAGTTTTCCCTTTCTCGGGATGTCATTGAAGAGCGTATAAGACCCGCGGGTCAAAGGCTCTTATTGGTCATGGGAGTGAGGAGGGTATGATGGAATTAGTGTCTAAGGCTAAGGGGAAAAGCAATCACTGAGTGAGGGATTTACTTTATCTTGCTCAGGGTTAGGCATTCACTGGAAAGGGTTAGGGACTACGGGGCTAACGGCTTAATGACAGGAAACCATATTGGACTTGACTTAATCCACATGCTACCCATTTGTTTAAACCATACAAACTAACAGTGACTGTACTATATAACTACAGATTGACCTTAAACCTGATACTCTCTTGAAAGCATTGACTGAACCTATCCTCTTTTCTTCCAGAGAGGCCCCCATAGAGGAATTTGTTCACGTTCACATAAGGAAGCTTAAACAAGACGAATTCTTCAAATGCGGTCCGAAGAAGGGAAGGTTGTTAAGTCACATCCGATGGATAAGCAGTGATTCCTCTCTTAGCATCTTACCCGCCTTAAAGAATGAATAACAGGATCAAACAAAGCGTAGTGGATTTCTTCCTGCTAATGAATCAACTGGTATTGGACTGTTCTCAAAGTCAAAGGCAAGGGGTCTTCCTCTTAACTACCTAAGCCTAAAAGTAGAGAGCTTACCGCTCCTTCACTTCATGCCTTTGCTCGCGCAAGCTAGATACTAGGAAGAAGAGTTAGTTTGTAGCCATTGGGAGGGCTATTAATCACATCAATATATGGCACATCTGCTATCCGTCCTAAAAAGAGAAAGGAAAAACCTCCAGGCCTACTAACAGTAGAGGCGAACGAGTAGTAAAGGTAGGATGAGCAAGGTCGATTGATCGTATCCAATCAGGCCTCTTGTCTCCTGTCAGCTACTCGCCTTCGAAACTCTCCTTCCCGTACCATCGATGAATTGCTTTCCTTAGGTAGGGTATAGTGGCGAGCTTACTTGGCCAATATTCTCTTTCAGATCCTTCTTTCGGGCTTAGTCCGTTCATTCACCTGCTAGAACTAGTCTGACTTCCACTGCTCCTACTTGATTTGCTACTTTAGGTCGTGCTGCTACTCCCTTTATTGCTAGAGTATCTTCCTTTCTTGCTTGTCTTGATTCTCCTAGTTCGGATGGAAACCCTAAGCTTTCCACGAGACAGAAGGAATACCCTCATAGATAACTTTTGCTACGTAAACGAAGGCCCAAAGCTGGAGACTGAGTTTCAATCGCTTTCGGAGCTCTTTCTCTAGACTACCCCAAGCACCCTCAGATTGACTTGACGGTGGGAACTCTGCCCACGTCTTCTCTCATAGCTTTCGACACAGACCTTGACAAAGAAATACCAAAAGACGCACCTGAAGAGATATACTCCTCAAAGTAGGCCAGACAGCGATTTTCTTACTGAGAAAGCAAGCTTTCACGAATCATTCGAAGAAGATGCTTTTCAGGCGAAGGCAAGGGTCATTTACACAACTTCTTCAGAGCTTTTACTGAGCTTTCTTGCCTACATTCGTATAAAGACCTCACAAGGTAAAGGTAGTGTAACTCGTCTTCGTTCAGACCTATTATCTAAATAAAGGGATCAGTCCATAGCAGAAGAACCCCTTCTGCCTTTGCACTCGAGGGCGTCCTTTCCTTACTAGACGATTTGGAAGGCCCCTTTCCTATATTATCATTAGAAGGCGCACCCGAAGACAGACCGTATTCGAACGAAATACCTTCATCTATATCTTCTTTTCCCCAAGCCCCACGCGAAGACAGAGATTTCAGGAATGGGTAGAAAGACCAAGGGCTTTCATTTCCCACCGAGCCTATCCGCCTTTAATACAACTCATATGATCAATCAAGAAGGAGAGACTGAGAAATACTCTCAAGAAGGAATCGAAAGAATAAGAGTCTTAGGCTGAAGATTGAAAAGAAGCAAGCGAGAAAATGAAATGGCAAGAGCTCTTATTTACTGCGAGCACCAGATCGAAAGCAAGGATTGGCAGATGTAACAGGAAAAGAAAAGACTACCGGGGATTGATTAGTAAGCTTGCCCTGGTAGACCGTTGATTCAAGCAAGAAAAGAAAGCCCTTGCTTCTGCTTCTTAGCGCGTAGTGGGAAGAGGCTACTATTGTTGTTTTCAACAACGACTTACTTGTTGAACTTGATCTATCATAATTGTCTCAGTACTCAGTATCAGCTACAATAAAAGGATGCTTCTTTTTCTCACAGAATCTTTTCAATTTGAATTTCTTCACCGGGCTTGGACCATGTCTCCCGAACAATCTCAGTACATATGGCGCAAGACGATTCCACATATCGAGGTCGGAATGGGATCGGGTGTTTTCACGTCTCACCGTAGTGCCCGGTTTGTCTTGATTTCCGATTGATGAACAAGAAGGATTGAAGAACTTAGTGCTTGCGCTAAGGATAAGGAAAATGGAACTCTTTTCTTTTGTGACTGGCCCCCGGCTACGTGTCCTTTGGACCCTTCGCCCGCCCGCCACCAGTGGAAGCAAGCTAGCCCCCTATGTTGGTTGGGGAAAGAGGGCATTTCCATTGCGAAGGATTCAATCCAGCCACAGGTTCCCCTACGGCTACCTTGTTACGACTTCACCCCAGTCGAAGACCCCACCGTGGTATGCGCCAATAAGACCACCAAAGCCTTTGTGGCACTAGTGTGACACAGAAGTCATGGGTGATCATTGGTCCGATGCTTCGGGCGAAACCAATTCCCAGGGTGTGACGGGCGGTGTGTACAGGGCCCGGTACATATTCACCGCGGCATGCTGATCCGCGATTACTAGCGATTCCAACTTCATGTTCCTGAGTTGCAGAGAACAATCCGAACTGAGGCAATCTTTCCGGATTCGCTCCGCCTTACAGCCTTGCTTCCCATTGTAATTGCCATTGTAGCACGTGTGTGGCCCAGCCCATAAGGGCCATGCGGACTTGACGTCATCCCCACCTTCCTCCAGTATATCACTGGCAGTCCTTCGTGAGTGCGGCACGCACCTTTTGTTTGTTTCGGAGCCGTTTTGGCGGGGCGTACTAAACCCACTACGTACCACACCACCGGGCGGCTCGCCTGAATGCCGAGTCTTCTTCTTAGTCAAGCGCGCTAGCTGCAATCAAACTAAAGCGCTAACTAGAAAGTGCTTCGAAAGGCGCCGGCTACCTTCTTACTGACAGCACAGCTACGTGCTGGCACTCAATTAGTAGCGCTGGCACGTCACTCGGCTCCTCGGCTCACTTCGGTTGCCCTTAGCGGCCTTAGGCGCATGTCTCAGCAACACAAAACGAGGGTTTCGCTCGTTATAGGACTTGACCAAACATCTCACGACACGAGCTGACGACAGCCATGCAGCACCTGTATGAAAGTTAGTACCATCCCGTTAAGGACAGGTTTTGTTGTTCATATGTCAAGGGCTGGTAAGGTTTTGCGCGTTGTATCGAATTAAACCACATGCTCCACCGCTTGTGCAGGCCCCCGTCAATTCCTTTGAGTTTCGGTCTTGCGACCGTACTCCCCAGGCGGAGTGTTTCACGCGTTAGCTGGGCCCCTGATCCGCGTAGACCAAGGGCGAACACTCATCGTTTACGGCATGGACTACCAGGGTATCTAATCCTGTTCGCTCCCCATGCTTTCGCACCCCAGCGTCGGTAGGGACCCAGAGAGCTGCCTTCGCTTTTGGCGTTCCTTCGTAGATCTCCGGATTTCACCCCTACACACGAAATTCCACTCTCCTCTGTCTCACTCAAGTGAATTGGTTTCGAGAGCATTCCGCCAGTTTTTGGCGACTTTGACTTTCAACCCGATTCACCGCCTACGTGCCCTTTACGCCCAGTCATTCCGAAGAACACTTGCCCCCCGTCTTACCGCGGCTGCTGGCACGGAGTTAGCCGGGGCTTCTTCCTCGAGTCATGTCATGATCGCGCACTCGACGAAAGAGCTTTACAAGCGGCATTGCCCTTCTTCACTCACGCGATATTGCTGGATCGGGCTTTCGCCCATTGTCCAAGATTCCCCACTGCTGCCCCCGTGGGAGTCCGGGCCGTGTCTCAGTCCCAGTGTGGCTGATCATCCGAAAAGACCAGCTAAGCATCATTGGCTTGGTCAGCCTTTACCTGACCAACTACCTAATACTACGCAGGCTCATCAAACAGCGCTTTTAGCTTTCTTCAGGATTTGGCCCGAACTGTTCGGCAGATTCCCACGCGTTACGCACCCGTTCGCCACTTTGTTCTCAACTCTTCTCACCTCCTGGGCGAGACAAGCTACCTTTAGCTAGGAGCCTCTTTTCCTTCTGCCTAGCTCCCCGAAAACAACGTTCGACTTGCATGTGTTAAGCATATAGCTAGCGTTCCTTCTGAGCCAGGATCAAACTCTTATTTTGAGTATGATTGGGACCTAAAGTGGTAGAACCTCGTGAACCAGGCGTACTACTTGACTTGCTTTTCTTCTCTTACGTCAATTCAAGTTTCTATAGAATAAGCAGAGGACCTCTTCTGTCTACTGCCCCTTCTTCTCTTATGCATGCAATTTGCTATTATGTGAACCTTAGCGCAAGCACTAAGTTCTTCAATCCTTTATTACAAAACAAAACTTCACATTGGCCAATTTCACACCTTCCGAATGTGTCATTTGACCGAATGTGACAGCTAATCAAAAGACACCAATTCGCTTTCAGGAACAGGAGAAAGGATAGGGTAGGGTCTTTCAGCTAAAGATCAATCTAGAAAGCAGAGTCCAAGGTACATGTGTTAAGCATATCACAACATATGCCAATCCGTTTTCTTTCGGGAACATTAGACTAGTATGTCTAGCTAACTAAGGGGCATAACAAAGATTGAGACCTAAAGTGGTAGAACGAAGAAGGTCAACCCCGCGGCTAAGGAGGTCAGGGAAACCGCTAGAATAACCATACTAAATAAAAGTGCGGCGCTATACTGGGATTTGTAGCCTGGCAATCCCGGTACGGTATAGCTGAACTACTTTCTACAGGCTTCTCAACATATTATCTCACGCTACCCAAGCCGAATTCCTGAATTCCTGTAGCAGCGTTGATAGATGAATCCGCCTACATCGATACCGGAAGGATGCCTTATCGCATTACAGATAAGGTCTGGGATTGGCATTTGATAAAGCTCTGGGAGACAAAACCAATAAAGACAGGATCCCAATAGATAGAATGCAGATTAAAGATTTGAGAATCTCTGCCAAAGGCAAACCTAGACAAAATCAAATGGAAAAGATTTCAAACTAGATGATAGCACCAATCCACTCTCACTTTCACAGGTGTGACTTTCTAGTTTCCTGGGAAGCCCCTATACAATCAAGTACGCTTGCTTGCATGCCCTTATTAGGCCATCTTATCTTGTCCTTTCAAGTCCTAACCCTGCCTTGCTTTCTTGAAAGAGTCTTTGAAATGGAATCAAGAAGCACTACAACAATAAGCCCACATTACGACTAGAAAGCCGGCTAGATTTCTAACACTGTCTTGGTCCTCCATTAGTAGCCTACCTAGGGCAGGGACAAGGCTATAAGCGGACTGTGACCCGATGCGAGAGGCAGAAAGTAACCTCATAAAAAGGCAGGTTTTTCTTTTTCAAAAGGAATTGGAAGGAAGCACCTTCAATCACTCGCTATAAAGCAAGCTTCGGCTTCGAAGGGTGCCTGTCTCAACCCTTCTCACAAGCAAAGAAGGACAAAAGAGTCTAAAGTCAACAGCCTTAGCTATTCTTGGACTCACTTATGCCTCTTTCAGTAGAAAGTCTCCTGTTTCAGGTATGTCTATTTCTATTATATATTCCATTCCATACCTTACACTTACAGCGATGGCTGGCTTTCAGCCTTTTTCCAAACTCAACTTCATTCATAGCTTAATAGATTGAATAGAAAAAGAAAATGAGAAAAGAATGGATCTGACCCTTCTACCTACTGGGTATAGGGCACAAGTCTCAGGGAACTTGCAAGAAAGCCCGCTACTGGACCTGGGAAAGCTCTCATCACACTCTCCAGAATCAAGGTGCACTTTTGATGTTTCCTTTCTATTTAAGAGAACCCTACTTTCTTCTCTGCCCAACAAGATGGAAGTTCTTGGCAGCGAGGGACATATGTGAAGGGACTCAGGATAAATGCTTTCGAGGCAATGGGTGGACTGATACCTGTTACCAGGATATCCTTTTGGAGACTTTCACGGAACTGAGACTTGCTATCAGTCTAAGAGAGGAGTATGTGGTGAACCTACTTTCATCTTCACTCACTATCTGCAGGGACTACTACTATGAAAAGAACTTTCATACAGACAATTGAGTCCTCCCTCCAGGAGCTAGCTTTCAATCTCTAAGAGCCGATTCGATAGCATCTTCTCTTATGATCTTTCTAGTTAGCACGTAGTGGGAATAGTCCCCGGAAGAGAGCTTCAAGCAAAAGGGCTTGTGCAAGCAAGTGACACTTTACTTCAAGCGAATAGTGCTGCCTCACTTCCTCTTAGAGCGATAGGATTCCTTTTGCTTCTGGAAGACAGTCTGAATTAGCACAACTATAGGGATACCTTCACGGGCAAGGCTCCAACCTCAAAGCCTGTGCCCTACTTGCTTCCTCTTTATTGAAGGCGGAGTCGTCCCTCCACTTACTTTAGGGCTCTCCCCGCCCTGACTCTGATTCTTATCGCACGAACTCAATCTTCTCCAACTCCCCGGCATTCTTTGGAGTCGATCTGCTAACATTGATGCCAACCCACTAGAGGGCCTAGAACTTTCTCTGGATTTGCCGTCTCCGTAAGCAATGGAGGGAAGGACTTTGATTACTCCTAGAGCGGTTTCCTTTGCCGGATACTGCATTCTAGATCGAAACTTCTGCCAAAGAAATTAGTGATCACTAGCTCGAAACTCTATAGACGATTAGGAGAAAACTTCCTAGTAAGCGACAAGATCCCATTCAGTGGTAACCACGATGCCGCGGAGGAGTCTGATGTTAGTCTTGAATTGTCTTCTAAAACGGAGACCGTGCAATCATTTTCAACAAATTCTGTCCCGGAATCAGAGGTTGTAGCCCTTTTTGGTCGAGGTAGCACAGGAGAACCAAGAGAGTGCCCACCGGCAAGTAATCAAGATCTGTCTCCGGATCTTCTTCTCCAATCATCTGGTTCTGGTGGCGCAAAGTTTTCTATCGCTTCATTTTATTCTGTTAGAGTATAATGCTGCTTAACCGGATTGCTGATACCACTCCTGGCTTGGGGACTATTGAAGTAAGCTATCCCACTAATGCAATTCGATGCTTCCTGCGTCGAAGGAAAGTATGGTCAATCAGTCCTCCCTCAAAGCCCCTTCTCGAGACAGTCTTTCTTTTCTTAGTCCCTTTGCTTTCTCCTTTCGATGCCATCTCTTGCTAACTTCGCATTCTATTCTTCCTCTTTCCCCATGTCAGTTGCTTCTGTCTTCCGGATAGCAATTGCAGGGGCTAGGAAGCAAATTCGGATATTTTAACTCATCGTCTTCGGGTGAAATGGTGGGTTACAGAGTTAGAGCAGGGTCTTCCTGCCTAGCGAAGATTGATGAACTCTCTTCCTCTGGGTCGGTTTTAGAACCTTTGAACAACCTGTCCTTACAAACATCCTCGAGAATTTGACTTCCTCAGTGGGTCTTTTCTGCCTCAGGCATAAAGAAGCCTGTACCGAACTAAGCCATTCCTTTTTCCAATCGAAAGCAGACTGAGGGAAAGAGAACAGATCTCCTCTCTAAGAATAGAGAGTAAGAAAGTCTCATTACAAGTAGGGCCTTAGATCTTCCTCTCCAAAGTGAGTCCTCACTTCTTTCTATCAATATCAATACGCCTATATATAGATAGTAAAAGAGGGAATGCCGAATGCTACTAACTAAGAAGCGGGAGGGAAGCTTTCAAGGAAAAGTATGTAAATTCCTAGCATCCCACATCCTCCTAGCAACCCATTGGATCTTATTCCCCACTTAACTCATACTTCTAAGCTTGGTCTTAGATAGAAAGAATTGAACTTTGTCAACGAATGACTCTTTCTTCTCGGTATCGAAGGAGAGCCAAGCCAGAGGGTAAACAGAGTCTCGGAGGAGAAGGCTAAACCACAGTCTAAGACTAAGAGCGTGGGAATCTGACTTTGTTTGAAAGTGGAACTTGAAATCTTTCGCTTTCTTGGCGAGTGTTCGTCTATCAATGAATGGAATCGGTAAAGTGGTCTCGGCGGTTCGACTGCTTTGTTCTCCACGCCTTCGTTCATTTGCCAACTTTCTGACTGCAAAGCCAGTCCTTGCTTGCTGCCTTCTTTCTTTAGAAATATCTTTGTGCCTACTTTCTTCATAGGCGATCTAGTTGGTGCTTAGCTCTGTCTCAACAAAGGAAAAATCCTTCGTTCAGGGCAAAGTGCACTTCCTTCTTTTTCGCTAATTCGAAAGCGCGCCCTTCTTCCTTAGCGGACTCATGGGGTATTTCGTTGGAAAGCTTGCCTTCGTATAGGTATAGGGCCGGTCTCATAGAGTATTTACCCGTGGCTCAAGCAAGTTATTAGAGGGCAGCGCTTCCAAGAACTACCTAATTCTAGGCTGACCCTTTAAATCGCCCTTCTGCGTCTGAAAGAAGACTTTCAGTTTATCCTAGGGAAATGAAACCCTGTGCTTAGTCTATCTCAATGATTTGGAATCCTTTGCTCCGGGATCCAGTACGATGCTATAGGAAATGAGGACCTCTAAGGGAGTCGAAGTAAGACGAAAGAAGCAGCGTCTGGCCTTGGACTTGAACCAAGATCTCCTATTCTCTTCTGTCTATACCCTTTGGGAGAGAGGACCGTAAACTTTTCTAGGAATTCAAAAGACGACATAGGGCCTCTAGATGGAGCCAGCTTAGGTCCCGACTCCGTTGTATCTTGGTTCCGGCAGTGAGTAGGCTGGAAAGAAGCGTTTTTCAATTGGAAACCCTAGGGATTCTCTTGGCACTTTCGATTGAATCCGTTGCTTTCAAGCGGTCAGAGGGAAAGCATTAGTTGTCTTCTAATTCTTACGCCATTACTCAAATGTCTTCTATCTTTGTGATAAGATAAAGAGTAGAGTAGGTCGTCTCATTCTCATTCATTTCAAAGAATGAAAACATCCTGTTTAGGCTAGAATCCATTTTAGCCTACTAAGGAATGGAATTTATTGTTGTATGAGTTAGCCAGAACGATGAAAGGTCAGACAGTCATCTCACACGCCTAGCTAGAGCTTTCCTGGGATGAGACCATAGAGTTGAGCTAAGTTACTAGTTAGGAATGAATACCAGGCTCAAGACCAAGGCGAAAACAATCTAATCTATTCTATGTTGTTAAGCGGCTCCCGGGAAAAGGAGTAAGGAAAGATCCCATTTCGGCATGCCATCCTCGAGGATTTCGCTGTTTAAGGAAGAAGCGCAATCGCCATGAGGCAAGACCCCCGACCAGGCTCGTTAACTTTAAAGAAGAAAGATCAGGTGCAGGAAGCATCCAATTCCATTTGATGCGGTACGGTAGAATAGATCTCTTGTTAAGCTCTGAACTCTTGTCGACTACTCTGCGAATGCTTTCATTGAAAACGAATAAGAATAGCTTTTCCGAGGACCAGTAGTGAAAGAGGAAAACGACTCTGTTTAATAATATTTAATAGTATGATATTTTCATGCTGGAAGAACATATATGATATATTATCAAAGAAGGTAAATCCAGATGAACTAGATTCCGTGAACAAATCCATCTTCACTCACGGAAAGCACACTTTTCACTTCGTACCTGCCTTCGAGTTCAAAGTGGAAAGTTCGATCCACGCACTCTTTCATTAGACGAGGAAAGCGCAAAGACAGATTAGCTCAATGCTTTTACTGCTCGAATCAAGGACTTTCCCATGCACACTTACTCGGTTCTATTCTAAAGCCACAGCTGAATGCCGTACTTGCCTTTCTTTAGAAAGTCCTGAGTTGTTCGCTAGTTGAATGAATCATTAATCGCACTTGCGCGACTAACCGCTAGCTTCCTTGCTTTCATCCCTTTTCTTCTGCGTGACCCCCTTACGTGGATTAGTTCCAACATAAATCCCGGTGGAGGAGAACTCAAGTAAATGCGCTTACCAACCCAACTCGACCTTCCAATCTCTTATCTGGACAGGATACGAGCAAATGAACCCTTCTATTCCTCCTCCTGGCTTACCGTCGGTGGGCATTAACCAGACCTGTTCTCATAGCTATCTATTGGTGCTCCGCCATAGGAAGTCTCGCCAGTTTTTCTTATTTTAGTTCTCTGCAAGCCAGTTTAAGGAAAAGTCCTTCTCTCTGCCAGTGGTAAGGAAAGGACTTTCTCCTTTATTTTGTTGGTGAGTGCTAAGTGTTTTGATAGTGAGCCAGTTATACCTGTTATAGATATAGAGAATTTTGAAGTACGTTATCCTTAGCCTATGTAGGCAATTTCCTTTATTTCTTAGCGAATTCATATGGAAAGAGAGTGAGCTTCCATGCTATATTAAGATAGAGCTTTGCCAGCAAGTGGTTATACTAGAAGTAAGAGCCTTTTAAGACACCTAGTCGTAGAAGACACCCAGGATAAGCAGCTAAGAGCAGCCCCCTTACTATGTCTTTTCCTATTGATTGACTGGTAAAACTATCCGATCTCGTGAGAAAACTTGGCCACGCTAGTCGGTGAGAAGCACTTTGCCCATATAACGTAATAAAAAGATATCCCTTTTAGGAAGTTTCTTGCCATTTTTAGCCTTGGTAGCTGGCCAGGCAATGACTTAGATGGCTTTCCCAGCGGGTGTGTCCACTTTTCTTCCTATTCCAGAAAATTCAGATTTGAATTTCCACCACTCGCCCTACACTGGCTGAAAGGGAATTTGATTAATTGTATTTCACTTTACTACGTCCACTCACTCTTAGGCTTCGCTGGAGTGTCTTCCGCTACTCTCTAGGCATACCCAGGATTCATCGCAAAATACGGGGAGTTGAAGGCCACCTTCGGTCGTCTCAAGAACTCAGTAGCGAAATGGATAAGGAGAAGGTAGTCGACTGATCGGGCAAGAGTTCAGTTGTAGAAGCGAGAGTGGACTACGTGAAGCTCAACTGAAGATTGGTGGAACATGGCTAGTTCCTTGTTTGAGAGGAAGATTTGTTGTAGCTCGAGCGAAGCGAGATGTCAGGAATGGGGCGACTTTCAAGAGTTGCCTACGCTTCATATGCCAATGTCATTTAGACTGAAGAAGGCTAAGAGCGCATTCTATGCCTAGTGGTTTTGGAAATAGGCAATCAGTTAACTGTCAACCATGATCCTTTTGGCTCGCTAGGCCCCTTACTCTTAGAACGGCTTACACATGCCCTCCTACTGCTACTTTGAATCTGAAGGAGGCTGACTGCCCTGCTAACTCGTCTTTATACTATTCTATACTATTATAGTGATGTCACTTCTGGTAAGAAAGGGATAAATTCTCAAGCAGCTTGATGGACATCTTCCTCAAAAGCCCTCAAAAGAGCCTATTCCAATCCATTCCAAACCACAGCACTCTTTATTCATCTGATGAACTTGAAACAACTGCTCCTTCTTTCAAAGAGGTGCAAGGGCTAACCAGGTATGAACTCATGTTACAGAATGAAGAGGAGTCCTGCTATATCTACTGTATTTTTCCAGGACCGCTTGCTTCTGATCCCGTTTCAGAGTCTGTGTCGGTTGATGCGCCTGCTAAGAAAGCGGACTCCTATTCTGAAAAGAGAAACAACTCAGCGCCCCTTTGACCGCTTGTTGCTTTTGGACTACTTGGAACATGGGGTTGCCCTCAACCCGAATATTCCGCTTTCTCTACAGTTCGGAGAAGAGAGTAAGAATCTCATAGTTAACCAGAATTCTGTCTAACCTGCACGCTCTTTTCTTCGGCCGAACTCTTTGTTAGACCGCAGGAGTCCCCATCCCATTTGGGTTCCACCAAAGCAAGGGCTTCTATGTGAATTCCATGAGAAATTGGGTTGCTGAAAAACACTTTCTAAAGGTAACTTCTCTCTCCGGCTATTCTTTTTCGAATGTAGCAGCGAGCAAGCACTCCTCTATAAGGCCTTTCTTATGCGAATCAATACGAGCTTAGTGATTAGCGTTCTGATCATCCGGGAAGTTCCACATAAAGGAAACCTACAAGATTGTCTTGGGATTGAGTTGAGCCGAGTTATAGGGGAAAAGATCCTTTCCTTTTGATGCAATCGGACGCTGAAGGTAAGGAATTTCGCCTTTCGAGTTGTCTCTCTAGGGGACAGAAGCCTAAAGGCGACAAGAAATCTTCTTCCTCATAATTCAAAGAGAGAGCAAGCTAAACAAGCAAGCAATCCAATATCCAGGCCAGGTATGAAGTCACTGGTCTGATAGGGCAAGGCACATGGGAGTGCAATACAGGTGTATAAAGCATACATTATGGACCTTGGTCCTCTGCCCCCTTCCCCTGAATGCCCTTTACTTTTAAATAAAAAGCATGTTGCATCCTTTCCAAACCATGTTTTTGCCCCAACGGGCCTTGAAGAATAGAGAGAGATTCTGGAATATCTGTTATAAGGACACCTTCCTTTGTAAACCGAGGCCAATCCATATCGGTTAACACCTAGAGGTTCTATATACAATTCCTACTGCTTAGTCTAAGGAAGAAGACAAGGCTAACACTTGGATTCTGTTTACTTCCTTCTTTCAAAGCTGGACCAGGGAGATTGATAGAGCAAGGCCTATGGCTTGAGAGATAGGCAAACCCGCCACTGTAAACTGGTTTAGGAAGATGCCTACTTGTTCAAGTCCCTCTTCCCCTCCATAGAACGATGGGGCTATGGTGGATAGCTCATGGAGCGAGGAAGCCTGGTAAGCATAGAGGTGGTTCGATAACCCGCATACATAAGTAGAGGATACGGGGATCTAAAACACGGGTCCCGTAAGGGATAGGTCCAAACCCGAGATTTGCTAGTTGAAGCCTACATATCTCTACCTTCTTCTGAGCTAGTAGTCAATGCTCATTCGGAAGCATGAGCTTCTTCAAGTCAAGTGAAAATGCTCTTTTAACTGAACCGAAGGTAGCAATGCCTTAGTGGATCATCCAATGAACTAAGCGTGTACCGGTCCATCATCAACATATCTCGTCACCCTCGCCCTTGCCCCTTACTCGAAGTGGTGAAAAATGAGTTGTTCTGTTTTCAACCTGTTCTTGTTTGCGATTGGCTTATACATTTCCTTATGACTATCTTATTGCCTGCCGTATACAGGATTGCTGTAACTGGTGCAGGGTCGGTGCTACTACTCTTTAGAAGGGAATGAGGACGAAGCACTTCCCTCTTGAATTGCGTTTGCGGGGCTTCTTGCCTCGACATCTGTCTTGTCCGTCTGAATGATGGGACTCCCAATGGAATGGTTTGAAAACTCTGCCTAAGGTCGAGTTACTTTCTCTTGTTTTGCTTTGACTTGAACGCACGTGACTCACTCGCCCGAAAGAGTGCTTGAACATAACTAAGATTCTCGTATTCATAGGCTAACGAGGAGGTTAGCCCTTTCTATGGGGTGGGACTTCAATAGGGATTCGATTCCTCCTCTCCCGAGTGCCTGAACGCGCTATTTCACTTCTCAGGTGGGTCAAATGGCATTCCGCTACTGACCTAATCGACTGTTAAGCCTACGGACTTTTATCCATCAGACATGAATGAAAGGGGATAACACCACTCTAATGAAAAATCCTTAAGGAAAGGTTATTTAATATAATAATATAGGATAGGCAAGTCGGGTCTCTCAAGTTAATATTCGAAGTTTTCGTATATGGTATGGGAAGCTCTTAGAACTCACATTCCTATCATAGAAAAGTCGGTGTGCTTCCAACCAAATCCCTAGACGTTCACACTGACTTCATGTCAGCAATCCCCATTCGACAAAGGAAAGGCCGCCGAGGAAGGCTTCCTCTCAGGGAACGTACTATTACGATCCCAACATGGAAGCTGATTCCAACGTAGTGGAGATTGGGAAACCGACCACTAACGTGTCCTGCTTTACAGAGGAAAGTAGGACCAGGACCAGCAATACCAGAGAAATCGGTGCCCAAGAGAGGGCGATTTGACGAATCGAGAATTGGATATTACGCGATCACGGCAAAATGAATGAAAATGCCAGTAAATCTGAAACCGCTAACGCTAACAAAGGAATAAATCGGTAAGAAGGAATACTGCAAGTGGCACAATGTCACAAACCACTCCACGGTCAACTGCTTGGCGTTCCGGAACGTCATTCAGGATCACATCAACGACGGTCTCCTCTGATTCCCGGACGAGAAGAAGGCAGACATGAAGATCCACAATGACCCCTTCCCAGCCAATTTCGCCGAGCTTTCGGTACTTTGGAATGGAGAACAGTTACAGAGTTTTCAACCAACTAGAGGCATAAGGCAAGTTATTTTCTCCTTACCTATTTGTCCTTTGCATGTAGGTGCTGGGTCAACAGATTACCAAAGCCGTGGAGGATAACAATTGGAAGGCTGTCAAGGTTTCTCGTGGTGGCCCCTTGGTTTCACACCTTTTCTTTGCCGATGATTTCTTTCTTTTGGAGAGGCCTCTATCAGACAAGCCGAGGTCATATAGGATATTTTTTCTAAGTTCTGTAAACAGTCCGGGCAGAAAGTGAACATGTCTAAGTCGAAGCTGTTCGTTTCCAGGAATGCTCAGAACAAGGTTGCTAAGATTTGGGATTCCATTGACCTCGAATCTGGGGACCTATTTAGGTATGCCTTTGTTGCATGGCAGAGTGAATTCTCAGACCTATGGTAGGTAATGTTCGGCGTAAGTTGTGTGGATGGAAGAGGAGGAATGCGGCTAGGGCTATATTGATACAATCGGTTTGTTTCTTCCACCATCCCTTCTTATGCCATGCAAACAACTAAGCTTCCCTTTGGCTTAATTGATCATTTGGAAAAAGCCAATAGGAAGTTTTTCTGGAATGAGAAAGACCATGAGATGAAGTTTCATTCGATTGCTTGGGAGTCTATTTGCCGGCGTTCAGTTAAACTAGAGGACAGCCTTTACGCTAAGAATGGGGGCATTTGACCTATTGAATCCGCAGCTGTCTCAGTAGGAGTAGCACGTTTTCGATCAGGAACTTCAGATAAGCTAACAGAATCCGATCTTTAGCATCCTAGTCAGGAAATCCCTCATAATCCGATATCTCGATAATATCTATAGATGCGGAATAACAGCTGTCATAGCGCAATCAAGACATTCATGCACAACGCTGGCAAGATCTTAAGTAGGAAAGGGATCCCAATAAGGAAAGCCAGCAGGCAAGTAAGCGAGAGCAGGTAGTATGAACTAACTGGAACTAGGGCTATGAAAAGCATCGAGCAGTACAGAAAGCATCTAAACATAAAGGGGAATAAGCGCTGTGAAAGAATCCGCAGTAAGGATATCCCCGAATAGGACAAATGCTATCGAAAAGTGAGCTTTTCTTTCTTAGCCTCAAGTCAGACTCTTGGCTTGGTTTCCTCTCTAGTACATGAGTCGATACGATCTCCCTTAACGAGCCATGGAGAGACAACGTCGGCAAGACCAGAGAAGCTGCTAACACCGGTTAGGCCTTTGACTAATGCCCCAC

>Decalepis_hamiltonii_chloroplast_contig7

CCTTCCTTCGCTCCCCATTTCTTTTACTGGTTTATGGAAAAGGAAACCATAAATGCAGGTTATTATTATATATAATAATCTCTCTTAAAACGTTGACAATAATGTTCTTGATTTGGATTTGTGGTACTTGTTGGCAATTAAATGATGTTTCTTACATCTGAGGTATCCTAAATGCGAGCTAGATATGCTGACTGATTTCTGGTCTGTCTCATGATCACTATTCAGAGGAAGGAGAGATAAATGCAAATTAGTCTCATAATGTCACGAACAAGGAAAAGGTTATTGCAAAATTATAGCTCAGGAAAGAGCACGTAACTAAACAACTCTCCTTATCCAGAAAGCTACAGGGCTTTAACAACATTAAGTCCATACTAAGTGTGGAAGGTGAGTGTCGAGCTGGCCGGATCTGTAAGACGTCATCCCGGAGCGAGGAGGTTTATTTCTTTTTAGGAGGAGAGGGAAGTGAGGAGAGAGAGAGAAAAGGTTTGGAACCCTGTAGAGAGGAATAGTGTAGAGGGAGTAGTGGGTGTACTGGCTTGGGGTAGGAAAGGACTATGCTGTCAGTGACGATTGGCCGAGGGACTTCCATCATGTAGCTGGGTACAAATAAGCCAGTTAAAGACGAGTAGGCAGGGCGTTAGGCTAGTGCCAGTGGGGTACGTAAACGAGGACAGATCACATGGTTTTGTACTGGTCAGTTTTGAGCTGTCAGTGACGATTGCTCTATCTCTTAAGAAAGGGAGTACTCTCTGACGGATTCGCTCTATTATTGCTTCCTATTCTTGAAGGAGTGATCGGGATTAAGCCGCGTGGCGATACCCGCGAAAAAGAAAGTCCTATTCGCTCTTTGGGGTGGGCCTTTCGTACTCAAATCCGTACGGGGAAAGGACAATTATTCAGCAAAATCTAGTGGATGGGGTTCCATATTCATGAAAAGCCAGGTTTGAACCATGTTACGGAACCAAGACAAAATTCTTACTAATAATAAGAAAGGCCCTTAAGCATAATAAGAAAGGGTTAAGGGCCTCCAACGCCTATAAATAGAAGCTTCTTTCTCTATTTGGCAAAGCAAAGGAAGATATGGAGCCAGCTACCATTGTGAGACTTACTCAAATTGAAGGTGAAGTTAGACGTATAGAGGAGGAAAAGGGTAGACAGGAGTTTCGGCTGGGTCTGTTCTGGGAGCATCCGCCTGCTCTCGATCCGGAGATCGTAGGACAAGCCATGCAAGGATCAGGGACCGCATTCGCGGTCTGGAAGACAGGAAGAGGGCTCTGCTCCTCGAGCAGCAAGAACTAGTTCTCGCAGCAGCAGCAAATCCCCACCCAATCCACCGGGAAATGAGCATTAGCTCTTTCTACTTTCAATAATATTCAAATAAGGAGTCCGTCGACCCAAAGTAGTGTGTGTTTTAATCCAGGGCTTTCTTTCGTGGAGATCTACTCTATCCTAAGTAGATTGCTTTTTTGGGTGTGTTTGCATGTATCACTAGTAGTCTTCAATGCTTCCGTTGTTAACTTTGTAATGTTGTGTGGCTGGTCTATGTGTGTGTAAGCTTCCCATTGGATGTACTCCCATGTAACGGCTATTAATGAATAAAAAGTGCTGGTTTGTCTATGTGCAGAAAGCTTCCATGCCTCGCAATCCTATCTTTAAAACCGCCACTACGGTGTCGTTAAAGTGCAGTAACGAGAGAGTTAAGTTAGATAACAAGATACCCGATACTAACGATTGAAAGTAGCACAATTAGCCGTAGAATAACACTTTTCTCGTAGAGCACTGAAGGCTACGCTTGCTCGAACATCCAACTCAAAACCCGCCCGCTTGCTTGCGAACAACAACTACCAGTTTACATCCCGGCTAAAGCCCCTAATCTTGAATTCCAGTTCCTTACTGAACAACTACAACTACTGAAAGGAATCTACCTAGCTCAAGTTCCCATACCGTAGCTCAAGTTTACCTTAGCAGCAGCAAACCAGACTTAGCCCTTAACAGCCTTTAGCAAGCAAGTTAGCTACAGCTGAGCCTGATTTTGAGTCCTTTTCCTATCTTACTACAAGGGCAAGTGGGTTGATGCCATTTATAGGCTTGTTTTCAGGCCAAGAAGGAAGAGATCAAAATATTAGCAATAGCTGCACTGCTTACTCAAGAGAGATTCTAAAGCAGGATGAGCGGATCTACCGTGCCAGGTCCAACATTTATGAATTATTATAATACGTTAGGGTATAAAATGGAATCTGTTGGCTTGGAAAGAATCCAGGAAAGTGGCCGCATAAGCGCTTTGGTAAGTTTGTGCCCAGGTATATGTTAGGGCAGGTTGGTGTTCTCGGTCCTCCGTTCTCGTACCATGCCATTCTGGATCCTTTAGAGAGAAAGAAATAGGGGCAAAAAGAATGAAGAGATGGACACATCCCACGACCAGAGTTTATTCACCGGATAAAGGCTTCAGTCGAGCTAGCTTTTCGCTTCCTTTGATTTGAATGAATGAGGAACGAAGGCGCATGCAAAAAGCCCCTATTTGGGCATGAGCCCAAGTACATTGACCTCTCGCAGACCTGAAACTCAAGTTTTGCCAATTTCAAAATCAAATAATAGAGAGGACCAAAGCCATATAGATTCTCAATAAGGAGGAACTCGACCTCGGCTCCTTGTTAGTTAAGTCTTCGGCCTATTGATTGACACTGGAACGACTGGAATAATAGTCCCACTATGGTTGGACCCTCTAGGCTGTTTCAGTTCGACGCTTTGATCCCGGCCGTAGAAGGTCCCCAGGTATGAGGATCGGACGGGGAAGTAGGAGGGAGGTAAGTTTCTCGACCCCGCTTTCCTCTCGGTCAGGCAGTAGTACCGGTGTGGGCTTCGCCCATAAATAGAAGCATGGCACATGCAATTTCTTTCTTTGGCACTGCAGCTGCACTTTAGTTAGTCCCTCGCTCTGGGTGACCCCCTGTGGAGCTAACTTCCAAAAGAAAGAATGACTTATTTGCACTGCTAACTCTTCGTTCCGAAATGAAGAATCCTAACCCACCAGACTCGTTCGTGAACTTTATATGCCCCTTTCTTTGTATAGGTTGGGCGAATCCCCGAACTCACGTCGAATACGAAGGCCTCTCTTGACAAAAAATCCCCAAAGAAATTCTATTGGACTAGGCCGAACAAGTAGCTTGTTCCATAATACAAAAGTCCATCGTCTCCCAAAGAAGGGAGTCAGTGCGTATGAATGTGGGTATGCCCTTCGAAGCACCTAAAAGTCTTTATTCACACTCCTGGACCCTGGAACCATGACATGATTGAACTCACACAAAGCCGAAGAATCGAAGGTTGAAGGGAGAAATCGACAGAAGAAAAAAAGGTTTCATTCTTTTTCGAAGCGATGGAGTAGCTATGATGAGTCGTCGGCCTTCCTGAATTTGATCCGGTGGTGGGGTTGTCAAAGTCTCCGTTCTTTGAATGAAGTCTGGAACCCCGTTCTTTAAACGAAGTAGTAGCTTCTTATATTCGTCTCTCTTTCCACCGCCTCCGTTAGTTTATTTATGTATACTATGTCTTCGGCCTCGTAGTCAATCTACTGATTCGCCCTTACGTGTCAACTCTGTAGGGCAGGTTGTAGTCTCACTTTCTTCCTGAAGTCTTGAATTCCGTAGAGCGACTTCTCGCCTTTTCTATATAGTAAGGGAGTTCCCTTCTAGGAATTCCTCTGTGGCATCGCCTACCGCCTCTGTTCTTTTAGACTCGCGTAAGGTTGGTAACCGAAAGAACGGAATTCAATACTGAAGAAAGGGGCCTATTCAAAGAAGAGAAAGTGAGCAGAAGAGGCGCACCGAGGCTATAAGGTTAATAAGGTAAGCGATCGAGCTTTGGCGATAAGGAAGTAGTAAAGCGATACGTACAGGGATTAATACCATTGCAAAGCAAGTAGTAAGCGGTGGAAAGGCCGGTACCGATCCTGTAGCTAATCTCCCCCCTCGGTTTAGGGTACTAGGGGTGGGTTGGATAGTTTGTGTTTGCCAGCAGATTCTTGTACTTAATATCCCCAAGTATTCGAAGAAAAGCAGTCACGCCAGAAAGAAGAAAGTCAAGCAGGCGAGTGTTCACAACGTTCGAAGATGCTCTTAGATAGGAGCTTTGGTACGATACGATTTTGATAGGGATAGGGTGGCAAGAGCGGAATGAGTAGAAGACTACCCCCGATCGAGGGATGGGGTCATGAGATTCCAATCTTTGGAAAGTACTTTCTTTTCTGCACTTCCTGATAAATAAAGAAGTAGAATTGGCTGAGGGAATAGGATTGTTTAAGAGGAGATAGCGATCGAGTTCTGACTTTATGAGTGAATGAGTTGCTCTCTAGCTTCCGGAACTACTACTGTCCTATGGCATCGTAAGAATAAGACTAGTAGGGGCTTAGACAGAGCTTTTATACTAAACCGTAGTTTCGAAAGCCTTTCTATTTCTATATATATACCATTATCGAATCCATTTCTTCTGTAGAGTCGGATCCCTTAAGAGAACTGCTCTCTTGCTCTAGGAGATATTTGGAAAGTCAGACTTGTTTGAAGAGAGAAAAGACTCTTAGGAGAGACTACTCTAGTCGGTGGAAAGCTAAACCTGAAGTCGATTTCCCCGTGTCTGTTGTAAATATAGCAGTTAGCACCCCCGTCTCGATCGGTAGGCCTGCCTCAGGTTAGTCCTCTGATTCGTATATGGAATTCCTATATAGAGTGATTTTCCTACGCATCGGATGCAAAAGAACGAACCCTAAATAAATCCCCAGAGGAAGGACTATGGAATCATGCTTGTCTTTCTATTCGGTTCGCCCTTTCGACCGGACTTTGTATTCCTTATCACCCTTTCTTTGGAGTATGGAACCCTTCTTGCAATAGGCCACCGCCGAAGGGGATTCCTCAGATGATACTATTGCGGTATCGGTTGTCCCTTGAGATCGATTTCCTGATGATACCTTTCCTACTTACGAAGCGGCGGGCTTATCTTATCTCTTAAACCCCCGCCTCGCCGATTAGCTCTTACTTTCCTTTCATTCGGTTACCAACCACCCTGTAGCTAATTACGTTACAGGAAAGAAAAGGCGATACTGCTTAGCCAAAGAAGAAGTTCGGTTCCTGAAATACAATAGACTAATCTGAGGCCCTACTAAGGCGATAAAGTAGATTGACTACTGCTAGAGGGCTAAGTGAGAAAGAAAGAGGTCCCTAAGCTTTCCAGCTCACTAGTAGGTGAGAGCTTCTTTATAGAATAGGGCTTTTTCCTTCTATACGATAAGGTCTTCGAACCTTTGTTAGAGAATACGCTTCTTAGCGAAAAACTGACTAAATACAAGCTTCAAATTCGAGATTGTACTTGAACCAGAAAGATTTGGGCGTCTTCTTAGGGCAGATAAAGCTGTGATAATGGCGTGTGAGGATGACTTCGTTTCTCTTCTCGGCTGAGTGGCTGAGAGCGCAAGAGAGGAAATCAAAATGGAAATGGAATTTTGCTTTGGTTTTCTTCATTTTTTCTGAATTGGCCAATGATCTTTTAAACAGCATAACCAACCAATGATTTTTTCTTGGGGAGTAAGTCATCACAAATCACCAATTACAAGACTCAGGAGTCAAGGACCCTCTGCTCAGCTCAACTTAGTTTTTGACGTTTTCCTTTTCTTTTGGGTCCATCTGAAGGGCTTTTAAGCCAAAGTAGTTGTTGACATTAGTGTGCTGTATATTCGGAGCAATCATTGGGCCTGGCCTCATCCCATCATCAGAGACCATCCTATGTATCCATTACTGACCAAGCAACCAAACCAGCGCAATTCCATTATTTGGCCCATTTAGCTTGTACTCTGTTTTCCCCCTTCAGGCTCTGTTTGTTCTGAGTTATCACTGGGGAAAAAAAAAAGAAAAGGTATATAGCATTTTCCATTAAATAAAAACAAACAATTTGAACCTTAATTTGAGGTAATTAGTAGAAAGATTTGCCTTTTCCCCAATTCAACAGCAAAATGAAACATAGGCCAAAATGGCGGTTACACCAATTCTGCAGTTTTACATGATCCAATTACAATGTACGTTGGCACCGCACCATAAGAATCATGCACACCAGCCAGAAACTGTTACTGAGCCGCTCCCTGAAGTACAATATAGCTCACAAGTCGCGCTTCTGTAACTGAATTTCCCACCAAAAGATACCGCTCTTCTCTCTACCCTCTTCTCTTCCCTTCTCTGCAATACCCAAACCCAGAGCCGCTGCCTCCTCCCTTGCCAACCCAACTCGTTGCCGCAATTACACATTTCTCTTCAAGAACCCTCTAAAATAGAAAGAGAAATCTTTTCTCATCACCAGGCAACAATGGCAAAGCCCACTCACCTTTGCTCTCCACTTCTTGCCTTCTTCACGATAGCCACTCTGCTTTCTTTGCCCACCGTATCACATTCTCAGAAGAGTCATTTTCCTAACTTTGTCTTGTTTTCTTTTTGTACCGCTGATTGAAATTAGTTTCTTTAAACAATATAATTGTATAAAGAAATTTATTGAAGTTAGAATCTGATAATGATTACAGTATTTGCGCCTTTCAGGTCCAAGTTATCTGGATTTTGCGTTTAATACTACAGGGATGCCATCAGAAGATTACTATGATTACATCATAGTTGGAGGTGGCACTGCTGGTTATCCACTGGCAGCCACATTATCGGCAAATCATAAAGTGCTTCTGCTGGAAAGAGGCGGCGTTCCATATGGGAAGCCTAATTTGATGACACAAACAAGAAGGTTTTCTCGCTACCCTCATGGAGGTTGATACCTATGACTCCCCTGCCTTGTGCAAGAAGGTCAAACTCTGTAGATAAGCTACCCACCTAGCGTGCCTCGCACCGAGCTTATCCTGCGTCTTCAGAAAGCTTCGAGCTTAATACACGTGTACACGAAAAATGCCTTCGGAAGTTGGCCTCCAGTGCTTCAAAGACCAATGCATACAACGTTTGTCTTTGTCGGCTCTTCCTTGCGCTTCTCACTGAAAAATGCAATCGGCTTTCCTTCCTCAGCACCGATCGGTACTCGTAGAACGATTTGACTTTTCTATGTATATACGAAAAGGATTTATTTGGAAAAGTCTCAAGTGAAACCTGAAAGTGGAAATGGAAGCAGATCCGTTTCGAGTAAATGGATACTCTGAGATAGAGCGAGCGGCAGTATAGACATAAAGCTATGGAGAGCTTAGGCAAGTTTAAAACAGAAATCTTGAAAACTAGCAGTCTCTTTTAGCAAAAAGGAGAATCTTAAAGCTTGCCCCTCCTTTTGTAAGTGTATGGATGAACTTCTTTTGCCTTAATGACACTAGCTAACCAACTGAGAGCTACTCTAGATGTATTAGTAGCGGAAAAAGAGGAAAGCCCTTGCAATTACCTTATATATGCAATTATCAGATCCTTCTTTCCCTTCTTGTTAAGACTGACACCTATCATTGACGAGCCTTTGAAGTTACTAAAGATTTTACATAGATAGACGACTACAGCGACTATTCTTTTTCTACAGGCAAGTACGCTTTCTTCGTAGATGAAGAATGTGGAGGTGAGTGAGAACAGTCCCTCTCAACTGGCTTAGGCGAGGGTCTGTCGAATGGGCTGGTTGTCGGTCCGAAAGGAAATGGATCGTCGCTGTCGATAGCTGATACGGTGCAGCTCAACACCACTCCCTCAGCTAGAGTCCAGAATCCTGGGAGTGGGAAGGCTAATGGAGTTCCCGCCTGTTAAATGGACCAGACGCAGACCTCTCGAAGGGATTTGATTCCCAATGGCTGGGCAAGGCTTCATCGAAGCACTTTTAGCTTTCTGAAGGCCAAGGAAAAGCCAAAACCCCGTCAGGGAACTGAGTAACCTAAGCCGAAAGGTGGATGTAAGGTAGTTACAAGTGAGATAGATGTGTAACTAACCTCTAAAACTAAGCCAAGGCCTGCCGATTGAAGGGTAAGAATTCGATTCTAAGTCGTAGAATGCCCATGAAGACGGTCTTCTTACAGAATCCGCAGTTTTGAAAGTAGCCCTAGACAGATCATTGCTAAGAGCAGAGAAGAGAAAGCTTTTCTTGCTAATCACTAATCAGGTGCTATCATCGTATTCTAATAATTATCCTTTTCTGTTGGAAGAGTTTCAAGTGGATTTATCCAAAAGGTGGGAAACAAAAGGGTTGGTGTGGCCACAGCAGAGGTTTTTCCTTTCTAGAGCTCGCCTCCGCTTCACTGGAAGGCTACCAAAGGGATATAACCTGTTAAACATTCCTCTGCTACTATTGATACTGTTACAAGAGGGAATGAAACTACTGCTACGAATGCTGATATTGATATGAATACTGCAGAGACCGCCCCTGTCTCTGCCCTCCTAATAGCTCAATTTTTTTCCTGACTTGTTCACTTGATGAGCTTACCTTAGCATTGCTGTAAAAAGGGCTTTCTAATGTAATAGCTTACCCCGAATACGTTCAACCAAAGGAAAAGACCTTTGTTGAGGCAGGGCGATCTATTATAAAGACATCTATCCCTCTGTTTCCGCCCTTGCTCGCTTGACGCTTATACTGGCTAACCCAGTTAGACTTCCTGGCTTGAAAAGTAAGTAGGTGGGGTGTTTCAAACCTTATCCTTCAAGTGAAATAAGTGCTTAATCTGCAAAGGAAATTGAATAGATGCTAAAAACCTTTTTCTCATGGGAAATCTTTCCTGCTGATAAACCATATTCAGAGGTAGAGGGCTAAACGAAGTCCCGACAATGAGCCCGGTTACTTAGCCTGGCTCTTAGCTTCAACGATCCCTCTAGTAACCCTATTTAGACATCAAAATCGCGTTCAACGATATCTATTGACACCCCAAAGCAAAGTCAGGCACCTACCCGTACCACCTTCCGTCCAGTCCTCTATCCTCCGCCTTTCTGTTAATGAAAGTCCGCCAGAGCAGGATCTTTTTATGAAAGTATTCTTTACATCTTCGGTTTCCCACTTCTCTGTCTCTGGGTTCATCCCTCTCCTTTTAGTCGAGTCATTTCATCCCTCTACTCTAGGAAGTTACCCACGCACGGGTGTGGGACGCGAAGCTAACACAAGAAAGTAGGAGTGACCTGTTTAGGATTAAGGCTACCCACCTTGTTTTAGGAAGGCTACCATCGCCTGCGTAATGTTGAGGCGTAAAGACTCTTTTGGTAGAAAAAGTGAACCAGGCGCTGTAGTAAAGTAAGACCATACCATACTATGAGTATGTATGGATTTTCATATAACGTATATATATGATAATACGATAGTACTTAGAGATAGAAAAACCTAGTTCACTCGCCCTGGCCGCTGGTCTTACAGTCATCTTCTACGAACCCAAGCATCTATAGACCGGGCTGGCACTGACTGTAACGATCCAGAAAGACCGAAACCAGAAACGGAGATTGAAACAGAACCATCAGAGGATTTTGACTCCTCCGAAACGTTGGCTATCCGCCATCCCACTGGCATTACAACCCCTCTGGGGCCCATGCCGGATCTCAGAATCCTAGATAACTTCCCGAAGTTCTTCGGTGCAGGATCTTATTATGCGGAAGACTTTCCAAATCTCTGCACTGATCATTCTATCGAGGATGAGACGGTAGCAGCACATAATAGGGGCGGTTCGTCAAAACATTTGACGGATTAGCTCTGAGCTGAGGCAGTTGCCCCTCCTAGAGTACTGTTGGGAGGAAGACCTAATTCACACGATTTCCTGCTCTTGGAATTGACGTCGGAGCCTTGGAATCTCAGTAGCCCCTCCTCTTCATCGCGGAATGATGGATTCGAATAGAAAGCCCTCCTAGAGCTTTGGGGATATGTGCCTACCTTTCCCTCGGGAGTAAGTAAACCGTCCACTCACTGGCAGGGATAAGCGAATCCCCACAAACATCTGTACCGAAGCCTTCTTCCTTAGTATCAGTACGCCCTGTATAACCGACCCTGGTACTCAGTTTCACAAGGTACCTTTAGTTCCACATTAAGGAATTGATCTTAAGGGTGGTAGCTGTCAGACGAGCCTGCACATAGGCTAATTACTTTTCTATTCGATACGGCTGTTGGAAGCTTCATTCAGTTAGGAAGGCACAAGGTTGACACACAGTTCATTGATAAATAAACCATGTCACGAGTTTGGCTAAGCAACTAGTTAGTCTTCAATCAGGACTGCCCTGCTGCAACCTAGGGATCACCTGGTGAATGGACGGTCCACCTAGCCAACTAACACTGGAACAGAGGTCGACCGCATAAAATAAAACAGAACTGGTTGTGATTCCTGCCTTCGGTGATAGAACAATAGACCCCTATACTAATCTAAGTTTAGGAAACTCTGCCCTCCCCGACTTACTTATAGGTTATATAAGAGAGAGCGCTTTCTACTGTCGATACACCATAGCGAGAAAGAGCTGGCTTTATGAGAATGAAGGGAATTTCTCTTCCTCTGTCGAGCTAATAAATTCCTCAAAAGCAGTAAAAGTGAAATAAGCTCATGAATGCCAGTAGTCAGACAGGGCAAACAAGCCTTGTCTTAAAGCTTCAAGCAAAGACGGATTGAAACATCCCGTCCTGTCTTTTGTCTCGTTCATTCATCTCGGAATGCTGACTTCCTTCTACAACTCATGAACAGCATTCGAAAGGGCCTTGGCTTGGTAACGTAAGATAAGATCGTCTTTGGCACTCATCCTTGGCTTTCCACTTGTCTCAGTAAAATTGCATTTGCTAACAGTAGTCCATAGATAAAATCAGCAAGAAGCTAAGCGGGTGGAAGTCCAGAATTTTGCTTTTGCCATTCGCCGTGAGAGGTGGTTTTGATGAGAAATGCGGAAAAAGGCCAGATCTTGATTGGTTTTGAGCCTCTCTTCCTGTGACTGATAGGACCTTATCCTGAGAGAGAAAGAGTAGCCGTCCCTTTAAGGAATTTCAAATAAGGGTGCAGATAAAGCAAGGAAATAGAACGAAAACGAAAGGAATGGATTTCGCTAATGAGATGGAGTCAGCATTAGTAGAAGATGAGCAAGGGAAGTCACGCAGTTCATCACTGAGATTCGCTCTCAAGCAATCAAGAAAGAGGGTTGGTTAGGGCAAAGCATCCGAGGCATTTCCCTTAGAAAGTGGGGAATATAGAACCTATGAGGGCTAGCCAAAGGTTCCGAGAGAAAATAAAGATTTGTAGATACTATATCCCGGAACAGAGCAGATGGATGAAATTCAGGGTAAAAGGAATGAGCAAGGGCTTAGAACCCATTCGACTTGGGCGAGACATTAGATAGAGTGATGAGCCTGTTCGCGCTTCGGAGTAAAGAACCAGAACTAAGTAATAACCGAAGGTACTCCTGACATTCGTTCTTATTCTTAATAGGCGGATTGCTTTACCGAAACAAAAAGAAAATCGAAACTTAGAAAGGTCCGCCATGCCTTACTCAACAATTCTGTAACAAAAGAGATAGGCACCTATTATAGATATAGATAATTAATGGTTTAGCTGTATTGCTAAATCAGTTCCCTCCCGAACACTATTCTTTCAATGTATTGCTATTCTTCAGCCGCATTCAAGCTACGGATCGGTAAGAAGATTAGTTTATAGCTCCCTTTCCCCTTTGTCCGCTCCTTAAGCAAGATCCCATAAAGGAAAGAGTCCGAGCCGAGTTAAGCCTAGTGATATCTATGATTGCAGCGAAAACCATGCTTGTTGCAGGGAATCGATCGGGGTTGACGAGCCTGCGGCTAAGAAAGGGATGTGCTTGCGTCCTCATCAACAGGAACGTCAGAAGGCTAAGAGCTTTTATTCCGTAAAGACTTCCTTCTTATGTCTATGTCCATCGTCTAGTGGTTTAGGACATCTCTCTTTTCAAGGGATTCGAGTGCGAGAGCAGGAAAAAGGAATGAATCGGTTGATAGCCTTGACCGGGATATGGGAACTAAGCAAGGGATACTAAACTCAAACAAAAAGAATGCCAATGTGAGCATTTGAATTTCCCATCATCTACCCAATTGACCTATTCGATTGATTTAACAGACCGTGTTACTAATGAAGAGTTCCTTATTCGAAGGTCCGCTAGGATTCAGGCTACCCCTAAAGGCTATCCTACTTTCATGATGTAGTTGCCGTTGCGGTACTCCACTTTGTCGAGATTTTGTTAGTTTTCTTTTAGTGATAGTGACAGATCTGAGATAAAAACTAAGGATTGACAGATCAAGGTCTGGTTTTGAGAATCAACCTTTCTTTTGTTCCATTCCAGGATAAAATGCCATGCCACTCAACCAGAGGCGTAGGAAGGAAAAGCGCTCTCTAACTGATAGTTGAGCTCATTGAAGAATGAATCCAAGTGCTGGTGAACCGGCCTGTTATTGGCTTATCTTGTCTATCTCCTCCCTATCGCCCCCATGCGGCCATGTCTTTTATTTTATGTTATGAAGTATTCCCCTCTTTCTTTCTTTTATAAAAACTAAAGAAGTTCGAACAATTTCCTTGCTTTCGTAACACGTGTTTGATGCAATAACCGCAACGAGAGTAAGCGCTGTTGGATAGAAGACTGGTATAGAAGTGGGGAGCGTAGCCTTGCTATGCTAAAGGAATGGATTTCACTCTTTAGGGAGTGATCGAGGGTGATATCATAAGCTGTTGTCGGAATAACTGGGAATACCATAGGTAAGAATTTATCCTCTTTCGCCGGAATAAGAGCTTTTGTCGACTTTCCTTTGTCCTTTGACATCGAAAATCGGTTGTGCTAGAATTAGATCTACCCGATAGTTCCAGAGTTCAGGACTTCTGATCAGTGGGCGAATAATGAAGCTAAAACACACTTTTCTTGATGACTAGAGCTGGCTTTAAACTAACTGGAAACCGATATCCAACTAGCTCTCTCTCTCTACGGGTCCCTTCTTTCTATAACGGGGTACGCATAAGGAAAGAGGGACTATCGCTTGAGAGCTGTCGTACGGGACCTAGGCACTTGCTAACGAGCAGGCGGGATCAACTAATCAGAGCTAGGACCTCCTTTGAATCTTTGACTCAGTCAGCTCTATGCCCTTGTCTTGGAGCAAGAAGAGCGGCAAACAGCCTTCCTTCACATTGTTCCGCTGGTCCGAACTACTTACTTTACCTTTTCGGCTCGTGAACAAACTATCTCTTATCCGTACTCCTACAGAGTTACTGGTCTATAAACCTTTAAAAGCATTAGAGTTGCGCCTTGGGCAGCAGGGAAAGAGAGGATAGGGGCTCAAAACCTTGAATTGGAAGGTCGACTGGCTTTTCAAATCTATGCCCACTATCAAAGTCAGCAACCTTTGAGAATAAGGAGTCCCGAACCGAACCCGCGGGATTCGAATTGAATTCCTGGGCCGATTATAGTGCTGATTAAGCGAATTTCCTATAGATTGAGTACCCGAAAACTAATGATCCAAATAGTAACTTACAACTTGGCCTAATAAAAGAAGTCAAGTTGCGAGCTACTTGACTTTGTTGATTCGGACTTCTCCTTCCTTGGCTTGAAAGCAGTAAAATTTCATGTGTGAAGCATAGTGAGAGTGACACAGATCCCTTTTTCAAATAGGGAAAAATCCTAATCATCAGGTAAGACGGGATCATTAGAATACGAAACTGCCTTCCAAAATGAAACTGCATCAACAAGAGCAATCGTAGCTTATTCATATCAAAGAAGGAAAACAGAAAGAAAAAGTTTTTCTACGAAGAGGAGGGAAGAAGATGGCTCAGCTCTTAGCCGATCTTTTGATTTTCTTTGCTTTGGGCATTATGAGATAGTTGAAAAAGAGCCTAGCCCCTCCCTAACCTATGTTGAACAGATGCGCGATCCGCTCGAAAGAAATAGCTCGAAAGAAGTCCACCTTCGAAGCGTAGGAAAGGCTCATTCTATAATAGTAGTTAAGCGGGAAAATCGGAATAGGAGGCCATTAAAGGGCCTGCCTCCTAAGCCTAATAGAAGAAAGAACAGACACGGGTTCCATAGTTCTTAACCAGGGAAATCGATCGAAAGGGTCTACGACTGCAGGGAAGCAGAGGTCAAATCCTCAGCTACACCCTTAGATAGAACTCGTGTAATAGGAATACCTTACACGAGTATAAAGGACCAGCCCAAAGAAGCAATAAACTGACCTACAGAGTGAGTTTAAACAGGCGATAAGCCGACTACATTGCTACCTGAAAAGCGAGTTTACCAGTCTTCGTCTCCGGATAATAAATAGAAGTTTGAGTTGCTTTGGCTGTCGATCCAGCTGAGAGCATATGATCCAGGTGAGCTTACTTTAGGGTCTTTCTTGAGCTACCTTTAAGCCTGTGGGATAGATGCAGTTCCAACACTTTCAGTGCCTCCTCACTTTTGGGGCATGCGAGAGCTACTCATAAGCGTGGCACTTTTCTTTCTACATCCCTCGTGCTAAAGCTAGAATAAGTCACCCTGCCAGAAAGGATAGACCCGAGATTTTCTCTCTCATAGGCCGGAGCAACCCTTGCGTTGCGTTTATTATAGCGCTCTGACATCTATTGGATTGGAACCAATATCGACGCTACTTTTCTTCAAACAGCAGGGCGTTTAGCAAGAAGTTCCTTAGCTTATAATATAAAATCAGTAAGACATGCCATGTTCAGTCTAATCTCCCTATAGGTCTTACAGAAAAAGTATGAATAGTGTCTGAGCTAATCGAATTAGGATCCCTTAAATAGTCAAGATCTTGCCCCTATGTCAGTTGGATTCGTGAAAGAGAGCAGCTCCTTCTCCAACAACCGAGTCTTCTTAACTGACTTCATCACCAGCAGCGGATAGGACCAGAGCAGAGCTTCTATTTACTCAAAACTCTTACTGTAACAGAAAAGACTTGCACCGGTTATTCCACAGAAGAGAAAGAGTCTCATGCTGACCCTTGACTCTCTTCATGAAGTTGGTGGACTCTCTGTCCGTGCTCTCGTCACCATGCTTTATGTCGGTAGCTGTCTTGTGTTCTTTGGCAAGGGCGCTAAGGAGCCCATATGGATCGTGACCTGCCGCCCGGTCTTAGTCAAAATAATTGGTAGTATTATTCTTCTATTTTATTCTAATAAAATCTCCAACTGAGTAGTGAGTGTTCATGCTTAACACATGCGATTAGAAGTCGGAAGACCCTTGCTTCTAGTAGAACGGGAAATATGGGAATAGTAGATTATTATTCTTTAGTCTACGTGAGTCCCGTTAGCTTCTATGAGTCAAAGGTAGGACGAAATGGCTAATGGAGTGAACGTTGTAAAGCCCCGAGGTTTCCGGTCCGAGATCATCGGGAAACATAAGAAGTGAATTTATAGGAATTCTAGAAGAAGTCGGTGTGCATAATAAGAAAGAAACTAAGATTTTGCTGATATTCGCAACTCCTTTCAAGCCTTTAAAAAGTCCTTTAGGCAAGTGCAAGCTAAGAAGTCAAATAGGCACACAGGAGGGACGGCAAGGCAAGTCTTTAATTCAAATGAGTGCTTTCTTTCCCTAGAACATCTTTCCCATTTATATTTATATTACTCCCTGGCAGGTTGGAGTGCTTAGATAAATCTATCCAGTTTCAATCATAAGCTGAGCGCATCCTGTGAGCTAGCCAGTTTTCAAGAGTGTAAGCTCTTTCTCAATAAGCAAGCTAAGTGCTTTAGGGAGTTTTGTAATGCCTTTTCTATTAGAAGAAAATGATTCAATTCAATATGCCCCGCACAGGACAAGCTGTAGTTTTTATAGGAAGCTAGGGCTAGCTAGCAAGTCAGCAAGACAGAAAGATGATATCTTTCTACCCTTTGATCTTGCTTTTCTTTGCCTGTCCCGATAACCGTATCGAAATCGAAGGAAGCCATTGTATATATATAATTCCTCCTGGGAATCTTAGGTTTATTGTGAGTTATTCGAGACAGATTCTAGTAAGTGAAAGGGAAGGTCAGTACAGTAATTGCCCATGCGTAACATGCATAACCACGGATACCTTCTGATTGAGAAAGCATATCTAGTGCCCCAGGTTGCTAAAGAACCACCATTCGTGGCATTCTTGCCTTGGCTGCATTGCCCTTATTTTCACTTGACGTGAAGAATGCCTTTCAACAACATAAGGGAGGGGGATCCGCAATCTTTTCTATTCAGCCTCCCCACCTCCAGGCTGATCTTCCTAGGAATCCTAACTTGGTATGCAAGCTCAAGAAAGCGATTTACGTTACGGCCTCCGACAAGCTAAAGCAGGCACCCTTCTTAGCTCTTGGTACCTGCTTTCAGAAATTGAGCTCGTTTCTCACTGCTTCATTTTGGATAAAGGGTTCCACTGTAGCCGTGCGGATTCTTCCATGTGTGTTCGTCGACGTCATGGTCGTTGCCTCATCCTTCTTTTATACGTTGACGACAACATTCTCACCGGGAATGATTCTTCTCTTTTATTTTCTTTCATAAAGGAGCTTGGCAACCAATTTGTCTCTTTCATCCGCTGCATGACTTTTCGGCATGGAGGTCTCGCTCCACCTCTGGACTTCGCCTAACCCAAACAAAGTATACTCTTTCATTCTTATCTCGTCCATCCTGCCCTGCGCTACGCCTATATCTCCAGGGAAAAACCTACTCCTATAAAAAGAAGACGGAGTCTCCCTGACGACCGACGCTCTACTCGGGCTTTTGTTCTTGGTCGGAATCTCATTTCCTGGAGCGCTAAGAAGCCAGCCCCTTACTCACCTCTTAATCTATAAAAAAGCCCTATTAGTCAAGCTTTGAAGCAAGCTGCTAGAAGTAGCGCGCTAGCGCTTTACTAATCGAAGATAAAGAAAGGCTCTACTCATCGAGTAGGTTCTTTTCAGGTACAAAAAGCAAATCAACATTTGAGACCTCTTCTGACTTTCTGTTTTCCAGCAGCAATTACACATTCCCTCAGCCTCTGATTACAGTCGAAGTGCCATTGCCCATATATGAAGAACCTAGTTCTTCAATCATATATGCACCTTCCAATTCTTCCTTTCGGAATCAAAGATGATGCGAGGACCCCATCCACCAACTATATGAAATGTTGGCAAGAGGGCCATAGTAGTGACCAACACTACCTTTTCCTGATCATCATGGCTTTTTCCTTTGCCCTTCTTTTTTATGGGAAAACTAAAGTCAAGCGACCTTTCTTCTTGCAGGACCAACACTCTATGTTTTCAGGTCCTTCTGCACTCACTTTGTGCTTTTTCTTCTTTTGAAACATATTCTTTTCTTTTGCGCAGCATTAGTCTTTTCAGACTGCTGTTCAAATCTCTTTCCTCGCCCGATGGTAGAGGTCGATCCCCTATCACCTTCGGTGCCTCCTTGTGGTCCTTCTCTTTAGCTTTTCCTCGGCCTTTAGAGCAAGTTGATAGGCCACTTTCCACATCCGATGCATCGATCTTTCATCTTGGATTTCGAACCTCAAGCCCTATCAAGGAAGGGATTCTTCGTCGGATTCGTTCAAGCCATTGCGAATAAACAATTGGTAGAACTCCGCAACTGTTAGGAGTAGGGGCTTTCTTGTACGCTTCTCTCCCTCTATCTATCCGCGAAGTGAAACCTTTGGAACAAGCTTTGGGTCTAATCGGAGGGTAGAAATTGATCCGCAACCTCGATCGCATCTTGTCCCATGTACTTCCCTTTGCCTTTCCGCTCACGCCTTGCTTGGACTTGATCCCACCAAATGTTGGCATGACCAGTGCTCAGGAATTTCATCTTCGCACACCTCGGGTAGTTCTTTCCAATCAAAGCACTTCTCTACGCTACTTAGCCAATCAAGAAAGTCCTCAGGATGCAATCGGCCATGGAAATCAGGAACGTCCACTTTGATCTCCTTTCTCTCGATGCCCTTCGTTGACCCGGGCGCTTCCTTATCAAACAAAATCGAAATTGGCTGGAACTTCTTCTTCATGAATAGTTCTTCGATCATGTGATCGGCGGAGGTCTTGAACTCGAGTGTTCGAAGTGAGAGACTCTTGCTGCCACCCTAGCTCTTTAATTGTTGCCCCCTAAAGACCCGGCTTCTTCTTCTTTCTTTTACGAATCCGGCAAGCGAAAATCCTTTCTTCTCTTTCGTGCTTGCTTATGGCAGGCCCTTACTCGAGGCCGCGCAAGAGGGCTTCTTCTTTCGCCGCTTCCGCTGCTTGGTGTTCTGATCTGGGCTAAGTACGAAGCGAGTACTTTAACCAACCTTCTTTGTTCTTTGGAAAGGCCTTCCTTTCACCAGGTTTTCCCCTCGCTTGCTTATACATATGATGTTGGATTTGCAATTGAAACGAAAGCCTCACCAGCAGCTTCATCTTCAGGTTTGAAACCCTTCAAAACCGACGTTTTGGACCGGGGACCTAAGCTTTCTTTGCTAAAGCCGTGAAAAGGTGGACGGTTGTATTTTGGCTTAGTTGGACTGGTTCGCCTAATCGAAGAGACCCTATCTTACTTGTTCACCGTACTAGACCTCCATATCATATGTGTAAAGAGCCCACCTCGGATATGAAACTCAAAAGGAGTGGCTAGACAGGCATCCAAAGGATGAGGAGGCAGGCAGGAACGAGACCTTGTGGGATGAAGCCTTCTTCCTATTGCTATTGCTTTGGTCCGGAGATGGCCTGTGACTTGTATGGAAAGGGTAGGCTGCTACGATGTGGCAAAACGAAACTGACCATGCTCAACTTCGATCTAGTAGGGGCAGCCCCTGTGTTCGTTCAATGCTTTGCCGGTCAAACATTTAATATGAATTTCTCTAGTAGTACAGCTGACTGAACACTAAGACAATCTCGATTTATTAAAATAAACATCTTTCATTGCGCTAGGTTTTCGCCAGCTAGCTTTCCCGCTTTGGTTCAGCTACTCTTCTTTTCTTTCTATGATGCTGTTACTCCGATCTCGTAACTCATCAAGAAGCCCACGGAGCGGTTTCACATCAAGAAAGCTCCTTGATCGGCCTCTCAACCGGGACTCCAAGCAGACCTCGACTAAGCTGTACCGCATGTGGCAATCCAATCTTTTGATAAGGGCTACCAAAGATTGATCAAAGGAGAAACAAGTTCTGCCTATAGTTGCTTGTGAGATAGACTTGCCTGTCTGATCATCATCTTTGAGACCAATCGCGTAATCTTTTTATATGTTTATTCGTTGATTACACCACATTCCTATCCTTCCTTTCTCTGCTCTGTTCTGTGTATCCGTGCAAGCTAAAGAGAGAGCTTTGCGCCTAACAGTCTCATTTTCTATTTTCTAGAACTTGTTTCGGATTGAGCTAAGAAAGAAGGCATTGAGCTAATGAGGGCTAACAACAACGGAGAATCGGCGAAGCCGGACTAAGGCGATAAGCCGACTACATTGCTACCTGAAAAGCGAGCTTACGCTAATGGAGTTAAAGCAATAAATAAACCAATACCTCTCCTCTTGCTAAAAGGGGAAGAAAGTACTACTACTGAGTTTAAAGCAAAGAAGGGGATGAGGGAGTTCCAGGTTGGAGCTCCAACCTATAGTTAAGGGAATGCCATCCGAAGCTAACTACGATATTCGAGTTATACTAGTTAGAAGGGCCTACTGATCGGAAGAGGCTATACTACAGATTCGCAAAGGCGCCTCCCTAGTAAGGTAGACTTTCCCATTCCAGGGTCTTCTAATTCGACCACCCTGGCTCCCAGCCCTGACTTTAGCCGATAGTCCCCAGGCTGGAAGTAGGACGTCGAACTTCAAAAGATGATTGCACTTCGCAGCCTTAGGATCCATAATTCCAAGAAAGGCCGTAGTTAGGCGCCGTGTCCAAAAAGATTGAATCTAGTTTTGCTTAGAATCTCAAATATTATAGAATCTAAGTCTTCTAAATACAAATCCATCAAAACAAGGGAATCCAGCCTATTCGGGTTTCTTTTACTTTGAACGAATTCCAGGATTCTATCCAACGGATCCCCCTTTGATTGGAGTTTTGCACAGAGAAGTATACGAGGTTCCGGAGACTTCTACTATTTACCAATCGGTCAACCTTTTGTATAAGTTTCTCTCTATCAATAGGAGCCGAACCAGGGCTAAGTTCATAAAGCCTGACCGATCCCAGTGTGACCGTTGACTTACGAGCTACTAGGCTATCTATAAAAGACAAGCGCAGATAATGAGGATGGCAAATATTTAGATCTATTCTTTCGGCTCCACACTTCGAGAGGTATAGGAAGTGTGCTAAGGATGATTGCACCAATGGATCCATTATAGGATCATAATGCATATAGGCATAGTCAGCTCCGTTTTCCTCGCATTTGATTCCAAAAATTTGGAATATCTGCGGGGGTAACCAGGGAAAATCCGTTGAATTCTCCGTGTAGTTCTGATGCAATAAATCATAAAATGAGTGAAGTTCCGTTCGGTCCATGGCAGATTTGACTTCTATTCCGTTGCTAGTCTTGCGACTCATTTCATTTCCTTAAGTGAGAAAGCGGGCACTGGGTCACTTACGACCTACCGAACAAGTACCGGGTAGTGAATGAATTTCTGGTAGGTAAGATGGAGTAGCCTTCTTCTGATTTATGCTCGGCAGACTCACAATTTACAATATTTTTGGCATTATCTATTCTTAATTACTGCTGAATTTGGTATTATCCTTGAATCTTTAACAACAACTGCAAATAATACGAATCCCACATACCGAAATAATGCAATTCATTTCAATTGTTTAAAGTAAGACCCGATCTCCATGCATGATTTCCTCATGTTGATCAATTTCTCATATTTGACTGAGGATTTTTCCTTTAAAACTTTTACAGATATAGATATATAAAATGAAAGCTTAAAAGATAGAATTTTTTCATATGAAATGAAAAGAAAGAACTTAACACAAACATAAAAAGAATTTTGGAAGGGTGTAATTGAGCGAGACCAAACGAGCGATATAAGCGAGTACCTAGGAAGCAGAAGCTCGACCAATGGGTAAGAGCTTAGTTGTAGAAGCGAGAGGCGGAGAGGGGTGAAGTTTAGGAAAGAAGATTTAGAGGGCGTTAAGCAACCTTTATTGAAGAAAAGCTCTTGCTTTCCACTCAAACGGAACAGAACGTTCCTTCCGAAACGCAAAGCCGATATCTTTCGTAGCAGCTAAGGATGAGTTCGATCCATTAGGTTCTTCGATTTGTTCAGAAAAGAAACGAGAGACAAGAAAACATCTTTTATTACGATTAAAAGGAACAATCTCAAATAGACCAAGATCCAATTTCGGGTCATTTCTTGGTGAACATGATCTGCCATAATTTCTTCGATCCCTTCATTTATGTGCCAGAATAGAGAGAGATTTGGTAGGAAAGTGGAGGAAAGTTTTTGTATATAATAATGTTAGGGAGTGAAAGCTGCAGTAATTCTTTTGAAAAGCCCGGTTCTCTTTGTCTTCGAGCTTTCATTCCTCAATCCACTGATTCGTTCCTTCATATACCTCCCCACCAATAGATAGAGACAAATGGGAATAACCGAACCACGTCTACAAAATGCCAGTACCATGCAGCTGCTTCAAAGCCAACGTGATGCTCCTTGGTCAGATGACCAAGATATTGGCGAATACCACATATGATCAAGAAAAGAGTACCTATAATCACATGAAAACCATGAAAGCCAGTTGCTAAGAAAAGGTAGAACCATAAATACTATCAGAAATCGTGGGGGTGCTTGATAATATTCCATTCCTTGAAAGCCAGTGAATACTAGAGCCAGTGAAACGGTAGCTACTAAAGCGTAAACTGCTCGTTTTTCCTTCCCCGCGAGTATAGCATGATGAGCCCAAGTTACGGCTGCTCCGGATGAAGGGGAATAAGGGTATTAACAAAAGGTATTTCCCAAGGATCTAAAACCTCAATCCCTTTTGGGGCCAAATACCTCCGATCTCTACCGTAGGTGCCAAAGAAGAATGAGAAGAAGCCCAAAAAGAGCAAAAGAACATAACCTCCGATACGATAAAAGAATAAAACCATATCGAAGCCCTAATTGTACGATTTTGGTATGATGTCCTTCCAACGTGGATTCGCGTAGAACATCGCGCCACCATACAAACATGGTATATAGGAGAAATAGGAGGCCCAAACTGAAGTGTTGCACCCCTTGAAATGAGTGCATGTACATCACACCTCCTACGGTTGTTGCCAAAGCTCCGAGTGAACCCGAAATAGGCCATGGACTTGGATCTACCAAATGATAAGAATGCCTCTGAGATTCAATCATAAACAACTTTTCCTCGGTTGTATGTAAACCCCCTTCACCCCACCCCTAAACAAAAGTAGTAAAGAAGGGCTCTTTGGGGTCTAATTTTCTTTCTATCTGACAAGACAAACAAATAGGAAGGGATGGTTCTTTCATTGCATTGATAGAAGTAGAAGATCTTTATATTACTTGACTTTGAGAAGAAAATCGTTGGTTTGCCCGACGAACTACGTGGGAAAATGGACCTTCTTTCTTTGTTTAGTTTAAGGGATAAAACCTTGTTGTTGGTGACGATTCTAAGAAACTAAGTTGAGAACAAAGTGGCGAAGGGTGCGTCAATCCCTAATTTTCCATGTTCTCCTTGCTTTCTATTGTCCGACCCTATCCTACGCTATCTCAGGAAATCAAACCTTTAGTGCCTCTCTACCTATCTTTCGGGAAAGGCTTGACTACCCGCCCTATCAACGAAAGCGACCAGCATTTCTCATTCTCTTTGTTGGCGCATTACGATTTCCTCTTCCAAGCCCATATCCCTAGCGAGGCTGAGGTGATCTCTGGTTGAGGCTGCATTCATTTATTCAACTTGACACGTGGGAAAGGCTTACTCCCCTAGTGGCCCGCCTTGGTCTTACTCCCTTCCAGCACTATTCCTCCCCACTAAAAAGAGCGATTGAGAATTTCAAGAACCTTTCTGAACGTCTGTCTTCGCGGGGCGATTGGAAAATCTCTTATTTCTTGTAGTGCCTTCCATTCTGATCGAGAAGAAATAGAATCCGAACTACAAAGTCATAATGAGATTAAAAGCGATGCTTCCTTGGCCGTGTGGAACATGGATTAGCATTATCTCATTCCTACAAGTGATCCCCCATCCAGGATTGGAAGAAGTGCTATATGAGGACTTCGAGATCAAATAGAATATGTTTCTTTCCATTCCTCGTGAGCCACTTATTTCTCCGAAACAAGAGATCAAAGTCATCTTCCTCCTTTTTCCCAATAAGTCGACGGCGTTACACCATTGGGATACTTCCAATAAACTGGAGTACATATAGGATAGACCGGTGCTAAACCCTTTCTCAAGATATCTTGCATATAAAAATCCTTGCTCCGGATCTTTGTCTCCCGGTGTGAAAGCAGTTGGTTTCGTAGTTTTAGAATTCTGCTGATCCCAAGTACTCCATCTCCATCATTGCATATGGCAATATAGAAAGTAAAGAAGAAAAGGCATAACCAGAAGAATTGTGTGAAATAAGTGAATTGATCCAGTTGAGGCATGATTGATTGAGATAAATGATTCTCCAGACAGCTTAACTCCGTAAAGCCTGTAAGAGTAGTGAAGAACTATAGTGAGTTGGTTGTTGACCAACGGAAAGCATGGGAGTAAGAAAGATGCGCTAAGGATTGAAGAACTTAGTGTAAAACTAAGGAAAGGGTTCAAATCCATTTTGAAAAATCTTTTATCTTTCTATACGAAATTTGTTCGCCCCTTTATTTCAGCGAGACAAGAGAGGCAATGAGAATTGTTTTATGACCACATACCATACTTGATTGAAAGCCCAAGGGAGAAGCGAAAGCTGGATCGACTCCAAAGTAGAGTAGCTGTACTTGCCTTTCTTTAGAAAGTCCCGAGTTGTTCGCTAGTTGAATGAATCATTAATCGCACTTGCGCGACTAACCGCTAGCTTCCTTGCTTTCATCCCTTTTCTTGCTTGATTTCGTGATTCAAAGGAAAAGGAATTGACTAAGCTGACTGACTTTCCTGATTACTTCTTTTCTGCGCTATACTTTTCCTGCTTGGAGGAAGAGAATCCGCTGTTACAGACCCGGTTGTGAAAGAAGGCAAGTATACTGACTTGGCTCTTTTAAGGACAACTTCCCTTGTTAATGTACGAGCAGGAGACTAGAATAAAGATGCCCACAATCAGATGCTATAGGAACTGAACTGCCAATATGTATATAAAGATATGTTGTATGAAAGGGATCGCCTAATGCCCATATTAGCGAAATCTGACTCAATGGGAACTTCACTCAAAGCTGGGGAAGGGGATGCCCCAGAAGAGTCCTTTCGGCTAGAACATCATTAACGAAAGAGCGATCTATGCTTTACGAAAACGACGGAAAGTCAACTCTTTTTTACCTTTCTTTCACCACGGGTGCTAAAAAGAAATTTTTATTAACACAAGCAATAAAGGAAAAGAGAGAGATGGGTTCAGGCCTGCCTCAACGGCATAAGATGAATATAAAACCAAGCGCTAACTGAAACTGGGGTCCTTATCCTTAAGTCAGGTTTTTACTAAATAAAGAGGGCCTACGAGGCGCCTGGAACAGGTGCTAATGGAACTACTGGAGCGAGTACTACTGGTTGAACTCTTCTCGCTAAGACTGCTACTACTCTTCCAGTTGGAGCTACAACCACTGAATTGGCTAAACTCTTACTTGACCTGGTACAAAAGCTAGGTCTCGTGGGGAAGCTTATGGATACCAGGGGCTTTCTCCCTATAAAATCTTTTGCCGATAGATACCATCCTTATCCAAAAGATGGTTATATTCTAATTTTTTGATTAGCTTGAAGTGAATTTCCAACCTTACTCAAATAGGGAATAGATCGCAGTTCTTAAAC

>Decalepis_hamiltonii_chloroplast_contig8

TTTTGATCCTTCTGACCCTGTTCTTGATCCAATGTGGAGACAGGGTATGTTCGTTATACCCTTCATGACTCGTTTAGAATAACCAATTCATGGGGCGGTTGGAGTATCACAGGAGGGACTGTAACGAATCCGGGTATTTGGAGTTACGAAGGTGTAGCTGGGGCAATATATTGTGTTTTCTGGGTTATGCTTTTGGCAGCTATCCGGCATTGGGTGTATTGGGATCTAGAAATTTTTAGTGATGAACGTACAGGAAAACCTTCTTGATTTGCCTAAGATCTTTGGAATTCATTTATTTCTTTCAGGGGTGGCTTGCTTTGGTTTTGGTGCATTTCATGTAACAGGCTTGTATGGTCCTGGAATATGGGTGTCCGATCCTTATGGACTAACAGGAAAAGTACAACCTGTAAATCCAGCATGGGGCGTGGAAGGTTTTGATCCTTTTGTTCCGGGGGAATAGCCTCTCATCATATTGCAGCAGGGGCGTTGGGCATATTAGCGGGTCTATTCCTTCTTAGTGTCCGCCCCCACAACGCCTATACAAGGGATTGCGGATGGGAAATATGGAAACCGTCCTTTCTAGTAGTATCGCTGCTGTCTTTTTTGCAGCTTTGTTGTTGCCGGAACTATGTGGTATGGTTCAGCAACTACTCCGATCGAATTATTTGGGCCCTTTCGTTATCAATGGGATCAGGGATACTTCCAGCAAGAGATATATCGAAGAGTTAGTGCCGGGCTAGCAGAAAATCAAAGTTTATCAGAAGCCTGGTCTAAAATTCCTGAAAAATTCGCTTTTTATGATTACATCGGAAATAATCCGGCGAAAGGGGATTATTCAGGGCGGGCTCGATGGATAACGGGGATGGAATAGCAGTTGGATGGTTAGGACACCCCATCTTTAGAGATAAAGAAGGACGTGAACTTTTGTACGTCGTATGCCTACCTTTTTGAAACATTTCCAGTCGTTTTGGTAGACGGCGACGGAATTGTTAGAGCGGATGTTCCTTTTCGAAGGGCAGAATCGAAGTATAGTGTTGAACAAGTAGGTGTAACTGTTGAGTTCTACGGTGGCGAAGATGCTAGGAAGGAACATTCGAGGAGGCCTTCGCTGAGTTCGGAAGGAAGGGACCAACGACCGGCGGTGATCTCCTGGGGCGCCAGCTTTCTCGTTCCACTTCTTTCTGAACTCATACAAGATTTCACTCGAGTTCAAGCTTTCGTCACTTAGTCAGAAGGGATCACCTGGGCATGTCAAAGAACCGGCGCTACCAGACCTTGGTCGTCTCCGCTGGGCGGGGCTCACCGCAAACCGACTTCCTCTTGGATCCTTTCCCACTTTCCTGAATCGAGCAAGAAGCCATCTTTAGATCTTCTACATTTACCGAGGCAATCTCGACGAAAAGAGAATAAGACTCAAGATCACAAACTATATCTCGTCCCTTTACATGTTTCACTTGAAATGTGGAGACTGTTTCTGAACCAGAGCAGTCAATTGAAAGAAGAAAGCAAAAAGCACTCTCGTTGACCAAGAAAAGAAAGAAGAAAGGGAGAAGAGAATAGGCTATTTCTGAAAGGGATATGCCATTTGTCTTCCCTATGACGAGAAAGCCAACTTCCTCTTCTCAACTTTCAACTTCTCTTAAGTCATTCTTTATTCCATTCCTCCATTCCGGACGGAAGATCCTTATGCCTAATAGCCTGAAAAAGTACTTTCTGTATTTAGTAATTCCTTTCTTTCTTTCTGGAAAGCTGGCCGAAGACCTTTTTTTATTGTAGGAGCTGCATGGATGACTGGAAAGCGGTAAGGAAGACCTTTGCCACCAGAAAGCCCGTGATTCGATTCTTATTTAGAGTAGGATTCCCGATTCCACTGAAAGAGATGCTACTTCCCATCCTGGGCTTTATCCGTTCGCTGAGCACAACAGAATACAGAGGAATGCATTTCAATCATTGAAAATTGGAATTCAAGATAGCCTTGCTGCAGCCGGAGGCCTTTTATTTTACTAAAACGAGAAAAGAAAGACCAAAGGGCGTAAAGCCAAGTCTCGGCTAAGACCTCGGGATGGGATATTATGAGGTTTAGTGAGGGGATATTATTTTGCCAGGAGATATGTACAAGTAGTTGATCTACCTGACTCCGCGATCCCTGAGGGAGGTTGGGCAAAGCCTAATGATCCGAACCCTTTCTAGTGGAATCTGGAGAAGAAGCTTTCGCGGAATACAATTTAGTCTTAACACAGAAGGCAGTGGCCATTTTCAGAAAGAAAGACTGATATGGTTGCAATGTAGAGATGAAAGACCTCGTTGACCTCTTTGTCTACGTCGGGCCGGCATGAGGCAATTGGCTGAATAAATAGCCGTAGGGCTCGCCGACTGGCCTTTATCGGCGCTTCTATCGAGTATGAAGAAAATGGGGACACCCCAAAACCAGGCTTTCTACTAACTATTAAAGAGAAAAAAGAAGCCATACAGAAGAACTAATGGATCGAACTCATCCTTGGCTGCTATGAAAGATATCGGCCTTGCGTTGCGAAAGGAACGTTCTGTTCTGTTTTGAGTTGGATCACTCTCTTGAAGAAGGTGCTCCGGGGAAGGAGGTGCATTGACCTTGTCAGTTCATAACCATTTCTTGTCCAGGGGGACTTGGGCAAAAAGCATCTTTGGGCGTAGGGAAGCAACTTACTCTGTTCAATGGCATGAAGGAAGTAGGGTAAGGCATTCTTCGGAATCTAGTAAACTAGTAAATAAGTAAGCATGAAGCTTTGGCATAAGGGTTCACCAGGTGCAGATCGTGAGTAAACCGAAGATCCATACCCATTTAGTGATGCGGAGCAAGGTCTGCTGTCATCTCTCACTATACACCTTGCTCAGATGGGCGGATGGGAATAATGCTTTCTTCCCTCGCAGCTGGTCTAGGGGCTGGCCAAACCAAGCCTTCGATGCGGAAAGCAAGCCAATCTGTGAGTCATTCAAAAAGTGGTGAAAGGAAAGGGCTGTGCCCTTCTTCTTCAGGTATGAACCAGAAAGACAGATTTGCGCCTATTTTCCTCTTTAGTTTTCGCCCCTGTTTGTAAGACCAACTAGCGCACTCGCATCTCTCTCTTGTGAAGCGTGAACCTTTCCTATAGATGAGTTAAGATAGAAAGATATCCGTTAATGTGTGTTTTCTTTAGCGGCTTTTCTCCTCAAGCTCTTCCATTGGAGCTAGCTATAGAACGCACTTTCTTTTTCTATACTTTATTTACTTACTATAGAGGCGGGGAACCTGCCAGATCTTCCGCGAGGATCAATCACAACTTCTCCAGCTCCCCACCCTGTACCCATCTTGCTCTTGATGCATAGCGGACCCATCCAATTCCAAAGTCCACGATCCTTCTTCAATCTCTTCTTCCTTTAGGTTCCAATCCCATGTCTTCTCTTCCTCGAAGATCATCTCTTCCCAGTTGTATAACAACTAGCCTTTTGAGCTTGAATCGTAGCCGATGAATATGAGCTTCTCACTTCTGTCATCCGACTTGGTTCTCTTTTGTTCTGGAACATGCATGTATGCGATACTCCCAAAGACTCGTAGGTGAGATGTTTGGCTTGTGACCACTCCATGCTTTTGTGGAGTCATATTCCATATACTTCTTGTTGGAGAACGATTTCTTAGATAGACTGCAAGCTACAGCTTTGGCCCAAAACTCCTGAGGCATTCTCTTGCTCTTCAACATGCTTCGTGCCATATCAAGAATTGTCCTGTTCTTGCCTTCCGCAACTCCATTTTGTTGAGTTTACCCTCAATTAGGAAA

>Decalepis_hamiltonii_chloroplast_contig9

AGTACGAATGGTTTCTTTTTCATTTTTAGGAAAGAAGAAGAAAAAAAGACTTAAACCCTAGTCTAATTGATTTGATGATTTTATAGAACCTTTTGTCATTTATGTAATTTATTCGATCCATACCATAGATAAAACTTCAAATCAATTCTTTTCTCGAGCCGTACGAGGAGAAAACTTCCATACGTTTCTAGGGGGGTGTATTGTTTATCTACATCTATCTCAATGAGTCGTCTATCGAATCGTTGCAATTGATGTTCGATCTCGAAGAGAAGGAAAAGATCTTCAGAAAGTAGGTTTTATGATCCGATAAAGAATCAAACTTATTTAAATGTTCCCGCTATTCTCTATTTCCTTGAAAGGGGGCTCCTCAGGCACTGTTCAGGATATTTTAAAAGGGTGGGGGTTTTAAGGAACTTGATCTTAATCAAACGAAATTCAATTAAGGAAATACAAAAAGGGGTAGTGGTTTGTATATAACTTTGGATAACCTTTCTCTACTCTTTTTATTTCTCCCCTTCCTGCTCTATTCCTATCTATTTATCATTGTTTCCGTGGAATCTTGTGTTCTATAACGACAAAACCTTATTTTCTTGATCCTAGAAAATTTAGACATTCTCGACAACTGGATGGTTTTGATTTTTATTTGAAATTTCACTAAGAAAAGTGGGGGTACTTTTTAACACTCCTATTCATAAAATGGTTTTGAACCCCACTAGCAGCCGGGCAAGATGGCGGACATTGACGCCGCTCACCTACATATTTCATCATACAGTTGACCTTCTCACTCCCGGCACACTTTCTATATCAGACTTTCACTAAGTTACTATCGTTATACGCATTGAACAGTTAGCTAAACAAGAATAACAATTACATCCATCCTCTTTCGCTCCTCTCCTCCGGTCGAGACTTTCAGACCTACTCGTAGTTAGAAATCGGATGCTGCCCTCTTAAAGAGGCTGATTAGCCTATTCTTTCAAAGCGGAATCTATACTAGGAATATTCTGTAACACCGTCTATTGCAACAAGCCCATCAACCGCGGATACGACAGAGTAGCACCGCTTACTTAAGTACCGGAATCACTAGCAACGGCTATTCAAGCACTGGCTAAGAACTCACCATATGGAGGAACAGGAGAAGTGAAGGCATATCAACATATCAACAAAAGTCCCACAGCTGTTATTCCGCACAGTCAAGGGCTTTGCCCTCGTATAGAGAATATCTCTTAACAAAGAGCAGGGGATCGAGAAAGTCAAGGATAAGATGTAAAAGCTTACAGGCTTAACCTACGACTGCTTATAGAGGGCAGGTGTTGGGTAGGCTACCACTGCTGATGGAGGAGCTTTGGGCGATGCTATTGGCTAGAAGGCAGAAGCAATTGAATCCGCAACTGGCTCGCAAACTCTTCCTGCTTAGGTATACAACCTATGGAAAGGCTGACTTACTCCAGCATTGCTTTGATAACTTCACTTGCTTGCTTGCCTTAGGGACTCTAACTCCTAGAAGTCGAACTCGCTCGCAGGTAGGGAGAAATGGAACTCCATGTGAGCTTTCTCCTTTCTTCTTAGGAGAAAGCACACAAGAAAGAGGAATGGCACGGGAAAGAGATGCCACTCTATTTGGTTGGGCAATAACTGGTACTGCTACTTCGTTCCTCCTGCTATACAGGCTTCCACTTAAGATATTCCTGTTTCAACGCCGAAGGAATCAAAAACTGGTAAAGTCAATCATATCCTTCTTCCCTTTCCTCTTTCTTTTACTCAATATCTCCCATCCATGGACAACTCCTTTCCGTAGCTTGCTTGCAAAGGGTTACCTCTAGTCTCCGCTTCTATCTTCCGGGATCAGAAGCAGGCGATGGCGCAAGTGGAGTGAAAAGCTTTTTTGGAAGGAAGGAAGATCCTTGTTTGGTAGACAGATTCTCTTTGAGTTTAGGATGGGATCCGTTGTTTCGGGATAGACTGGATCTCTTGGTTCGAATCCTTCTTCGCATGCTTCCACCAAAACCACAACACTTCCTGTATCGCCATCTCCACCCAAACAAAGAGGGATAAGCAGATGCCGGTAAGAAAGAGATCAAAAGATAAGGAAATGATCCTAGAGAAAGAAGAGAAGGAAAGAAGTGTGCTAAGGGTCGAGGAACAATCTAATAGAGATAGATATAATCGATCTCTTCTTTTACTACTTAGACAAAGCCCCTCATAGTATATCTGATGAGGGGCTTCATGCTCTAATCTCATATAAGAATAGAAAAGTACCGGCAGGCTGAGTGTTTTCAAAGAAATAAGTACCAAGAATCCCTTAACCCTCAACAAAAGGAAAGAGGGGATGTGAAACCTCAACAGACAGAAGAGCACAGATAGAAGACCTCGTCTATCGTAAAGAGGCCTTTACCTGCTTGCATTTTGATCCCCATCCCTAACGGGACTTATAGGCTAGACATAAAACTCACTACACAAAATGGGAACTGAAACTAAAGTCTTAGAGCTGAAAGACCTACTTTAAAAGCACTATCAACAGAGAAGAATCAAATCAATCTCGAGAGGAAAAATTGGAGATACTCTATAAGCAAATCCATTTACTTAAGCTATCGAATGTCTTAAACGAACTTGCTAGTTAGCTTGGCCGGAGAACCAATCCTAGAACCGTTAGCTCATTAGCTCAGCGCTATTAAGGGGCCTCGCTGCTTTGAAGACCTGTGCCAGCTGAAAACAAAAGAATGTAGTAGTCAGGGAAAGGTGCCCTTTATACTAAGCTTAAGCTACTCTTACTCTTACGATATGAGAGATTCTAAGAAAGATATGAAAGTCCCTTCCTTTAGATATTTGATATTCGATTGTGATCTCGTAAAAAGAGTCTTTATTGCAGCTAGTCAGCATTGGTAGTCAAAGTCTTGAACAACTTATGATGATTCATTTCCTCTTAGAATACCCGTTTTTTGGGCAACATTTCATTCCCTTAATAAATAAGCTGATTCAATAGCAGTGGCTTCTGTAACAGCTGAGTCCATAGCTGTGGTAATGCCAATTCTTGCAAAAGAAGAACTCCCCTTACTGTTATACTAAGACGGCATTCTATTCCTATCTTTTCTTCATCTCCTCTTGCTAATCAGTAGCCTGCCTTCTAGGGCAAAATGAAAGAAAGCGCTAATACCAGACTCTACTCGATCAGTCCTGATTCGAACCAGACTCGACAAGTCCTGTTTGTAGAAGAGCATCTAATCGAACCATTGACAAAGTCACTAAAGGTGCCAATGGAAGATATCTCTTTTTCTATCTTTTTCTGATAAAGCTCTTTTCGTCTTTGTTTATGCTTGGAAGTGATAACTAGAATTCTGGATTGTCCCTTTTAGTGTTCCACCGGGGCTGGAGCATGCACCCTATCTCTGTCTTTCTTTGTTTGTCCATTCTAATATGTTGTCCCAAAGCCTTCCATTTTCCTTTCCCTGTAGGAATTCCTTTTCATTCGTTCTTCTGTTGAATGTAGTCCTCTTGAGCTGAGCTCTCTTTGAGGGCGGATTACATACACGATTTCTTTCATTTTGGTCTAGCAGAAAGTGGGAGGTGCCTTACCTCACCAAAAAGGTGTTTACCTCTTTTCCTTTGAAGGCACTCTATGGCTATGTTGTAAGGATTAATCCATTCATTAAACGTGCAGAGACTGTTAGGGAGGAAAAGAGCGGACAAACGGAAACTTCAGCAGCATCTAAAAAAAAGAAGAGGCTCAGGAAGTCAAGCAGTCAATAGGGAGGGGCGAGACCGAAGGAAAATAGGTTACCAGATCGACAGAAAGAGAATCATAGTCTTCCTCCAGGGATTGGTCTTACGCCCCCTTCCTTTCTTTACGAGATCAGGCCAAAAGAAAGAGTAGCACTCGCTGAGAAAAGTAAGCTTGCACATTGAGAGGCTTCCCCGGTCAACAAAGAGAAGGAGACGGCTCGCGCACGTGTGCATATAAAAAAAGGAGAGCTAATGAGAGGAAGGGACGCTAAGGTCGTCACCAATATTATCTGAGGTCGGGATCTACAGTGCTATCCAGGCGGGTCACCCTACCCTAGATAATAATAATAAATAAGTGGATCTTGGCCAATGCAGCCAGCTCCATCAGAGTTTCACTCTGAGACTGTGATTTTGAGTTGTCGCCATTATCTGTATTCTTGGACTCACTCGAAAAAGGGCTGAACCATTCACTAGGCTATTCTAAGCCCCTCCTCGCCTAAAGGCTCTTCATAAGGATTTTTTAATAAGATCCGGAAGAGGGAAAATAAAAGGATACATTTAGACGATATGCTGAAACAGCTTTCAAGAGACCAACTAATAGAACGTATGAAACTGAAGAGACCGGCAAAGTGACTCTCCTCTAAGGCTACAAGGCTACCTTCTTGTGAAGAAGACTGACTTTGCAGGCTTGAGATTAAGAGATTGGCACCTTGGCCCTTACCTTCTTCTTATGATTCTGCTCGAACTGTCTTCTTTAAAGCGAAAGGGACTTTTATGTTACCGAAATCTTTTTGTCAAGTGGGTCTTGATTACTTTAGAAAGAGAATGCCATTCAAATCAATGGCCATGCCCGAAAGATGCGAAGAAGGCAAAGGGACTCAACCTGTTTCGTAGCCTTGGGGTTCTACTTTCCTTCTGGTATCTATTTCTCTCCATTGCTCTTGTCCTTGCCCTTCTACTGGCTTGGCTAGGAAACTAGTCCCTTCTTTCAACAGAGGGAATTCATCTCAGTTTCGCTTTCGCCTTTGGGTTCGGCTTCTGAAGTCGCGAGGAGCGCTTTTATCAGTAGAAAGATATTTCATCTAACCCTTTATTTAACATGGTAACAGCTTTTCTTATTAAATAGTGCACCTTCACCCTTTGAATAGGCCAAACCCAATCCTTATAGATCCACCCAACATAGATATAGAATAGATATAGATAAGGGGTGGTGCTTTTATCCGAGAAAGACGTTTACCCTGGATAGATATTAGTAGATCTGACTTTCGAATAGTTAGTGCTTTTTGACCAGCAATAGTTGGAAAGAGTGCTTCTGCGTTAGAGTGAATTTACTCACTCCCACTTACACTTGCACCTATTGTTTGTAGCCTTGTCTATTACAGTAAAGGAAGGAGTGTCAGGCTACTCAAAGGCCAACCCAAGATCTGATTCATCTGATCGCTTCCTCTAAGAATAGATATCCCTTGCCCGGGCTACAGCCAAACGGGAAATAGAAAGGCTCATTCACGAGAACGAAGGCAGAATCGAAAGGCTCAGAGTCCTTTATAAAGAGAAAATAGACTTTTGGACGAACAGATAGATAGACTAACGGACAGTTACGTCAGTGATCGACTAATCGCTAAGAAAAGGAGGAAGTGAGCGCACTAATAGCAGACTTTAACAGCAAAGCCACTCCACTACAAAGAAGGAGAGAACAATTGAAAGAACAATTAGAACTGGCCCAACAAGCACTAGCACAGGAAGAAGCCGAGGCTACACAGCTGGGTGTTCCTAAACCAGACGAATCAACTAGGACACATAGCGATCAATTCTCTCATCAAGAAAGGCGTCTCCGGACTAACAAGTAGATAGGCAGATATTATGGGATCCTGCTGCCGGAGAACTTACGGAAGTTCGATTCGATTCGATCGTCCTGTTTGGGCAAGTAGCCAATACAGATAGCCACTCAATTGATAGATCTCTTTACGATGATCAAGAGCCCGCCCAACCTGATCTTCCTGTTGAAGTACGGTCGGACGAACCAGAGTTACCAGAGGATCCAGACTTTGATCGAAGATGGCAAGAAGCCTTGGCATTTGCCAAAGAAAGGAAGTGGAAAGAAGGGTCTCAAAATGCTGACAATACCACTGCTACAACCACTGCAGCTCCTTCGTTGAACGAAACTGCTACGGTGAATGCCGCTGTTCCAGCCGTGAAACATGAAGACCGCTACTATTATCACTCCTCAGGGATAGAACTATTGCAGAAGAGACTTGATCAAATCGAGGGTGAGTTTGCTGAGTTCGAACGCCAAGTGTGCTGGCGCGAAGAGCGGGGCATGCCAACAGAGCCAGTCATAGAACGGATCCTGGATCTCGAGAAGGAACAAGAACTGCTCATCCAGCAAGTGAGAAGAGAGGAAGAGCGCTTGAGAAGGGAGGAGTAGCTAAACGAAGATGCTTCGATCGGTGGGAGCCTACGGTTGGGAATGATAGTTGGAGTCAGTACGATCGTAAAGGTGCTGCTGAGACGGCTGGAGAATTTGATTACGACGGCCCAGCCTACCATACGAGAAATGATAGGGCTCCAGTTGATGGATCTCCGGTTTTGTTTGATAATGGGGAAAGGGACGAAGACTAAGGTTCTGCTTTTCCTTTTGGATGCCCATTGGCGAGTGTCTTAGTCAGCCGTAGACCTTCCATTCTGTATTGCGTAACCTATGTTGGTCTATCGGGCTCTGCCCTCCTATTCGAGCAAGGCTAAGGGTTTTTCTTTCTTGGTTGAGGCCCGACCTTCTTGTGTGACTTGAGGCTTGAAATGCTCTTTCCACTTTCTTTCCATTTCCTGAGCCTGAGGCGAGAGCTTGCTTTACCGAGAACTAGGCTTTTGCTTCATCAGATGCTATAGACGAGACCACTTATGTCACTTAATTGTCTGCCTTAGAGGATAGACCTTCTGTCTCTGCCGCAGAATCAGATTTTTTATAGTACTTATTTAGTTGAGTAGTCTCATCTAAAGTGGACGATTGAGCAACAGAAAGAGGTTCGGCGGTGGAAGAGGTTTGATCATCTCCTGCAGACTCCGCGGCACCAGACGCTTAAGGAAGGCACTCTGGGGAAAGATCTTTCTTACTTGCCGACAGTTACTCTCTAGTTATGTATGGGTCTCCCGCCTTACCTCGACCCAAAAAGGTAGCAACCGGTTCCGGGATAGCGGACTCTCTTTCCCTCGTCTTAGTTGGCTAATGAACCAAGGATGAGGAATGAGTCTGTGCCTTACTCTGTGCCTGACACTCCCGGTTCATTAAGAGTAGGCTACTTTGCATTGACTGATTCTTCTGCTACATTTGGGATTTCTCTCTAAAGCACTAATTCCTAGCGTTCATTCCCTTGGGCTAGCTATTCATGTTAAGGGCAGTTGGTGAAACCATTTCCTCACTTCTTCTTATATGATATTTGTTGAGAAAGGATTGGGTTCAGAGTTTAACGAGTACTCTCCTACCCTCTTGATCAGGAGAAGACTGATTATTGGCGCGAGGAGGATACCATTCTGAAGAAAGTCTCCCCATTTCCTCGTAACCGAAGAAGGAAGATGGTTGCTTGCGATCTTTTCAAACAAGTACTTTCCGGCCCAGTTGACAAGTACAGGGAATCCTATTTGTTTTATATCTCAATCTGTGAGAAAGCTCTTTCTTCGAAAGATCTCATTCGTTGTGCAGATTCAAGAAAGTCAGATTCTGAACCGGAAAAGGAAGAAATTATTCTCCGAGAGTAAGGGTTAGATTTTCGATTCAAGGGGTTAGAATATCTCGACTTGTAAACTGGATTATATATTTTTGTTCCTTTGACTGGTTCGATAGGACCCGTAGTTCAGATTTAGTGATGATTCTTATTCGAGTTTATTAAGGAATGATTGGGAACCTAAGGATACCAGAACCCACCAAATCATTAAAGAGATGATTGGCAGTGATGGTCTCCCATGACATACCTTGTGCCTGCACAGAGAGTTCAGTATTCAGATTAATTCAAGTCAACTAAAGAAGGAAAAGGAAACCTATCCACCTCCATCATCAAGAGGACATCGATTCATCTTGGCAGCTACCGAAACCTTTTCTAGATGGGCAGAAGTGCTTTCTCTTAAAGAGATGAAAGCTTCAAGCCAATCGGTCGGGTAAGCCAAGGGCATGGATGAGTGAACTCGATGCTAGAAGGCTGCCGTAGTTGTAGAGCTTTCAAAGAATTGTTGAAGCTGCCGGGCATCTACGTAGGACCTCTTGCGAAGTCTTCCCAACCATAAAAGCCAAACAGTGCAGCTCCTACTCCTAAAAGGCAAAGGGAATAGAGCGATTGTTCCAATCAGAGAAGCCTGCCCCGACTGTGCATCGAGAAGTTCGTCTAAGCATTCCTTTATGTGGGAACGTCGATCTCTAAATCTTCCATTTCTAGCGTGGATAGATAAGCCCGAAGCTAGTCTTTTCGTTGTGATGGATTGGATTCTGCAAGAAAGGCTATTATTGCCCGCTCCGCTACTAAACTCACTTGGAGAAAGCAACTAGGTCCCCGAAGGGCCAGGACATAAACTCATTTTCCCATTGCATGCGACAACTAGTCGAGAAAAAGGGAAGAAAGCCTAAGCGAGACAAGACAAGAAGGAATGTTGGAGCTATTCCCTTAGTGGCAGTAGCATCTTTCTTTCGAACATTCGGACATTGTAGGCTTTCCTCCAACTATTAGTTGGACTGGGAAAGAAAGACTCACATTCCCGGATAACCAGACAGTCTCGGAAGATATCAACCATGGAAGGTCTCCTGTAAGCCAGAAGGTGCTACTAAGTGGTCTAACTCGAATCTCTTCCTTGAATGGTTAAATGGGACAGCTTTCATCTAATGCCTTTGTTCGGACCGGACTCTTGACTTGTCAACTTCAGCCAATTTCAGGTGCTAGAAAAAAAGAAATAGGAATGGAATTTAGGGACAGATATAGTCTCGCTAAACAGCTAAAGTCAACAGTAGCGGTAGGTTCTCCAACTCGATAAAGAAGCTGTACCGAGAAAGAGGCACTTCTCTATTTAAATATTGAAATTCAAAAGGATAGGCGGAAAGCCAGTCGTTATCCTGGAACCTTAGCCCGAGCATCTTGTTATCATATCTTCGAGACTTGCTGGTCTGCTCTGTATGGAGAACAAGTCCTCTCCCAGCCCAGCTGTACATTTAGGAAGTAGATCGAAAGAAAGGAAATTTAGATTATCCTTCAAACAGTCACCACTCCCTCCTCTGGTAAAGAAAGTGGGAGATCTCAGCGATTGGACTACTCATCTCTCGGGCACACGCCCTAGCAAGAGCAACTCTTCTCACTCTGACTTCGGTAGAACACTGGACACCTACTATATCGCCGATTCCTTAGCTCTAGCTCTCCTCTAGCGAAAAGGTCAGAGGCAGATGTTAGGCTATTGATCCGGTCTCTATTAATAAGGCTTTTCATCACCGGCTTTTCCCACTCAATCCCTCCGAAGTCGTGGAAGAGCAAGTCTTATTAATAAGGAATTTCGCCCATTCCTAGATAGAAAGAAGAAGGGCATCTCAATCTCAACCAGCAAGAGACGCATCAGCAGTTGAAGGCAAAGAAAGAGGAAGAGAAGAAATGGATTTGGATCAGCCGTCTTGATGATGAAAAGGGCCGCTAGGCCGCTGTCCCAATAGGCAACGGAGCGGAACGAGAAGGAATGAATGCAAGCAGGTCAAGTACACCCCAGGGAAGAGAATCCGGTTAACATAAAGTCTAAAAAGAAGAAGATCTTTTGAGAGATCTCATGAAAGCAGGAAATGATCAAATACGAGATTGAAATAGAGGGTGGGATCAAGCAAGCATCGCCATAGAGTGCTAAGGATAGAGTGAATGGGAGAAAGAATGTCTAAAATAGAAGGACTGCTACTAGAAAAAATTAGCCCATTGGACTTCAGTAATCTTCTCTGAAGAAGAAAAAGCCAAATCCCTGGTAGAATGTCCCGGACACGTAACTGGGACAAGAAAGTCAACCTCCCTCGTTAGACGGCATACAGTAGCAAATGAACTAAAGGCCCGGATGCTACTTGATGTCTCCTGGTAACAGCGACCGGTCTGTTACCCCTTGGAAAAAAAGCTTCAAGGAAGTAGGAATGCCAGGCGAAGAAGGTGGCTCCATACGGGATGAACTGGGTTTTGTCTCGCTTAACTCGTCGAGTAGTTATTATCCCGGAATGAAGATCTGCTCGATCAGTATCGCGAAAATCCGTATTCCGTATAATGATTAGCTCTTAGCCCCTGCTTCGATCATTGGTGCTATCATCGTATATATAGAATAGATCACTCCTGCAGGACCTCTTTCTATAAATTGAATAATGGAAGGTAGAAAAGAAAGGGATGGATGGCCTGGCCATGAGAAGGAAGACTATATATGCTTAACACACGCAAGTCGATGATCCGCTTAGCAGAGTGGATTCTCTCCTGAAGGAACTGGCTAACTCAAGACAGTGTAGGAGTAGGAATTTCAATCAACTCAGATGCAACAACAGAAGCTCCAACGTATCCTGCAGCTACGGAAATGGGGCTCCTAGGGTTCCTGAGAAGGGTAAGCTAAGAAGAGGTCGAGGGCAAAAGAAAGAAAAAGCAAGTCAAAACTTATCCGGGATTCCCTGGATTGGTCTATTGTATAGTTGATCACGGCTGCATGACGGAAGGATCTTTCTATCTAACTATCAATTTCATAAGAGAAGAAAGGTCCACAGAAGGAACGTAGTGCTCGACCGGAACGGAAGGGAGAGGATGGAAAGTGCCCCGTTCACTTACTAGGATCCGCGTTTCCTAGCGAAATCCAGGCGGTTCAACAAATGAAGGTAACCGCTCCGTCTAAGGCGGAGCCGGGAAAATTGGAAAACCATGAAGTCTATGAAAGGTCCTAGGGAATTTTTTGTGTTTCAGAGCTAATATCATGAATATCCTTCGCCCGTTATCTACTTATTTGATTTTATTGGAAACGATTGTCGATTCCTGTCTTATTTTGTTTCATTAGACTTTTGTTTTTTATCTTCTATGTGGAAATTGGTTCGATAGAATTGTTTCAAACCTTACTGAAAAAATCCAATCAATGATTCGGAAAGGGAATCCATCTCCTAGTAAGGTAAGTAGGAGTGGTTGGGGCCTCCTTTGCTCTGATTCTGACTTTTTTGTAGCGACACCTGCACTAATGCAATGGCTCCAGATACAAACGACAGGGTCTCAGAAGCGACTGTAAACCAAGCGTCGCATAGGAATGATGCGGGTCCTTGACAGCGGGGCCTGCTAATATAGCACCCATCCCTGGGGAAAAGCTCGGCGAGGGCTTTATCGGAAGCGGGCTTTGGACAGAGCAGACGGGAACCTCAAATAGCACTTAGTAAACTAATCGTTACTAAAAAGTCTGAGCTTGGTCGGTTAATCAATAGTTAGTCTTCGAAGGCATAAATATTTTATCGCCATTGGCGGGTATTGCACACCGCCTATCTCTATATGGGCTTGGTGGGGCACAGGACAGCAAAAACAGCCCAGCCGACTTAATGATGGCCGCGCATGAGATCGCACGAGACCACTTCGATTATGAGTCGGGGGATGCGAACATAGTGGCCCTAAAATACCTTATGCGCTATCTTAAAGGCGAAAAACTTCCATTTTGAGACCGCGACCCATCGCGGAGCACTTTCTCGCTAATTTTTGAGATAAGTGTGAAATTATCCTCCACAAGACTCTTTATGTTTTTGGGTACGAGAACATAATTGGAAAAGGATCGAAGTTTAGACCGCTCACAGTAGTTCTACCTATAGAAAGATCATCAGAGGATAAAACCCATTTATGATTCCAGTCGAGAAGACTAGTAAAAAGAAGGATCTATAATGTTATATATTTCAGGAGCTAGATCAGTTGCCGATGAACAAGTAAGAATTGCCTCAACCAAAATTGATGGAATTGGACCTAAAAAGCCATTCTGGTTCGTTATCGATTAGGTATCAGTGGGAACATAAAGATAAACGAATTAACTAAGTATCAGATCGACCAAATTGAACAAATGATAGGTCAAGATCATGTTGTTCATTGGGAATTGAAGAGGGGAGAACGAGCAGACATCGAACGATTAATTTCTATTTCTTGTTATCGTGGAATTCGTCATCAAGACGGATTGCCCGTACGCGGTCAACGAACTCATACTAATGCTAGGACTTGTCGCAAGCTAATTCGGAAATGAAAGAAGAAGTCTACCGAAAGCCCTTGGTACTTGTCTGATCAATTACACTGATAGCTTCAATAGTTCACTTTCATTTTTTAGTTTTTTGGTATTTCACTGGTGACTAATCCAGTATAGTAGGCCTCCTTCGCCACGACATTAGTGGCTTAGCCCTTCGCCCGCCTATCGTATCGGCTCGGCTTCATCCTAGAAGCGACTGCTTCTGCCTCTCTAGGCTTCGCTATCGCTCATGACTGGTATATGGATCTCTCTATGTAGTGGTCGGCCTTCTAGAAGCTTCGCCAGAAGCGACTAGTCGCTTCCCGAATGCCTCCTTACTTTAGTATAGTCTAGTTCGTTTTGCCTCCTTCCTTGCCGCCAACGTAGGCTACTAGGAGAGTAAGCAAGCTAGATTCTAGAAAGGGTAGCTTCCGCGCCCTTCCTGCCTGCTGAAATTTATGTGAATGATCGATCGTGACAGCTCTTCAAATCCTATTCAGTTTTCTTTTGTCACTGAAAGAAAATGATTCCGTTCTGTCTCTGTTTTATTTTCGGATTCCGGGATGAGCCGGCCGATCCTAACATCATTTATGAGGAGCCGGACGACGAAGCCTCCTCCTCAGATAAAGATGTCTCCGACGCCGGCAAGAATAACTTTTCTATTGTGATTTTTTGGCGAGTTCGGTCAGGAGGGACAGAGATGCTTTCTATCAGTCAAAAGCAGACACCGCCCCCATGCTCCCACGGTCCGCTTACCGAGGAATAGAAAGGAAGGACCAGGGCCAGAGCAAGTTGGGTTGGGGTATAGAGCCGTAAGCGCGGTGGGGGTGACAGAGGACGTGCTCGTACGGTTCATAGAAGGATATGATCCACTACTTGATTGTTGGGAACTTTCAGTCCTCTATATTCGAAATATGCCTTTCTAGGAGCATTACGATCTGCAGCTCAAATGGTCTCTTATGAAGTCTCTATTGGTCTTATTCTTATTGTGCGCCTTGTGAGCGCGTTTGGATCTGCGAAGGCAATCGCTCGGATGTTCCCTAACCCAACCCGGGAACGGACCGGAGGGAACCGCAGCATGGGGAATGTCCGCGTCTCGTCGCAAGGCTCATTTTGAGTTGTGGGTCATAGGGCGGGCAGCTTTATCTGATCAAGGGCCGGGGCACAAGGGTCCTGGTACTATCCAGGTGCGAAGAACCCCGGAGGTGACTGCAATAAGCAGAAATCTCACTCACGGGCCTAAACGACGAGCAAACACTCGAACGTGAGAGCAAGGGATCACCCAACGAATGGACGAGCTCAAAGGGGCGGGGGCAAGAACCATGTTTTCAGAGAAGTGGCGGTCCAAATCTTATCTGAACTGCGAGAATAACTGACTAAGCCGTGCCATAAGGGTCATTCCCCAAACGGGACGGGGAAAATCCTTAGGTTGTTTAAGTAGGTTGGGGACAGATCGGCCATAGGAGTACTCCGGGATATAAACCAGGGCAACAAAAGTGGAACATACGACGATGCCGCCCGTTTTCATTTCGTGGAAGTCCCCGGCAGAGGAAAGGGCTGTAGGTGATAGCGCGTTCTGCTTCTTATCTAGAGAGGGGCGCTGAAATCCTTTCTATTGGTTTGGTTCGTGCGTGGCAGCTGGTATAGATGAAGAAAGGCGGGCCGCCGGGACCTATTCTCTTAATAGTCGTCAAAGCGAAATAGGAAAGCCGAGCTAACTGATGAGTGTCTTTTAGCCTTTTCTAAAGGCTCGTGTGGAGCTAACATGGCTGTCTACATACAAGTATAGCCAAATCAAGATGAGACGGGACGGACGGTCAGAGGCCGCAGCGGGACTACCATAGGAAAGCCCGCCCCCGCTAGCTAACATAGAAGGATATTCCGGGATAGCAGCAGTCTCTATGTGAATCTCTTCCCGACCGGGCCAGGCCGAATCGGGCCACCGCTGGGATGGGAATGGCTCAGCCCACATGCATCATTTGATGAAAGCACTCCAGTCCAGTCGCCCTCCAATGATTGGTTTGTCAACCACGCTTTCCCTCCCTCAAAACAATGCTCCTCACCAACTTGGGTTGGGGCAACACAGCAATGAGTAGTTCGCTCCAGGACCCCACCCCTCGAGAGCAGGATGCCGGCCGAGATGGAGCTGGGAGCAAACCTAGACTTTCCTGGGGCTTGCCTTGCCTCAACATCTAAATAAATAAAGGGCGGGCGCCGAAAAGAATTTCAGCCTCTTCAAAGCTTTGCTTGGTAGGCGCTGCCTACTCACTCGGACAATGCTCTGAACACGAAAGTGCAGTTCCGCCCCTTCTCCCATGCTGAGTCACAGGCAGCGCCTCGGAAAGCAGGACGAGCCACATGCAGGGAAACTTGCACGTGTGGTTCTGGCC

>Decalepis_hamiltonii_chloroplast_contig10

TTATTTCAGATTGATCGAAACAAATACAAATAGGTGTAGCAAATAAATAGAATTGGGCGCTATGTCAATTCCATATATGGAATTCATATCCACATACATATATAATATTGTATATTAATCTATATTAAGATTAATCTATATTAAGTTAAGCCTCTACCTTTATTTTATTCTTTATACACTACTATAATACCAATAGATCATATGGTCGAAAGATTCATCTATTTCTTTCTACCATACGATCTATCATTAGAATACTGCGGATTATAAGCCCGCTTATTTCATTTAAGACAAAAGTCCTTTTCATTTTATTTCGTCAATTTTTGATAAGAACTCAGAAGTCAAGTTTAATTCAAATTAATGATTAATTAATTAATTATTTTGACTGATTGTTTTTACGTAAATGATAAGTAGAAAGGCGGTAGGAACTAGAATGAATAGTGCAGTAGCAATAAATGCAAGAATATTTACTTCCATAATCTAATCTTTTTTACTTCGCAATAACTCGGGATTTAGTCCCATAGAGATGATAAACCTTTCATCTGTAAATTCAATGAATGAATTCCCTCTCAATCTCGCTGGTTTTGAATCGGATGAATATCATGAATAACAATATCTGAGCTGTCAAATCAATTTCTCGTCGAAAATGGAATAGTATAACATAGGAAGTTCTTTTATCCATACCGAATCCCAGCTTGGATTCCGGACCCAATCCAAAATTCCTTTATTTATCATTTATCCTCTGTTTCCGTTCTTTTTTCTATAATCTACTTTTATTGTGAATTCCATTTGTGTATGTGTGCCCTCTCCAAAAAACACATAGTATTCGATTCCATGGATCGGGAACAATCGAAAAGCGGATCCAGGATTTCTTGGAATGCTTACTATAATGCTACCGATAATTGTATTAATCCGCCCACATATGTTTTCTCCTACCACAAAGAAAAGAAAAAGACTTTATTTTGGGAATGAAATTCTGCCCTGGCCCCCTTTCGAATTGATTGATGTATTTAGTGGATCCGTCGGGACTGACGGGGCTCGAACCCGCAGCTTCCGCCTTGACAGGGCGGTGCTCTGACCAATTGAACTACAATCCCAGGGAAAGGAAATAGGGATCTAGCAGAAATTTTGATTCTTTATCTCCGTATCGAGTATTTTTCAAGGGCGCGGGATTCTACCGGGGATTATACGTGGTAGATTGGCGAATTTGTGGGCCGAGCTGGATTTGAACCAGCGTAGACATATTGTCAACGAATTTACAGTCCGTCCCCATTAACCGCTCGGGCATCGACCCAGACCCAGGTGTAAGGTTATTAGCAATTCGCGATCAACTTCCTTTCGGAGTACCCTACCCCAGGGAAGTCGAATCCCCGTTGCCTCCTTGAAAGAGAGATGTCCTCACCGCTAGACGATGGGGGCCCACTTGCTTCAAAGTCATGATAGTCATCATACCGTGATGATAGTATCAACAGTTTTTGGAAAATGTCAATATAATAGAATGGTATGATTAGATCCGAGGAATCTTTCCTCTTTTCTAATTGCCGATAACTTGTTGATTCGTCATTCATATTCATGAATCATTCATTGGAATGGGCATTCTCTACTCTATATCTCTATATATATCAATTATATATATATCAATATATAAAATAGAAATGGATTCTATATCTATATTTATTTCGTCTACTATAAACGTCTAATATAAAAAGTGTAAATAATACAAAGTAACAAAGTATAGGGTTTTCGGGGAGTCGCTGGTCCAAAACAAAAAGGGGTTAAGTTCCACTTCTTTCGCTTTCATTCATTGATTCATTTATTCTTAAGATGAGATAAGAATATCTCACAATAAGAAATTAACAAACGGTAAGCGTGATGAAGAAATTATCGAAAATTGTTTGAATTTAGTTCAGGGGACAAGGCAGGTAGAATTTCTTCATCACAGGATTTGATGAAATACCTTGAAATTTATGTCGAATTGGTAGGTGTACGTGTTGATTCTGATGGGAATCAATTCATTCAATGAAAGAAAAAGAATTTGGTTCGGTCTTGAAACAATTCATTCCATTTTACCCTAGACTTGCTGGTTTTATTATTCAATAATAAGCCACTAGCAAGTATGAGTCTACCGTACTGTATGTATTATATACATATGTATATATACATAATAGAAATAATATAATAATATATATAATAGAATATATATAATATTCTATGTACATATAGATTTATAGATTCTATAATTTCTATAATTCAATAATAGATATTGTATCCACATAGTAACCATTCAGGAATTCAATCAAATAAGCCCTTTTAACTCAGCGGTAGAGTAACGCCATGGTAAGGCGTAAGTCATCAGTTCAAATCCGATAAGGGGCTTCGGTTTTTCATAAAACTCCGGTCGGAAGAATAGAGATATTTTTAATACTTGGAATAAAAACGGAGAATACATTATTATAAGTTATAAGTATAGTAGTAAAAGTTCAATAAATTGGAATTATTATGAATAATGATAAGTTACCTCTTGAATGACGAAACCACATATTCCTATTTTCCATTCTACCAATAAAATCCATTGGAAAGAATAGAAATCAACAAAAGAAAAAGTAAGTGGACCTGACCCATTGAATCATGACTATATCCGCTATTCTGATATTCAAATTCGATAGAGATGAAATTGAAACGGTTGATTTTTTTATTTCATTTTTTGACTCCGCAAGAATTTGTCGATATTTCCGATTAAATCTTCTTGTTCCTAGATTTTCGATATATAGGAATAAATTGTTATTCCGTTACTCCCTATAGAAGGGTTTATTACAAGTCACAACATAAGAGCCATTTCCACTATTTCTCTTTGATTACGGATCAAGATTCCTTTCTATGTATATAAGTATATTTAGACGTATATCTACCAGATCGTTACTTCATGTACCAAATATTTTGCATTCTATATTTTTGTTGTGACAGTGCGATAGAGAATGGATGCGAAAAGAGACTTTCATTTCCAGTCTCCTATTTCTTTTTATTTTTAATTTAAGAAAATAGGAAATTCTCTCTTTTCGCAGGACTGGATGAAAGGGAGATAGAACTAATAAAGAAGATACTAAAGAAAAAAAAAAAAAAAGTAGTAAAATACTGTAATTTAAATTTAGTATACATAGAAATTAGAAGACTCTATAAATATAAATATCTTTATTTTTCTCAATCTCCCGAAAAGATCTAATAATAAAGATTAATTAATCGAACAAAACAGGGGGTTAGGTCTGAAGATCGATTGGGAGTGAGAGAGGGGTTGATTGTTCCTTGAACGGGTCTTTCAAAAGATATTCATTTATCTGATTGATAGATGAGTCATAAGAAAACAATTCGCGGTTCAGAGACTTAGTAAGAAGGAATAATCAATTGAGTTCATGGATTTATCTAGGCGAATTTATGGACCAATACCACTAAGGGATTTTTATCTTCGAAACCCATTGGAGTGGGGCAATGCAAGAGAAATCATACAGAAATGATCGAATCTTCGAACGCCCCGAAAATGCTATGAGGTGCTCGGAAATGGTCGAAGTAGTTGAATAGGAGGATCGCTATGACTATAGCCCTTGGTAAATATACCAAAGACGAAAATGATTTATTTGATATTATGGATGACTGGTTACGTAGGGACCGTTTCGTTTTTGTAGGCTGGTCCGGTCTATTGCTCTTTCCTTGTGCCTATTTCGCTTTAGGGGTTGGTTTACAGGTACAACCTTTGTAACTTCATGGTATACCCATGGATTGGCCAGTTCCTATTTGGAAGGCTGCAATTTCTTAACTGCCGCAGTTTCTACTCCTGCTAATAGTTTAGCGCACTCTTTGTTATTACTATGGGGTCCCGAAGCACAAGGAGATTTTACTCGTTGGTGTCAATTAGGCGGTCTGTGGACTTTTGTTGCTCTTCATGGCGCTTTCGCATTAATAGGTTTCATGTTACGTCAATTCGAGCTTGCTCGATCTGTTCAATTGCGCCCTTATAATGCAATCGCATTCTCTGGTCCAATTGCTGTTTTTGTTTCTGTATTCTTGATTTATCCGCTAGGTCAATCTGGTTGGTTCTTTGCGCCTAGTTTTGGCGTAGCAGCTATATTTCGATTCATCCTTTTTTCAAGGGTTTCATAATTGGACATTGAACCCCTTTCATATGATGGGAGTTGCCGGCGTATTGGGCGCTGCTTTGCTATGCGCTATTCATGGTGCTACCGTAGAAAATACTTTATTTGAAGACGGCGACGGTGCAAATACATTCCGTGCTTTTAACCCAACTCAGGCTGAAGAAACTTATTCAATGGTCACCGCTAACCGCTTTTGGTCACAAATCTTTGGGGTTGCTTTTTCCAATAAACGTTGGTTACATTTCTTTATGTTATTTGTACCAGTAACCGGTTTATGGATGAGTGCTCTTGGAGTAGTTGGCCTGGCCCTGAACCTACGTGCCTATGACTTCGTTTCTCAGGAAATTCGAGCAGCGGAAGATCCGGAATTTGAGACTTTCTACACCAAAATATTCTCTTAAACGAAGGTATTCGTGCTTGGATGGCGGCTCAAGATCAGCCTCATGAAAACCTTATATTCCCTGAGGAGGTTCTACCACGTGGAAACGCTCTTTAATGGAACTTTAGCTTTAGCTGGTCGTGACCAAGAAACCACCGGTTTCGCTTGGTGGGCCGGAAATGCCCGACTTATCAATTTATCCGGTAAACTGCTAGGAGCTCATGTAGCCCACGCTGGATTAATCGTATTCTGGGCCGGAGCAATGAACCTATTTGAAGTGGCCCATTTCGTACCGGAGAAGCCTATGTATGAACAAGGGTTGATTTTACTTCCCCACCTAGCTACTCTAGGTTGGGGGTAGGTCCTGGGGGAAGTTATAGACACTTTTCCGTACTTTGTATCTGGAGTACTTCATTTAATTTCCTCTGCGGTTTTGGGCTTTGGCGGTATTTATCATGCGCTTCTTGGCCCTGAGACGCTTGAAGAATCTTTTCCATTCTTCGGTTATGTATGGAAAGATAGAAATAAAATGACCACAATTTTAGGTATTCACTTAATCTTGTTAGGTCTAGGTGCTTTTCTTCTAGTATTCAAGGCTCTCTATTTTGGGGGCGTGTATGATACTTGGGCCCCGGGAGGATGTAAGAAAAATTACCAACTTGACTCTTAGCCCGAGTATCATATTTGGTTATTTACTAAAATCTCCCTTTGGAGGAAGGGTGGATTGTTAGTGTGGACGATTTAGAAGATATAATCGGAGGACATGTATGGTTAGGTTCCATTTGTATACTTGGCGGAATCTGGCATATCTTAACTAAACCCTTCGCGTGGGCCCGACGCGCACTTGTATGGTCTGGAGAGGCTTACTTATCTTATAGTTTAGGGCTTTATCCGTCTTTGGTTTCATTGCGTGTTGTTTTGTCTGGTTCAATAATACCGCTTATCCGAGTGAGTTTTACGGACCCACTGGACCAGAAGCTTCTCAAGCTCAAGCATTTACTTTTCTAGTTAGAGACCAACGCCTTGGCGCTAACGTGGGATCTGCGCAAGGGCCTACTGGTTTAGGTAAATATCTAATGCGTTCCCCAACCGGAGAAGTCATTTTGGAGGAGAAACGATGCGTTTTTGGGATCTGCGTGCTCCTTGGCTAGAACCTCTAAGGGGCCCAAATGGGTTGGACTTGAGTAGGTTGAAAAAGACATACAACCTTGGCAAGAACGACGTTCCGCAGAATATATGACTCATGCTCCTTTAGGTTCTTTAAATTCCGTGGGTGGCGTAGCTACCGAGATCAATGCAGTCAATTATGTCTCTCCTAGAAGTTGGTTAGCTACCTCTCATTTTGTTCTAGGATTCTTCTTCTTCGTAGGTCATTTGTGGCACGCGGAAGGGCTCGTGCAGCTGCAGCAGGATTTGAAAAAGGAATTGATCGTGATTTTGAACCTGTTCTTTCCATGACCCCTCTTAATTGAGATAAGACAGGAGATCCGATGCTTGAAATAGGAATAATCCCTTTCATTCCATCATACATCTTGGGATCGGGTCATTCTTAAAATATTTTGAAATCTTTTTTTCAACTCATTTATATAATCATAATCTATTTTTGGCCTGGCTCGGTGGGATAGCCGAGCCATTCCTCCTTCTTTACGATACCCGTTCGGAAAAACTAATAAAAAACAAACCTATTCAATGAGCAAAAAGGAGAGAGATTCGAACCCTCGATAGTTTTTGTTTAAAACTATACCGGTTTTCAAGACCGGGGCTATCAACCACTCAGCCATCTCTCCGAAAGACCATTTTATTTTATTCCTCCGAATAGAACATGACCATAGTGGTGGTATCCACCACTATCTGTAGAAAGACCCCGGGTGAATCTACCGATGGATTTATCTATCCGTATATATGAGCATGCCCATTTGTGAAATAAAAAATTCATTTACCCTGACCCCATGTATGATAAAAGGGTTAGTAATAGGTCATATAGAATCAATAGATTCATGGTAAAGTAAAATCCCCTGCTGTATTTTATTACAATTTTTGGCTAATAGAGGGATCAAATGGTAGAAATTCATTTGTTGGTAGCTTGGAGGATTAAAAGCATGACTCTTGCTTTCCAATTGGCTGTTTTGCATTAATTGCTACTTCATCAATCTTATTGATTAGCGTGCCCGTTGTATTTGCTTCTCCTGATGGTTGGTCGAGTAACAAAAATGTTATATTTTCCGGTACCTCATTATGGATTGGGTTAGTCTTTTAGTCGGTATTCTTAATTCTCTCATCTCTTGAACCTATCGTCCCAGATCCAAAACCGAAAAGACCCCCGAATTTTTCTCGGTTGTAAGACACATTAGAATTTAATATAAGTCCCCAAAGAAAATAGAAAATGCAAATAAAGAAAACAAAAAATTAGAGGGGGTCAAACTTATTCCAAACTTATTGAAAAAATACAATTAATAATTTACAATTAATTATTAATAAAATAATTGGAATCGAGCTGAGGAGAGTCTTTGGTCTGGGACTGCACAAAATGATCGAGGTATATATTAATATATAAATATTGTGTGGACATATTATGTATCAAGAACGAAAAAATGCGGATATGGTCGAATGGTAAAATTTCTCTTTGCCAAGGAGAAGATGCGGGTTCGATTCCCGCTATCCGCCCAAGATAAAATAATTTATTAAATATGATTTGGTATAGTTGTCCGCGATCGGATAGTGATTCTATCCTCCCCTTTTCCCTTTCCTATCGAAAGGGTAATTAATTACTAGTCGCCTCCAATTACTAGTTAACTTAACAAAGTGAAATCTACAATTTTTGACAAACAAGATGTTGCGGAGACAGGATTTGAACCCGTGACCTCAAGGTTATGAGCCTTGCGAGCTACCAAACTGCTCTACCCCGCGATGAAGAGAATAACTGAAAACTAATAGATAACCAAGGGTTGAGTTCGCCCCTCTACCATATCTGTACAAATAGGGTAGCCCATTTATACAGAATGGTAAAGGGTTATACAGAATGGCAAAGGGAACCTCTACGATCACCGACCCTAGAATAGAAAAATAGAAATCAAGAGTGAATCCTTACCAACTCGATCTTGTTGCTCCTGGCAACAAACATGCATGAACCATTTCACGAAGTATATGTCCGGATAGCCCAAAGTCTCGATAATTAGCTCTCGGCCGTCCGGTCGCAAAACAACGTCGATGAAGGCGTGTAGCTGCGCTATTCCGCGGTGGGGATTGTAACTTTCTATAAATTTCCCATTTCTCACTTAATGACGGAACTTTGCTTATTTCTTTTTTGAGGATTGACGAATCAAATGATATTTCTGTTCTAATTTTTGCCTCTTCTTCTCCCGCTGAATCAAACCTTTCCTTGCCATAATGGTTCAGTTCCTATTAGTATCCATGATACAAGTCGAATCCTAGATGTAGAAATATAAGAAGGTGGTCCCTGTCTATCGAAAGAAATGATCTTTTCGCGGATACAAGACATTCAAAAAGAATTCAAAATTTAACCAAATTTGCCCGATGTAGAGGCAATCAAGAAAGCCGCATAAGTGAATATATAACCTACGGAAAAGTGGGCTAATCCAACTAATCTTGCTTGCACAATGGAAAGGGCCACTGGTTTATCTCTCCAGCGAATCAAATTGGCTAAAGGTGTGCGTTCATGAGCCCAAGCTAAAGTTTCAATCAATTCCTGCCAATAACCACGCCAGGAAATTAAGAACATAAATCCAGTAGCCCAAACAAGATGTCCAAATAAGAACATCCACGCCCAGACCGATAAACTATTCATACCAAAAGGATTATATCCATTGATAAGTTGTGAAGAGTTTAACCATAAATAATCTCTTAACCAGCCCATCAAATAGGTGGAAGATTCATTAAATTGTGAAACGTTACCCTGCCACAATGTGATGTGCTTCCAATGCCAATAAAAAGTAACCCATCCAATAGTATTTAACATCCAAAAACTGCCAAATAAAATGCGTCCCATGCCGAAATATCACAAGTACCACCCCGTCCCGGGCCGTCGCACGGAAAACTATAACCGAAATCTTTTTATCTGGCATTAACTTGGATCCACGTGCATCTAAAGCACCTTTTACTAGGATCAATGTAGTTGTATGTAAACCCAGAGCAATAGCATGATGAACCAAAAGTCTCCAGGACCTATTGTTAAGAATAATGAATTGCTATTTTCATTAACAGCATTTAACCAACCGGGCAACCAAATGCTACGACCCGCATTGAATGCCGGCCCGCTTGTTGAAGATAAAAGTACATCGAACCCATATGAAGTTTTCCCATGAGCCGATTGTATCCATTGGGCAAATATAGGTTCGATCAAGATTTGTTTCTCCGGAGTACCAAAGGCAAGCATGACATCATTATGAACATAAAGTCCCAAAGTATGGAATCCCAGAAAGAGGCTGGCCCAGCTTAAATGAGATATGATAGCTTCTTTATGGTCTAGCATTCTTGCCAATACATTATCTTGATTCTGCTCCGGATTGTAATCTCTAATGAAAATATAGCTCCATGAGCAAAAGCTCCTGTCATGATAAATCCTGCAATATATTGGTGATGAGTATATAATGCAGCTTGGGTAGTAAAGTCTTGTGCTATGAATGCATAAGCAGGTAAAGAATACATGTGTTGAGCTACCAAGGAAGTAATAACCCCTAAAGAAGCTAGAGCAAGGCCTAATTGAAAATGAAGCGAATTATTGATTGTGTCATAAAGACCCTTATGCCCGCGTCCCAACCGTCCCCCGGAGGAACATGTGCATCTAAAAGATCTTTCATACTGTGCCCAATCCCGAAATTTGTTCTATACATATGACCAGCAATGAGAAAAATAAATGCAATAGCTAAATGATGATGAGCAATATCAGTCAGCCATAAACTTTGCGTTTGTGGATGAAATCCTCCGAGAAGAGTTAGAATGGCAGTTCCGGCTCCTTGGGAGGTCCCAAATAAATGACTACTTGAATCGGGGTTTTGAGCATAAAGATTCCATTGACCTGTAAAAGTGGCCCTAACCCTTGGGGGTGTGGTAATACATCTAAGAAATTATTCCATCGAACATACTCCCTCTGGATCCAGGAATAGCGACATGTACTAAATGCCCTGTCCAAGCCAAGGAACTTACGCCGAACAGTCCCGACAAATGATGATTCAGCCGAGATTCGGCATTTTTGAACCACGAAACGCTTGGTTTCCATTTCGGTTGTAGGTGTAACCAACCTGCTATTAAGAATAGGGCAGAAAGAAATAATAGAAAAAGAGCTCCAGTATAAAGATCTTCATTAGTGCGTAAACCGATTGTATACCACCACTGATAAACACCAGAATAAGCAATATTCACTGGGCCAGGAGCACCGCCTCGAGTAAAAGCTTCCACGGCCGGTTGACCAAAATGAGGATCCCAAATTGCATGAGCAATAGGTCTTACATGTAAAGGGTCCTGTACCCATGACTCAAAATTTCCTTGCCAAGCTACATGAAACAGATTTCCGGAAGTCCACAGAAAAATTATTGCTAATTGACCGAAGTGAGAAGCAAAAATATTCTGATAAAGACGTTCCTCAGTAATATCATCATGACTCTCGAAGTCATGTGCGGTAGCAATACCAAACCAAATACGACGAGTAGTGGGGTCCTGAGCTAAGCCTTGGCTAAACCTTGGAAATCTTAATGCCATAATGCCTTTCAAATCCTCCTAGCCATTATCCTACTGCAATAATTCTTGCTAAGAAGAATGCCCATGTTGTGGCAATTCCACCCAGAAGGTAGTGGGTTACTCCTACAGCACGTCCTTGTACAATGCTCAAGGCTCTCGGCTGAGTAGCAGGAGCAACTTTTAATTTATTATGAGCCCAAACGATGGATTCAATAAGTTCTTGCCAATAACCACGTCCACTGAATAGAAACATTAAACTAAAAGCCCATACAAAATGAGCACCTAGGAAAAAAGGCCATAGGCAGATAATGACGAACCATAAGACTGAATTACCTGGGATGCCTGCGCCCATAAGAAATCGCGTAGCCACCCATTAATAGTGATAGAACTCTGCGCAAAGTTTCCTCCCGTGATATGACTTACTACCCCTTGATCACTTATACTTCCCCAAACATCTGACTGCATTTTCCAACTGAAATGGAATATTACTACCGAAATTGCATTGTACATCCAGAATAGTCCTAAGAAGACATGATCCCAAGCCGATACTTGGCATGTACCCCTCTTCCAGGCCCATCACAAGGAAAACGAAAACCAAGATTTGCTTTATCCGGTATTAAACGTGAGCTACGAGCAAATAAAACACCTTTAAGGAGTATCAATACCGTCACATGAATCGTAAATGCATGAATATGATGGACCAAAAATCTGCGGTTCCTAATGGAATAGGTAACAAAGCAACTTTGCCACCCACTGCTACTAAATCCCCACCCCCAAGTTAAACTGGTGCTTGCTGTTGCACCAGGGGCCGTCGCGCCAGGTGCAAAAGCGTGGGTATTTTGTATCCATTGAGCAAAAACAGGTTGTAATTGGATAGCGGTATCTGAAAACATATCTTGAGGGCGCCCCAAAGCGCTCATGGTATCATTATGAATATACAAGCCAAAACTGTGAAAGCCTAGAAATATACATACCCAGTTGAGATGTGATATGATTGCATCACGATGTCGAAGGACACGATCTAATAGATCGTTGTATCGAGTAGTTGGATCATAGTCTCTTACCATAAAAATGGCTGCGTGCGCAGCCCCAACTATGAGAAATCCACCAATCCACATGTGATGTGTGAACAACGACAGTTGTGTACCATAGTCAGTAGCTAGATATGGATAGGGGGCATGGAATACATATGGTGAGCTACAACAATGGTTAAAGAGCCTAACATAGCTAGGTTAAGAGATAATTGAGCATGCCATGATGTTGTTAGGATCTCATATAGCCCTTTATGACCCTGACCAGTAAATGGGCCTTTGTGAGCTTCTAAAATATCTTTCAGACCATGACCAATGCCCCAGTTGGTCCTATACATATGACCTGCTATTAGGAAAAGAATTGCAATAGCTAAATGATGGTGTGCAATATCGGTTAACCATAGACCCCAGTTACTGGATCTAATCCTCCACGAAAAGTAAGAAATTCCGCATATTTTGACCAATTCAAGGTGAAAATGGGGTTGCTCCTCGGCAAAACTGGGATAAAGTTGAGCCAAAAGATCTCGATTCAAGATAAATTCATGAGGAAGTGGTATCTCTTTAGGATCTACTCCCGCGTTTAGAAATTGATTAATTGGTAAAGATACATGTACTTGATGTCCTGCCCAAGAGAGACCCAAGTCCTAGTAACCCCGCTAAATGGTGATTCAACATAGATTCTACGTCTTGAAACCAAGCCAATTTTGGAGCTGCTTTATGATAGTGAAACCAACCAGCAAAAAGCATTAACGCAGCAAAGACCAACGCACCAATTGCGGTACAATATAGTTGTAATTCACTAGTTATTCCAGATGCTCGCCAAATCTGAAAAACCCGGAGGTTATTTGTATTCCTCGGAAACCTCCGCCCACATCACCATTCAATATTTCTTGGCCCACTATTGGCCAAACCACCTGGGCACTAGGCCCAATGTGAGTTGGATCACTAAGCCATGCTTCATAATTGGAAAAACGAGCACCGTGGAAATACATGCCGCTTAGCCAAAGAAAGATGATGGAGAGTTGGCCGAAATGGGCACTAAATACTTTTCGAGAGATCTCCTCCAAATCACTGGTATGGCTATCGAAATCGTGAGCATCAGCATGTAGGTTCCAGATCCAAGTGGTAGTATCCGGCCCTTTAGCTATTGTTCTTGAGAAATGACCCGGTTTGGCCCATTCCTCGAAAGAAGTTTTTACAGGATCCCTATCTACCAAAATTTTTACTTCTGGTTCCGGCGAACGAATAATCATTGAGTCCTCCTCTTTCCGGACAACACATACAAAGAAACCCGCCAACAGTCAAATAATTAGTGAACCTTTGAGAGATATTTCTATAATTCGTTTATTTCTTTTCTATCTCCCATCTATCTATTTTCTTTAGTTATTCACTAGAGCAATTATGATCTGGAAGTTGATCCTGGGCAAGTGTTCGGATCTATTATGACATAGACATGAGGCGCCCAACGGACCTTTTCATCTTCTAAAACCCTTTTTAGACTTTGGATTGATGCAAAACGACTTTTTCTTTTGTGTAACCTAGTGTATATTCATATCTCAATTAAAAGCCCTTAGATGAAGCTTCTTCATCTTTTACATAGATGATATAAATTACTTACTCTATCCAAATCACAAATCGGGCGAGCAGCCATTACTAAGAGACATCCCAGTATATATATTTAGCCATTCGAAAGTTCCGTTTATTTGATTCTTTTTTATTTTCATTATTTTTTTTTTAATAATAAAAGAAATGGTAGATTATGTACTCTATCTATGTATCCCTTTTATCCCTACGAAATACCAGATTAAATAGAGTACTTAGAAGGGATATAATGAAATTCTTTGGTTGGTTCTTCACAAAAGAATGATCGATTTTTTATTTGACTGATGGGGTCAACAAATAAGTCATTTAATATTAATAAAAATAATATAAAATTATCAAATTATATACATAGCTAAAAATCTAAATAAAAATGAAATAATAAACTTTTATTCGAAACGCCTCGTGATCTTCAACCAATTATGCGCTTCAATACCAGGAGTAAGCGCTATAGCTTGTTTCCAATACTCGGCAGCTTGATCGAACCAAGCTTCCGCTATTTCTGAATCTCCTGTCGAATGGCCTGTTCTCCGCGGTCGGAATAGGTAGGTCAATCCCTTTCCTTAGAACCGTACTTGAGAGTTTCCTACCTCATACGGCTCAGCAATTAATTTCCTTGGTGCCGCATTTTAATCGATCCTATCTAACGAATGAGACTTCTATGGATCTATCCAATTTTTCGGGTTATCCAAAAGAGTTTCATTATTAATTACATGAGTTTCAAACTTGAATTTGGATTCCTAATCCGTTTTATTTAGTTTTACCGTCAGAAGAATAACGGATAGACATTTCCTATCGTTACAATTTTCTG

>Decalepis_hamiltonii_chloroplast_contig11

GACTTTCATTTCAGTCTCCTATTTCTTTTTTATTTTGAATTTCAGAAAATAGGAAATTCTCTTTTTCGCAGGACTGGATGAAAGGGAGATAGAACTAATAAAGAAGATACTAAAGAAAAAAGAAAAAGTAGTAAAATACTGTAATTTCCATTTAGTATACATAGAAATTAGAAGACTATAAATATCTTTATTTTTCTCAATCTCCCGAAAAGATCTAATAATAAAGATTAATTAATCGAACAAAACAGGGGGTTAGGTCTGAAGATCGATTGGGAGTGAGAGAGGGGTTGATTGTTCCTTGAACGGGTCTTTCAAAAGATATTCATTTATCTGATTGATAGATGAGTCATAAGAAAACAATTCGCGGTTCAGAGACTTAGTAAGAGGAATAATCAATTGAGTTCATGGATTTATCTAGGCGAATTTATGGACCAATACCACTAAGGGATTTTGATCTTCGAAACCCATTGAGTGGGGCAATGCAAGAGAAATCATACAGAAATGATCGAATCTTCGAACGCCCCGAAAATGCTATGAGGTGCTCGGAAATGGTCGAAGTAGTTGAATAGGAGGATCGCTATGACTATAGCCCTTGGTAAATATACCAAAGACGAAAATGATTTATTTGATATTATGGATGACTGGTTACGTAGGGACCCTTTCGTTTTAGGCTGGTCCGGTCTATTGCTCTTTCCTTGTGCCTATTTCGCTTTAGGGGTTGGTTTACAGGTACAACCTTTGTAACTTCATGGTATACCCATGGATTGGCCAGTTCCTATTTGGAAGGCTGCAATTTCTTAGCCGCAGTTTCTACTCCTGCTAATAGTTTAGCGCACTCTTTGTTATTACTATGGGGTCCTGAAGCACAAGGAGATTTTACTCGTTGGTGTCAATTTGGCGGTCTGTGGACTTTTGTTGCTCTTCATGGCGCTTTCGCATTAATAGGTTTCATGTTACGTCAATTCGAGCTTGCTCGATCTGTTCAATTGCGCCCTTATAATGCAATCGCATTCTCTGGTCCAATTGCTGTTTTGTTTCTGTATTCTTGATTTATCCGCTAGCTCAATCCCTTAAAGTACCTCCACTTTTCTTGGTGGGAACTCTTTGTATTAAATTGTTTGGTCTTCTTTCTTGACAGGAAGATTGCCGAGTAATGTGAATTAGACTTTCCTTGTCTTAATCCCAAAGTGGATCATCTCCTGAGAAATTCTTTGATTCGTTCGAGCAAGGCTTGTGTAGTCTGTGCGGAGCAAGCCGGAACTGCCTTCGTGACTACACTCCCAGTAGCCTCAGTAAAGAAGAAACGTAAGTTTTCACTCCTATTTACCTTGGTTATCATAGGACAGAATCAATGACTGACCCTTATACCAGCTGTAATCGAAGAAGACGTCTACCTGCATATCCATGGTATCAGCAGCTAGCTTGAGTAAGAATCTTACCATGAAACCGGAGCTGGAAAAGTTTGGTGCAGGTCTCATGGTATCACTTTGGCTCGGTGAACATGTAGTCCCTTACTTCTCTGTCCTCGGATTCAATATCCCTGCGATCCTTAGCTGACGGTTGCGACCAGAGATGTTTAGCTGACGTATCCTTCAAAATGCCTTTAGGTCGTAGGCAGGAATAGGAATTCGGCTTTTAGGTAAGGCTTAACCTGCCCTTTCCGGCTGCTAAAGAACAAAAACCTGGCCTCTCCACTTATCGCCTCCTAGCAAATCCCTTCAAAAGCGAAAAGGTCACATGAAGATCCAGGCTGTCGATCTCACTCATCTCTTTCTCATCGTAAGGCTAAATCGGCTTAGGTCTTAGCAACTTTGCTAAGAACAAAGGGCAACACCCGGAAGTCGAGCTCGGCGGTCAGTTCCCAGTCGCATTCCACCGGGCGCATTCCAAGTCGAGCAACAGAGCAACTCTTATTAAGTAAGGAAGAAGGATAGGTTCAGCAGGCCTTTCTATCTCTTGCATTCATCTTATCCTTATTCTTTGTTGTACTCTTTCTGAAGTCTGGGGCGAAGGGTGCCCTCCGTTTACAGGATTAAAAGCGGCAACGGCCCTCAAGTCAAGACTAGAACGAAGGTAGAGCCTTACTCTATTCCTTCGCTCGTTCGTCGCTAGTCTATTGTATTCTCAATCGCATTTTTTCTCTGTGTGGCTACAGGAAGAATGAAAGGAGTAAGTGGTGTAGATTCTACCCGTCAAGTCTGTCTTGTCTAGTTATTTGGTAAGCAATGCTTTGAATGTCGGAACGTACGTGGGAGTCGAAGGGTTGCGCCTTCTATCGATCAATCCTCTCTCTGTGTAACCTCGGAACATAGGGTCTTTCTTCTCTTCTCCCATGCCAGTCCAGTACCGAAAGTCTTCCGCCAGTCAAACAGCTTGATATCAATAAGTTATGGTCGAGAGTCGAAAAATCAATCAATCAAGACAAACTCTTCTAGAAGAGGGAAGTGGGCCAGTACTGGGAATCCAGGGTTTCGGGTGAATAATGTTCTCAATCATAAATGAGACATGAGATGCACCTACAAATAGAGTAGAACCAATTGCGAAGGCCTCTTTCTTCTATTTGCTAGCCTTACGTGTGGCTTCCGTCAAGGTATCTTCCCCATTCAGGCCAAAGTTTTAGGGCACAAGCAAGTAAGTAGTTTGTAGCTAAAGCCAATTCCGGTCAAGCGAGTAAGGTCTTGGTCTCTGCGGCTAGAGCTAGAGCACTTAACTAATATACAAGCACTCCTGCCGAGCTAGGGCCAGTAGTAACGAATTGGTAGCAAAAGGTCAAAGAGCTAGATCACTCCTAAAAGCGATCAACTATACGATACCACTAAATGAAACTACCTTCCTGCTTAAGGCACTAGTTCGGATGGAAACCCTGGTCGCCAATGTTGTCATTTCCTATGGATCCCATTTCTTCCGCAAAAGTCCCTCCACATTTAAGGAAGAAAGAGATTCTTTTTCCTCCAAAGTACCTCCAACTTTTCCGGAGAATCCAAAGAAACTGAAATTGAATAGAGAAGGGAAGAAAGGAACTGAAATAGAGTTCAGAGTTTGAGTTCAGGGAGAGGAATGAAGCTTGTTAGAAGCTTTGGAAGGAGGTACAGGTTGCTCGGCTGAAAGGAGAGGAACGAAGTAACCAGCAGACTGCGATCTGCCTACTGACGTTTAGCGAAGAATATATGCCATCCTTAAGCTCCCGGCAAAGGAACTCGTTTTAGTGCAGGTAAGTTGTTATCTGTTGAAGTTCATTTTCAAGTCACTAATTTACCCTTCCGCAACTTTCTCTTTTATCCCTAAGACTCCTAGGTCAGTTCCCAGGTTCGGGTAGAGTTATTCAAGGCAAGGGGTCTGTATAAGAACCAATGTCAGGAAGCCGGTTCTAGTCAAGTATGGCAGCTTGAAGTCTCCTATTTCACTTTCACCAGAACTCTAATCCACTTTCATTTAACGGCAGGCTAGAATGGATAGCAAGAAAAGAAAGGCTTCATTTCTCCTTGGCTTTGACTCTTTACTGATGGATTTCTTCCTCTTGCCGGAAAGATAGCTGACTTGTATAAGTCAGGAACGAAAGTAACTGCTTTTATCAAAGCAGGAACTGAGTATATCTCTTTGTTTGCTTTTTATCTTGATCCTGCTGCTTTATTTGATACGGCTTACTTTTTAGACTAAGACGGATTATCCTAGACTGGCAACTCTCACCGAAATTCAATCAACTTCTCAACTGGAAAAGAAAAAGGGTATACAGGAAAGATGGCAGGGGATCCCTAGAACAATAGAAGAAAGACTCTAATAGAAGAGTCTTTCTTCTTCGGAGCTTTACCTAGGTCTCAATGGAAAGACCGGAAGCAGGCCACGTCGAGTTAGATAGAGCAAAGGAAGTCGCAGGGGTCCGAGGACAGGCTATCCCTAAGGCCTTACCGTTAGGTAATTGTTCTATTGAGGGTGGCTGGGCATGCCTTTGGTCCAAGCAAGGGCTTTAAGCATGTGAGCTAGGGCCCAGTTAGGAATGGCTTTCCAGGCTCTATATAGCGTTAGAATGCCTTTTCAGGTCAGAATGTGGAAGATTCCCCATATTCATCGTTAAAGGTCGCTTTCTCAGTAAGAGGATCAAGTGTTGCTGGGATATTGTTTTGATAGGGAAAGAGAGGCATGACCTCTTTTTCGGATATGTCCTATGGCTAGCTATGTCTTCTATCGGGGAAGGAGACTGGATAGCAAGGAAAGGATAGGCACTTATGGGTCTGATGGTAGCGGTAGGGAATGACGGTCAAGTGAATGCTAAGGAAAGGTAAAGATTTTGACTCTCGTTGTAGTATCGACTTTGGAGTCGATTCTCCTTCATTTCCTGAAGAATCTCAGGAAGTTTTGAAAGTCCCCATTCATCTCCTGCAACATTGACACGATCTGCTCCACCTTTCAGGAGCAGGTAGCGTAATATGCTGCTCGTTCCGCAATTCGCGGATGAGATTCCCAATAGCGTTTTCGTGATCCCATATCCATTGGAAAGGCATGGAATCGAAATCTATTTGAGGAACAATATCGCCCTCATCCGCCGGCTCCGGCTCTGGAGCTGGGGGAAGTTGAGATCGAGAAGCGGTCGTCCCGAAGGGCCGACACCGCTTCCACTGCTTCCGAAGGAAAGAACTGGCAGGCGAGACCGATCGATTGAAGTTACGGTTCATAGCTAAACCCCGAAGCGAAGTAAGCAGGAGCTTCGAGGTCATAAGCCCATCTTCGATTCCCTTCTCTTCCGGTTAGCAACTAACTGGCTCTCCTACTCGGTACTCTATCCTCTTCCCATTGAATTCTATTACATGACCACATTGAATTGGCTAGAAAGGAATGGAATCTTAGTTTGGAGAACAGCCCCTTTCTTTTAGTTTCTAATTGCAATAGCAATGGGAAGTCCACTTTGCTACATAATGCCCTTACCCTTAGTCAACCACCAAGACCGAAACCCGCCCTTTCCTAAGCCAATGCTGCCCACGTTCTCTAACTAATTTCAACCCATAAAGGTAGCACACTAATCTCCGTCACCCTCGGGTCAGGTCTGGCTTAACCTTATTCCCGTCAGTTTCGTCACTCTTTTCTTTCTTTCAATGGCATCCGCTTTCAAAGCCTTTTTCCCACAAAGCCGCCCTGCTTGCAACAGTAGATCCTGTTGATTCGGTTGAAGCAGAACAACCACCAATAGTTGCATCCGTTAAAGACCAATTACCGGCTGCAGTAAAGTAAAGGGTCTGAAAGAGTTGGATAAAGACAAGACCCTTCATTGCTAGCTTCCTTGCCGATTGAGAGAAAGCACCGAAGCCCTACTCTTCGCTTGGAGTTCCCTCCTTGAATGCTAGTCTTACTTTGAGCTATAAGGAAAGGAGAAGATAAGGAGCACTTAGCCTTACTTAATAGGATCCATGTGCGTGCCCAGGATCAAGGGATATGCCTCTATATAAGAGGATGAGGATCAGACCTAAGAGAATTAGGTGAGGCCTAAGATAGCAAGTCTGGCTACGAACGAGATTTCACGTAGGTAGGAAGCCTTCGACTAAAGAAGGGCATGAAAAGGGCACATGAAAAAGAGCAAAATCCAGTGTACAAGTGCAGTTCTTCTCTTAAGAGATTTCACTTTCAGTGGTTAAAGGGTCGGATAGTTGCTGAGGTATAGCCATTTTGGTCTGGGGAATGCCTTCTCACGTAGGAAGTTCACAAAAGCGAGGAGCAAGCCAAAGAGCGAGTGAGGTCTACTCCCCTTAAGTCAAATAGGGGCCAGGCCAAGTCTTGCCTAAGCTCTTTAACCAGTTCCTCAAGGACAGGAAAGACAGATTCTATTAGATTACACTCCTATCAGTGCAACGGCCGCTTTATCCCCACCTGGCTGCTCAATCTAAACCATTAGGGGTGAGGAAGATTGATCAAACTCCCTTGGGGATAGGAACGTACTGAATCAACAGGCAGGCAGACAGGACTTTAGCGAACGAGCAATCGAACATTCCCGCTAATTACTCGTACTCCTCCTAGCTCATGATTCAGTCTGTGGTCACGGAGCTATGTAAGACCTCTGTACTAAAGTACCTGGTCCTTTACTTGACCAAGGGAAAATCCCTAAGAAATGTGCCGAGCAGAATAGCAGCATCATGCTAACATAAGGATGTTAAGCTAGCTCGATCGCAGGAAATGATGGAAAGGCTTCAGCCTTTCTCCTTCTATAGTTCGTCTGTAAGGCTTCATTCAGAAATGAAAGGGCCGGGGCGTAACCCATGATTAACTGAAAGGTATTCCAGAGCGTCTCTTTCCTTGTATATCTTCGTAACCCATCATCTGGTCTTCGATGGCTCAAAGCAAGGTCCGTGACCGTGAAGTCAATGAAGTCGCGGGGTCAGCCGGTCAATCCCCGATAGCTTTAGCCGACATTTTGTCAAGTTGAGACCCCGGAAAGGAAGAAGGAAAAGCTTTCGTGTGTAGTCAGTCAAATTCTCTTTCTATTTTTTTACGTAAGCCTCTTATTAGTGCAGCTCCGCTGGAAGAGATTCTAAGCGAGGAACAGTACTGAACCGTATCCTTTCCACAACCATGGTTCTGCCCTACTATATTCATTATGACCCAAGGCTCACTCCCGCTAAGCCCTACGGTTCCTTCGCTTCCCGCCGTTAAGAGTTCAGGCTTCTTTCCGGACCCAGAAGAGTAAGCTTTGGTTATCAGATCTCGCCTAGACTCATAGGGCAAGCACCGGCCCTATCCGATAAAGACGAACCTGCACCTGACCAATTAGATTCAGATTTCTATTTCTTTCATCGAGTGGTGTACTTTTCTTCTCCCGGTCGTTGAGCCTGATCGACTCTTTTCCCAGCCCTCCGGCCTCTTACTTCGTTTCCTTCGTCTTCTGGAGAAATATAACAAATAAGACAGTTAGGAAGAGCCAGAAGCTGGTAAAGAAGAACCGTCTCCTAAATCATCTCTACCTCAAAATACCCCAAACTGCCACTCTTTAGGGGCATTTATAGGATAGCATTCGCCACACACTCCTTGGTCTGGGATTGATCCGATCCCTATAGCCGCCTTAGTTTTAGCTTTGGAGCGATAGCAGAAGCGAAAGGGTTGTAGCGGAGATTGAGAAGGTTAGCCTTTTCAGGGCTTAGTTGCTATTGAGTGAGGAATCGAAGCTACCCTTCCTTAAGAATAAGGCAGACAGACCTAGAGCATCCCATCCCGAACTAAAGACTCTATTTTGTCTTTCACTTCTGCTTCCTACTAAAAATGTTCAATCCCTTGACCCAGCAATCAAACTACTGCAGCAAGAAAATTCTTCTTTTGGCTTGCGCTAAATAGGCTTCCGCGCTAGCACCGAAGAGCTATCGTTCTACCGATAAGACTTTATTTATATTCGATTCAACCGGAGATGGAGATATAGAACGTGTTCCGTGATGAAAGATGGAATTCTAGCTTGTCTCAGTCTTGTCTTAGTAGTAAATCCTCTCCTCTCGCTATCGGTCTCGCTGATGTCATGTGTTCGAGTGTGCCTACTCTCTGGGCTGGTTATGGGCCCCGTATGTTAGGTACGCATTCGATTCTTTCAATCGTGAGAATTTGGAGTTGAATCGCGTATTGGTCCGTATCCGCCACATCCGTATACGAGGCTTAAAGTCAAGATCTGGCAGCTGAAGAAAGTAAGTGAGAGTGACGGACTGAACGGCAGGGCAGCTTCGAGTTCCAGAATGGATCGTATTGATTGATTGGTTAGGGGTTGAGGATAGTATTGATTGGATTCATTGGTTAAGATGTCTTAATTATTTGGTTATGCCAAAATAGGCTTTTGCTCTAACCGAAGCTACAGCTTTGTTTGGATTTCGCGCCACAGAATGGATTGGATCAATGAGCTGAAGACGTCTCCAAGACTGCAGGCTCCCGCTAAAGCAATTATAGCAGCACGCACCGGCACCAATTAAGGCAGCTAGATCGATTCCAAACGTGTGATTAGCAGCTGCTGATGCAGCTGATGCGGATTACCCTGATCCGGATTCAGCAACTTATTCTCCTGATGCGGATGATTATTAGCTTTGCAATCAATCTCAATCCGTTGCTTGTGTTTATAGAACTAAGGAGAATTGAATTTGCTAACCTCAATGCACCGCACAAAGCAAAGGAGGAAGGAGAAGGGTTCGGAAGATGGGCTTGCAATCAAGCAAGAAAGAACTACGAAGGCCTGTGAAATAGTGCAGCTAGGCAAACGCTCGAATGTCGAAAGCCTGTTCTATGCCTGAATGGACCTACCTGGGGAAAGACAGGAAGGAATGTGGACGAGACAACTGAGTCTTCCGATTGAAGGTGATCTATCTAATCAGGAACCTCGCAAGGTCAGTCCAATGAGTTCAGGGAATGATCCATTCGATTCCACCCATCTTTCATGGATCTCGGCTTGGTGTTCAGCTAAGTCCAGTTCGTTAGAAGTCAGTCTAGTCTATCAGCTTATGAGAATGCCCAGGCTTGATTAAGGTAGTTCAGTGGTTCAATTACTTCGAATTCCGGAGTGCAATAGAGAGTGTGGACAGCCTCAATCCTGAAAGCAAGTCAAGCAGGGGAGAACTAGGGAGAGCCAAGCCAGAGGCGAGTGATGCGCACTCATCATACGAAAGCAGTCCATGCAGATAGAAAGCGGAGCTAAGCTAGGTGGATCCACTGACAAAATGGAAGTACCGCACTACGCTCAATTGAAAGCAGGAAAAACCACTTCACAACTAACTTCCTAGGATGTGAAAGTGTGATAGACATTCAATCAACGGATTCAAAGGCATCGGAGTCGAAAGTCTTAGTGAAGCCTTCTTAGGACTATTTCAACTCTTCTCTGAGTGCCTTCAACAGTGCTTAGGTAGCGCTCAAAGCGAGATAGGCAGCTCAGCTTACTTATTATTCGAGAGCGGGGAACTTCCCTTCGAGGTAAGCTTTCTTCTCCGGAAAACTCAAGAAAGAAGGACCCCTACCGCGGATTCTTATCTTTAAACCGACTTTCTTCCTCTTCTTATTCGCTAGCTACGAACCTCAACGAGGCATCTATGAGTTCTATCGGCATCGCACCTTTAGAGGTAAGACATAGAGGGAAACCAAAAAGTGGAGGTACTTTTCACAATTGGGATTTTTTAAATTGGAGTGGGACTGTGAGATTATTGATCTGGAGTGGCTTTTTCTCACTCGTTTTGACTCCAAACGTGATTACCTTAATGTATTGAATAATGGACCCTGGGTGATCCTTGGTCATTATTTAACTCTTACAAAATGGAGACCCAATTTTCGGCCATCTTTGGAAAAAGTTTCATCAACTCTTGTCTGGATTCGTTTTACGGGAGTGCCGATTGAATTTTTGAAGAGTCTGTGCTGCTCCAAATGGAAAACCTTGTTGGGCGTGCTGTTAAGGTGGATGATACCACCATGGAAGTTTCTAGAGGAAGATTTGCTCGTGTGTGTGTTGAAATCGATCTTTCCAAAAATTCCTAGAATTTCTTTGCTGGGCTATTCACAACTGGTGGAATATGAAGTCTTCATCTAATTTGCTTCTATTGTGGTGAGTATGGACATCGCATGGAAGGATGCCCACAGAAAGGTGGTGTAGATGGGAGTGTTCAGAATACCTCCATTAATGATCTCAGATAAGCCCACTCCACTCTATTAAAGGCTTTGCTCATATCCAATTTGAGTGCCACATGATTATTCCCTGCAGAGCTAGAGTTAAGAGTGTGAAATGCCTCAAAAGCTATTATTGAGTTATTAGTGATAAGCCGATCTTTGATGAACGCACTCTGAGATTCATCAATAACTGAAGGCAACACCTTTGATTTTAAGTCTATTCGCCATAATAAGCTTATACACAACATTGCATAAGCTTATAGGCCGAAAATCAGTCATCTCACGGGCATCTTTGATCTTTGGAACAAGAGTAATATAAGTGCCATTGATATCTTTAATATCAGCCATATCATTCAGAAAAGAGAGGACTAATTTCGTTTTAGTAATGTCCCTACCAATCTGAATCAAAGTGCATGCAGTAAATAATACCCTGGTGATTTCGTAGGGAACTTGAATTTGTACCTCATCTGCCGTGTAAGTTTGAGAGAGCGACTCTGCTTCCTCTGGTGATAGACAGCGATCAATTAACTCATCATCAATACTGCCGCCGGTGCCATTAGAGGTGAAAATATCCTTGAAGTATCTTACAGCTTCATTTGCTATTCCATCCCAAGTATCAGTAACCAAATGGGCAGCCAAACTTAAGCATTTGAAATCTGTTTTACGGGCAGAGCAAGACTTTGAGTGTTCTCCCACTCCTTCTAATAGGAGTTCTCTCAACAATGCAAGAGCCAACTTGATATTTCATTCCCGCTGCTTTTGGAGACAAAAATCCAGAATAAAGTGGTTGAAGTTGAAGGAGGGATGCTAACACTTCTTTCTTCCCACCCTACCAGCCCCTTTTTCCAAGATCTTGCTTAGCTAGCTTGCCGGCTATATCCGAAAAAGACTGAGAGAAACTACCCGAGCAACTATCCTATTGAAATCCATTTCTTTTCTCAACAGGTAATTACACTTTACACTTTTGCATCTATCCCTAGAAAACTATTCCCTATCCGGATCTTAAGTATGTCACTTCCAACAATAGCTAGTCTTGTCCTTCTATTCCTTTTCCTTTATTTCATTTCTTTGTAAAAATTTGATGTCAATTACCTGCGACGTCTTATTTCAGCCGATTCGTGCTAAGGTCTTAAATTGAATACGCGTATTCCGATGGTCGAAAATATCCATCACACCTCACACCCTGATTAGAGTTAATCAATGTCATGAAGGAGCCTAGTGTCCTATTTGACTCCTACATTGCCTACGCTTCGAACCTCCTCTCTTTCATTTTCTTCGAAAAGAAGCAAAATCCCTTATGTCATTAAGAACCTATTCCTATTCATGCGAAAAAGCGCATTGTATTCCATCTTCCTTAAGTTAAGCTTTTGTTTGATTAAACCAAGGGATTCGTGTTCAATTCCATTGCACAAGCCATTCTAAATGAAGATTAGATTGGCATCTGTCTTAGCCCTCTTTTCCTCCCTAGCTTAATTGAATACCAGCTTCCTCTTTTTTAGTATTATATGTCACTTCCTTGTCCTATTAGTATGCATGGGGCTCTAGGTTGCTTTCTTTGATCTTATCTGCTCACGAATGGTCTGCTTAAGCCAAGAAGATCGAAAAGGCTATCTCAGTGGCAAAAAAGTGCGGCACTTTCCTTCAAGCAGGAAGGATGCAAAGTAATCAAAGGCTAGACCTCAAGTGAGGAAAAACAGGAGCCACTTTAGTGACCTTTGAAAAGGGTCCGTACTAACCTTACCAGTTATTACTGCCTAGTGCCCAAACTGATTAGAGTTGTTGAATAAGCAAATCGGCTGTTTACAGTTACTGCAAACAGGAAAGAAGAGCGGTCGTGTGGAAGGTCTTTCTTCGGCCTTTGCTTATCTTATGTTATGTCTAGCCTAGACTTGAAAGTCTAAGGACGCTAGAAGGAGTATGAATAGTTGGATTCCTTACTAGGCTTTATCCCTTTCTAGGAACTAGTCAATTAAGGTAGGGTTCTTGTTAGTCTATGTCTACCCTTCTTTTCTGATCGATCTGCTTGTGCTCTATAGCATAGATAAGAT

>Decalepis_hamiltonii_chloroplast_contig12

CAGCAGTAACCTATCCTTCAACTAAGTTATAACAACTCTATCTCCATTTCCTTGAAGGCCTAATCCTATCCTTCTTTCTACTTTGAGGTCTAGCGGGAGATCAGGGTCTCTTTCGTAAAGCGAGGGTAGGGGGCGGAGGACCCTTCGTATAGGTAGTTCATGTGTATCCGGGTCTGGGGCTCTGCGTGTTATTTTAGCTTGTAAAATGCCTTATCTCTTCAGTCAGCCCTAAGCCCTTAGTCCATGCCCGAGCCTCTCCTGGTCAATTTATTGAGGCGTCTTCCCAATAGCATCCCCAATACCTTTCCATGCAGTCGTTGCATATTCTTCCAGTGGTCGATTCGGATTCGGGACACGAACCCAGGCCCCCACGGAATCTATTGTGGCCTCTGAGGAAAGAAATCCAGGTGTCCAACGGCGAATGTGTAAAGCTAGAAAGCCTGGTCTGTCTTTCACTTCTCTACAAGCTCGGCGAGCTTTCACTCAGTCTTTTAATAAATTTGGAAAATGACGAACTGGATACTATGGAAGATTCGGGGAATCTCAGACAGAGGTTCTTTTGCCCGATTGAAATTATTAGTTCGTGATTAGTTGTGTCCTCTAATTGTGATTCTTGAACCTTTAGTTGCTGCTGATAGAATTAGATCGTTTGCTCTACGACTGGGCTTTTCTCATTACACTAATAATGTAAATAACAAGATTTGGGTTTTGGCAACCAGTTCTTGATTTCCAAGTTTTGTCCTCTTCTGATCAAGTTATTCATGGCTCTATTTCTTCTTTTACAGGAGACTGTATTCTGCTCTTTCATATATGCTAAGTGTAGCTATATTGATAGGCGTGATTTATGGTCTAATTTGCGGGATTTCTCAGTTACAAATTCTGGACCATGGGTGATTGGTGGTGATTTGAATACCGTTTTATCTGTTAATGAGAGTCTTGGCTCGACCCCTCCTTTATTACCAATGAGGAGTTTTCAGATATGGTGGATTTCTGTGGATTCACTGATGTTCCTTTTACGGGCAGCCTATATACGTGGTGCCGTAGCTCAGAGACTAGGCCTCTGTTTCGAAGGCTTGATAGGATTCTTGTCAATCTCCCTTGGAAGAATCTGTTTCATAGTACTTCTGTAGAACATCTAAATAGAACTGGTTCTGATCACTCTCCACTTTTGCTTTGTATTCAGCTGATGTCTCCTAATATCATTAAAAGCTTTAAATTTCAGGTTGTTAAAGGCAGGTTTTAAAGATTTGGTCCGTTCAGCTTGGAATATTGGTGTAGAGGGTAATGGTATGCAGAAGTTGGCTGGGAAACTCAAGCACTTGAAATCTGTGCTCAAGGATTGGAATCGTGATCATTTGGCAATCTTAATAATAATCTCCGGAGGGCTGAGGATGAGGTACTTAAGGCTGAGCATGATTTCCAAACTTCTCCTTCTCCAGCCAACCGTGCTAAGCTTAATGGAGCTAGGGCTGATTTATTATTCCATTCTAGATGCGAGGAAGCTTTTGGAGACAGAAGTCTAGAATCAAATGGGCTGCTGAAGGGGATGCTAACACTGCTTTTTTCCATGGTTTATGTCAAGACAGGAGGAGTAAGCTGCAAATCACTAAAATCAGGGATTCCCAGGGTAACATGCTAACGGATGATGCTGCCATAGGGGAGAGGCTCTTGTTTTTTCCAGCATTTGCTTTCGGCTGAGCAAGGCTCCTATGCTGATATGAGTCTTCTGGATGTGATCCTTCAATCATTACCAATGAGGATAATGCTGGATTAAGTGCTTTCCTTCTAATTCAGAAATGTTGGAGGCAGTCTTTTCTCTGAGTGTTCATAGTGCGCCTGGTATTGATGGGTACTCAGGTATGTTCTACACTCAATGCTGGGATATTATTGAAGATGATGTGTGTTTGGCTGTTCGGGACTACTGGGCTGGGTTGGATACGCATTCATTCCATTTATTCTGGCGGATTACATCCTATTAGAAATCAAGGAGATAACTTAGAAGAGGGCCATTCTCTCACTCGGTTAAGAGTCATACTAACTCCCATTCTTGCTTATTAACCAAGACAAAGAAGATAGAAAAGAAAGGACACTGATCCTCCTCTTCCTCTTTGGAGGCATGTGTGGGGCTTTATCGTAGAATAAGAATTGGTAACCCAAAGAAAGAGATAAGCGATCGATAGTTGGCCTATTCCGCCCTGTCTTTGCTTTCCTCTTATCTTTTCTAATAAGAAAAGATCTACGTGCGGATAAAGGGCGAAGCGCTAGTTACCTAATAACATCCTTCCTCTAATTCCTCGCTGGCGGATCTAACTTTCTTTCTTTTCTTTCTTTTCTTGTGGGTGTATTCCATTATCGCTCTGTAGTGTACTCAGTAGGCCCTGTAGCTGTAGTCGCTTATAGTATAGCTAAAAGAAAAGGAATGAAATGAAAGCCCTTGCGAGGATGGCCTATAGAAAGACATGGGTATGGCTTCCTCACTGCAACGGTATTATAGAAATATATAAATACGGACTCCCGGTCTTCCCTTCGCGAAAGGAAATAACTAGCGCAAGCTGCGATCTGAGAACTTTCGAGATGTGAATCATAGCCTAGTCTAGTTTCGTACCCAAGGGATTACTCGCTAAAGGAATGCTTATGAAAGCGTTTAAGCTGCCATCCTGTTCAACAGTTGAGTTGCAGGAAAGAGATAGCATTCGCTACGTGAAAGCTAAAGTAGAACTCCTTACTTATTCTTTTTATACCGTTCATGGCATCGAGAAATACATTGTTTAGGCTACAGACTTGGTTGGGTTCGTTCACTCAAAAGCACAGGAGCGAGGAGGCCATCACACGATAATCGTTGTCTAGGTCCGGAACGCTCGTGATCTTGAGATTGACCCTTACTGCGAACAATAGAATATAAGAACAGAGAATCACATTGCACTAATACTTCCTTGAAGCTAACCAGTTTCAACCCTTCCTTTTATGCTTCTTTAGCTTAGGGCTTCTTTATAGCTCTTATAGCTAGGCTCTTCGCGCTGCACTGCACTAAGCTTTCTTTCTTCTTATAGTTGTCTCTTTTACAGCCTGCTTTCAAACCTCGGTATAACACTGGGTGAAGGCCCCAGAAAAACAGATCATTAGACAGGATAAGAGCTGCGATAGTGCACGGCATCGACTTTCTAGGTCACGGACTTCGAATAAAGCCATACTGCTCTGTGGGCCTCAGAAATGGCCTGAACGAAAGCGAAAGCACTTGGCGGAAAATACTAACGCCTCCCTTACTACCACACAAGTTGTTCACGTCGACGTGGGAAATTCACCCTTAGTTCACTTTGCCTCGGCTGCTCGATCATCTTCGGCTAGAGCTTTAGATGCATTCTTCCTCTTTCCGTCCCATACCTTATTTCCTTTATTTTGTGAAGTCACAGCCAATGCTGAAAGTCATGGTGAGGAAGCCACATCCAGCACATATAGCCCCCCACCTCTTTACCGCGTCCAATCGTCTTCGCGGTCCTGAACGACACAGCAAGAAGGCGAACTCTTTCTAGTTTGACTGACTAATAGAATAGAGAGAATTTTAAGAAATTCAAAAATGGATTCCCTCTCCTTTAGTCGAGTCATTTCATCCCTCTCGTAGTCGATTCGGTATGTGTAGTATGTACCCTAGTCCCTAAAGACTGCGCATTTCATTCTATTCTTTCAAGTCCCACAGTTCGAAATGTGCCTATTCCTTCGACACCTTTCCATGGAAACCGGGGAATTTCGTCCTTAGATTTGACCACCGGACCCCCACGAACTGAAGACTGACCGAATCTCGATTAGGGAAATGACTCTTGAATCAGTCTCTAATATCTATTGAACTTTCCTTGCTTGTTTGCTTGTCTTATAAGGGTCAGGCTATCTCAAGAAAGAAAAGAAAGACGGCCTAAGAGGTCTTCCCGCCCCGAAGAAGGAGCTAGACTGAGGTAAAGCACCCAAAGCTACGTACGATGCTTGCAGACCTTTCCTTTACTACGATTTTCCCGATCTATAGAATATGCGATAGATGGCTTGCCATCGCTAGGAATTCTAAATGTCATATATAAAGATCAATCACGCCACCCTTAGAGCTCCATCTCATGGAGCGAATACTAAAGTGCCTGGAGCGAGTCATAGAGAAGAAGGAATAGTAGTTCTAGATAGAGCACCCAAAGGAATAGGTTCTAACTTTGCCCGCATCTGGCCTCTTGACTGATTTTTTCCTTTCTGAAGGGGGCTTAAGACGAGAGTTTCGCTCCTTGGTTCAAGTATTCAAGTACTTCTTTTTCTTTCGGTTGAGACTTTCACTATTCACTCTTTCTTTCTTAGGCCCTCTTTCTTTTGCATCTGAAGCTCTGGTCTCTTTGGGTATTCTAATGACCTCTTCCCTTCCAGTCGCTTCCTCAGATTCTGGATCCCTCTCTCAGGTTCGTCTCTCCTCTCAAGTTGCTTATCCTGTTGAAGGAATTTTTCTTATCTTATGAATTGTTTTCCCAAAAGATCGAGCTTACCTCATGTCAGTTCCATCATCTCTTTTTTTGAAGGAAAATAAATATTATTTATTAAGATAGGTCCGCTAGCGCGGCATGAAATACATCAAAGGGAAATCTTCCCACCAAACACAAGAGGAGTGAAGTCTCGCCTTTTTTGTGCTAACAAATGTGCGGGAACATTACCATTACGACCTACATGAGAAAAAGAAATAAAAGAAAGGCTACTCGAAATTCTTTTGATAGAAGAAAAGAAAGAACCAAGCTCAGATAAGTCATTATCTCCAGAGGAAAGTTTGTTGATAAGAATGAGGGAATCGCTCTCTAAAACCAAATGTTCGACACCATTGCAGTGTGCGAACCTTAATCCTTCCAATGCTGCAAGGGTTTCCCCATGCAAAGCAGAGTGAACATGAGATACTGTCTTTGTCATTGCAGCTAGAGTTGTGCCGTTGTGATCCCGTCGAATGGCTCCAATACCACAAAATCTTCTGTCCATGCTGCATCTACATTGAGTTTCAGCAGTCCCTCGCTCGGTTTTCTCCATTTCTCAATGCCTGGAGCTGCGAGAAGCATTCTGGATTTTCTGGCTTCCAAAGCGGATAGGTTCTCTTCCGCGAAAGCTTGGATTTGCTGTGCTAAAGACGATGCATCAGTACTTTGACCTTCCAAGCATGTCTTGTTCCGATTGAACCATAGAAACCATAGAGTAGTGATGAAGAGGGAGACGTTAAGTTCGGTCTGGCTAATTTTAGCAAGCATATCTTGGAAGCTACTGCCAGTAAATGATAGCCATAAGGTGCTGCGTGGGATGAAGCTCCAAACTTGTCTTGCTGCTGGACAGTCGAAGAAGGTGTGCCTTGCGTGTTCTGGACTGGAATGGCAAGCTGGACACTCTTGAGAGATAGAGGACATTTTTCAATTAATCTTTTTAGGTTGGAAGGATATCCTAACTTCGTTTCCACAAGAAACCCTTGAGCTTTGGTTGTGTCTTTACATTCCAAAGTGCTTGCCGGAATTGTCTCTCCTTTACATCACTTGAAGATGATGCCGTAGAGGCTTCTTGCTTTGAGATAGCTCGTCTAACTTTGTATCCATTCCTGACCGTGTAAGTTCCATTTGGGGTATGGTGCCAAATGAGTTCATCAGCAGGGTAAGATTTTGGAATAGGAATGCTGAGAATGGTTTCCACTTCGTAGGGGCTGCGATAAAAGGCTCCCGGGAATAGGGGAAATATAAGGTTGCTTTTGTGGTGCCGAAGCACTCGCCTTACACGAGAGGAGGGCCTTCTCTTCTGATAATCTAATAAGTGGAGCTCCGGAAAGCTCAACCCGTGTAACTGTGATTTGAAAGGGAATTCAAAAGCGAGTAAACGATAGAAGCGAATGAAGATTTCGATTCTATACTATCCAACCAAGAAAGAAATGGGAGTCTTTGGGCGGGCCGAGTTAAGTAAAGACTGGCTACCTAGTGATTTACAAGCACCTTCACTGCACACTTTCTATATATCTGTCTCCATCCAATCAAAGACGTCCATACCATAGAGAATCAGGCTCGGCAAAACGAGACTAGGGACCATACTCCCCGCTTTCCTCCTGTGGTTACTATTAGAACACAAGCCAAGGAACTCAAAACCACAACACAAGCACCCTTTACTATACTATGGTCTTCGAACCTTTGCTTACGGCTTTGATTTGAAGGTAAATAGTAATTTCATTCCTCGTAGAACGCGAGCGTTGAGGCTTTCATCGAAAGAAAGAGAGAGGCTTTTATTTGTTTTTTCTGTGTTAGTCAGGTGAAAGGCTTGCTTAACTATAAATAGATTAGTAATAAAATAAGGTATTAAATGTTGGAACTACTAAAACCGCTATTCCTGTTTTTGGGCTATAAGACTCTTAGTTTCCGGCGAGCTACGTGCATAAGCTTTTTTGACTACAAAACGATTCCTCCCCGGAAAAAGTGGTCCCTTGCCTTTTGTTGTGATAGGGGTACCGCCTTTCATTCCCACCAAGGAGCCGTAGCTTTACTTAGCCACACCAACCCTTTGACTGCCTTACCTTCTATTATATTTATTAGTAAAGGGCCTTTAGGCTTTACTATTACTAATATAATATCAAGTAAAGGGGCATGTATCTATTTTTTTGTAAAGAAAGGAAAGAGGGCTACTTCACGAGCCGCTACTAAGAATTCTAATTAATCGAAAAGGTTCACAGCGAGTTCAGTCTATAGTGAGGGAACGTTTCCGTCGGAGGGGTTTGTCTCCATGGAAAAAGTATTCACTACTGCTTACAGCACCTTCATTCACTCGTCTTGCTACTTCGGGAACTGTTCAGCGGACACGTGGAGTCGAACGTCTCTCTACAATGTAGCGTAGGTAGACTGATTATTGTAGGTGACTGTCTCTAGTGGTTTAGAAAGAGTTCTGTTGCTACCAATTTGTAGTCTAGTAGATCTAAAGTAGGTTTATGTTCAGCAACAGAAAGAGGTGAGGTTTTGCTTTCTTACCCGAAAATATTTACAAGGGAAGCCTCGAAAGGCATACGAGTGCGCCTCTCGTAGACGAATACCGACTCAGCCCGCTCCTACTGCGCGGAAGTATCAGCCGGTCGGTGTCGGGTCGGCCCCTAGGGCACTATTCGCCTTGGGAGTGGTTCGGCCATCAAGCTACTTTGTGAGGTAGGGGACCAGACTGGTGACTGCTGCAGTTGTTGTCTGCAGGGTATGTGACACCCCACAAGTGGAGTAGTGCTGAGCCAGGAGATCTTCCTTTGGATCGTTACAACGCTCGTTGAACGCTTCAAGTAGAAGTGAATAGGTAAGAAGTGGAAACCCCCATATCAATCCATCTCCATAGGAGACAATATGAGCAAAGACAGGAATCCCTAAATCCCGCAGAAATATACTAACTCCGGGGGAGTGAGGGGTATTAAAAAGGGGAGCCGCGGGTTCTTGCTTTCCTTGTAGGAAAGGTCTGGCTTTGGTATGGCATTAGAGAAGACTTGTTATGGTTATACGGCTATATAGAAAAGGCGGGTGGGGCTTCCGGAATAAAAACCTTCATATAATCAAGAAAAATTCCATCGATTGTAGCTAGTGATTGCCCGGGTCGGTGTGTGATCACCTTTGGTGAAGCAACCCTTCTGATGCTCTCCGCCCATGCCGAATACCCCCGGCCGGTTCCGCCGGGTAAGGCCAGGAGCTGGTTTGGGGAATCACGGGGCCAACAATCCTTTCATTTCACACCCGCACATGTGGCTGAATATATAAGGAAGTTCACCCCTGGAAAAATCCATCCTGTATCCCTTACTTGCCAGCTTGATCGCTTTTCTTATTTACCATTTTCCTTAGCGGGTGGTTTCTGTCTGGGCCTTTCGGGTCGCCTGACCTGGGCTGATGGCGCTTCGTAACGGCTTGTCTGGAGTCCAGCGGCTCTCTAAGGCTTCAAACTACTAGTCATTAAGACTTACTAAAAAGAAAAGTAAAGTCCTTTTCTTGACTTGACCTGCGTGCATTATATCTACCCCTTTTTAAAGAACTTGATTTCAATCTCAACAAAGAAATGAAAGCATAACTCTTTCCTTCTTGTCCTCTTACTCTTTTAGCCTGCCTTGTTTGTGGAGGTCTCTCGGGTGTCTACGGCAGATCCGATCAGTCTCGTGATGAAGAGAAGTTTTTCCGATCTTCCACTAAGAAAGGTATCTCGTCACGACAACGCAATTTGTCCGGTAAACTACTCGAGGCTCCCAAGGTTTAGTAAAAAGGGAGGGAGATTTTCTCTTCATCCGGGTGTTCATTTCATTCATTATGAACTCAAAAGTGTAAGGCTGATTAGTGAGGTCTTAAAAGGTCATACAATGAATGATCGGCCATTCCATTTTGCTTTCAGCAAGTAGGCGACGCGAATCTATGCTTTCTATGTTTCAATCGAATACCAATTGCTTGAATGCTACCTAAAGGCACCTCGAGATTACTTGAAAAATCAAATGCTTTTAGGTTTGTCTTGTGTCTCCGAAACAAATGGTCCATTTCTTGATGGGCGAACGACGGGGATTGAACCCGCGCATGGTGGATTCACAATCCACTGCCTTGATCCACTTGGCTACATCCGCCCCTACCCCGCACAGGTTAAAGTCTCTATCTACGATCAGATCTTTCTGAACTCCCGCTATCAAAGATAAGCTTTTCCTATACTATAGAAAAAAATTGTTGTGGGGAAACAAAGCTTCAACATATGAATCTGCCTGGCAAAGCTTCAAACAAAGAGGTTGGGCTATTCACTTCATTGATTTTACTTCACTTTACTTAAGATTAAAGATAGGGGCCGGGCGGGCAGTGAAAGGTTCATTTAGTAGACAGCGAGCGAGCACCCGCGCACCCCTACTAAACTACAAGCTTTCTTTGAAGCCTTTTTATATTCTACAAATCTTTCTATAATATAAGAGAAGCTCGAAGAGCTTTCTTCGCATGCTTGAGCGCATCCCTTTCTATTAGTAAGGTTGTCAAAAGGCTTTCCTTTAGTTCCTTCTTGCCTTTACTAAGATTAGAATCTAATTGCCCTTCTTTCTCAAAGTCAACTTTCCCCCTTCTTGCTTTAGTTTTCAATAAGGGGCCGCCTTCCTTCATCCCTATCTATTCATCTCTGTTTTCAAATGGATATTTAGGGATCTCTCTTGTTCATAGATCTTGAAACCAGATCAATATCCATTTCTCAATAGATCTATCTATCGATAGAAAGATATAGATCTCTATATGGAGATAGATCCATATCTAACTAAATATGGAAGACCCAACACAACAAGGGCGTAACCAACGGAAGGTTCTCACCCTGTCCCCGACATTGTTCTACGACGATGTCCCCTGGGCGAAAGGAGCCACCATTTTCTTTCTTTCTTCAATCGTTCCGATCAGGACAAGTGGGTTGGTTCCTTTCGTAAACCTATCTACGCAATTCTCTGTGATCATGTTGGGCGAAAGGTAGTTGTAGAGGAGCGGCTGTAAACGGCGATTCCCCTTTCCATTGAAGTAGGGGGCCTCCTTCCCCACCATTCCGGCCTATTATTGATAGGGATTTCACCGATGCTGAAGGTAGCTTGCTTACCAAGCCACCCACTTGAGAAGCTAGTGTCGCCAGAAAGAAGCGACGAGGAAGCGAAGCTGTCTACGAAGCTTTGCTTCTCCTCCTGTAGTCGTAATACTTGGCTCGTCGCTACTTTGATTCAAAAGGTACGGTTCCCGACTTTCTCCGCTTTCCGCAACCGTAACCATTTGTCATTCCGATTACTTCATCCATAAACCATTTTGGATTATTTCAAAATAGCGACAGGCTCTAAAAAAGTCAAGGATAAGCTTCCATTCTAGAAGCGGTAGCTTCCCGCCTCCTTCGGTGAGAAGTTCCTTCCCTTTTCTAAAATGCCTGATTGGTCTGCTCAGCAAACGTACAAAGTGGACCGCTGTTTTGCTGACCCTCTTCTTCCCATTCGGGTTGGGTTGACCCCAGAGGTAGAGTAAGAAAGAATGGAACCACTGGAAAGTCGTCTGCAGTTCACACTTGGGCAAAGACGACTGGACTTTGAAGCGGAACACGAACCGATTTCCGCTATTCCGGGTTCTTATTGAGCGTAGCGAAATCAAGGTTTATGATAAAGTCAGCGAAGTTTCTATGGTGTTCTACCTTTGCGTAGTCTCGGTCCACAGTGGAAGGCTCCGCACCTGAGCCTCGTTAGACTCAGTAAGGTGTAAGTGAAGAGAATAGAAGAGATGAAGTCCGCCCCCTTAGCCCAGTCTATATCCACAGCTGACGCAACTCCGTACCGACGCCTCACTACTACAACATTAATTAACGGCGTCGCGATTTCATAACCTGAGCTTTCCTTAACCAGCAGTTGACTTTACTTCGTAACCTCAACGTACTTTTGACTGTAGCATAGGCCTTCGCCACTTCAAAGGTTTGGAACCCTTTTTTTAATTAGAAAGAGCGGGGTCTTACTTATTCAGGGGATGAAAGAAAATAAAGGGATCGGAGGGCTTTATGATCGGAGAAAGAGAAAGGGCGTGGTTGGTGTGTCACTGGGTCGGTGGGGAACCCGTAGGAAGGGGGCGCCCCGCCGAATTCACATCAGAAATCGCCAACATGAACACAAACGAAATCTTTAATTACGTATAGAAACAAAACGAACCACTTCTATTCTCGGAGCTGAGGCTGCTAAAGAAGAATGGCTTTTGGTCCCTTTCGTCCAGTGGTAAGGACATCGTCTTTTCATGTCGAAGACACGGGTTCGATTCCCGTAAGGGATAGGTACTCATTCCCGGCCGCTTTCAGTTAGTGTTCATTGCTGAGTGATCGCTCGCTATCTGGCTGGTAAAGGGTGGTCCGGCTTCTTCTCTCCCAGCAAGCAAGACGAGATCACCACTTCTCTCAGTAATGGACTTCCTTTACTTCGTAACCTTAGCCTTAGATGTCCTTTCATAGATTCTTGAACCTCCGACAGACAGAATCTCCATGTATGTCTTCTTTCTCTCCTCCCTCCCTAATACATAGTTCCGTCTATGGAGCCTAAAGCTTCAACCATGGCATGGCAGTAAGTTGGCCTAGTCTCTTCTCTCGCCCAGCCTTCGCCTTCTTCAGTTGCCAAGTTGAAGCCTTCAGCGGTTCTTCGGCCTACCACCTTTCTTTTTCAAGATTGAGCTTGTTCAAGAGAAAGCAAGGATGGTCGTAGATCATAGCATAGCTACCGGCTCAAACAAAGAGATTAGCCTCTTGATCGATCGATTGATTGGATCGAAGTACGAAGGGTTGATTGAAGTCTGAGATCAGCCCAAGACTAAGGCCGAGCAACTAGCTGATGCAGGATTGGACGTCTATCTATCTCACCACTCCCTACTCCTATAAAGGCCGGCGGAAGGAATGAATGCTCTGCTCATCTTCTTACGGCTCGTTACCGCAGCGCTCGTTCGCCCCTTCGGCTTCTTCCATTCAGTAGTGTCTTTTCCTATTCTTATTGGTAAATAGCGTCTTGAAGTGAAGCCAAGGCATTATTGAAGGAAAGAGATGGCGGGTCGGTCAATTGCATTAGGTAGGAGATGCAGGACCTGTCTTAACCGCAAGTGCATTCGCTATTCGATTCCATCCTCAATCCCAACAAATTAGGATTCCAAAGTTTTGATCTCTTCTTGACTTTTAAGTGACAAGGGAAAGGGTATACTTTCTTCTCTATGTCGCCGTCTCATCAATGAGCGTAGATTAATAGATATGGCTTTTGTGCTTGTCAATCTGTATCCCATTCTTCAGGTATAGTTTTCTTTGATCACAGATCGACAGGCTTCCTTCCTCTCCCTGGGTACTCGCTTATATCGTTGGGTCTCGCGTCTTCAAACCTTTCTCCCCTTTCGTCATAAGGCGTGGTCTGACTCTGATAAAAACCATTCTTGTGTTTAGGTCCCTACTAAGCAGAATTCGTAAACTATGCAAAGGCTATTTGAATACTTTTCAAAAGTTTTTAGCAATAAAAGCAAAACGATTCTCAACGCCCTTACGAGGTAGTGATGAGTTAACTACTCGTGTTGGCATGGAAGTCAGCCCTGATTCCATTCTGCTACTAGGGTTCAAAACCTTCCCCTTAGCTCTATAACTTTTCTTATCAAGAGATGCTAAAGCTATTTGATTTGAATTTAGACTAGGTATTTCTAAGTCGGAAGGAGGGCTTTGGGCAAAGAGCAACTCGAAAGTACTTGACTTCAGTCCCAGTACTGAAAGACGTGACTTCAGGGAAGGAAAGACTTCGTTTACTTCCTGCAAATTGCTTATGTAAGAACTAGTAGAAAGAAAGGAAAATCAAAGCAGCATTGATAGACCGTTGAATGAGAATGCTTGAATGGGAATCGTCTTAGCAACTGGCTGTAGACATGAGTCAAAGCCGCCGGGAGAGAATTTTCATGAGAGAGGGACGAGCAAGTGACAGATTGGGAAAAATAGGTGGCCTTCTGACTCCACTTGCCTCTACTTTTCATGTCAAGCGTACAAGTGTAGTTTAGTGGGATTTACTTCTAAAGAACGGAATTCTTTTTCCCTCGATAACCTGATGAGCTCAGTTGATTGTTGAAGCAGTAGCTCTGGATCGAGAGCTGGATGCTTACCTTATTATTATGAATTCTGAAAAGGGGAAAGGATTGAAGAACTTAGTGTAAAGCTAAGGATTGAAGAACTTAGTGTAAAGCTAAGGATTGAAGAACTTAGTGTAAAGCTAAGGATTGAAGAACTTAGTGTAAAGCTAAGGAAAGGGCTTTTTTTTTTAGATTAAGAGGTGAGTAAGGGGTTTGCTGGCTTGCTCGTGCAATCAACGAAAAATGCCTTCGCCTTAAGCTAGCTTTGTAAGCTAAGCAAGCCTGGTTCTCCGGGCACTACGGTAGGACGTGGATCGATCATGACGAGAATGGACTTCTCCGTAATCTAATGTAATAGAAGAGGCACAGTAGTCCAGTTCCCCGGGCTTTCTCCCTTCTCGACCAGACGAAGAAGATATGAATTCCATTCAGGCGGGTAAGGGCTTGGCTTGACCGTGTGTTCTCTCCTGGGGGCGAAAACTGGAAAGTTCATTATTCAGTGGTGGGGTGTTCATAGAAAAGAAGGCGTCGCCCAAGGGGGGCAGATCTGGATAGAGTCTCGCTCATAGAGGAAGAGTACGCGCTAGCTTACGGCTTACGCAGTTGATCGGATCAGATAGCCCTTCAACCAACCAAACCCGGCACATCCAATTCATTCCGATCAACAACTTGGAGGTATGGCTGAGTGGCTTAAGGCATTGGTTTGCTAAATCGACATACAAGAAAATTGTATCATGGGTTCGAATCCCATTTCCTCCGGAACGGAAATGAAACGGGCAGGCGAAATTACGTGAGAGAAAGAACCTCTTGGTGGAGTCCCCCGGAGAGAATAGCACTACTTAGTGAGACGAAGCGGAGAGCCCGTTGCGCCTTGCTTTTATTTGACCGGCCTATCTTCTTTCTATAAGCAAGCTCCTGTAGGCCGTCCAGTCCCTTTAGGTTTTCGGTTCTTGAGCATGTTGGGGATTAGGCTTTTCAAGAGCTGCTCGAAAGCTTGACGAACAAGCGAAAAAGCCTATCTATTTTCTTAGTAAAGGGCTTTTCCCTTACGGTAAGGTAGGGCGCTATTCGATGAAGAAAACAGACTTTAGGAAAGTGGTTTAGGTAGCTCAGCTGGTTAGAGCAAAGGACTGAAAATCCTTGTGTCAGTGGTTCGAATCCACTTCTAAGGGGTCCAAAGGGACACGTAGCCGGGAGCGAGCCGGATTTGGAAATTCAAGTGAAAGAGTAAGCCAAAATGTGAGCGCTCCTGCACTATACGGCTTTTTGTTGCTGGAGGCAGATTTTCTAAAAAAAGCGGTTGATTACTCGGATTGGTTCTCGCATCCATGTGCCCAAAAGGATGGGCGAGTTCAGTTTGAGTGTTCATCGATCGGGTGGATCTATATGCTCCGGGGTGGTGAGACGAGGTGTAGCGCAGTCTGGTCAGCGCATCTGTTTTGGGTACAGAGGGCCATAGGTTCGAATCCTGTCACCTTGATGTGATGGGCTGAAGTGCACAGCCCAGCCTATGAAGGCAGACGAAAAAAGGCACATTTGTCGTGAACTGAACGGTAAAGCATGAACATCTTTCTTTCTATACTTCTTTCTTGACTCGGTAGGGCTCTCGGTGCCTTTTGTAGCAATTTCATTTGGACGTTTTCTAGGATCAGAAGGAACTTATAATGACCACGGGCCGTTAAGGTCTCGTGCTTTTAACAATACTGATTTTCGGTCTTATATTTCTTTTCGTCTTTGTTATTTTCTTTTCGTTTGAAAATCAAAAGTACGGCTTTCACCTTGTCTTGATGATTTATATATCAATTCTTTTTTCATCTCTTTCTTTTGCTATTTCCTACGAATCTACCTAGTATCTCGCTTCGTTCTTTTTGTTCTGATCTTTTCTCCTCTGTTTAGGTTTTGAGTGTTCTTTCTGGGGAGGGCACTCACTTCCTCTTCCGGCTCCTCCGGCCATCAAGCTCGTCCTCCTATACAAACACAGAGATTCTTTCGCAATTCGGGTCTTGTTAGAACCTTTTTCTGATACGGAGATGGAGGCACGTTAGTGAATCAGCCAGAAGCTAGACTCTCTGCTAATGCAGTCGCTTCCTCGGGGAGGAAGCTGGTCCATCTAATCGGACCCTCCCAGTGGTCCCCTATCTGTATCAACCGGATGACGTAATAGGGGGATTCCGTTCAGTCGATCCAGCAGGCTCCTAGCCAAATACTCCTTTCCTTCTGCCGAGGACATACGACAGGCCGAAATGGAAGCCGAAGACCTATTCGAGGTCAAGGTCGACATTATCAGGGTCATGTCTGTCCTTGATCCAGAAGGAGATTGGCTGGGACGGGAGCCCGGCCCCGAAAATTCCCGTACCTCCACGGGAGAATACTCCTTTGAGAAAGTCCATACCCTTCTTTCCGATCTGGAAAAACGAAGTCAAATCAGGCCTTCTCTCAATTGAAAGAGAGGGTGCCTCTGCGAATAGATTCGGATGAGAACTCCTCCGCATAGCCTGTACAACAACCTTAACCACAAGCCTGGGCTGGGCCTATCTCCATCCCTAGAGGAGCCGTATGAGGCGGAAGCTCCACGTACGGTTTTGAAGCCGAGCCTTTCCAGCAATGGGGCCTAGGGACCGATATGATGATTGGTTTAGGTAGGGCGGCCGGCCTACCACGGGCACCTGTAGGGATTAGTGTGTGAGACCGCGATCCACAAACTGACGCATGGGACTCACCCTTTACTTGGGAATAGAGAGGGAAACATAGCATGTCACAAGAGCGAGGCGAGGCGCTTAACGCCCTACTGCGAGAGGGACGCCTCGCGAGCCGGGCTTTTAGAGATGAGGCCTTTGGCGAAGCCAAGTCAATTTCAGGCCACCAAACCCTGCAACTGATGAGAAGGCCCTACGGAGTCAAGGGAGTTGTCACTCCCTGCCTTCCAAAGGTGCCTAGAGGACGGGCCAGACACAGCAGAGCGACACCCGGAGCGGATTCCCCACCGGCAGGAGGGCAGGAGACGGCCATCTCAAGGCACATCACGACCTACAGGCAAGACCGGCGAGACCTGGGAAAGCAACCCGATTGGGAGTCAGAGGATCCATAGTACTTGCAGTCTCCCGGACTTCATATTCATCATTTTGAAAGCGGGGAAGGGATCTCTTTCTGCAACGGAAAAAAACGGAGCAGATTTGACTCGGCACAACCTAACGATACATCCAATACCAATGATCTGTGCCTAGAATGCGTTGCTAGATCTCTGCTCTAGAAAGCTATACAGGCAATAACGAACGCGATTTCACCCCCTTTTCTTACTTCTTTCACGACGAGAGAACCGCGTCCGCATCAGTCAAAGTGGTGGAAGGCCCCCTGAGTCAAAGGGAGAGCTTCTGCACCTCTCTCCAAGAGATACGATTCGGTATCTGAATCGGCTGAAAGAGAGTGAAGAGGCGGCTACTTTCCCAGCCCAGCCCACGGAAATCCTCTTCTTTCTGGTCTACATCAAGTGCATTGAATGCTCCACAATCAGAATCAAAGAGGATGGGTGAAGGGGAGGTTCGGAACGAAACCGGAGTAGATCTGCGGCAAAGCAGAGCCCGGAGCTTACTTCCCTACCTCCGGAGGGAAAGGGATTTCGTTTTTAGACTTTGGAATTCGACATGGGAAAGAGATAATAAAAGTACTTTCAATGTGCACAAAAACACAGAGAGAGGAGGCCACTAGTCCAAGCATCAGAGCATAGGAGGTTACTTCTTGGGCTGTGGGAAGAGTGGAGACAGTGGGCCCCTTGATGGCGTTCGAGAAAAGAAAGAATCAAATGAAAC

>Decalepis_hamiltonii_chloroplast_contig13

GTCACTTCAAGGGCGAAATCGAGTGTACAAGTGTAGTTCTTCTCTTAAGAGATTTCACTTTCAGTGGTTGTGGTTAGAAGGTCGGATAGTTGCTAAAAGGTATAGCCATTTTAGGGGCCTTATAACGTTCAAATGAAGCAGGCCATCTCTCGAAAGCTTAAAGTTCCTGAGGAAAGTTCTTCTTATAGCGAAGAGCCTTAACGCCCATACCTGATCCTAGGGGTCCAATCACTTCCTCAGACTGGGGGAGTAGGAGAGCTCACTTACTTCAGCTTCCACATCTCGATCCCTCTTCTTCTTTTTATTAGACGTTCGGGATCTTCAAATCAATTGTCGTAGGTAGTGTCCAAAATCGCCGATGTGTGAGGCATTCACTGTAGTTACGTCCTCGGAACCAATCCCCTAGGGTACGTTTCTGCTCGGTTTTCTTTCAACATTTCAATAAATAGATTTGAGTTATTAACTCAACCGAAGTCACTCATAAGCCAATCGAAAGGAATTCCTCCAACTGACCAAGAGCACACGAAAACCTGATCTCTCCTTGTTTTATGCCAGGCGAGGAAGAAACTTCAGTCAGATGCGAAGAAGACAACGAACGTTTTGGGGTCGATTCTGATTTGTTAGATAATGTTCTTTCCGCGAAGCGAGTTATCTTATATAATGAGATGAGGTACTTTTTCGATCACCTAGCCTTATTGTTCAAATGTCGTGGTTCGCTATCCTAGATCAACTCCCTCGATCCTCAAGCCCTCTCAGCCAAAGGCTAGAAATAAGCTCAATCGAGTACTAAATAAAAGGATCTCTATCCATGCCGGATAAACGAGAAACTGAGAATAGAACGAATGTTAGGTAGCGCACTTCTGCCTTAACCGGAGGATAATCTCACCGTTCAAGAGTATCTGCGATCAAAAGTCAAGATACCTTTCGAGCTGTAGCTTTACAACGGTAGGTAGGGCTTATATAGAGGCCCTTAATCACCACCTGAAAAAGAAGTTTTGACTTTGAAAAATAGGCCATACAATCAACATCTTAGGGGAGAAAGAAGAAAGCAGGTACGTAGTTCTCGACTGACAAGGAGAGGATTGAGACCGATAGTTCAAGGTTTGAAGACGCTCACTAATTAGCAATCCTTCCCTCAGTCCACTCCTAGCGCTAAGCATAGAAAGCTCACAAGAAAGCAAAGCCTTTTTCCTTTATCATGGTTGGGGAAGTCAAGAGTCAGAGCTGCTATGCAAGCACACACATGGGCACTAGTCAACGCCCTTGCTTTGTTTGGTTCTTAAATCTTGGGTCGACTTACGTAATAAGGGCAACTCGCCCAAAAGATGAGATGGATGCTCCTTTCACACTATCCTGTGAGTCTCTAATTCAAAGCTAACCGAAGCGTGACTTTCAAGAGCATTCCTAAAACGCGCGAAGAAAAGATAAGATAAATACCAACCGGGGTGAACCCAACAGGAGCTCGAAACCGCCGATAAGGGCGAGTCCATCGAAACGAAGCTAATTATATATTATTCTAGTAGTAGCACTGATTGCCACTTATGCCTTCTTAGCCGTGTCGTGAAAGTCCTTTGCTCAAGTGCTCAAGGAAAAAGGGATCTTTTACCCCGAGGGTGTAGAGTTCTTCTCACTCGTATTTGCTAGTGGTGAATCATATCTATATTTATAGAAGTTAGCCCCTACATTTTCATCGTATGCTATAGCCCTCTCTATCTTTGTTGGCGTAACCGCCCGGGCGTATGTAAATGCCCGGTAGTATGAGCTACTTCCTCTTAGAATGATCACTAGCATTACCTATTGAAGACTTTCTTTTCCCTCTTTCACGCCGACTTCCCTAATACCTCTTTTCACTCGGCAGTCTGTCAGAGTTAGCGCTTCGGCTCTGATTTTATCTCTTATCTTTCTATATGATATTACTCCCCGGAACAAGGGGATTGAGAGCCTTTATTTCTAAATGACTTTTAGCTCTTTTACTAGGATAATCCAAAAGCCTTTGTAAAGTCTGTTGGAGGTGCAAATTACGCCTTTACTCTAGCTGCTCCTGGTGAAGCAATTGATTGTTCACCTGAAAAGAGAAGAGCAAAAGCCAACTCGAAGTGGTACGCTAAGTTTCATTTCTCTCCAAATTCTTCTAGTCGAGCTCCCTGTTGGATTGGTCGAGCCTGAACGTAGGAACTCTTTCTTACTCATTCCTCTCAAAGTCATACGAGCTAGCTGCGATACCCTTTCGATTACTAAAGGCACCTGGCTTGGCCTTCTTTGCTATTAGTATCAATATCTGCAGCTTTTCCGCCAATCCTATTAGAAGGAAGAACCCTGGGCTGAAAAAAGTATACTCTTTCTCTTTTAGAAATAATAGGTCAAAAAGAGAGCACAATCGCGTTAGGCAAAGGGGTCTAAAGGCTGCTAGTTGCTCTTGGATATCCTGCTATTTAAATTAGCCGGTATTCCCATTTAGTTTTTCTCAAGTTTATTGGTACTGATGTTGCTATTGAAGCGGGGTTGCTGAAGCAAGGAACGGAACTTCCATCCTTGCCACTGATACGGCCGGGGAAATGCTATTCCGCTATGTGTGGTTACTCGTTATTGGGTATTGCAATGAGGTACAGAAAAAGGAAGCTATCTTGAGTAACCCTTTAGAGAGTGAAGAGAGGAGTTGCTATGAGTCACAGAAAGAGTAGATCCAATGGACCAACGAAATAGGGCCGGGATCCGAGCATCTTCTTGAACCACCAATCATAGACGGATGGAATACGAGTCGTAGACCTTCAGGCACCACGAGAGCAGCCCTAGTAGGAGTTTCAAGTCTGAGGTCAACAGCTGGTGTAAATCGGGCTCTCACCTCTCCTTTTAGGAGGTAAGCGAACAAAGACAAAAACATCATTACAATAAGAAGTTCCGTTTTCAAGAGCCGGAAATGCCTGACTTATCAAGCGAAAGCGGTGCTTGGTCACGACCAGCTAAAGCTAAAGTTCCATTAAAGAGTGTTTCCACGTGGTAGTTGCGTCAAGCAGCTAACCTGATCTAAGGAAAGAAGCAGCATAAGCGGGAGACATGGTCCAACCTAGTTCGAGCTTGCTTCTATTAGTCCCACCCATGCATATTCTCCAGAACCAGGGGCTTTAGCCTATTGAAAAGCAGGCCTATTGAACCTTGCTCGGAAGTCAACAACCCTTCCCCTGTCACTAGCCTAAACCTATGAAAAAGTACGCCCTGCCAGTACCCATTGAAAGTTCAGAAGTTCAGCTCTTTGTCAATACGAACCTACCCTGCCTTGCCTTCTTGTCAACTCCTGACATCCGCTCTTAAGTCTTCAATTGCTTACTCTTTTCCATACGATAAAGAAAGGTACCTCCTAACTCATTGATTAGCCCTGAGACCCGTATTCCTTTGATTCTTCTCCCGGAAAGAAGTCCCAACGGATTCTTCCTTCACCCCGGAAAGGATTCGTGAACAACTGCAGCGGAGATGAAAAAAGACTAGATAATGGGAATTGTCTTTCCTAGGCTCAGGAGCGTAGGATGAGGTCTTTGCAGCTTGAATTGAAAGCTTTCCGCTTCGTACCTTTGGTGCGTAATATAATATATAGTAATAGTAGAAGATCCGGGCGAAAGGAAGTGATCTTTCTCTAGTCATATGCCATTCAAATGGCTATTCTTTCAGCGAGTGTGGGCTCTCCGCTGCAGGCCAATGGGCCTGAAACAAATCATTAGACAATGACCCAGCCAACAAAGAAAAGCTTGGGCTAACCCTCTAAAAGCGAATGGTAATAGTCTTCCAGGTCTTGGCCTTGGTTGCACTTCTTCCTCCCTTTACACTTTCCATAATATTCAAGTCTGAAGTCTACCTTTGTAGAAGATCGAAAATAAGTCTTCCCTATGACAGCAGTCAAGTGTTCCCTTCCTTCCTTTGATGAAGCATAGGTAAGAATTCTATATGGGAAAAAATACGTCTTTTTCAACTCGTACTACTATTCCGACCGGGGAGCTTAGTCCATTGATTCAAGATACGAAGCTACGGGAACACTTGACTCTCTTTTTCTAATACCCAGTCCCCAACTCCAGAAAGGAAAGAAGAAGTCGATGTCTTCGGGTATGGGCAAAGGCTTCCTCGTAATCTATCCCATATGTTTGAGTAAAGCCTTTAGCTACTAGCCTAACCTTTTCCTCCTTTCTCCCATAAAGCTTCCCTTCACCCAATCCCATATCCAATTAGTAGAGATCGAGATGGGGAGACTCCAAATAATACACATGCGCATTTCCTCTCTGAATCGAGAGGTATCAGATCGAAGTAAGATAAGTCATGTATTCTAATTGAAGTGATTTCCGGAGAACCAAGTGGATTTATGAGCGCATGAGAGGACCAATGAGACAGAACACTGTTGGATGCATTCCTTCGCTAGCAGGGCTTCGGCCCATCTCAATTGAAGAGTCTTCGGTCAGTAATCCCGTACTCGACCGGCTCGAACCACTACGTTATACATGTTCCGGCTCATTCGTATGCTCATCCTATTCATGTCACCAGAACGTATAATAAACCATCCTGTTACAGGGAATCTTTTGATATCAGTTCGAAGGAGCGCCGAACACCGACTACAGAAAGCAAGGAATAAACAGACACGGGGTACGTTTGGCTTTTTTCATTTCCGGAGTTTTAGCCCTGATTAACAAAGGACCAGAGAAACTTTCTAGCTATGAATCGGGTAAAAACCAATGGGCGATCTCGAGGAAGATTCTGTGGGGTGCTTTCTATATCAATCCATTTGCTTGATTTAGGTGAGCTAACTTTCGGGAAAATTGATCCGGGGTTGGGAGACGCTCAGGTAGCATTAAGTGAGAAGGCACTGAAGAAAATAAGATATATGATACCTCCACCCATACATTTCAAAAAAAAGAGAAGATCAAAGAAATTGAACCTAGAGAGAGAAGAAAAGCTTAGCGAGGAGATCAGGAATGAGGCGGAGATCGACGGCATTCCAAAGGTAAGACGACGACGAGATTCCTCTGAGAATCGGGTACAAGAACGTATTGTCGCCGCAGTTAACGGAAGAGTCTTCGAGGACGAAGAGAGCAGAGATTCTAAAGAGCGCAAAGAGAGAGCTTTCGCAAAGGTTAGCGAATAATCCAATTTCCTCCTCCTTCCGACAACGCATTCAATAGCATAAGGCTTCGCTACCTGACTTATCAGCCAGCAAGTAAACTACTGCAAGATGCTATACTTCTTCGGTCGAGCCTTGAATCTGTTGTGTCCTGCTCTTGACTTCCTGAGGAAGCATCCGGAGGTTAGTTCCCTTGTGAGTGAGCTCCCGTGCTCCTCTTTTGGGATAGGCCATTCGGCTCACTCTCATGGATAGACCTTCGGTCTACTCACTTTGCTTAAGACAGGGCAGCAGGACTTTTTGATCCCTCACCGACCTTACGACCTGCCTCATTTCGGCATAGCTGTAGACAAACTGAACATGTTGTAAGGGCTTTTACGTTAAACAAAAGGCCTTTAGCGTGCAAAGCAAGCAGCCAATGCGAGAAAGGGGCAAGAGCAGTACTCGGGGAGGGAGCTGCGCATCCTCAGGATGGGTCCCTGCCCCTGTAGAGTAGTCCCTTTAACTCAGTTAACTAGAAAGCATCTAATTCAGAATATGACTAATCAATACTAAAGCTCGCGAGCCTAACTTAGGATGGACTGGCAAGAGAAAAGAACTGGCATAGACCGCTCGTATAGAAGTTCATCCCTCAGCAGTCAATCTCATAGTAAGATAGATGAATATATAGACTGGGCTGGCTTGATATGGAGATCTGATAGTGACTATCTCGCTTGCCCACTGGCTCACTGTAATAAGGACTAAACAAGGGATTAGACAAGCGATATGAATGGAAGGACAAGCAAGGACACTATATTAACTGGGCTATACAGTAAGAATTTATCCTAGAATTAGTAGTTGAAGAGGTAATAGCCGATACGAGCAACTAGGTCTCATAGGCGGGAAGCTTTAAGTTGGCCGCTTCCTTAGCCATTCCAGACAGCAACAACTTTGAGGTAGCATCAGCAGACGAGAAGGAGGAGGTCTGAGAAGAATCTTGAGTCTTGAGCTTCTTATTGGCCGCATCCAAGTCATTAATGAGAGTTCTCTTCCCAAGCACAGATCCTGGAGCCAAGTTAAGGTCAAGGGCTGCTTTGTTGACTAAGCTATGTCCATCATTTACTGTCTCAGCCGGCACAGAAGGGGTTGGATCACCAACAAAGAAGCAAAACCACCTGGTAGCCATACCAGAGATATTGGGGTAAAAGCTAGAGTACCACCCTCCAGAGGAGAGTAATCTAAAGAGTTACAACAGGCAATAACAGCTTCGAACTCATGCAATGCAACAGGGAGCTACTGCCACTCCCATGGTGCATTGTGATTACATTCTACAACAATCCCAGTTATACTTAACACAATTAACATTCCACCACTTAATAAGAGCATTTGGCGGAGAAGGGGTATGCTTGGCCTTGACCCAATTCATTTTGTGCAGAGGAGGAACTGGGTTGTCCAGGATGAAGCGCTCTCTTCGATCATCATCAAGAATTGGTTCTTTGCCGAGAATCTTCCTGCACGGATTCATAAAAGCTTCAACTTCTTTATTAAACATGTCCATCTGCATCTAATCTTCTCTGCTTGCATGGCCGCTCCATGAAGCTTGAAACAGGTTGAGGGATTCTCTAATGCTCCAGCGAGTATGATTCCTGACTGTTGGCTGTGATCGAAACATCTATATTACCACGATGCCTTTTCAAGGAGGACTTGCTTCCACTTTGACAAGGAGAGTCACCGAAAATGCTTTTAGGAGATCACTTGATGAGCTTTTACCTGCATGACACTGTTCACGAGAAGAGTACATGGCAGCAGAAGAATTCACAACATTCAAATTACAATGAACAGAAAGATCATGGGGAGGGGCAACCCTTTCTGGGCTACAAGGGATAACACAGAAAGAAAATCGGGATGCAAGAGCTCGTCAATCATTCTTTCAATCTCCCTCCTATTGTTGGGAGGGAGCTGTGAAGACTCCGGTCCAGTATTAACATGCTTATCATGCTGCACCCAAAAGCCATCAGCAGAACCATCAGGAAGGTCCGAATCCGAGGAAGAGTCATCGTCAGAAAGCGAGATGAAATTGTTAGCACCATCATATCTCCCCACAAGGATATCTTCAGGCTTTGTCTCGAAGACTTTGTGGATCTGCCTGTCTCTGAAAAACCTCACCTTTGTAGTACCCTCGAGCGATTTTCTCTGATCCATCTTTGCCACTCAAGATCAGCTACACTTGCTTCAAAGTTACACGTATACCTGGTACCAAACCTGCTAAGAATGCGTTAATTGACAGCTTCATACCCTCGAAAAGCATCAATAACACTCCTAGGGCATTCATAGATGGTATGCCCCAGAATGCGACAGAGGTTGCACCTTCTGAAGACTCGTTCATATCTGCACTCCACCCATACTTCTTTTCGTCATCTAGTTGAACCTTAATCTTCTGAATGAAAGGTTCGGAGACGCTCGAGCGATAGCGAGAGGAAGCATGGGTTGATGTGTACTCTGACCCTGAGGAAGTTGAGGCTGCTGCTGAAGATTGCACCTCCTTCATTCGTGACAGTATTCCCCACCAGGTTCCCCAGAACAGTAGCAACATATTCATTGTGATATTCCAGGGAAGACCCCAATATTGCACCCATAAGGGGAACTCTCGAGCTGAATCTCTCTGACAATGGTGTTGGGGAGCCAACGATCCACCACAAGTAAGGAGTTTACAATGGACCAAGGTCCATTATTGACAAAGTACTCCCGATCCTCCTCGTTCTCAAATCTAATAAGAAAGAAGCATTGTCTAGGACAGAAAAATCCGCTCGCGGACTCCATGCAGATCGAAGCTTCCTGGACAGTTTTTGATGGAGAAATCATGGACATCAACAAGAACAGCAATGACGCAAGTGGAGAGATTCCTCCTAGGCTGATCCAAAGATGCAGGATCAAACCTAAGTATAGGGCGGAAAAGAGATTGCTAGCTTGAGCACTGAGCACAGAGAATCTTTTCTTCTTCACTCTCCTCCGGTGAAATAAGATAGAATATTCAAACTATCTAGTTGAAATCTGAATGGAAATTTAGTTGAAGTCTCTGGGAAGAGTCCGTTACTTAGAAAAGTGAGCCACCGGTACAAGAGACTAGAATTACTGATGTTTCAGTTCAGTGACTGAAGACACGAAGGAGAAGAAGAAGACTTGAAGGAGACCCACTTCGACCTAATTTAAGAGAAAAGAAAGCAAGCTCAGTTCAGAATGGCTACTTACCAACAGAGGAAAGCGAGATACTACAAGGCCCGAGTGAGATTTGGAAATGGATCGAGCTCAGCCAGAGCAGCTCCTACTGACTATGACTGGACAGCAGTGGACTCTTTCTCAAAGTCTGACCCTGCCACCTATATTTATTTGATTGATTAGCCCCATTTAACAGGTTTGTAGCGAAAGGGAAGAGGCTTCATTCCCGAGCGGACGAGAAGAAGAGCAACCCCATGTTGGGAAGAGAATAAGGGTATTTGACTAGACTAGCTACCGAGCTGACTCAAGGAAGCCTGAAAGAAAGGGCTGAAAGACAGCTTCGGCAACAGAAAGTCGAGCTGAGGAATACGGAAGAGAAGCGGATTCCACTCAATCTTAGCCAAAGAGCAGCCCAGAGATGAGAGGAAGAGGCTAATCGTAAAATAGCATATATAAGAGATTTTCCCGGGCTAGGAAGTGAGATTAGTCTCAGAACTGCTTGCCAGTCAGATTAGGCTGCGCTTTCCCATTCTTGGGCTGAACATTCGGGATTGGACTCTTTCTTGCTTTCGTGACTCGGGTAGGAACTCTTACCCATCGCTTTCTGCTCCCGTACGCATTTGTCACTCTTGGTCTTTTGCGCCTGACTCCCGATCCGATTTTGGGGTCTACCTATAAGACTTCTTAGTTCCGTCTCAACACGCTCCGTCACTTAGAAGAAGTGCGGAACTCGGATCCCTATGGGGAGTTCCCGTTACGCTCCGTCATCTACTGCTATTGGTTGACTTTTCTCCTTCCCCTAAGCAAACTGGGCACCCTCTTGGTAAGGTCGGTCTCTTACTTAGTGCTTGCGCTAAGGTTAGAATATCTTAGTGTGAAACTAAGGATAAGGAAGGTTGAAACCGCTAAGGTACCTCGCTCATTAGTCACTTCCCTACATGGTTAACTCTTGCCGGGTTAATGTATTTGAATGAATCATATATTGATTATTACGCGTAGAGATACCTATTTCTAGTGGTTTGTTCCTGCATCAATAGTAGCTTGAAGCTCTAGTTGTTGAGAGTGACAAGATCATCAAAAGGAGGCACTCAAGCTTGACTATACGATTGGTAGGAAAGATTTTAGACTTGAGCATATGCCTTCTTCCTCTATGGATAGGGCGACGTGACACGCAATGGATCGGTTAGAAAGGAGAAGAAATTGATCACATGTCCCCTTCTCAACAACCTTTCCCGCATAAACATAAATAAGTAGATAGAGTCGATTTCCTATCCTTGGTCAACCTAAGGCTTTTCTTCTCTCTATATTTAGTTACCGACCCGAGCCTATGCGAATAGAGCCTACACTGGCTCTAGGCCAAAGCAATGAAAAGGCGACGGTCTATATGGAAATAGAATAGGAAGGTCCATGAGAGAAAGCCTAGCCCTATCTTAACCTTATGATCCTAGTAGGCTCGAGTCAGGCGGAGGCAGTTGGATAGGCACCCCCAGCAGTTGCTTCAGCTAACAAATTGGAATAGGTACAACTGGTAAAGCAACAGCTGCTCCTCCTACTAAATCGACTTGAGCAACGGATGCTGGCTCTAGGAACGGAGGAAAACCGCAATAGTTCAAATAAATCGGTAGATGAAGAATTCATTGTCAAACCATAGCCTCTGGATTTAGATGGACGTTGCCTGCCTTTCTTTCAAGATAAAGAAAGTCAAACTACTACTCCTATGATTTCATTACAGGGAAAGGAAGTTAAACCTCTCAAACTCGGAAAAGAAAAATCAAATGTTTCTTGACCAGGAGCAATAGACCTCGAAAGGTGTGAAGTTAGTGCCTCCGCTATATAAGCAATTAGCTCGCCTCGTCGAAATACATTTTTGGACGTATTCTCTGTCATATTCCAGCGGAGTCAAGGCCCATTTGCTAATCGGCTTGTCAAGACTGCTTTGGTCATCAAATCTTTGTTTGATAGGATCGACTCTGGATATCAATTTGATTTGGTGTTCAAGTAGGTAGTGCCAACACTTTTCCACTTCGAAAGCTTCACCATACAGGAATGCCACATTCTCGCTTTCGAGGACTTCCAACAACACTGGCTGAGGTTTGAGCTCCAACCTTATCTGCCTATAACTCTCTCACTTTAGAGCCCGCTCTATCTTCACTTCGAGCCAATCCTACCTAACATTTGAACCTTTCTCCTACTTTTTCTGACCCAATCTTTTCTTTTCCTCCCTTTACGTCTCTAACCTAAAGTACTCCTTGTCAGCTCTATCTGGTTTGATTACCGGATACGGAAGTTCACTAGTAAATGTCCCTTCACTTTTGAGCTATGGAAAGATGAAAATGTTGAATGAATTTCAAAGTTGTTACCGGACTTCGCTTCGATGTGTCCATCCAGTAAGAAAACGGAACTAAAATGAATTCAGAATTCCGCCCTTATCGATTCTCGATTCTAAGGCCCACCGCCTTCTTACTATAAGGTCACTTCACTCAGGCAGGCCGGGTTTAATAGAACGGATATGTCCTGATCTTCTTCTTGCTTACTGAGCTATGGGGCTCCTGTCCTTCCTTGTGCGGTCACGTTCCCGATCTGATTCTATGGGAGAAATAACGTAGGTGGAGCAGCGGAAATCTCTCAGAGGGCACCGTGCTGCTTTCCGGTGCTTATGCTAAATTATGAAAC

>Decalepis_hamiltonii_chloroplast_contig14

TTGGATTTGTTCGTACCATTCTGTCATCGATCACTGCTGGGTCGGTTGGTTTTTTCACCCAAAAGCATCGAGAAAGGCCCATCATACTAGAGTATATGATATATATGAAATGATCCGCGGTCTCATTTATGCTATGCCCTGCATCGTGTTCGTGTGTTTATTGAGCTTTCTTCACTTCAGTGCCATGCGTTTTGCTTCGCTTTATAGAAGAGAAAGACCGTTGTCCCTGCTTTCCTTCTTTCTTTCAGGCCTGGCTTGCTTGGCCGGATTGCTTTGATCTCTTGAAAGGTTTGCTCCTTGGCTTGATTGCTTACTCTTTGCTTTCCTCATATAGATATAGGCTGTTCCCACCAAAGTCTGAAACTGCTTGGCTTGCTTAGGAATATCAATCCCTAGGTCGTTTACTGCTCCGAGGTATGAGGGCTCGGATCTTGCCCTAGTCGTATATATTGAAATTGTTGGCTTGGTGACCGGGAACTGGCTGGCATACTACAAGGAAGCAGGTTCTTTAGGGCTTTTACTGAAAGGCTAGCTTTCCTACTCTTATATTATAGTAAGAAGCAGAGGCGAGCAACTTCTTCTTTATATGGAAAAAGCGTAGGAAGTACCGAAGGAGTTGCATAGAAAGTACTAGGGTTCGAGACTCCTTTACTTTCACTTTTATCTGTGAACCAATACCTCAAGCAAACTAGAAAGATCGTAATATAAGACAGACTGTTGAAGAATCACAAGAATCAATAGGCAGGAAAGCCCTTTCTTCTGAAAGTACTTCCGATTTGGCGTTGTTCAAGCCTTCTTTTCGGTTGGAGTTCTAAAAGAAGTGGGTACAGAAGGGCTTGAAGCAAAGGCAGATTAGAGTCAGAAAGGACTAGCTAGAGTTAGGATTGAATGCAGCTATGCCTATAGGCTCTCAATCAGATTAGGGCATCTCCTTCCTCATCATAGCTTGGAGGGTTAGCTAGGCTCATTCATTAAAGGAAGGTGGGTTGCGTTCCTTTTGAAAAGAAGGGACTTCCTTCCTAGGACCAGCTCTTACTATAGCAAAGGATTATAGGCGTGAACTTCCTTTACAGCCTTTTCCTCAAGACTGACCTTTAGAACAAGAGATCCTGCCCTCTAAGCTAGTGAATATGAGACTAGTCCCTATTCTAACTGGCATGGTTTGTTGGAATACAGTTTTCTCGTGGAAAGAAGGACAGATTGCAGGGCTTCTTTAACCAATGTGATATAGTTTGTGTTAAAAGCCCTATTCTGGACGAATGAGCTAGGTTAGTCGCATATCGGTTGTTTAACCAATGGGATTGAAACTGGTTGGCTTGGGGCTCCCTCGAAGTAGGAAGTCGTCTCAAAGGACAGGCTTGATCCTAGCTTGACTGCTACCCTTGGCCTTAGACCGTTAGGAAAGGAATTCTAGAATAGCTCGACTGAACCACAGTAGTCAAGTAGTCACCCTACTTCTAAAAGAACAGCAATGTCACAGGCGGCTATGCGACAAGTAGCTCTCAGAGGTTCCGTCCACTCATTCCTTAATAAAGGTCTTTCCTACTTATGTTCCAGCAATCGATGAACGTTCCATCCGGGCCCGGGCCTTCTCAATCAATATTCCCTCCTCCTGACCCAAACCGGTAGTCCCACCCACGGTGGTAAAGAGAGAAGGAATTCACCCGGAGGCGGCGACGGTTAGGTGTTGATGCCTCGTTCATTTGCCCGTCCTTGTCTCAGCCTTTCTATTCAAAGTTCCTCGAATCGATTACTCCGTAATTCCTATATCCGTCTAGTCTAGCAGCCCTTTTTCGGATAATCCAGCGGCAAACTACTAAACCCCATGAGCAGTACAAACTACGCGAAGGCATTAGGGACTTGTTCGTCGCATTCAAGGAGGAATATAGCTTAGCTATGTTCCCCAAAGATACCTACGGGATGGTCCCCATAACCAACCCCTTCTATTCTGAGCGAGCCAAGACCTCTTCGTGTATTCGACCCATAAGAGAACTATCTTAGCACTCCCGCTATTCGTACCAAACGAAAGCGCCTGACTTGCTGTGTAATTTCATAAAGAAAGAAAAGTGTGTGAACCTCAGCGAGTCTGGGTTTCCAACTAGCCTCCTGGACTGAACCGACCCCAACATATGAATTTTGAACTATAAAGAAAGTAGGAATGGAATATAAAGATATGCACTTTATGGACAGAGTTTTTCGAGAAAAGCATATTCCACTCTGAATAGAATCCCGAATAGAAGTACTATTGTTGGTCCTAGATATAAAACACTTCCGGCTGTAGTTGCCCCTGGAAGTTCCGGATCCCGATCCCGGCTACTCTGGCTAGAAGACCTGCGAGAAAATAGTCATACGTAGACATTTAGTACAACTCACCTTCTCCGGTTAGTGAAGGAACACCTCGAAAAAGAGAATGAATGCCAAATTTGGTTTGGTTGCGTAGCTCGTGTCACCTCCGTAGTCCCCTGAGACAGGCCAGCTACCAATTCTCCACTTGACTAAATTATATGCGTGAAGAGGGTTTCCACTGGACACTAAGCCCGAGTGATCCCCCAGTTCCCAGGCCTAACTTCACCTCACCTGCTTTTAGTTTTGTATTAATCACTTCTAAAAAAAAAGATCTTTCTTCCTCTTTCGAACATAGTCAGTATATCTAGTTCTCATGTATCCTAGGGTAAGCTGTGTACTAAGACACCTATTTTCTTTGCTTAACACAATCCCCTAAATTTCCAGCTATATATCAGGTTCGAACAGGTCGTCTTCAGGGCAAGTCCTGGCTCGGCCGTAAGGTTAACCAAGACAGACAGCCAAGCCGAAGAGGCAGTTAGATTAGGAAGCCTGAATACGGAAGCTGACTTAGTCTCAATCATTTTTTTTGTTTGATCAAAACAAGGAAAAAAGCCTATGTGGTGGGTTCGACTCCCACAGGAGCGCACATTAATTACCAATTAGCTGCTTTTCGTTCAAGAAAGATTGAATGAATCCATTATGGAATTTAATAGTAAATTAATCGGCTATATGGCATCTATTTTCAAGTGCGAGAGGGGTCCCATTTTGTCTTCTTTGAAAAATGGGAACGGAGGATCAAATCATAATCATTGAAAATGGCATTTCTGCGGCCTGTAACACATCAAGCCGTGAAGGCCCCTACTAACAATTTACTGATTTACAAACCTACCGTAGGCCCTATGGGGAAGTTAAATAGCAAAAAGCTATTTTGAATTTCTAAGATCTCCGGGCCTAGAACCAGAAGAGAGCGCTAACAATCTGTTCAGTTGATCTCAAAGAGCCAACCGAACCGAGGTCGGTGGAAAAAGATTAGGTAAGCATTCAAACCAGCATTAGCCGAACTAACATATCTAGTTTATCCTGAAGCAGCATTTCTCGAAATAGCATTCCCATAAGAAAGCAGAGGATGAAACCCTTGCTTGTTTATCCCGACGAGGGTTTTGAATCACTCAAACGAGCCTTTCTAGCCACATCTGAATCGGCATCCCTATCCATATCTTTATGTGCAGGGGCACCCAGATCCGAATCTGTAGAATCAATTTGAGAATCCCCATCCGCACCCGAATTGGCTAAATCTAGTATAGTAAGTGTTGTTCCGATGCGGGAATAATTTGATTTTAAAGAGATAAGGCTGCAAGAAGAACCTATAGAGGAGGCTTCACCGAAAAAGAAGAAAGAAAACTTGAAATGTTTTGGGAGGGAAGAGGAAAATTCAGAAAAGAGAAAAAAGAAGAAGATTGAATGAATTATCCCGAACTTGCCTCCGCCGCCGTCGCACGAACCATTTATGATGGCCGCGTCTAGGTGGATTGTGGTGCTACCATTTCTTTAGGATACCTTTCGGCTTCAGTCAAAACTCTTCCCCTTTGTTTTTATAGAAATGGAGTTTGTACGGGAACTCCACTTTTAGTATGGAATTTCCTTTTGGTTTGAGTGGAGTTATTGTAGAAAAATCGTTAAAGGAAGGACAATCGATCGCACTTGGCGCAGAACCACCTAACGGTGGCTCTTTACTCCCTCAAGACTTGCTTCTTATTTTCTTTCATCTTCATCCCCTTTTATATCAATAGGGAGCTACAAGTAAGGCAGCTCTGCTCCAGAAAGAAAAGAATGGAACTAAAGGAATGAGTTTATTTGGTGTCTTACTCATCTCTTAATAGAAAGAGTAGAGAAAGAAGTGGAACGAGGATGAAGAATATGATAATAGATTTGGTTATTCCAGTTCCGCTGCTTCGAAAAGCCTTCTATCAAGCTTTGTCCAGAGTCCCGTCTAGGTTGACTCTTCGCTAGGTGCGGAGCGAAAGCCCTTCAAGCAGCCACCAAGAAGAATGGAACCATTGGTCTAAGGATCTTCACTATGGGGCTTTTCTCTTATGAGATCCGGGGTTGGTTGGGAATTCCCCGATCCTCTTGAGTATTGTTGTTTAGTTTACCAACCCGGGAAGTACCCGTTGTCCCCTTGCTTTGCCCTTCCTCTAAGGGGTTGTTGTGTAACAAGCGCATAATGTCCACTGGAACGTCCGTGGATTTTATCATCAACTTGATGAATTCATTTGTTGATATAAGACTTTCCTATTAGAACGGGTTGTTCAAAGGATTCCCCCTTCCATCAAATATTCGGCTTTTTCCTGGATACTCGGGTTCAAATACCCACGGATTCGCGGTTTGCTTACTAGCTTGATATAATTCAGAAAACACTAGTTTTCTCGAAGCTTCTTGTTCATATCTCTCATCAAAAGGCGCTATTCGATAATGTCTGTCTAGCAGACTCCCCGCTAACCCAAGTGAACATTCAAATATCTGTCCTACATTCATTCGTGAAGGTACTCCTAGCGGGTTGAAGACCATATCAACAGGTCTTCCGTCTTGCAAATAAGGCATATCTTGTCTAGGCAAAATTTTGGAAATGATACCTTTATTTCCGTGTCTTCCGGCTACTTTATCGCCTACTTTGATTTCGCGTTTCTGTGAAATATATACACGAATCGTTTCTGGATTCTAACTAGAACCCCCCTTTCTGGATCCACCTCACATCAATAACCCGACCCCTACCGCCTATAGGTAGTTTTAGACAAGTTTCTTTTGAAGTAGATACCTGAATGCCAAGTATGGCTCGTAACAATCTATCTTCCGGGGCATATGATGATTCTTTTACCATCTGGGGTGTTAATTTACCTACTAAAATCTCGCCTGTCTCTACCCAAGATCCCAGCACCTTTTGTCTAAATTGCGGAGTAAATGGGCTTCTAAATGCGGTATTTCGTTAGATCTTCAATCCTTGGCTTGTCACATGACTTCGACTTTTTCTAGGAACAACAACCTTAACGGCCTGCTAACAGCACCATAAAGATGCGGCTGATGTACGTATGTAGCCCTTGCGTCGATCTTACCTCCGCTGCCTGCCCGTACGCGGGTCGACTCACAAGAAATGCAGCTAGCTCGCCAACTAACTGTTCATGTTTGAGTTTGTTGGCTTGCTCTTCTTCATTGTTATTTAGACACTACCTGAATTTACTTGGTAGCCTACGTGAGTATCCGGATGGATAGAGACCGATCCCTTTCTACATACATAGCCCCACCCCCAGATACATACATACTTTTCTTCCTTTCTCCCAAATGAATTCCTGAATCTTAGCTCTTCTCTTTCTTATGTATTCTTACTACTATTACTTTCTAAATCGGGCCGGCTTCTTTTAACCGTAACGTATTGGGGTCGTACGCCTGGAACAAGAGGGGTCGTCGTACAATTTCGGCGATTACAAAGGAGTATATCCCTCTCCCTTAGTGTCTTTCCACTTCCGTCGTTCAGGAACAAACACTTCGCCTAAGTCTTTTGAATCCCGGCCCGGCTTTTCCCCACTAATTAATTAAGTTTACGACCACTGAACAAACTTGGTTGACGAAGATGGTTTATGCGCCGCTAATGTAGCGGCTTGTCGAGCATTTGACAAACTCACACCATCCATTTCAAATAGGAATTGTCCCGTGGACACACGAGCAACCCAACCCGTAGGATTTCCTTTTCCTCTTCCCATTCTGACTTCTGTAGGTTTCCCAGTAATAGGGTAATCCGCGAGAACTCTTACCCATATCTTACCATTTCTTCGGAATTGTCCGCTCATAGCACGATGGAAGTGTCCGATTATAGCCCGACGCGCTGCTTCAATGGCTCGATATGAAAGACGACCAGCTCTACAACTTTTAGTACCATATCTTCCAAAACCAAGTTGTGTACCGTCCGGTTTGCAACGCCTACTACATCTGCCTTTACGATATTTACTATATTTCGTACGTTTCGGATATAGCACGTCCCTTTTTTTTTACTATATGAAATCCACACTTTGACACCTGAGATTCCGTAACGAGTAGATACTTCCGCAGGAGCATAATCGATTTTCTGGTTAAATACATTACGAGATGTTTTTCCATACTTTCCGCATTCAGTTCTAGCTATTTCTGCACCTTCTAATCGACCTGAACAACATATACGGACCCCTCCACCCCTTTGTCATTACTAATGGAATATCCTTCACTATTTTACTAAAAATGGAACGAAATGATCTTGTTTTGTTCCTCGGTTGAAAAGAGATGTCTTGAGCAATCGGAGAAGCACTTTGATAAACAGATTTTATCTTGACCGATTCAATTAAGGTATTAGTGTTTGTTCTATTAGACAACAAAGATCGCATTTTTCACTTCGTTCAAATAAGAGTTGTACCCGTACGGAATTCTTCTCTTTCTCAGTATGATCTCTATCATCATCTCTATTCCCTTTATCAATTCTCCTATCCTACCTAGGTCTATGAATTTCTCTATCAATTCCATTACCTTAGCCTTACTCATGAGGTTCCAACATTTTATCCTTGATTTACCTAGGAGTTGCTCCGGGCATCCTCAAAAAAAGGTTATTATACACCCCAACCCCATCCCTTGGAAAAAAAAGGTAGCACCGAAGAAAGGAAAGAGCGAGGTGGTGGTTTTGTTGTTCCCGCGAAGCGAAGATCATTTGCTTGGCGAATGAAGTGGGTCAACCTCTTTTGGCTTCGGCCAAAGACTTTTCTCGCTGGTCGAGCTTTCTACAAAAAAGCGATGCGAAAACGTATTCTCTTATCTAAGCTTCTTTCCTGTGCATTAGCTCTCCCCATCGTAGATGGTTCAGCTACGCCCGGTGCCACGAAATGATTGAGAACTACGACGGGGTCGAAATTTCTTTTGTTCTTTGTATTCAATAAGTATTGCATGACCAAATAATTCAAGGAAGGGCGCACTGCAGGGAGGGTCTTTTAAGTAGATGACTCGTCGGGCCGTTGGGCAGGACTTCTTTGGGAAAAAGAACTTGAATAATTTCGTTTTTCTGAAGGAGTCATCATTTTCTATCAGGAACGCTATGTCATTTACAACCCCGGCGTATTTCGGATGCTTGAAGGCCCCGCTGACCCGAAGTGATTTAGAAATATTCTTCTTTATCGATGGTGATCGGTCATGGTATGCATAGCGTTGCTTCTTTTTCGGCCAAATCCTGATTTCGTTTTGCTTCTCCCGATCGTCGAGCCTGATCGAATCGACTCTTTTCCCTGCCCCCGGCCTCTCACTTCCTTTCGTTCTTCTTCTGTACCGTCGTTTGAATGAAGACACCGATCGGCCCGACTTTCCCAAATGCCCACCACCGGGCCTTCCCTTTCCGGGTCTGGATTTATCGCGTCGTTTCAGTCGTCGTGGTCGACGGGGAAGAAAGAAATGAATGAATGTTCTTTTGGGAAAATGTAGAATAATACACCTACCGAGACGAAAGCCAAAGGTGAGTCTCGTAGGTGGACGTATCGAACCGAAATAAGATCTCAGATTGACATCTTGATACACTGATTTACCATAATAATAAATAGAGTCAATGGTGACTCCGCCGTGGGAAGAGGCTTCCTTCTTGCGGGAGGCTTCCATTCACCAGCGCAGACTCAAAGTGCTCTCGAACCGTGCTGGATAGTCGCCCATCACACGGCTCTCAAACCCAACCTGTGGGGATCCCGGGAGACAAAGTCAAAGCGCTTGATCCTTTGCCCCATACTTTGAGATGCTCCTCCTCGCCGAGCAAGGCCATGTTGGTCGTTCTAGGCTTTTCTTTAAGCCGCTATCGTTTGGTTCGGATAAGTCAAGTCCCTTCGCTCAGGTGGTCTTCCCTTATCTTCCCTAACGTTCAGAGTTGTTCTGATTTGAAATGAGAAAGGAGCACCGGCCGAGCGCCATGAATGAATAAGAAATTTACAGCAGAGGGTTCCAGTTTCCTCTTTTCGACTGAGTTCTAGCTAGTAACATGTCGATTACAGGAGGTTGGTAAGAACTGAGGACAATGCCCCCTAACTTAAGTGGGCCCAGCTCCGCAACTGGTACTTTCGGGGGCGGTTTGGTTTCGTGCAACGCCCTCGCAGCAGGAAGAGGCAGTTGTCATTTGAAAGGAAAGGCCTTTTGTATAGGGCGGCGTTAAGCACCTGCCCACCCTGATGAAAAAATATATCTTATTGCCCTGCAATCAAAAAGAGGGCTAACGCGCATCCCTTTTCTTGCTTTAGTTTGATTGCAGCTAGCGCGCTTGACTAATATAAATAATAGAAAGGGTAAAGGTTCTTCTCCTGTTTTACGAATCTACAAAACTCTTTACTGAGCGAGACTCCATCCATATCCCTACTAGTGGTTATTCTTTTTTTCCATTCTATCATTTGTTTGACAGCTTAAACTAGGCTCCACTTTAGATTTAGATAGGGTTTTGGTCTTAATCAAAATAGGTCAAGGGCGCGTCGGAACGATCGGTAGCTGCTTAGTCTCATCCGAGTATAATCTCCTTTACTGCTTTTTTTCTTTGAAAAAAAAGAAATCTAAATAAAGAAGGGGGTCGATTTAGGCTCCTTCCTTCCACTGCATAGCTGTGCTAGCAGGTACTACGAGCCCTCTGTCCCACGCATCTAACCAGCTCGCGTGGTTCACCGGTTCCACCGAAAACTCTCATTTATTGAAGCGGAGCATAGTGCGCTTTAGGCGCCGAGCGAGAAACGTCTCTTCTTTCGTTTCGGTTTATTCACTGATCTGAAACTTAGCACTTCTTTTCTTATTGATTCCAGGGGCGGCGGCATTATTGAATGAAGTCTATCCGAACCGAATTAGCACTTCTCAGATCAGGCTAGGCCTCTCCAGCTGAGCTGGGCGCCGGGCCCCTGAATGCGCTAGCGATTGACACATAGGGAGTGGTAGCCCGGGTTTCTCCGCCTGGTATCCTGCACCTCGCGTTCATGATATCTACATTCAACTGTGCCCCAGAGACACGGTCGAAGCACGCCCACTGAGTGGGCTTCTTTCACGACATGCTCTAGTCCTGGGTGTCTTCCAGTGGTCTTCTAGCCTTAGAAGAAGTCGTGTCCGCCCCGGACATCTGCAACGTCCTCCCACACCTTTTTATATTTAATGGGACAAACTGAAGGAAAAGACTGACCCATTCGGTGACTTTCGCGGTCGCCCTCACTGAACCAACTTGAATCTGAACTACGATTCAGATCAAGTCTTACCGAAATCGGATTTCCTTTTCGCGCCATATGTGCTTAGACTTGACTTTTTCCCCTTTTTCTTCCCGGTCCAATATTTGTTCTCGAAGGTCTTCGTTTCCGTGTAGAAGCAAACTCTCCAAATTTATGACCAACCTTTCCTTCAATAATCTTACAACGAACAGAAGTTTTTCCATTGTAAATTCGTACGGAGCAATCAACGAATTCCGGCGAAATAGAAGATCTACGTGACCAAATTTTCCTGTTCAAAAGAAGATCTCTCTTCTTCATTCTCAAGAGGAATGCATCAACAAAACTGCCCTTCCATATAGATCGTCGTGGCATGAACTCAGAATTTCTTACCTTCGATTCCGCCCTGAAGGCTAGCTCCTACTCCTACTCCTTCGACTCCTGAATCCTCGTTGGTCCTTTTGACATAACACTCGCGGCTGCCTCCGGTCCGGTAGGTCGGTCGCCCTCTAGCCAGTGACTAGCTCCGCTATGGAGCTACGTGTACACCGCCACGTCGAGACTCTCTTTCGAAAGTGAGATCACCACCGGCTTGTTGAAGAAGGAAAGATCACTCCGTCATGCAGCCGTGATCTTATATACGATACCACCAGACGCGTCTTGGTACTTCCATCCACCAATCAAGGCTAGTGGAGCGAAGGGAGCCAAAGATGACTGCTTACACCATTTATCTTCTCTTTCTCCTCGAGGAAAAAGCAATTGCTAGCAAGTTGAAAGAACGATAGGAAATTCAAACCAATCCATCCAATTTAGTCTAGCCAAGCCTTGGAAACAAAAAAGCAGTGCCAGATGCGGATTCTATAGCATCTGATTCTCAGACTCTATCTAAGTCAATTTCTGATTCAATTCCTGGTCGGCCCTCTTTTACTGATGTGCTTCTATCTGGAAGCTGCGAATCCTTAGCCTGCAAAGAGCCTATTCTATCAAAGAAAAAGAGGCTCGCTCTAGTCTCGTAACCAAGACTCTTAGACCTCTTCTTGGCATCCATGTCCTCCTTCCTAGCAACCTGTCTCTTTCCTTCAAACAGCCAATGCCAGCTACTCCTGTAAGCCATAAAGCGACGGGCTAACCGGGTATTAACTCCTCCCGTCCCGCTACGCTTCTTCCTTCCCACTGTAAAGGGAATCAAAGGATACCCGTTCGTAAGCTTTGGCATCGGGACGCATTACGATAGCTAGGCCGGGTGGGATCTAAGATTGGTTAGGATCCTAAATGTATGACCAGGATGTGAGACACGCTGGATTTCAAACTGAGCTTTCACCTGCTAGAGTTAGAAAGAGCCCATCTGAAAGAGTTCCATTGAAACTTATAGGAAAATAAAAGGGTGCGGGCCTGGGGGCATCGTCGTCTTATACAAGAGTGTGACTCGGGAAAGAAAGACAGGTGGCTGGTCTAGAGCTAACCACTGAAAGCAACATGCCTGGCTGGATTCAAGGATCAAGACCTACTTTACGACAACAGATGCGGATGAACTAGCTTAGCTGCTCGGAGAAAGGCAGGCCTTCCTCAAAAGAAAGTTTATGGGGAAGATACCCTGTGAAGAGATAGGGAATAAATAGAGGGATTAGGAAAGATAAAAGATATTGCTGTAGTCTGATATTGCCCCATAAGTAGCGCGAGCCGAATCGGGAACAAGAGTGGAAGCTGGCGCGGGAACAGTAGGAGCTCCTGCTCTTTCTGGACAGCAAACAGCATAGCCCTAGCAATAGGAAGAAAAGAGTAGCCTAACAACTACCTACACCGGGGTGGATTGACTTTGATTTCTATCTAAGCATTGGTGTAGTCTCGCCTCTCGCTAATAGTGAAAGTGGTAGAGGTCACAGAGCCTTCCACAGAAAGAACACCTTCGTTGGAGCTTTTGATTTTCCACAGACATTTCGCCCATGGTTCAAAGGATTCCGATTTCTGCTCCATGCTGATCTAACAGTGAATTCTCCATTTCTGCTGAGTTTCTATAGCCGAACGTCTTTTGTTGGGAATATGGTAAGTGGAATCCTGTCACAAATAAGAGAAAGAATGGCTTTTGTGGTCGGAGGTAACACCCATGCTTGTAGAATACCAGCAATGAGCTGAGCAACTCGTAAAGAGAAATTGTTGGTATTGGAGTGAAATTATTAAATTCCTCGGCAACAGAGTTGGCTTGTTAGAGCTAGTTGAGTTAAAGTAAGCGAGCTAGTCATACGAGCCAGTGAGCTAGGCAGCTTGCTGTAGTTGCTGCAGTAAACTGGAGTGAAAGGGCAGTAGAGACTGAGGACTAAAGATTTCTCAGAGGTGGAGCTAAACCAGAGGATATATTACCAGATTCTTTCGCTCCAGGGATTGGGATCTGAGTCAAAGGCATCGATCAAAGAAATTTCGTATAGTTATGTCAGAAAATGCATTCCTTTCTCTTCCTAAAAGCTTTCAAATTCATTCCTCCGGCTTTGACTTCTTGGTATCGGCCAATGTCTTATATCGGCTAGAAAGAGTACTGGGATGTTCCGGAAGAACATGGTATTGTGGCAAGAGAAAGTACTCTACTATATATGAATAAAGGACTCCTTTAGAGCAAAGAAAGCGCTGAACACAAACTAAGAAGTACAGCCGGGAAGAAAGGAAAAATAGTAAGAAAAGAAGAAGGTAGGAAAGAAGAGAACCAGCTAACCCATTTCCAGCCATGAGTTCCCATAATGACATACAAAAGAAAGTCATTGCGCAATTCACACCTTTGACTTCTTTTCAGTTCCCTGGTCATGAGACCTCTTTTCGCCAAAGGCCCTTTTCGCCTTGTAGCTCGCTATCTTATTTGCAGCAACCTTCTCAATGCCGAGAGTATTCCAGTTCATCATCGCGATCATCGTCTTTCTTCACAAGAGGCTGAGCCATTTTGAGATAACTGAGATCTGCGGGAATTCAATAGAGAGTGGAGACTGCAGAAGTTGATTTAGTGAGACAAACACAAGATGAATTGCAGATCTCTCACTTAACGTCATGTCATATAAATGAAGTTTGTAGCGCTAGTAGAATCTTAAGGCCCCTTAACACAAATCCAATAGAAAGTAAACGTTGTAGTATAGTATGACAAAAATCCCTTCCGAAAGGGGACGGTCACAGATTGAGAGAGGAAGGAACTCCAAAGTCAACCTTCTCCTCGTCTGCTTCTAGTCTATCTCTATATTCTGATACTCCTTCCCGCGTAATTCCGCTATACCTCGCAGGAGCAGAATGAAGTGTCTTTCGACATTTAATAAAGTAGACGAAAGTCCCTCTTCTTTACTCTATAAATGTTGTACTTGCATTCCTTAATGAGGAAAACAAAAACGAGGTCTCAGAGCCCCCGCTCGTTCGTTGCCTTGAGAGGAAGACAGTCTAAAGGTCGCCATTTACCCTACCAAGCCGTGTTGCGGTTCTACGGTTGCGGATTGGTCTTGCCAATAAAGTGAAGAATGTTCAGGTTATAGAGACCTGGAAGAGCTCCTTTCACCAGGTTCCCAGCATGTGTTCTTCGCCTGGTACGGTTAGCTCAGAAAATTTTGAACCCATGACGCAAAGCTAAGCTCGAAAGCAAGGCGGAAGGTTCTTTTGTAGTTATCTCGATGGTTTTCTCGGAATAGAATCCGCTGTTAGCAGAAAATCCATTGTCTTAGAAAAAAGAAATGTGGGAATACCAGGGCTGTCTTATGGCAGCGAATGCCGGGTATAAGAATAGAGGTATACTCTCAATGGGAATTTCCCGTGGGAATACATGCCAATATCACCCTCTTGTTTTAATCCCCAGTGAAAGCTCTCTTCTAGGCGCGGAGGTACATACATCCATACAGTGACAATGGTTCACAGCCCGAGGGCTAAGTGAAAGCTTTCTATCTCAGCATCTACGGTGATATAGACTCTACTTAGTTTAGTGTAAGAGCTAAAGGAAAAGAGCTATGATTTATACCAGTATTCGTATTGGAAGAGTGACTTCTTAACGCTACTAGAGAGTTGACTATGAAAGATGAAATATTTGCGGTAACTCTTACAACAATCAATTCCATAATTCCAAAATATTATATAGATGCTGGATTACTGGTTTTCTATCTTTCCTATGCTATCAAGAAGCAGGAATTGCGGGATGCTAAAGAGAATGCTGCTGCTGCTTCTGAGTTCGCATGTGC

>Decalepis_hamiltonii_chloroplast_contig15

GTATATTCATATCATAATATATGAACAGTAAGAACTAGCATTCTTATTGAGACTAGAACTCATAGGGAAGAAAGTCGATTTATGGATGGAATCAAATATGCAGTACAGACAAAAGTATTCGGTTATTGGGGAAAAATCAATATACTTCTAATGTCGAATCGGGATCAACTAGGAAAAGTTGGAGGTACTTTCCTGAGTTTGGCTCAATACCGAGAACCTGTGGCGGAGGTAGGGTAGGACCCACCCGCTCGAAGTGGTACAAATCTTGAGATGAGAACAATATATTGTTCTGATCCCAGGCGTACCAAACTCGAGGCATCCACAGAGTTCCTTGAGACCACTGCTCACCATATTGATGAACAAAGGTCATCAAGAAGACAAGGATGACATTTCATAGACTAAAGATAAGAATGGAATCTAACTCTCCCATACTTCTCTCTAAGCTTAGAGGTGTGATCAGATCCTTTGTCTCACGGTATCATGAGTAGGAAGGGCTTTCCAAGTTGGGTCCCAAGTCATACCTTGGTTTAAAGGTATGGTTGGGATATCAGGAACATACCTAAGGAATAACTGCTTGGCCGACTGGCGGATTGGTCTAACCACTTCCGTTCGTTCCAAGGGAATTGAGGTGAATGTACTTTGACTCACCTTCTTGGCCAAGACAACTATCTTGTACAAGGAGAAGAAGTACACTTTTACCAACAGATCAGCCTTTGAGTCCCGTTTGTAGATTTGATGTCTAACAAACGACGGAATAATCTAAGGTACCCTGACCTGGTAAGGGAGATCAGAACAGGTAAGAAGTATGGTTTATAACCATATCCACCGTATGCCACCTGTAATGAGGAGGCACACTCTTTCAAATAGTTAGCGGTGAAGAGGATACCTGATTTTCGAGCAAGTCGAACTACCGGAAGGGTCGAACTCGAAAGGGGCGATCCACACCCGCACCTGGCGAAAGGGAAATGAGTTTTGAGTGAACGAACCCGGAAAAGTCCGATCTCTAGTTAGCAAGAAAACCGCTTCCCTGCTAATCGGAATGATTTTAGAGGTTCACACCCGGAAATCGCCGACTTAAAGGAGTCAAAGAACTCGGAAATCTCTGCCACTCTCGCCGAATCCTCTGGCCCTTCGCCCTTTCTTGCCTTTGGTGGTAGCGTATTCTAAGTAGAAGATCCAGTCTGCCTTTCTGATTCTTAGTCTTCTATCAAGGCGAAGGCAGAAAGAAAGATAAAGCATAGAACGATTGGCCCAAGAATTGCTATAGTTGACTCTTTGACACCGATAAAGGCTTTCAAGTCAGCAAGAATTCCACCATTGCCAGCATCTACAAGAGCTCAGTCCTTTCTCGCCTCGACAAGATCAGCGCCAACCGTTCCTGCTTCGGCAAAAGTGCTCGCATCTCCAACATCCTCGACAGCAGCTACGACAATACTTGCATCTCCAACAGGAACGTTAGAAGCTATTCCCGCATCGCCTACAGCTTCGCCTTTTAAGAATAAGAGCTGCGACAAGAGCTACAGCATCGGCAAGAGTTCCTGCTTGACTTTATCAGTAGAAAGAAAGATATGCCATCAACCCTATGCCAAAAAGCCAAATCGGCAAATGGTGAATCAGGATCTCCCATTACTATAAGGGGTCCATCGAAGCAAGCAAATTCTGAATCGACTTGCACTAAATTACAAATGCTGAAGATCTCTATCCAGCACCAACCCTTCTTATCTAAGCATCTGCTCTATCTAATAAAGGCAACGGGAACATCGCCCTATTGTTCAGCTGAAAATCTCCGTCTTTCTCGATCACAGTAAGAGAAGCATCGCCCCTACTATGTAAAGCTTCAGTGGGCACAGGCACTGATTGTTCATCGTCAAGATCCCCGGAAACATTGTCAATTGGGAGATCTCCTTGTCATCTGGAACAGGAAGAGGCATTGCTTGCTAGTTCATCGTAAAGAGCAACTCCCGCTTTCAGTTCTAGGATCTCCCTTGTCATCGGACACCGTAACAGGACCTTCTATAGGAAAGTGTCCAAAAACCGGTAAAACACTTGAATCCACAATCCGCTCTACCCGACTTTCAGCCTGGATTCAAACTCCCTAGCGGAACCCTTTCCCCTAGCGAGGCAGATATGTTCTTCTAATGTGTATCTAGTAGCGAGGAGAGATCTTCCTTTAGAATGAGCTATCCAACCGATCCCTGATGCTTTCTTAGGATTCCGGCTTGTAAATGCTTGACGCTTAATAAGAAGACCTTGACGTCCCTCTCGGTCGAGAAAGGCGAACAAGGGCGCAAGGAAGGAGTCAGCGCGTTAGAATGAGTCTCAGGCTGACGAATGAGGTTTAAGCGCGTGCAAGCTTTTCTTTTCTTTTACATATATAGAGTCGAGTAGGTGGGACTCATTCCACTCCAGCTTTCTACACTCAACAGAAGCCGCTCTAATCTAAGTTGAGGATGGGCTTCAACCCTTATACAAAGACTAAAAGGGCATAGCTCTCGTCGATTCTCATTCATTCACTATGCTCTTGGGCAATCCAGGCATTTGGGAAGCATTGAATCTGTAAAGCCTGATTCCAGCGTTAGAATCCGTAATGGCAGGAGGAAGGTTTCTAGCACTATGAAACGACTCTCCTTTCTTTCCTTTTGGTGTGAATGCGGGAAAGCAAGGCAATACAGCTATCGATCAATAAAGCTAAGCTGCTAACCTTTTTGAATCATGTTTAGTTCTCCTCATGTACCAAGTCCGTGCGACTGCGGAGAGCCGGTTGCAAAAGATCTCTTGTCTCCGAATGAAACCTTTGACTCTCTCTATTCTATCTAAGACTAGTAGGGTAAAAGCTTTCTCTTTCTTTATAGCATAACAGCCTAATACCCCTTTCCTTTACTAAAGAAAGGCCCTTCAAGCAGTACGGTAAGTTTCATTCCCGAGCCATTTTCTTATGAGAAAGGCAAAGCTCGAGTGCCGGATATTTTAGGTGACGAAAAAGCTAGTTCTACTCTGATCTGACTCTTGATCTAGCTACCGAGAAAAGGAAAGTCTATGATGCCCGCTCCTTTCGTTATGTTATCAGTCAAAGCTGAATTACACCGACCCTTATCAAAAGACGATTTTCACTTAGATTGGGTATGTGTATGGAACTTGTATCCTCAAGAACTCAGTCGACTACCAAGTCCCCTATCAAATGCATATCCCTGCCCTGTTGTAGTTGGAAGGAGAACATCCAGCAATAAAGAAAGTCCTGCTTTCCCAAACCAACGAGCCAAGAGAGGCCAAGAGCCTGGTGGAGTTACGACTATCGCTAGCAGTGATGCCTGAGTGAGCAACCCCTTTGATTAGAGCACGAGCTAAATAGTAATCAAAGAACCTCTTCTCCTATGTTAAAGGAAAGGGCATGAACACTTCCCATAGCTAAAATCTTAGCACCAGCCTTGTATCCTTGAGTCAAAAACTATCGGCAACAGCCTGTTTTGAGCCAGAAACTAGCAATTCTGCGCTTTTTCAAGTGAAACCCCTAAACCCTTGTTTTATCATCTTGCTTTTAGAACTAGAACTCTTCTTCTTGGTAATGTTACCCCTTTTGGTTTGGATAGAGCGAGCCCTAACAGCTCTTGGGAATGAGCACTTCCTATAGTCGAAATCGCTGTTTGGTGACCTTCTTCTATATAAGGGGATGGCATGAGAAGGGCCTCTTTTGGGCCTGAAAATAGCCATAGATTGGCTTTTCGAGTGGGACCCGAAACCCTTGTTTGATAGTTCTAAAGGCCGAAGCCTCTCTTGCTTACCGCAATAGATTCTTATATTATATAATAGAGATTGGGCGACACCCGCTCTTTACCCGGCACCTGCAAAACCTCTTCTTTGAATGTTCCCCTATTAGAAAAGCCGAACAAGCAAAGGCCTTCTTTCTCCGTGCCAGAATAGGGAGAAGAGAGAACCAATGGAAACCCCAACTCAAGAGAGTTAAGGTGCAAGTGGGATGGTACCTCGACCAGAGCGCTTTGGACTAGTCCGTACCTTAACGCAAGTGAGGCTCAAGCGTACACCTTTCCCGATGGAGGATCCGCGCATAAATCATAAAGTCAAATGGTCAAAGCGTCCCCGGAAACCTAGATCCAAAGAGTTTCCCGATCTTATTATAAAGGGAAATAGGAAAGACTCCCCAAGAACTGGTTGCTAGATAAACTAGTATTCCAAAACTGGTTGATAGCTGAACTCCAAAATAGCATCTTCCCTGCTCAGGACCCCGAAACAAAGAGAAGTTTTCCCCAATCCGGACTATGAAGGATTTCCCCGCCTAACCTTAACACATTCTAATGCTAATGGAGATTTTGCTTAACACATTAAGAGGAGGAAGGTTTAGTAAGAAAGCCCTAGTGAGTTCAGCATTTGAACAAGAGGAATGAAAGAAGGGCTTATTCGATAGCAGCTAAGACCGCTTCTTCCGCATCAACTGGTGATTCTTTCATTCCTAAAGAAAGTGGAATAAGTAAAGCTGGACCTTCTCCTTCATCGACAACCAAAAGAGAGACCGCGGTACACCTACTATATCTCTATATATATCTGATCGTCGAATCACTGCCTTGTTCAGCGAGAATCTATTTTGAAGTTTAGAGCAAGCCCCTTCCCTATTGTTATAGCGTTCGTGCCTTCACCCGTAACTTCCTTCAGTCCCTTTAGAGAAGACTCAAAAGGGTTCTATATCCAATTCCTCCTGCTAAGCCAGGTCCTATTACAGACCTAACAAATAAAGATTGGGAATAACCGGTTCAGCTGTGATCAAATCGACTCTTTGCGCAAATCCTTTAACCCTTAAGGGATGCCCCCTATTTTAGAAGCCCAGGAATATCTAAGGGTTTCATCATCCTATGATCACATTCTTATGGACTTATGGAGAATGGACTGCTATCACTCGTCTGCGAACGGATCCTCTCTTGTCTTAACATAATTACCAAATCCATTCCTACCGCTCGTCCTTCCCATGGGAGACTAAGCCGGAACGAGAGAACTGCGCTTACGTCTTTTGTTCACACCAAGATTGTTCCACGTGAAAGGAGGAAAGCTAGTCGAACTAGAGCGGGAAGGATTGCAGCTAGATAGTAGGCGGAGGAGAGCTAGGAGAGGAGAGTGTGATCTATGGGACCGCTCTGCGGCAGCCGAGCAGGATCGCGGGAAGAGGAGTGGCATAGGGGATCAGTCCTATGTTGGCCCTATAGCAGATGATGGGGGTCATCCGACCAGGCAGGGAACCCTACTTCTTTTGTTGAACGTTAGGAGAGGGAAGCGTAAGGTTTGGTTTGCTCGAGCTGCAGTTCTTCTTTCTAGCTAGGGATTGGGTTCCGCTCTATCAATTCCGTAACTCTTATCTACGATAACAGCAAAACTCCACTTCTTCAACAAGAGAAAAGATCATATCGGCAACAGCAAGTAAAGTCTGAAATGCCACAGAAGCAAGTCTTGGCTTAGCTGCATTATCTTACTACCGATGTCATACTAGACTCTATTATGACCTGAGAGTGACTGAACTACCTTAAGTCCCGAAGTAACTTCTCCTCTCTTCCCTCTCGGCAGGGAACTTAGCTAGAGGAATTCATTCTCTCGTACTTGAACTGCTGTAGACAAGAAGTGAAAGCCCTAGGGAAAAGCGTAGCGCTTATCGTTTAAGGAGTTGCTCTCTTCTCTTTGTAAGAGGGTTTGACGGAATTGAATCTGCAACTGTCTCGCAAACTCTTCCTGCTTAGGGATACAACCACAACCTATGGCAACTCCCAATGGCTGACTTAGTCCAGCATTGCCTTAACTTGCTTGTTAGCCTTAGGGCCAGATGAAAAACTCCAGGGATGTCACGAGTGGTATCAGATCTATAATGAGCCGGTCTTAAGAAAGCTCAGAGTCTATTCCTATGAGAGTGTGCAGAAGCTCTACGCCGATCTCAGGTTCGTGAGAGATGAATCAAACCCTTACCGAATGGGATCAAGTCGAGATCTTAAGAGGAGGCCGTTAAGGGCAGGAGGACTCAATCGAAACATCTCTAGCAAACCAAGTAGCGAACTGCTTGTATTGCGTCTATCGCCTATTTTGAAACAAAAATCCCTTCTTTCCTCAAGTATGATCATTTTTGAAGCAGGTTGGAAAAGGTCTTTCTTTAGGAAAAAAAGACGCCGTTTCCTAGCTAGATGCGGTAGTTTCTGGACTCCAATACCTGATAGCTACTCGAATCTTGCTTCTAGTCGCCGAGCTGAGGCTCTCTTTCCTAGGTTTGGCTAAGGCAAATGACTGAACCTATAAAATAGGAAATGAAATGTTATCCGCCAACTCCCTCTTTCTTCTTCTACTGATGCTAAGTATGCTTTGCTTTCCTCCAGGGATAGGACTGATGCCCGCTCAAAGC

>Decalepis_hamiltonii_chloroplast_contig16

TCTTTCCTTGACTTGAAAGCGTTCCGTAGGGGACTAGATGGTGAAACTGCAATACCCGGGCAGAGGTTCTCTAACAATACTCACATATAAGAAATAAAGACTCTAATTAGAATCCAGTAAAGAATTCATATTATGATTATTACTGCTCGGACGCCTTTCCCAAGGCTTCCTTCGCTCACTAGAAGAAGAAGAATTTATATATATATATATATATATTAAGAAGCGTGGCCTCACACGCCGGCCGGGCTTAATTTTCAAGCGCCGAGGGCTTAATCCCACCCTTTCTCATACTCCTATAAGAACTAGAATTAGGGAATAGGAAATTACATAGATGATCACAGAAACAGAATGGTGCCTAATAAGCCATCATGGGACCTATAGGTTAGTATACGGGGTAATCCTGGGTTCCATGCGAACAGCTGAAAGGGCCAACGGGCAACGCCTGAAGGAAGACGACAGCGCATACTTTGTTGTATGTGTCAGAGTACGCGTCAAAGCCGAGAGGGATTCCGGGAAGCTACATTCTGACAAGAAGGCTGTGATTGGTTGGCTAACCAAGGAGGAATGAAAACCCTAAGACTTGGCTCCGACCGCCGGGGAAGAGGTCGTGGTACTTCCCAATCTAAGGATTCAAAACCTCACTGAACAAACTCCTTGAGGAATACCTTCAGGCTTTTCTGCCAATGTTAAGAGAAAGCACTTAGATCAGGCATGCGGGTGAAGAAGCTGGCTCCATACTGGTAGAAAGTAGAGGAAGAAAGACATCAGTGAAACAAGACCTAAAGGGTTAATGTCACAGTCTTTACTCATTTCCAATACTACATGGAAGTGAAGCTTCTTTCTCAATAGGTGACGACAGGGCCAGAACGGAACGGAGGAGAACGGATAGATGACTAGAATAGTTCCGCAAAAGCACACTCAAAGACAGGCGAGCCGAACCTTCATTAGCTGACTTGGGAATGGAAGAACTGGGGCACTGACCTATCATTCCTACTCTTCTTATTCTCTTGGTATTGACCCTGACAGAGAGTTTGTGAGGCGACACAAGTATGGTATACAAAAACTTCTCGTGTAACCTACATATAGTGGTTTCTCCTTCCTTTCTCCTGGAGAAAGTTTAGCTATGCTTTTGAAAGAAAAAGAATGCTGGTCAAAACTTTCGATATAAGCATCGTAAACGGGCACCCAATCGACTCCACTCATAAACAGATAGATCAGTTGAACTGGAAAGGCTTCAACCCTCTGGTTGCTCGTACATTCACCATATTATCTTATATAAGCCATGAATGTACAGCCGAAGGCACTGAAAGTGCTAAGACCGCCATCTGCTATTTATCCAAGGAGGGAGTTTACGAGCTGAAATGAATGAGTTCACCATTGGGCGCTACGCGCAACGAATGAATTTCATTTCACCTTTTTTTAAGAAAAGAAGAGATGGGCGATCCGCTCTCTTATATACTGAATAAAGGGATTGAGAATGCCCTTGCGGGCCTTATTTAATTGAATATACAAAATAAAGTGCCCAAGAGGGATGAATAGATAGCAGGACAAGAGTTGCTTGCCCAACGATGCGAAAGAGAAAGAGGAGCATCCAATGGCTGTCGAGTTGAACAAGAGCAAAAGGACTGACCACTTCCCTGCCCCGAGTTGAGAGGAGTAGGAACTTCAATAACCCAAAGTCTGGGGAACTTCACTACCTGAACCAGATAGTGTCGTGGGAAGAGTAATAGGGTTGGGGCTTGTCTCAAAATAGGCTTCTTTTCGGTAGATTCGCACTACAGGCTTGACGGAGTTAAGTACTTCGCAGCCCATGGGAAGCGAGGAAACAAGAGTTCCGGTACTACCAGACGAAATAATCACTGAGCCGTATCAGCCAGACGAAGCAAGAAAGAGTGCCAGTAGCTGTCCACGGAAGCAGATATCTGTGAACAATTGAGAATGTTATCTAAAACATCCAACCGTACTTGAAGAACTTATGCGGTTGGTCTTTCCGATCAGGACGAAAGAGAAAGGAAGGTCTAGCAAGTCAGACGCAGTAATCGAGTAATCCGTACAAGCTCTTACGAACCACATTTCATACTCATAAAATGCCTTAGCAGCCTTAATAATCCTCCATCCGCCGCATTGAGCTTCAATTCTCCACGCCTTTCTCCAATGCTACACGTTCCGCTGACTGGGACCTCTTCTTAGGTTGATCGTCATACGGACTTGGGTCAGTCAGTCGCTACCTCTAATGCAGTCGTACGTTTATGCTATGGTATCGGAATGCCTCCTAATGGGAGAAGTCTACAAGGAAGAAATTCTGAGTTTGCTTAGCCCTTCGGGCGCTAACCGGCCAGCTCCAGAAGGGAAGATATTTGGAGTTGCTAATCCGCGTTGGTAATAAGTTTCGAATTTCTTTCTGGCTGAAGGTAGAGTTGCATTTGAGTTACTAAAGTACTGTAGTCAGGTAATCTACACTGCCAACAAATAGCCAACTATCGATAGATAGAGTGAATCTAAGCCGAGCCTTTTTCTCGATTTGATATCAAAAGCATATTTGGCGTATGAAGGATCAGCTCCAATAGTCTACAAGCTATGGCTAAGGGGCAGGAGAAAGTTCTTAGAAATCAGGGTAGGTGCTTAGGCCGGTTTGCTTGGTTATCGCCACCAATCGGAATTGATTCATTTTATCTTGATGTGACACAGACTTGAAAAATTCACTAAGCTTTGAATGGAGATTTGGCTTTGTGAGCCAAGGACTTATTCGTTGATTGCAAGCTAGCCGCCAGTCACCCTTGCTTTCTTTTCTTGTTTGGTTGCGCCCTTATGACCTGTGCCCTCAAAAGGGCTTTTTCGGAATAGAGGAATAGAATAGTGAATCCATTAGGAAAGATATGCACGGACAGAAGGAGCTGCTTTTGAGAGAAATTATACCCATCTAATTCATCTTCCTCGATAGCATCCGATTACTGAACACCTTCACAATCCGACGCCACACTGAAGCAAGGGATGACTCCTAGAAGGGCTTAGTGGTTAGGTTGTGCCCGTGGATATCTTACTTATTGATCTGACCTTTCCTTTTCGGATCTTAGATGTCTATTCAATCGGAATTTCGAGAGGCATATTCATGTGCAACCTATTCGTCGAAAAGACTAGCAAGCTATTCTCTAACAGCGATTCTTCTTATTCTCTTGGCATTCCTCCTTACCTGTGTAAGTTATATGAGATTTTTAGGAGGCTTTCAGTCCACATAAACGTCGATGCAATATTGATTACACATGCCCTCCTTCTCTCTGCTTCATGTGCAGCTCCTTCTTTCAAAATGAGCATATTCGATTAGGCTCTAATCCCGTATTCTTTCTAAGAAAGCCTAATTGTTATCCCTTGACACGAAAGAAAGAAAGGGAATCCCCTGCCGAAAGAGGTAAAAAGCGAGAATAAGGCTAAGCCCTTAACGGCCTCATAAAAGGTATCTAACCTCATAAGAATGGCACAATTAGCTTATTTAGTCCTCTCCCATCGGAAGGAAAGAGAGTTAGCTCTCCATTCCCATCTCTAAAGGAAGGAAAGAGATCTATTTGTAAAAGCTACACCCTTAGCCCTTCGGTCCGAACCCTCCTCGCTACAACTGGTTTGCCTAATCGCTCGAAAGCCGTATTGTTGGCTTCCTTCGCTTCCCAAAAGCGCATATAAGGAGAATCCAATACCGGAATAAGAGATTTTAGCTATCGACTATCGCGAACTAGTCATAGGGCTAGCGCCACCCTCATAGGGACTAACGCTCGTAGCCCGTCTGCTAGTGAATGCAGAGAATGAAGGGAGTACCTTACACGCTCGGACGCCTTAACCAAGGCTTCCTTCGCTCACCCAGGGGATACACAAGGAAATTCGTGATCCCAGTAACCGCTCTTGGGCTATATTATAGCCGTATAAGTACAGCCCTACCAATTTAGCTCTAAAAATCGATACGATAGTTAGTCATTTTCATTTTGATGCTAAAGGGCTTGAGTTTGAGCTCTCCCACTCGATGAGAGGAAAGTTGGCTAATTCAAAAGATTGAGCTCAATGTATAGATCCGAGTAAGATTCATGTGCTGCATCGATTGAGGGAACCGCTTCACATCCATCCCAGATACCAGAGACCCTCTATCATTCAGTGAGATCGCTCCTTTAAGGATTCCACTTTTCTATATGAATATGATCCCATCACTTGAAACTGATTCAACAACAGCCATTCTTCTTCCAAGAGCAAGAATATCGCTTATTCTTCATCAATCTTCCTTTATGCCTCTGTTGTCTTTTGCCGGGTTAAGGCCTGTTGAGACAATAGATAGTCAGGTGGCCTAGCCCTAGCTAAGAGAGATTCTTTAACCACGAAAGCATCAACCATTTAACCAACCTTTCACTCTTTCATATCCTACCGAAGCTTTTCTCCCGTAACAGGCCTGCCATGTGCAAGAATCCAAAAATGTCTTAGCTCGTTGCCTATGGTTTGCCAGATTGCATAAAAGTCTACTTCATGACTCTTCCTTCTTTGAAAGCAACTTAAGCCCCGTGAAAGGGGAAGTCTCCTCCGCTGGGGCATCTCTGGGGTAAAGACTAGGAGCCTAGCTAGGTCCCTTGTCACTAATCCGAATCTGCGGAAGAGTCATCCAACTATATGTATATTTTATCCCACCAACCTGTGTTATCTAGCGCGGTCTTGCTAAAGCTAAAGGGTTTCCCATGAACTCAAGTGTCAAGCAGCGAAAGGCCTATTTATAAAAGAAAAGGTGAACGTGGTGCGACGAAGCACTTTCCTGCTGTCAAAGGGTCTTGGTGACTCCCGTTGTAAGGCTCCAAGGCCAGATGCGGAGAATCGACTGCCGGTGATAAGCCGGAGGAAGGTGAGTATGACCACTGAATCTAGAAACTGAAGCTACAGAAAGGGCATCAAGGAAACTGAAATGCTAATACTTGCAGTCTATGTCCTCGGGAAAAGGTCTGCTTCGCCCCGTAAGAGAAATAGAAGGCAGCCTCTTTTACCTTTCGACTCTCATGAAGTTGAAAGCAGGGAAGCTTAGAGACGGAATTCAAATAGGTGAATAGAGGAGGGGCGAAGTCAAGATAACTCTAGCAATATAGACCTATACTATATGTATATTAAGAGAGTGAGGAATGGCATAGCTAATAAGGAAGACGGAACTACTATTGTTGGATTTTCACCGGTTCAATCACTCCATTCAATGAATGGGAAGACCTTCTCTTCCTGAAACTTAGGATTAAAGGCTACGATCAGACATGTTCCCATGAGGAAAATTAGACGATCAGCCAACTAGTCGTAGATCTGAAACTGGGGTGGGATTACTTAAAGGCTTAGTCCGCATTTCACTTCGTCATTCCACGAATCGACGGGCTTAAGCACCCGAGTGCGGGGTTCGATGGAATGGAAGGAAAAGACTCCATAGCCTGCCAGATCTTTCTCCTAACCTCCGCTATCCTGTTTGAGCAGATTGAAGGATGGATGTTTCGAGGATGAGTCGGGAGCCGCTGTCGGTATTGCACGAGTAGACGTTAGTAGTCACTTTGGAGCCTGTGAGGAATGCCCCAGACTCCATAGAGGAATTTGCCGCCGGTCGAAGGTTTTGAGATAAGGGCGGTGGGGAAGGTGCCAGTCAGTTGCAGGTCATACCTAACTGGAGATAAAGACTTTCCAGTATTCAAAGAGTGGGCTATTCGGTGACAATTCGTTCCCATGGGAAGACAGTGTTAGCGGAAAAGCGTGAGGCAGGAGAGGCGAAGGGAACGTTGTTTTTCAGAGGACAAGCAAGAAGACAAAGGATTCACTAAGGATTGTCAACGAGGGAAGGTTGGCTGAACTCCCAGAGACTTGGACACATCGGAGCACTAAGTGATCCCGTAGATTTTCTTGCAGATTGAAAGAGTGAGGATTACCGTCATCATGGAAAAGGTTAGAAGGCAAGAGGCGGAGCACTCGACCAACTAAGAGGAGGTGTGAAGCTAGAAGTCAAGCTCGTGTTAAGAGTACTAACAAAGTCAAGCGTTGAGTGGAAAAGGATAAGAGAACAGAAAGTCATAGAAGAGGAGTGAAGGCATTCTGTCTGACTTTTCTACCAAATGTAGCAAATTAGTGAGACTTTAAGTCAAGATCTGGTCAGTCACATTGATGAATCCATGAGAAGAGATAGGCCATGACGA

>Decalepis_hamiltonii_chloroplast_contig17

TATCGAGCCGGAACAAGCTGTTCTTCCTGAATATCTTGATTCAAGGCCAAAGAATTTCCGCCGCTACCCTAAAGTATTCATCTCTACCTGGTGATAAATAAAGCATCCGTACTCCTGTGGATCTCTCAACAATTTCATAAAATGGACTTTCTAGAGACCCCCAATGACCAATCCTCGCATGAATTGACAAGGATCCAATAAGTCCAACATTGATGCCTTCAGATGTGTCGATTGGACAAATACGCCCATAGTGACTAGGATGGATATCTCGTATCCGAAAACTAGCAGTTCGTCCTCCAGGGCACAAATAACTCAATTTTCTCCCATGAACTATTTGTGTCAACGGATTCATTTCCTCAAGAGATGGAGGCAAGTTGGGTACCCTATGAGAAAGACTGACCGGGGCAACCTTCACTACTGGAATTCCGATTCTGGTATGAGAGTCACCCAAGTAAGGATCACTTCAACTTCCCCGGTTGCAAAGACCGGACCCAATCAATCCAGCCGATCCTGATGCTGAATGCGCTTACTCAATCCCATCGCCTCATGCCTTCGCTTTCGATTCCAAACGATTCCTTTAATCAAGTCTCAAGTCTAAAGCTGGAATTGCGGATTCTTAGGCGGACAAGCTATTCTTGCATCTTGCTTCCTTTATTCCACAGTTTCGTCTTTCCGAAATCTTCCCAACTTAATGCGCTTTTATTCGAAAGCAGCTGACTTCTAACTCTTCTCTATCTGACCTGAGTAGAAAGTAGGTTCTTGTGGCCTAGGATACTCCTCTTTCCTTATAGACAGAGCCAGTTCTTGCCTATTCCTAAGTAGTAGTCGTCTTAAGACGTAAGGTAGTGCTTAGGGTTGGAGTTCTAAGCTAAGCAAGCCCCCGCAGTTCCCCTATACCTTTATTCTCCGTATGACAAGTGCCGTCTATGACTAGTATGCTTATCCCTATTTTTATCCTTTTGAGGGTTTCCCAATACCACATTCCTACCAAGGGATGCCAGTCAAAATATAATGCATAAGAGGGAGAATCCGGTTAAGAAGAATCTCACCTCTCATACAGAGGGAAGGGAAAGATACTGATGCTACTATCAATACCAGGTACCGGGCGAATCATATCAGTGAAGAACCCTTACCTTGCTAGCCTTTTGGCTGTTCATGCATCGTTCCTAGCTAACAACATAGGAATGATAGAGAGTTAGTTCGGATCCAGTCGCCTGCCTCCAACATTGGATCAATAAGGATTCAACCTTGGCCAGGTCAATCAATGGGACCTAGAAACCCACCCAATCAAAATATCCTTGGCCATGCTGTGAGAGGCCCTCTATTTAGGCCTTCCTTGAAGCAGAAAGACAGTCAAACTAGAAGTTCGCCTTCTACTCCCCAGTTCAGGTGCTACTTTTCTTTGGTTCCAGCTTCAGCTTCAGCTATTCTTGCAGGTCGGGCATGAACTTCGGATTGAAAACTACTGGAACAATAAAGACTGACAAGGTGCGACTTTGTTTATCAGTTCACTTGCAACCACACGGCATAAGGCTTCACTGACACGTCTATAGGGGGTCTGCACAAAAGCCACGGAGTTCGAGGCTAGGAAATCTGTCCGATCCTTATCTTACATCTAGTTCCTCTCCAGACGTTGAGAGTTTTCAGCCATCAAAAAAGAAAGAGTCTTAGTTGCTATATATGCATTTCATGGGCTAAATTAGGCACATCCAGTTCCCAACCATGAAGTGCTAGTTGGACTCTCTTCCCTCCCAAAAACCAGTTTCGAGAACCTAGGAATCCCCAAAATCCTTATTCCATAAGTGAAGTTCTTCCACCCAATACTTATTCCATTCCGCTAAGAGGATTTTATCTAAATGGGGAAAGACCTTGTTGACATGTCAAAGTGGGAATCGTGTCACGCTAGTGCCAGTTCAAGTTCAAGTTCTACAGCAAGTCTCAAGTGCCTTCCTGTTCAAGTTCCTTCTTTACTTCACTGCTCAACCAGCTCTGACTTGACTTTGACTCCCTTTCTTTATGATCTGTTTTTAGCTCCAGACCGCTAGTGGGACTTAAAAAGAAACTGTTCCCTACTCCTCTCCTTCTTTGTTTACCAATCTATACTATATATTTGTACTACGAGGTCAGGTTTTCCAGCCCAGTCCCACCACCAGATGTGATTGTTAAGGTGGATCACTAAATCCTGTTTCTCTATTTTATAGGCATAGGCGCTTAAAACAAGAGCTAGTACCCCATATGTTTGAGCTTAACTCGCATCTTCTAATGGAGAGAACTCAATGGTATAGGCACTTGAAAGGAAAGAGCGGCCGATGGACCAGAATCCGCCTTCCCTAATGACTTTCTCTGTGGTCGAAGGCTTGTAGCGAGGGTTAAGTAATTGTGTCAATGAGGACTAGCGATCACCCGCCCAACGATACTATATACAGAAATAAGAGGCGACAAGCCCCAGAAGCTTGCCGAATACCAAGCACGCAAGATCCGATCGGAAATAAGCCAAGAGGAATTCGAAGAGTGACTTAAAAGCTCCTTGACTTTCACTTTAAGGAATTGAATTCCCTTGAATTGCACGAGATTCATATCTCTTTGCGGATTCCACTAATAGATAGATGGGATAAGATTCTGACTACCGCGCTGGCCGTAGCCGCTTTTACATTTTATTTGGAATACCCTTGCAAACGAAAGCGGTACTAGTCTTTGTTGGTGTGATCGTAACAGCTGAGTAAATCACAGCGGGAGGCGAAAGCTCTATTTAAAAGGGTTTACGTTTGTGGGTAAGACTTCCTCCATATATGGAATGGAATCCATAGGATAAAAGTGGACAGGATCCCTACCCTAGGAAAGATAGACATTATAGGGAGGACGATCCACAAGTAGGAGCTGTGAACCTGTCAGCCCAACCAGTAGCCGGTCTTGCATATGAAAGGGAGATGGCATCTCCTGGGATGAATGCTTTCCTTCGTTACCCGATTCCTCTCCTTTTAGTCGAGTCATTTCATCCCTCTCCTGATTACTTTTCTTTATTGCCATTGCCTGCTTTACGATAAGGAATTACCGGGCTTGTCTTGGGAATAGAATCAGAATAGGGTTTTTCCTATTGTTGGTCTAGAAGGAAGAAGAAGCTGCACATGAACTAGAAGGCCTTACAACGAACTTTCAGAAGTAGCTGCGAGAATGCCTTGTTATAGGTTCTTTGCAAATCGCTTGTTTTCAGCTTCCCCAAAGAGGCTAATTCAGGGCATTTGTCTTTGTCCATTAACATTAAAGAAATGAAGGTACAATGGGCTGGCGGACCGGTCTTTCCAATAAGCAAGAAGGAAAGGAAAGAGAGAGAGTTCCCGAAAGGAGAAGGAGAATTTCCCAGTTCTTGAATTCACAACTACGAGATTGACATCCGCGGCAACCAAAGCAATCTGTAAGCACCCTTAGGGAAAGAAAGACTTTATGACCGCACTCAACAAGCACTCGTTCTAGCAGATATGTCTTTGTCACAGCCAGGCCAGTGGTTTTCTTCCTGATATTTGACTATCGGGATTGTGACCATCCTATGAGATTGGACGAGAGGATCCACACTACCAGCACTCTCAGGCCACTTTCCCAAGACAATAGGCTACGGGCGGGCAGCCTCAAACGAGGAAGCAGCCCAATCTATTACATACGATCACTGATCGAATCGGGAATCGGGGCTTTCTTCTTTCAGACATGATGGATAGAAGATCAAGATCTGGGTAAACCTCATCAATTCCATTCTATGCCGGGGCGGTGCCATCATTTCTCCGTTTAAGCTATTCCATGGAATGGGATGTCTTTGGCAAAGCCCAGTTCTTTTCTCATAGATAGAGCTCGTGACTCTTCTTTTGAAGCGCTGACAAACTTCAGACTCTCAAATCAATTTCGACGCCGAGAATGATTGATGTGTGTAGGTTTAAAGAACAACTCCATAGTGCTATACACAAGTCAAGTCTTTTCACAACCATGCTACCACGCCTACCTCTAATTGAGCAATCCTTGTGAATAGCTGAAACAAGTTCTACTGCCAAGCTAGTGGGATCGATCTCTTCTACTAGTGTCTTTCCTTTGAATCTTGATAACCCTACCCCACAACAAGCCTCCCAAACTATGAGACCACTTCAAAAAATCCTTTATGACGAGCTATTCGAACTATGCTCATCCGAGACAAGCCTGCTCAGCCAATTCTTTCTCCTTTTCCGAGGAAGCATGGTCAAGTGCCGAAGACTTTGACTGACTTTCAGGATGGAATTCAAGGGCGATGAGGAGAAGCCTTAAGGGCGGTTAGGCTCAATTCCTTTCATGTTGATGTACTGCTCTTCCCTGTTGATATAAAAGAAGGCGCTTTCGGGCGAACTAAGGCCTTTGTGTCTCGACTTCCAGTGTTGACGTCCCACTAGTTAACGGTGAAACGGATTTAGGAAAGCATATCAGGCAAAGTCAAGTCCTTGCTTCTCATCCCGGTCAAGATAGATATAGAAGGGAAGGAGCCCTATCGCTCGGATAGTTCCACCCCTAACTGGAAGTCTTGTAATTTTGATTTCGGTTGTAGCGCGAGTTTCCGTTTGGTGAACCCTTCTCAATCGTCTTATTGGTATTGTGGTAAGCGGCTTCTTGCCAGGTGCATTGCCTTCCTTGTTGTTGATTGTACTACTCTCTTTCCGCATTCGTTCATTTCTTTCGTAGAGGGAGTGGATTAAGTTGACCTTCTTCCTATTCTATAAGTAAGTTCCTTCAGCGGTCTAAGCCTGCTTTGCATTACGTATATCAGTAGATTCGGTAATCATTCTGTTAGCTCTGAATCCGTAAGGATTCAAGTAGTGGCTCAGTGTAACCGACCCCGCAGGGCATAAGGGCCCGCATTCGTGGGCTTGATTCTCTCATGATAGAGAGTTGACGAATACCTGAAAGCTAGTGAGAGAGAGACTAGTCGTCTAGCTCACGTATGTCAAAGGATGTGCGGGCAGGTTCATAGTTCCCAGTCTACTCGACATGCCGATGACTTCTTACATTACAATACGAAATTTGTGGATCTGACTCTTGAGTTGAACAGTAAGTATAGCGATGTGAAGGTTGGAGCTCAAACCTACTTGAATTGAAGAGGAGATGTATAGCCTTGGTCTTGGCCTTGAACCTGGTTTCCCAAGTTTTCTTACTGAGTGAAGGGATGCGGGGTATCCTGGGCTCTTTGAGGAAGGCTAAAGTAGACCAGTTGATAAGTCCACAGCTATGGATAAGTGGGTAAGTCCACTACTCGTGGAGGACCTTTCCCCACCTTGAACGAGGGGATTGGAAAGTCAGTTCACAGCTGCTCCCAAGGCAGGGAAGTTGAAGCAGAGAAGGATCTTGGTAGCGGGCAAGTCCCAAAACAAGATCTTAGATTGCGAGTAAAACAAAGACATTCACTCCCAACTCCTTTGAGTAAGTGAGCGATTCAAAGTCTAAGACTTTAGAACAACCAGAAGGGATGAGTGAGGGCAGCGATTAGTCATTACTTGATCACCTTCACTCACTTTCTATACGAGAAATTGGTTTATGGAAAAGATGGTGTAGAGAAAGGATGAGATGGTGCTGGGCCCTTTCGCCTAGGGAAGGCCACCTAGGTCTCTAAATATTTTATAGGCTTGAGCCAAGGGTTGATTCTTTCGCTCGTTAGCAGGGCAAGGTTCGGAGCGAGGAAAAGCACTCACCCACACTCCCATCTTGGAAACGAGGCTTTCTTACCAAGAGGTAAGGTGACAGGCGAAACGAAACTCCGGCTATCCACTATGCCATTCACTTCGCATCTCGAAAATCGAATGGAACTACGTACCTCTTGCTATGAGGATGAACTGGATCTCTCATTCTTTCCCCTTTCTTACTTATCCCCAAGCCAACAGGGGCTGCTTACCCAACAAAGGCTAAGCAGGAAGGCCCCCTACGTGGCAAGGGCGAGGTAGCCTACGCAAGGGCTAAACAGTCTTCTATTTTGACTTTTTAGGTAAGGGCTAGGACACTGTCTTATTTAGCTCCCCAATTGCCTTTCCAAATCTACTCATGTAATTGTGGAGTGAGTTGCTGGTCTACTGGAGCACCGGTTGAGGGCTTCTTGCGTACCGAAGAAAATAAGTCTCCCACCACAGGTAGGAAGACTTTTCCTATCTAATTTGACAGTGGAAGGAAAAG

>Decalepis_hamiltonii_chloroplast_contig18

GCTGCGGTGCGGTACACTGAACACCAACCCAATCTCGATCTAAAGCAAGTGAACGTATCAATTCCTTTTATCGAGATAGCGCTGTAGGCTAGGTAATTAGACTACCTAAACGATCTATCTCTATCTAATTCTCAGTCTAGCCAAAGATAAGATAACGATCTTACTCATTATCATCTTCCTATACTAACGGCTAGCTGTCTTTGGAACGGACAAGTGGTACTAGCCCGCAAATCTTCTTGCCCCTCGGAAAAACGAACAGGGTACGAAGGAGAGCAGGCTATCCTTTCTTGATTTGATTGTACGGGACGTGTCTTGGCTCGATTCTCGATTGGAAGGATCTTGACTGTCTTCTCTTTCTACATGAAGATGCATTCGGAGAAGAAGCCTAAGAGGTCCCTCAATGCAAGAAGACCAGACTTCACCGCTCGGTAGTGGTGGAGCTGTTCACGATTGAGCTAAGAGAGAGCAAAGTCGGTTGTATGAAGTGCAAACAGACAAACAACTTCTAACTCTAGTTATAGTACCCCTCGCCGACTGCTGTATGAGTGACTGATCACATCTCTATTAGTGACTACCAGACGTTATAGTGATCGCGTTATCGTCTTCTAGATCGCTACATCTACTTCAACTCAACGACAGAACTACTAGCCTAACTCCAACTCACGAGCGTAAGAAGAGCTACTACTAGGAAAAAATCAGAATACCCTTACAACTCACCTGCTAAAACAGCCTATTTAGTGAGTGTCTCATCTAGGCACTTCTAGCAGTTCACTCCTAGCTCAAGAGCACCAGAACACGAGCAACTAACAGCTATTAGCAGTCCCCTGTTTCCTTGCTTCCTACCGAGAAGCCAGAACACAAGCAAGTAACTGCCTCTAGCAGCCTAGCTTGCCTTGCTTCATAGAGCCTAGCACTGAAGGAGAACTGCTCTACTTCAAGGATAGCTGCAGTTTCTCCTAGTTCTAAAGTCAGCTACGACAGGTAATAGAGAGATGGTCTTTTAGAAGGCTAGTAGTCCCCCAACATAGGATCTTCTCCGCTTCTTCCTGGCATTAGCTAACTCAAAAGCAAGAGAGACAGCTTGATCGGACCTCTCGCGTTGCTGACTTCCTTCATTCATGCTGGTTACAAAGACAGATAGAAAAAAGGAAGGAAGTCGGTCGTAACCATTAACCACGTTCCTTCCATTAACCGCTCAAGAGCAAGAATCAAACAGCCTTGCTTCATTGCCCTTGTCCTCTTTGTGAAAGAGAGGATTAATAGAAGGAAATAAGAGGCAACAGCCCAACCCTTGGAACATACTACAGCCCCAGATGCCTCATCTCCACCATAAAAAGTAATCCATGTCATTGTCGCGGAAGACTACCATTCAAGAATAAGAGCTTTTCTCTTTGATTTCGCTGGATCCGACTCCATGGATCATTTGGGACCCAGAAATAGTCAAGTGCCAGCCATCCAAAAATCAGTCATATAAGAGCTGAATAAAGAGAAAGAAGTGGTTGCTTGTTTGTTGCCAAGAAGAGGACCTAAGCGCTAGCTCTATCCCGTCCCACTGGACTGCCCAGATATCAATGCTCTCGTCCCTTGTCCTAGAGCGCGCCTCATCAAGCCTAATTCTAACTAGGGGAGCCTTCGCTCCGCCTTCGGAGACTGTTCCGTCTCCCTTCATCTGTGCCTTTGACTGAGCTGTATTCGACCTCTGTTTCCATCAACGATGTAACTAGATCCGGTCGAATATACCAACCTGCTCAGTTGAGAGCTGCGCCAGCCATGCCAGAAGCGCATGCACCCGCGCCAAGCAAGAAGGGGAGATATCCAAGAAACCTATGATGTGGCCCAGCATTTGAAAAGGATCCGGCCAAAATCTCTTTTTGATCTGTCTTTGCAGACCTCGAAATCTCATCGCGAAGCCTTCCTCCAGGTCTACAAAATGCTCATGTCTCGCAAGGCACACCCTATATAATCTCCAAGAGATGATTGGCCCCACAGCCTAACATTTTCTTTTCTCTTTCTATTTTCATTCTAGATAGAGCAGTAAGAAGGATCTCTTATGCAAGCTTTCTTGATGAAGGGTACCTCTTTCTAAAACAAATTGTAATCTTCCCTTGATCAACCGAGCAAAATGACTTGCTTTACTAACAGCAGCACCTGCCGTGGGCTTTACTGACTAACGAGTTCTCTGTCGCTATCCGCCTAGAAGATAGCTTAAACGGGCTTTTCCTGGGAATAGAGAGACGGTTTAATAATGTTGCTGATGCCGTGAGCGGCAAAGCTGAGTTAGCTGCATTAAAGCTATACGAGCAGGCCAGAACAAGTGCCGCACGGACCGCGTTTAGATCGCTAAGTCCGTGACGGCTAGCAAACAAGCAGCCCAAAAGCAAGGAAAACACACTAAAGCGCGCTCAACGGCTTTTTGGCTTTTCGCGATGCCGAGATCACTTCTTCTTATGAATTAGAGTTGTCACCCGAGGCCGGGTCATAAAGCTTGGAATCCGCACAGTAAGCTTCGACCCCGGCTTCCGATCTGCGAAGACTGTTTTGTTTGTACTTTTTGTCCTCGTCGACATATTTGAAGCATTGATGGAAGGTGGATGCCCCGGCTGTCCCTCATGTAACTAAGGCCTTTCGACCGGTTAACAACTAAGTCACCTGTGTCTGCTCTTTTGAGAGGTTGTTTTTCCTCTGTTTATTCCACTGGTAAAATGACTTTTTGAAATTGAAACGGAAAGCCTAGGCAAAATTTCAATTTGGATTTTGGAAATGTCCTCAGTTTCTTCTTTGAATGATACGAGTTCTGTGCCATCGACTCTTCTTCGTCTATTGCTTTTCCCCCGACTTTTCATTCTATTGCTTCTGCCTCTGGCATTCCTTGGAACCCTTCTTCATTGAGAATTCCCCGTCTCTGTTCAAAGGAATGACGTAAGGAATTCCGTTATCGCCTACGCTGTTACTTTCCTTCTTTGGGTTACCGTATCGCCTTAGCGACTCCTAGTATCACCCCCGTTCTTTAAACTCAGTAGGAGCGCCTTTGCATTCTATATTAGAATTCGGTTTCCGGTAGCAATTCCGAATTTAGTATTCCCTTTCTTAGCGTTTCACACTAAGTTCTTCAATCCAACATTTTTTTATTGAATTTGATTTGAATAGTAAAGGGAAACCTCTTTACTAGTTGGGGGCTAGCGCGCCCCTGCTCTCTTCTTAAGCTTGCCCCGTGCTGAAAAGCCATATCGCGCGAGTACGCACGTCTATCCCCTTTTCAGCTTGACTTGATGAGCGAAGTAGGTTTTCCGGTCTTCCTGGCCTTCAGAACTAGTCGTTAATATCCTTCCTATAGCCTTCTGCTTTCAGACTTCGACCTTGTAAACGAACTCCTTGACTTCTTGCCTTCCTAACCCTAGCGCTAGTTGCACCATCTGAACTAGCAGTACCAACTGCTTCTGAAATGACTTTGCCGTTCATTATTAAAGGAACTGAAGGTAACTCTGAAACCTTCACAGGTGGGACACGCAGAGGCCGATCCTCATCACTTGCGACCTTGCAAGGATAGACGGTCTTAGGCAGAAAGAATTCGCTGAACAGAGGCAGTCTAAAGATAAAGCTCCAGTAAAGGAACCAACCTTCAAACGGCCAAGCCCGAAAAGCCGCCCTATCGGGATCTTTTTTGGGGGTTCAGACCCTTGAGAACCGCGCGATCTCATAAAGCGATTGCAAACAGCTCAAAATGAATCCGATGGTCTAAGGCCTTATCACACCTTTCACCGGAGATGAAGACCAGAAAGGACGCCAATTCAAGCCCACCTCCATAGGGATGGAAGTTATGGCACATACCTTAAGAAAAGGTCCTTCACTAGGATGCCGTACTTCTCCAAAACACTCTCGACGTCTGTAGACGCGACGGGTCGTCCACAATGCTACTAAACTGATAGTTGCTCTGTTTAGGAACAAGAATCAGCTTAGACACTGAGAAGATGTATAGATACATTTGTTCTAATCTGTCCGCCCGAACCATCTTGATCATTCGATCCGCATCTTTTCTACCAACGGATCTCGTTGTGCTCTGGGAGAAAGCCAACTACGATATGAATCCCTACTGGGAATACAAAGATCTTTCAAAAAATGCCTTAGCTTCGAGATCAACTTACTCTTACTGAAGAGAAAGAAAGAAGTCAAGTTGGACCAAAGAAAGAGAGAAAGCGACCTGCTTCAGTTCGACTTGCTTACACAAGACGAAGGGGATCACTATAAAGAAAAGGATGCTTTTCAATGAGATCTATGAAAAGAGACTGGTTTCATCTTTTAGATATTTTAGTAGGGCAGTTTCGCTCAAATCAAAGATTTCCGCCCTATCAGTACCCTTTTCCTTTTTCATGGGGATACCTTACTCTCGAAAGCGTAAGCTTATTTTGGAAAGCTTTTGTGAAACTTGGTGCCCGGAGACGAACATTCTGCATACTTCGGCTGGAGAGTTCTCAATCTCTCTTTGGGACTTGCGCAAAATAGGAGGTCTTCCCACTAATGGTGGATTGTACGAGGAGTTTTTCCTGTGCCAAGGCATGGATGACACCAATAAGCGATTTATCCCTCAATGCAGCGAATACTTATTTGCTGCATTTCATCAACTTCGAAAAGAAGAAGGAAGGCCGTTAAGGTCTCGCGATGGATTGAATTTTGGTTCAAAGTTCCTACCCAGAAGGGTAGTTCTTCCTTATGAAGGTGGGACTGTAAAAGCACCGGTTGAAGTACCAACTCCAACTACTGCTTTTCTACTGCAGCTACGGCTGCTGATCGATCTAGTCCTTTGATCTAGTCTAATAGTTTTCCCGGTACCGGTAGAAGTATAATGCTACTATAGCCGAAAGGCATTAAGCTAATCATGGTTCCCGCTCTTAGCCGTTGGGAGTCCGATGGGGCTAGTCAAAATGCTCGAAATCACACTATATGAGAATTCCAGGGCCCAACAGAGGCATGCCAGCATCAGCTGAGGAAAGCCTTAGAACTAGCGTTTTGCTTTCTTTAGTGGTCCCTTCTGGTGGATCACTTCGGTGTTTCAAAGTAGCTCCTTCTTGTCTTCTTTTCAAGTAGGTGAGTTCGTGAGTAACCCTAGGGAGATGCTTGGAATCGGCTTCATCTAGCCTTGAGGGATTGGGGGCGGAAGTTACATATATAAAGTAGGCATCGAATACAAGCTGTTTTCAATTCAAGCCTAATGCCGCATCAGGAATAGAATCGGCTGTGAAAGAAGGGCCAGCGAAACCGGCGGCAGGAGCTAAAGCCCAAAACGGGCATTTAGGTTCTGTTTGGGGAGTCGAAGTTTCAAACTAAGAGGCCGAAACTGGGTCTTTATATTGCCCAACGGGCTGAAAAGAAAAAGAGAAAAATCCTTTTGTGCTTCGAGAAAGAGATCGGATTAGTGGGATTCCTTACGTTTTCAATTTCTTACTTATTATTATCCCAGCTTTCCAGGGACTGAGGGCACTACTAGTACTAGGAGTAGGGTTTACCTAACTAAGCATCTAATGTTTTATCCGCTCGCTCTTGGTCGTTTAGTAAAGGCATGGGCCATTAGGATTAGGGGCTAGTCTTTTCTGTTGATTTAGCTGTTCACACGAATTGCTCAAGGTTTGAGGCGGGGATACAGGCTTCTAGATGGGATCATCCCACACAAGGAATAGAGAGGAATCCCTATTGAGAAATGGGAAACCGATGCAATTTTCACTCTTGCAGTAAGAAAAGTATGTTTGAAGGGCCCGCTTATTCAACCGAAACAGCTCCTGCTTATGGTAGTGGATAAATGGGTGAACATGTATCTGCTGCCTCTGCTGCAGCTGGTGGATCGACGAGAGCCTAGCCGTGTGGTTGGTGCCACCTCCCGTGTGCTAGCTCTGCCGTCGCCGGATGCTTATTCGCCTACTCTTTCACGGAGCGATGACTAAGAGCCGGGGATTTCGATGTTGTAAGTGTCCCTGGAAAGAGGACCTGATCGAGAAAAGCCCGATTTGCCTTAGTCTTTGACCTCGGAAAGGGTCCATTTCGATTTCGCCATCTGGTCCGCGGGGATGCCAGAGCATGGGACAAGCCTAGATCAACCCTTCCTTCATTCCCGTTGTAGAGATTGATTATCTGAGAGAGGCGCTACAGGCTTCTATTCAGGGCGGAGGAAATGGGAAAGGGATCCTGCAGCGCTAGGAGCACACGGGGAGGATTCATCCATCGAATGCAAGAGGGAATTCAGCTGTGAGGGAGAAGACAGAAGCAATTGACATAGTCCCTGTCCGAAGTGCCACTCCGACGGTAGACAGCGACCCTCTCTCTAAAGGAAAATCTCAACAACTGTCTTATGTTTATGTTGAAGAAGAAGGTTAGCTTCATAGCTTAAACCTATCAAAGCAAAGGCGGAACAGCTGGAAGGTCATCACTCCACCTCTTTGGAGGCGTGTCGATTGGGCTTGGTTGGGAGAGGAAGAGCGAGATGATCGGCTACGAAAAGGGCCTCGTCTCGAGTAGCTCGGCTCGCAAAGCGTCTCCTCTTCCTGTATGAGAAGTTCCAAGAGCGTATCCTGTCTTCGTTCCCCAAACCTGAGAGTCAGCCTGAGAGCGAGTTGGCTGTTTTCCTTCCCATCCAAAGAGCTTCTAGCACATGGGGAAAGCTTCGAAGTGAAATCTCCTTTAATGAAAATGAAAGTCGGTCCAAGCCGCAATATCCTTCTTAATTGATCCTAATATCGAACCAACGAATTCAACGCTTTAGCCTCCTTTTCGATCAAGAGAACGTCAGTTTAAGATCCCATTTGAATGAACAGACAGGAGCCCCCCTTTCTTTTCTATAACTATAAAAGAGGAAAGGTTACTGATTGACATGCCAGCGGGGACAGGAGATAAAAGGCTATTTTCGCTGTCTGCTAGCTAGTGGCATTAGACTAGACTCTGAGGCTGGTGCCAAGAGTAGAGCGAAGGCCAGTGCAGTGGGTAGAGACTTTCTAGACTAGATATTTAGGAGTCTCTCCACTTCTTCCTTGACTTAAGGAATTCTCTTCGGCCTAGGCCTACCTTGGTAGTTGGAAGATCTTTCCCTTAGTTTGACTTTCTTTCACATGATCTTCCGTCGTAGAGAATGCTTCACGCCGTGCTATAGCGATGTCCTCAAGCGGCTATCAGTTGATAAACAAGCTTCCGTCATCTATTCTATGTATAAATGGTTTGATTTAGGCTTAGGGTTTGACACTTTAGGAAATACCAAAGAATAGAGACTGACACACTAAGCTACATTCAATGCGCTTCAACACGGGAACAAGCTCTATTCTATGCCGTGAGGAGGCTATGCAACAAAGATTGCATTCGGCACCGGTGATCAATCAGGCAGGATAGAATTTTCCCTTTCCGATCGGTCTTTGAGTAGACCTTCAGCCGCTTACCCTTGACTGAGGTTGGCTAGGGCCTTTTATTCATAATCATAGTATGTGGCTATCGCTCCTACTTCTGCTTGTCATTTTAGTACTTGGAATGTCTTTGGCTTTACGCTCCAACCTATCGGTCAAGTGTCTACGCTACTCACCTTTTTTGGAGTGGCATTTTGGCCCACATAGATGGAGTCATCCCAGCAAATGCCTTTTCTTAGCTTCTGCTTTCAGGCTTTCCGGAAGAAAGGTTCCAGGTTCATCAGGGTCGAAATCAAACCAAACCTGATAGAAGATTCATCAACGCTGGGCACAGTGCTTGAAGATTGAGGGCTTTCCTAAGTAGAAGGCGTGGAACTAGCGCTTCGCACCTTCCCTTCCCGTTAGAGGAGGTGATCAACATTAATCTTAGCGAGTATGGTACCCATTCCTCTTATTGGCTGGGGATGTACACAGCAGTCGAAGCAACGTATAATAAGCGTCGATCGCGATACGCCTTCGATCACCCGGATAGATTGAGTCAAGTAATGGCATCCACTGAGAGCTTACCTTAACTGGCATGACATTTAAAGAAGGCTACGTCTAGACCTGAGGAATCCCTTAGTCTTCCGGTCGAGGCATATTATCTAAGCGATTCCCCTAGTGCTTAGTGGGCCAAGTGTATTTTTAGTTAATCTACCCGTTTTACGGCCAGTTTCTTATTATCCTTCGCTTGTAGCGACTGAATCTTTTTCCGGTACAGCACCATTGATTGGTCTATTGGCAAAGGAAACCGGGAATGAAAGATTTTGTTCTGTCTTCCGGACATTTATTATGCTGTCACTACTGCTTGTCAATTTGAATTGCATCAAAGCTCTCGTTCGAACTTCATCTACGAGAATTCTAAACAAGAACTCGAGACTTGAAATGAATCCACCAAGAGGTTCTTTCTCAGGGCGTAAGGAATAGCACGCAAAAGTCGAAATCCACGAGAAGGCCTCTTTTACGGAGGATCAAGGGCGTTAGCCCATAGAGAGAGAGTCAAAGCCTATAGCACTGTAGGAAGACCAAGGTCTCGATCGACAGATCTAGTGGTCAGTCACCGTCAATATTGGATTCTACGGCCAATGCTGCGAAATGAAAATCGAAATGAAACCATGTTCCTGGGAAAGCCCTAGCCTTGAAATAAGGGGCTTTACTAATATAGAAATGGAAACTCTAATTTCACCGGACAGCTTTTGTAGCTCTTTTCTATCTAACCTTTCTCACGAAAGAAAAACAGATTCTTTGGTGGCAGTGGCACTTCCAGACACCGGATTCCCATCCACAAGGGATAGGGATAGGCGGTCGCTTAAAGATAGGCATACGTCAGCGTAGAAAGAAGAGATCCATGTTCTACAGAGATAGTCGGGCCTAGCTAATCCACTCACTCAGAAGAAAAAGAGCACCCGTCTGTTGAAGAAAATCGAAACGACTTTCCTCTTGGAGGTCTGCCAAAGGCGCTGAAAAGCGCAGTTTGGTAAGAAAACGGGGCTTCTATTTCATTCCATTTTACCCATTTGTGAACACACCCTGGGGGCGGCCTTCTCTGACTCGAAAATACATATTTCATAGATGGAAAGTTCATTCTTATTTTCCTAAGCACTTCGAAACCTCTTTTGCTTCCTTTTGCTTTTATTACATTACTCCGCTATATACTGTTTCGCTCGCTAGATGCTCGACTCAGGTATTTCAATTGAGTATAAGTACTTCCGATAGACTTCGTACCTCTAATCCCCGGGAACTATCTCATACTAACACTTCCAGAACAGTTCGATAAGCTGATCCATCCTTCCCTGAAAGAATCCCCGGTGAATCTTTAGCTTGGAGTCAGTACCTAAGCGTACATAGCTCTATAGCTCGCTTGGGAAGCAGGTCGACTTCGATTTCTCTTCTGCTGACAATGAACCTGAGTAGCTTACCTGACGAAGCACCAAACCTCCTCCCGGCTTTATATGCTTTTGCAGATGAATACGAAGACTGTATTTCCGCAATCTGTCAAACATTTTCTTCAAATTCACGGTCTTCTTTAGCCCAAGACTTGGCCTTGGCGATCATGTCATCGACATAGACCTCTATTTCCTTGTCTTTCATATCATGAAAGAGCCCAGTCATGACTCGCTGGTACGTCGCCCCGGCATTCTTTAGACCAAAGGCATCACCTTGTAACAGAACGTGCCCTCTTATCTGATATGGTGATAAACGCCGTTTTCTCTCGGTCTTCCTCGGCCATCTGGATCTGGTTATATCAATAGAAAGCATCCATTTTTGCCAGCATCCCATGTCCTGCAGTGTTGTCCACCAGAACATCGATGTGAGGAAGAGTCAAATCATCTTTTTGTTAGGCGTCTTTGGACTCACGGAACCAGCTTTCATTTCAAATTGAGGGAAAAACGTTCTCGAAGAAGCGTGACCATCTAGTTGAATCTCCTTACCCTTCGCGTGTGGTTATGGGGGCCGGCCTCCTTTCCACTTTGTTACTATAGAGCCGGGCGGCAAACAAGTGAAAGCGATCCCCCTCCATCAGCCGGTGTGTGTGCGCTTCGCTCCTTATAGCTCTACACCGTCGCCGGTCACTTTCGCTCTTCTACCATATTCATGGATTCATTCTTTAGAAGTTTGGCACGGGACTCGATAGCGCAGGCACAAGACCTTTGAATGCAAACAAAGAAAAGAATAGCGAGGACCACATATCAAAAGGTTTGGAACCCAAGCTTACCTTATTATTATGAAAAGGGAAGGTTTGATAAAGTAGTTGGTGTAGGTCTTTCCAATCGGATTCATTAATGCAATATGGAACTCCAAAACTTGAAGAACTGGCCTTGGAAGGAAATTTGGATTCAAAGACGAATCTTTCAGAAGCTGACATCGCTAGTTGACTCCTCCTCGTCGACAGCTAGCAGTTTCGGACTAGCTTTTGAAGTTTTCTCGCTTCCTCCTAAAGGGGAACACCAGCTAAGACACTAATAACAGCTAATGCCGTTCCTCCTCTATCTCAACAAGCTTCTGCTTTCAGACGGATGCTCTAGTTGCTGCTGGCTGCTCTTAGCTGAATGCCTTGCTTCGCCCTACAGCTAGTGGAGACTAACGACAAATAAGATTGACGACAGGAGAACACCTTGGCTTTACAATTTTTCTGTTATTACTAATCCTTCCCTTCTTTCTAGGCCTGGACTAATGCTTCCTTATGTCTGCCATTAGCTTAAGGCTAGTGTAACAGAGGGCTTCCTCGCAATTTATAGTCAGGGGTGAAACGGCTTGTTTGGCTTTTTCTTTCTGACTACAAGGATTAATGACC

>Decalepis_hamiltonii_chloroplast_contig19

TGTGGCTGATCTAATAGTATACTAATAAGAATATAGAAGTGGGTGGGAAAAAGAAAAGATAGGATAGGTCAGTAGAACGGCTTGAGATTGCTCTTACGGGTCTTGGTCTCTATCCGAATGCTAGGCTTTATCGGGAATTAGGTCTTGGTTAAATCCGCCCGGGCGTAGCTCAGTCCTAGAGTTATCTCCCCTCTCCTAATAAAAGAACTCCTTTAACTCCTTTAGCTCCTCTAACTCAATTAGCTGCAATAAGGTAGCGGCCTTGACTCTATTTCCTTTCCTTCCGTCCTCAGGTAGGAAAAGAAAGAGATAACAGAACCTTTCAATAGACTCCGAAAGAAAGATCTTAGACGCTAAGCAAGCAAGACTGCTTGCCCTGATTCCCTTCTCTTTCCTTGGGTCAATGTCAATAAGAAGGAAGACTCCAACCACCGCACCGGTACTATAGAGATGAGCTTTTGAAAAGCCGGAGATTGACTCCTGAAATGAAAGTGGTAGTCATAGGAGACCCACCCTGGGCATCCAATTCAAATGAGGAAGGAGAGACCACAGTCTTGATAGGGTGGTCCTCAGGATAAGGATAGAATCATCGCTCAGTGAGCTAAGACCATCGAAAGCCTTTTCTGGTCTGAGCCGCGAGCAATTCCATTTCTAGTACACCTTCGGTTCCTAATGTAAGGAGTCCGTGTCACAACGAGATTCCATACTGACACAATTAGACAAAAAAAGGGAGCTCGGATCTATGTATAAAGTATAAAGGACGAAAGGTCGACAAAGGATGATGTTCTAGGCCCAAGCCAACCCGGGAAGAGAACGAATTCGAACTTTATGGAGTGAAAAGCGCTATGCACCGCTCCGTGGGGAATGGCGAATGCTACATATGAATGTTTTGGAAAGACGAATACAAAGCCATGATTCAATCCTCTACTATATTTAAATGGAATTTCTCGTATTTTAATCGAGTCAATTCTTTTTTTTTGAGTATGAGTAAGAGAATGGTAATAAGGACTTTGATGGGATGCTTCCTCTGCTTTTAGTGAAGTTCAGCTTATACCAACTCCAAGTCGGATTACCACTGCTTGTAGCGCTTCCCAGATGTGGAAAGCTATTCATAAGGGATGCAACATATCAAAAATGGCTTGACTTTAAATGGCTAGTTGGAATGGCTCGCAGGTCTCTTTTGGTATGATAGATGGGTTGGAGATTTCACCCTTGCGATAATAGCTATATGGCTAAATTGCTTGACGATCTATCCAAATCAAAAGGAGCCATGTCCATGCGCTTTTCTCTCGCATAGAGCCAACCTTACTCTATGTGCTGTCCTGGGTGGGCTACCCTCCTTTTCATTCCACTACTTTTTAGCTCGTCTCTGTCTTCGTCTTTGTGTCTGCCGATTCAATTGATGCTGCTCGTCTGTCAGGATGGCTCGACGTGGATCCCTATTCTTATTTTCTTCCTAGTTCCGCCTTCTTGCTTCTTGACATAGTTTCCGTCTCCTGAATACCAACTTTCTGCAGGGGTGGATTGAGCGGTGGCAACGTCCGAAAGGTATGATTTTGTAGTAGATAGATGGGGTCCTTCCGTCTTTGAAAGGGCTTAAGAGTGCCTCGCCTTGGTCGGCGAGAAGAAAGATAGAAAAGAATTCAGGCTGTCAACTCCGAAGGAAAACTGGCCAAGTGTCTTGTAGTTGGGGTCCCTCCATCCTATTACATTATGTAGGCAAAGGCTGATATGTAGGCCGAGTGAGTCTCTTAGTTACTAAACTTATTTCTATATTACATAAAAGGAAGCTGAAATCTTCTTAAACTTTCTCACCCCGCTTTTCGAGCTAAGTGGTCCTGGTCTTGTCCACAGGGGTGGTTAACGACCGAATTTTCGGTAAGCATCTAAACTTTTCTTGGTCGGGTTTTCCTCCCGGGGAGGAGCTGCATCTTCTAGTAGGATCTAGTCAAAATCTATAGTGGAGATCTATTTCCTCGCTCCTTCTCAACCGGTTGAACTACTACTATTCTAGTTCTGCATACTATAAGCCTTTAAGCTTCTTCTCTGAACATTTTCTCCCTCCTAGGGGTTTGATTCTTCTGTCCGCGTCCTCGGATTGAACAATTTTGTAATGCTTGCATTCTCAACGCAACGGAAACAAGGGTTTACGTTGAGCGAATCGGAATAGCGTGGAATTCCCTCATGATCAACAACTTAGGCTTTTGCTTTGTATTGATGCCATTCTTAAAACCATCCACGAAGGTAACTTCAAGTGACTGCTATGAGAGCCGAATCCCTTTAACACGATAACTAGAATGGAACTGGAGTACCAAGGAGGTACACTCTCCACCCGCTATATCTCATTGGGTGATGGTGTATAATGTATACCGTATAGGCGTCTGAATCTGTTGATATGGATTAGCCCATTCTTATCGGTCTCTTTCCAATCGAATATCAGAAACTCCCCATTTGGCCTCCTGGTAGACTTTTCTAGAGCTTTCTGGGCTTCTAGAGTCTCCCGGTCGGGTCCCTCACATTTCCCACATGTCTAGCCGGTCCGGTTCCAGAGGAATCTAGCCTTTGAGGCTTCGTCCTTCTACACTAAAATCTAATAATATGTACAATCCGGGCCTTATCAAAGGCACAAGGCAATCTGGAACAAGTTTATGCTAAAAGACGGCTGGTTAAGCGCTACATCATAGGCACAAAGATATCCCTATCCCTAGCCTGATCGGCAAGTTTGTAGGTTCTTATTTAGAGCCTGTTTCTCTCTCTCGTTCGTGGAGATGGCTTTGGGACGGATAGTAACTAAGAAGAATAGGTTCTTCTATCTCCACCAAAGGGAACTAATCAAAGCCAGTATATTGATTTCCTTCTATCGAGTTAGAGCAAGTAAGATAGGAGACATTAAGAGTAATCCCTTACTTGTTTTCTCTTTTGTTTACTCTTGTTCTTGATCCGTCAAGTAGAGTGAAGAGAGGCATGGGTCACGAACCTCACCTCGCATGTAAAGGTCATTCCTCTTGCTCGATGGGATATTAAGAATAAGCAGCAGCCTCCGATGAGGAGATGGCCAAAGATCTATTATCTGTCCCGTTTCCTTGCCGCAAGATCCCTCGTGCGGCTGTAAACCACCTTTTAATATAATCAACCTAGCTAGCGGGCCTAAAAGCCCTTCTTTCTGCTCCTTCCGCTTTTCTCAGTCCGAATCTGTGGACCATATTTTTGTGGATTGTTGGTTTGCGAAGGCTCTGTGGTCCCGTTTGGCATCGGCTTTCCTCATCTCGCTTGACCTGGATATATTAAACCTTCACCTTTGTGTTCAAGCCATGCGGCTTAATTTCAGTCCACAGATTGCTTCGCTGTGGAAGACGGCTTTGTTTCCTGCATCTGGCTGGGGTGGAATATTAGAAATAGAGTGGTGTTTGAGGGAGCTCACATTTCGGCTTCTCTGCTGCTGCTACATTATGGTCTCTTATTCGTGAATCTAACACGTTCGATATAGGTCCTATGTTTAACTCGTCGGAAGAGTTGGCCGATGCTTCTCAAAGGCAAGACGTGAAGTGACCTCGCCATGCACAGAATCACACCATACACGCGAGTCTGGAGAGTCGGATGGAGCACCATTCGTATATCGAGTTTATGTGCTTTCAGTGGTAGATTCAAGACGATAAAGTTCTTATTCTAATTCTAATAGTGAGAAAGCTGTCCCTTGGTAGGCTCTGAATAGAATCAAGCTTTTTAAAATGAAAAGAGAGGCCGATCGATACCGTATGAAAAGAGGAACAAGAGACGAACGAAGTCCTACATCGGTGTTTGCCGGAGTATTGAGAATGTGGTATTGCTTTACGCGGTCTCAGTCTCAGTAGGAGTACCTTGCTGATATGAGTTGAGAAGTTTCCGGGAAGTCCCCTATAAGGCTAGTCCGGTTCCACTCTTCTCTCAATGTCAGTGCTTGGGGATCGGGTACAGCGAGATCACTTTCTAGCTTGTTAGTCTTAGTCTGCTATGCATGAAAGCAAGGAGGCAAGATATATCGTAGTAAAGACTGCTAGATTGGCTTCTTGTAGGAGAAAGATAAGCGCTTGATTTCTGTCTCTGCTATTGGATGTTGGAGGTACTTTTAACGGGCTTGAAGCGAGGTGAAGTTACTTTCCTTGCAGCGTTGGTGGAAATAAATGTGGAGGCACTTTTCATATGAGCTGTAGCGAATGAGCTGTGATCGAGGTAGCCCCGCAGGGAACCCGGATAAATTCATCTATAGCTCAAGTGAAGTGACCAAAGAAGCAAGACTAAGCGCAAACCTTACTTTAACAAGTTCCTCTAACAAGCAAGTCTTTTAACAAGCAAGTCTTTCTCCATACCTCTCCTTTCCCTGCCCGATAATCAGACTGATCATGCCCTGATGACTGATGATATTAAGCCAAGGGGCAGGTACGAGAAATCAAACTTTCTTCCATTAGCAGCTCTAGTAGTAATTGCTTTATTCCCTTGCTTAAAAGGTCCCTTCCCACGGATTGGCCTGCTGCTCCTTCTGTGGCACTAAGACTTGCTGCTTTCATTCTGTCTCCTTCCCCTACAGTAACTGAAGTGGAGCTAGGGCAACATTAAGATAGTATATAGCATCCGTTTGTGACCCCAACATTTCCTCTTCTCGCTTAGAAAGAGCGTCTCCTTCCTCGGACTATTCTATCCTATGGTCTTGCTACTCTTCCTACCTTTCCCTATCTGTCGACTCACTTGTCACTTCCTTCCTACCGCTTAGCCTTAGAGAAAGAATTCTCTCTATCTAATACGTTTACGCTATGGTAAGAAATAGAAAGACTATACGTTGCAGGTGCTGGTTCGGAAGAAAGGTACGGCTTTTCCATAAAGTAGCAAGACTAAGCTTTGCTTTTGAATATCATAGATTAGAATTGAATTTGGAAGTGGACTTGTAGCAAGCAATTGAATACCATACCTTTACAGTCGAGAACTTAGTGCCTTTTCCTCTCTTCAAGTGAAGTGGATTAAGTTGAAGTTGACTTTCTTCCCTACGAAAGTCAAGGTTTTGCTCGCTGCTATCGCGTCTTTAGTTGCAGTCTTAGTCTTAAAGGTACTCAGTCCTTCCTTGGGACTTTCATTCCAAATCCCTATTTCTCGAGTAATTCATACAAGAACAAGCAAACAAACTAAACTATATAGTATAGCATTTCACACTAAGATTTTCTTACCTTAGCTCATGGGCTAAATTAGAACCTTAGTATTTCACTATTCGACTACCAAGATTTTTGGTTGAACCAATAGCAGGAGATGGAAAGAAAGAGAACTTCGTAACTGAATGAATGATTATTCTGATTCTCCTCGCTCTGTAACTAGATCCGTAGCTCTAGAAAGATAGTTGTTGTTTTCCCCAGTTAGGCAGTAGCTCTCGGCTTGTTCCTTGACTAGGTCTATGATCTCCGGTCTGCCTCTGGCCTAGATCAACCTAAGTTAAATGAAGTCTCTATCGTTCTGATTACAAAATGAAATATGAAACTTCATACACCTTAAAGTTCATAGGATGATGAATCGAAAAGGAGAATATTAGTATGCACTCAGCGATCTCTTTGACCACTACTCACTGATTAGCTGGGAATCTTTTGCTTCTTGCCAGTTAGCTCTCCAGATAGCGAAGACAGTTACTGATTCCATTCTTTCGTAGGGGCTGGCTCATGGCAATACGTTCTTTAAGTAGAGTCTCTTTCATCTAGGTCTCAGTAGACGGAATTAGTCTGGTAAAACTCTTGTGGCTAGCTCTTGGTCTGGGGAAGCGGTGTACTCTCTAGGTAGAAAGAAGACGGTCTTAGATAAGGAGTTTTAACCGTAAGCCGTACAGGAGAGGTCAACAGTGGCAACGAGACAAAACGACGAAGTAAGTCGAGCTAAAGGACTTAACCTAAAGATCGATATGAGATAAAGATTTAATAAAGACATGATTTCACCCTTTAAGGCATAAGGCAAGTATACTTTCTAAAATAAAGGAAAACAAGACCGAACGATCTAATAAAGATTGAATAAAGAAAGAATGCAACAATAGGAACAAAGCTGCTCAGCCATCCAAACATTGACAGTCTAAAAGCAGGGAATGCGCTTCGATATAATAAGAAGGGGGACCATCGACTTAGCCACACTCGACTCCCAACGGCATAGAAAGAAAGGCGCTTCTAAGGTTCGTGACTCATTCGGTTTGTTTACAAGGGGCGCCCAAGATGCATGCTTGCCTGGACGATTGGAATTTGTATTCGAGACTTCGATTTACAC

>Decalepis_hamiltonii_chloroplast_contig20

GTAATAGTTCGAGTAAGTCTTGAATGGGCCCCCTGAAAGCTTGATGCAAATAAACGAATTTTTGTTCTACGTCTACGAGCTATATATCTCGTCTAATTCTAGTCATTGAATAGATGAAACTTTCACGAATAACTAATTTCTTCTTTCTTTCAGTTATTCTTTTACCTTTTCCTAATCTATTTAACAAAACGGATTTTTCCAATGTATAAAATCAAAATTCCAATGGCTTTGGCTACTATAACCTTCCTGACCGCGATTTTTCTTTTTTTAGGCATTTCAATGCGAAATAAGAAATTTATTGTGTTATAGGTGTCAAAATAGAAAATATAAATGAATAAAGAAATAGCGGGTTCCTTCCTCTATGGTGACTCCTAAACGGTGAGGTCTTCTCTATACACCGGAGCCTTTACTTCATTTAATCAACGTTATTGGTAACTTGTATAGTTCACCCTTTTGGCTCTACCCATGAATTATCCAGCAATAGGCCTTTCACAATGAGATCTACCTATACAGTAACGGTATTTAATTATGAAAGTTAGCTGGGTAGCTGACCCTCTTAGTCCGTTCTTGCCAGAGTAGGAGCTTAATCTTTATGCTCCTTTAAGATTTCCTCCGCTTGGCTAAACTTTTGCTACCAATGGAGAATTGCTTCTCATCAACAATTGAGGTGATTGGATTTGCACCAACGGAAACCATAAAATTTATACACAATGTAGGAATGTGATAACTTTGATTATTTTTATATGGTTATATAGTGAATGGAATCCTTCTTACATTCTATACTATTCACCGGTACTGATCATCGATACTGGAAAGTTTTTCTTGCTTTTGTACCAGTTCATGGTATGATCTAAACGAGTCGCACATACACCTGAGTACATCTTCCTCGACGTTGAGGGCATCCCTGAAGAGCGGGGATTTCGTGACATTTCTGATTGGCTGTCTTGTATTTCTAATAAGTTGTTGTATGGTTGGCATACTGAATTGTATACATAATGGGCTGGTTTAGATTGATCCTAACCGGATAATTATGAATTACTTCCATTTATTAGATTTATTAGAATAGTCAAATCATAGATAAAATCTCAAATCACGGATTTCACACAAATCAAGGAAGGGGGTGCCATTGATCCTTGGTACGACGAAGCGAAGAGATGATTGACGAGAAGCTTGAGTGACTTGTTAACGATTTGCTGTAGTTTACTCTTTGACTTTTTTGTAAATTGCTCAGTGGGAGGAGCGCTTTCCGTAAGTTCAGCTGAGCAAGGAATGCTTAATGGCGAATACCGATAACATAAGACCTAAGACCTCTCTCTCTCCAATAAGCCACTGCAATCTTTGGCAAAGCATTGGCCCTAGCCTTCCCGATTCTAATCTAGAATAGAGATCGGATCCTTTCACGCCATAGCACTCTTTTCCACTCGGTTTAGGAAAACCCCTTGATTCAGACAGCAGAGGGAATCCTGTTCCTATCGTAAAAGATCAGTCTTGCTTCTTGGGATGAGTGAATTCACCTAAGAGAAGGTAATGCAGACATGGTGTCTTTGGTCAGCGGTACATAGACTTTGAGTCCTAACCAATTACTAGAGTAATTGCCTAGGATTAGGTAGGCAATTACTCTAGTGCGAACACCGAGGTGTCTGTTAAGGGAGTCAAGCTCTGTTCCCGGTGCGAATGCATTATGCCCGGTTGAATCAGTTACACCAGCTAATTTGAACTCAGAGTGGCATACCCTTCCTTGGGTCTACCCTTTCTACATACGTAGGTTCGGTCCTCTCATCACATGAAGGATATTTTTTACCATCTCCTGGATTTCCAGGAGACAGTGTCGTAAAGTAGACATGGTGAGTCGCTCGTTCGTTAAGTTGAGAAATTCAAATGTTACAACATAGCATGGGATTCGAACCCAATTCCGCCAAAACCCCTGATAAGAAAAAAGCAAGAATGATATCAGCTCCGGAGCACCCGCCCGTATAGTAGACATCGGTACAATTTTAGGATGTTATAACTCATAAGGCAATGAGAGGAGTACTCTTAAGAACCATCTCGCTCGCTGGTCTTTTCGATCGTATTATCGACCACGATCGAATCCGCCCGCATTCGGGATCGGGCAGAGGCCAACAATCAGTAAGGGCAATAGCATAGGGTATTATTGACCTGACCCCTATTACTTAAGCCTACTTTCTTCAAGATACGAAATGAGCAGCCGTTTCCGGCTGTCCCTATTGTATTTTAAATGGAGTTCATCAACAGGGCTAAGAATCTTACCTTTACTTAGAGAGGGGCAGCGGTACCACTATATTATGGCGAAGTTGTTGACTCCCTATGCATCCCGACTAAGGTCTCACAAAGGTGCACTTCCCTATGGATCCAGCCGCTTCACTAATGTGAATAGGTTAATAAGAAGGTGGATTGGTTATATACTCTTATGCGCGTTCCTTCTTCGAACCTTTTGGTATGTAGTATTGCCCCTTACTATGGCTCAGATCCACCAGACGAGAAAGTCAATTCCGCACTGCTGCTGAGTCGTCGGACTTATCCACCAAGGGATTCGTGGAATTTCTGTGGATTGCGTTGGGGAAATCTACCAAAGCTATAGGCAGATAGATA

>Decalepis_hamiltonii_chloroplast_contig21

TTGATGCAAATAAACGAATTTTTGTTCTACGTCTACGAGCTATATATCCTCGTCTAATTTAGTCATTGAATAGATGAAACTTTCACGAATAACTAATTTCTTCTTTCTTTCAGTTATTCTTTTACCTTTTCTAATCTATTAATAACAAAACGGATTTTTCCAATGTATAAAATCAAAATTCCAATGGCTTTGGCTACTATAACCTTCCTGACCGCGATTTTTTCTTTTTTAGGCATTTCAATGCGAAATAAGAAATTTATTGTGTTATAGGTGTCAAAATAGAAAATAGAAATGGATAAAGAAATAGCGGGTTCCTTCCTTTCTATGGTGACTTCCTAAACGGTGAGGTCTTCTCTATACACCGGAGCCTTTACTTCATTTAATCAACGTTATTGGTAACTTGTATAGTTCACCCTTTTGGCTCTACCCATGAATTATCCAGCAATAGGCCTTTCACAATGAGATCTACCTATACAGTAACGGTATTTAATTATGAAAGTTAGCTGGGTAGCTGACCCTCTTAGTCCGTTCTTGCCAGAGTAGGAGCTTAATCTTTATGCTCCTTTAAGATTTCCTCCGCTTAATGGCTAACCTTTTGCTACCAATGGAGAATTGCTTCTCATCAACAATTGAGGTGATTGGATTTGCACCAACGGAAACCATAAAATTTATACACAATGTAGGAATGTGATAACTTTGATTATTTTTATATGGTTATATAGTGAATGGAATCCTTCTTACATTCTATACTATTCACCGGTACTGATCATCGATACTGGAAAGTTTTTCTTGCTTTTGTACCAGTTCATGGTATGATCTAAACGAGTCGCACATACACCCGAGTACATCTTCCTCGACGTTGAGGGCATCCCCGAAGAGCGGGGATTTCGTGACATTTCTGATTGGCTGTCTTGTATTTCTAATAAGTTGTTGTATGGTTGGCATACTGAATTGTATACATAATGGGCTGGTTTAGATTGATCCTAACCGGATAATTATGAATTACTTCCATTTATTAGATTTATTAGAATAGTCAAATCATAGATAAAATCTCAAATCACGGATTTCACACAAATCAAGGAAGGGGGTGCCATTGATCCTTGGTACGACGAAGCGAAGAGATGATTGACGAGAAGCTTGAGTGACTTGTTAACGATTTGCTGTAGTTTACTCTTTGACTTTTTTGTAAATTGCTCAGTGGGAGGAGCGCTTTCCGTAAGTTCAGCTGAGCAAGGAATGCTTAATGGCGAATACCGATAACATAAGACCTAAGACCTCTCTCCTCTGCCAATAAGCCACTGCAATCTTTGGCAAAGCATTGGCCCTAGCCTTCCCCGATTCTAATCTAGAATAGAGATCGGATCCTTTCACGCCATAGCACTCTTTTCCACTCGGTTTAGGAAAACCCCTTGATTCAGACAGCAGAGGGAATCCTGTTCCTATCGTAAAAGATCAGTCTTGCTTCTTGGGATGAGTGAATTCACCTAAGAGAAGGTAATGCAGACATGGTGTCTTTGGTCAGCGGTACATAGACTTTGAGTCCTAAATCAATTACTAGAGTAATTGCCTAGGGATTAGGTAGGCAATTACTCTAGTGCGAACACCGAGGTGTCTGTTAAGGGAGTCAAGCTCTGTTCCCGGTGCGAATGCATTATGCCCGGTTGAATCAGTTACACCAGCTAATTTGAACTCAGAGTGGCATACCCTTCCTTGGGTCTACCCTTTTCTACATACGTAGGTTCGGTCCTCTCATCACATGAAGGATATTTTTACCATCTCCTGGATTTCCAGGATAAGACAGTGTCGTAAAGTAGACATGGTGAGTCGCTCGTTCGTTAAGTTGAGAAATTCAAATGTTACAACATAGCATGGGATTCGAACCCAATTCCGCCAAAACCCCTGATAAGAAAAAGCAAGAATGATATGACAAAGAAATAAGCTTTTCATTTCTCCGGAGCACCCGCCCGTATAGTAGACATCGGTACAATTTTAGGATGTTATAACTCATAAGGCAATGAGAGGAGTACTCTTAAGAACCATCTCGCTCGCTGGTCTTTTCGATCGTATTATCGACCACGATCGAATCCCGCCCGCATTCGGGATCGGGCAGAGGCCAACAATCAGTAAGGGCAATAGCATAGGGTATTATTGACCTGACCCCTATTACTTAAGCCTACTTTCTTCAAGATACGAAATGAGCAGCCGTTTCCGGCTGTCCCTATTGTATTTTAAATGGAGTTCATCAACAGGGCTAAGAATCTTACCTTTACTTAGAGAGGGGCAGCGGTACCACTATATTATGGCGAAGTTGTTGACTCCCTATGCATCCCGACTAAGGTCTCACAAAGGTGCACTTCCCTATGGATCCAGCCGCTTCACTAATGTGAATAGGTTAATAAGAAGGGTGGATTGGTTATATACTCTTATGCGCGTTCCTTCTTCGAACCTTTTGGTATGTAGTATTGCCCCCTTACTATGGCTCAGATCCACCAGACGAGAAAGTCAATTCCGCACTGCTGCTGAGTCGTCGGACTTATCCACCAAGGGATTCGTGGAATTTCTGTGGATTGCGTTGGGGAAATCTACCAAAGCTATAGGCAGATAGATAGACTCCTTTCCCGCTGCTAAGGGAAAGCAAGAAAGAAAATCCAATTCTTGTTTTTAACCAGCGCCCCTTTTTGGGTATGCAGGTACTAAGAGTCCTCTCACTACCAACCAGCAGACATCATCTGGCTGGGTCTTGCCGGTATCAACAACGAGAAAGAAACAACCAAATGGAAACAGCAACTAACTATGGCTCTTGGCCCAAACAACGTCCTAGGCGTAGGGGAGATCGGAGCAAGAGGGAAGGCCTACACGACGTTTTTATTCCTGGGCTGTAGTAAGTAGATCGAGAGGTCGTTCCGCCCCTTGTCCCCTAATTTGGGTAATATTTTCTCTTTGAATCTTGCTCCAATCTGACCTAGCTGGCGAGCGATCCCAGTTCGCGACAGAGAACAAGACAGCAAGCGAGCGTACCTTTGTTCGCTTCTTCTCAACCAAGCACGGAAGTTTAAGGATAACTGTAGGTCGGCGGCTACCTAAGGAAACTCCGATTTGATCCGCCCCCGGCTGGGAGGCATCTATCTCATCCCGATCACAAGGAGAGGTTCACTATGATGGGGGTACTTATTTTGCCCTTCCGGAAAGAATGAAATGCATAGGGGACCATCCTTTTGTTGCGGCATGCTCCTCAGATTTACGGATTCGAAGGGGCCTCAGGCCCTACTTAGTTGACTGACAATGACAAAGGCTTGCGAAACTAAGTAGGGCCTTTTCCTTTTGTTTGAATAACCCATTCTCATTATCTTTCATAAGTCAAGTAGGCAAACCTATAGGTCCTTAGTAGCGTTGACAAAGAAAGAAGAAGAGTCGGTTAAAAGACAGCGAATCTTTTTATTTATATTATATCTCTTTTGATTCTTAATTATATTCTAGGCCAGCTTGACAAGCAACCCTTCCTCTTGATCTGAGGTGCGAAGAGAAAGATCTCTTTTATGACTTCACTTCCACTTATCGATCCCGGCCCAGAAAAGTGACCGACCAGGGCCCTATTGATGAATGAAAGAAAGGCTTTATGAGCCCTAGCCCTGTAAGCCCACACCTGTGTAGAGTATATCGTTCAACACCTGCGGCACAAACCCATTTTGACCTTTTGGTCCTAGTAGCATAGGCGACCGAACGGCCGCCCCTAATTACTAGACCAACCAGCTATAATAATTCCATAAACACCTAGCGAAGATATGGCAAACAAATAAAGTAGCCCTATGTTCGGATCTGACAATACCATACCATAATCAAAAGGTACAACGGCCCAAGCGACCAGACTTAACATAAATGTAGTCACTGGAGCCATTCTAAAAAGGGAGAAATTAGCACTACTTGGTGAAATAGGTTCTTTTAGAATCAATTTCAAACCATCTGCTATAGGTTGTAACAATCAAACGATCCCACTACATCAGGACCCTTTCGACGTTGCACAAAAGCCATTACTTTACGTTCAGCTAGCACTAAAAGGCTACTCTAGTAGAAGTGGTAGAATTATTCCAAGTATTTCAGCTGGAACAGCTATGTACATTTTGATTTTCTATTTACTCATGATCTGGCCTGGTCGACCCAATCATGATATTGAAGGATGGGACCTTTTCTCGAAAAACTCCGGATCTCGATAAGAGTTGAAGATGGTGCAACATCGAATCCTGTTACCTTACTTCGAATGGAATCTTAGCCCGCCTCACTTTCTTTACTGCATAAGGGCATAAGCGAAGTCAAGGGATTTCCGTCTAACTAGTGGCTGAGAGTAAGCAAGCTACCTTCATTCAAAAGATTTGTGGTTGAGTTAATAACATGCTATAGGTCGGGAATCTTTGATCTTCTCTTTTGAATTTCCGCTGCCTGCCTTATTTTTCGTGTCCGTCCGTATCGCAAAGCTGGTCGACGCCAGCTGTAATGGTTGAAAATTGGGGCCCACCAAGACCCCTCCCTAAGAAAATCAAGCTTTTTCGATAAAGCAAACAATTCGTTCCGACAACTTCGGACCCCTTTGAATTAGCCGATCTTACAAGATCGATCGGGCGGCTGGTTGGGAAGGGGTGATTAGCGGAGAAAAGAATTGCTGAGAGAGATTCTACTATTTGGTCAGGACGAATGAGTGGCTGTGAAGCATGCTCCTCCGGAGATTGACCCTTCCCGAGTCAGTCCAGTCAGAAAGGGGCCCCCTGTCTAGACTGCATTTCGGGAGTTTGAACACCATCCCATTTCCACCAAAATACAACCCTGTCAAAGACCTTAACGGCCTTCCTTCTTTATGAGTGGAAATCCAATCCCATTTTGTCTTTTTTTGCATAATGCATAATAAAAGAAGCCGGCCGGTTATGTTATATATAAATAATATATATATATATATTATTTTCCTTTCCGAAAGCCTAGAAGAAGGAGGACAAAGGAGTAGTCAGAATGGTTTTCCTTCCTCGGTAAAGTCACTAACTCGATTTAGATCGCCTCTAGGTCCCAATAGACTGAATTAGTCCTTCTACCCCGCCACTCGCTATCCTCTCAAGCATCATCGGGATAGAGCTTCCGGACGCAGACTCCCGAGAAATCTCTTCCAACTCTTTTCGTTCGTTCCTATGAGCCAGAGATCTATCGAAGCCATGAATCCTTCCCTTTGCTGGGCGCTCTCAAACTATATATGCAATGGCACATAAAGCACTTTTTATACTAGATCGAATCATATCCTCTAGTCTCACTCGCTGGTAACCTGGTAACGAGCCACTGGCTGTCAGCAAAGTCTTGAATTGCCTACATCCCATTCCGAAACTAGCAACAATTCCAATTTCACCTGGAAATGCCCCAGCGCGAAGTCGAATCGATTCTTTCTTTAAAGCAACTCTTTTCTTATATACGATACCACCATCTGGCTTGGAGCACGCGAAATGCTTACTTGCTCTCATGTAGCAAAGATAGTTAGCACTACCTTATTCGCTTACCAGAGAGCTCACCTCTAAGAGTAAGAAATCTTTATTAAACACGGATGAGTACTTTCATCAGGAACGGAACTAGTAATAGACAGCAATGCTAGCACTATCAGCGGAATCAAAGAGGAAATGCAGAAAGACTTCTAGCTTATCAGCAAGCAGAAGTAAGAAACTTTCCAACAGAAGAAAGAGTACCCTGCTACAACACAGTCTTCGATGCTTTGAATAGCGTAGTAAAGTAGCCAGTCACTAGGAGTAGTAGGGCTATCGCTTACTCTTTCCCAGTTCCTAGGGCTAGGGAACCGGTCTGCTGTCATACATTTTTTTTCTAGAAAGAAAGCCGACAAAGCAGATGACAGCCCGATAGCGGAACCTAAATCCTCTAGTAATTGATCTGGATTTTCCACCCTATCCCCAGTTCTAATAAATGAAGAAGGCTCGGAGGCTAAAGAAGACTCGGGCTATATTAATATATAAGGCAACGGAGCCATCATAGTCGTTTTTAACTATTCCACAAGAAAGTGCTACACTCTTCGTCGCTAGTAGCAAGGGATTCCACTGCTTAACTTAAGTTAAGGCCCCGTAGCTTTATTGCCTCTTTCCTTCCCTGCGGGAGAGCTGAAACCTTGGCCGCAGATCCTAATCCTACGTACTGATGGCTCTAGCCCGCCTCTCCTCTAATCCAGTGACCAGTGGAGGTCTAGTTGCTTTCTCCTCGCTAGAAGAGCCACGGGGTCTTTTGACCATAAATCTAGAGTGAAGCACTCGTCTGACTTCACTTAGTGTTAGTAGCGGGAATGGAGAGGCAGTCTGTGGTGAATTGGAGACAGGCTACGAGAAAGAGAGTGATCGGTTGACGACGAGAAAGTCAAAATAGATGGGCCCAATCGGGCCAAAGTCCAATCCTCCAATGTGCCGGGTGTTCCGACTCCTAACTACCTTTACCTTACCTTCTATTTCTTTCTTTTCACTCTCCCCGCTGCCGCCCAAGCAATGGGACTGAATCTGCTATAAGGGAAATGTGAGGCCGATCTCTGTCTTGATTTGATCCCAATAAGTTAGAATGAATGCGACCTCAATCGGGCATTCGGGTGCTTCCAAGGAAGACGATCTCATCTTTGGTTTGGATGGGAGGAATCCTCGAAGTGGTCCCTCATCTCGCTCTTTGGGATGCGGGGCGGGCTCCGTACCTTCCGGCAAAGTGGCTCTTCTCCGGAACCTTGCAGGGCATGCTATCAGCTTCAAAAGGACCAGTGCTGGCATCAGCCTTTGCCCGACCTTCTCGTTTTATCCTATGGGCGAGCGTTCCAACTCCTCGTAGCGTAGAAAGAGGATAGTCGTCCCTGAGCCTTTCCACTCTGGCCGTCGAATGCTTTCCCCACTCAAGGGAGCTAGTCTCTCTGGCGTATAAAAGAAAGGAAGTGGAAAGTCATGAGCTTCTCCTTCTTCTTTTCGTTCTAGTCTTGTCTTGAGGAGAGAATGTGGTGAGAAGAAAGGATAAACTGTCCTTCCGAGAAATCCACTTTGCTTTAGCTTCCTCACCGTTCTATGGCTTGGGAAAGGGCATCCTTGCTCTTTTAGTTGGGAGAGTGCATTTCAGTCCGTTGGCCGCAGACCTCTCAAGGATTTAAACCCGAATTCTCAGCTCCTCATTACTAGCTTTGAGTCCCTGCTCCTCTACTTCTTCTAAAGATTCAAATAGAATCCCTGCACAAGCTTTTACCTCTGAAGATAAGGTAAGAGTAGCTGCACTTAACACATACCCAATCTTTTCCTAAATCCTATGGATTCTTCTTTCCGGCTTCGCCTGTCGCTTCGTTCTAGGCTTTAGCCAGTTCCAAAGACTTAATATATCTAACTACTAGCTAGTACAGTAAGGACTAGTCCGTGGGATGGACAAGGAAGTGTATCGATAGAAGCGTAGGATAGATAGATAAGAATGTGACTTTCGCCCCTTGGGGACAAGAGCAAGCTCAAGAAAAGCATCCTTTCGAGGACTTGATTTTCTTTGTAAAAAATACCTATTGTTGTCTATCTCACTAATGATTCTAAGCGCATTTATTTGCCCTCGGCTCGGACTCATAAGCTTGCTCCAAAGAGAAGAGAAAGCCAAAGCGGATGGCGGGTGCCGATGTCTTGAGTGGTGAACTACAAGTGAAGCCATCCAGGATTGAAGGTTGAGGACGAACTACTTTAGTTAAGTCTTTTTGGCAGACAATAACTAGGCCAGGGTCAATCTCAAGATCACGAGCGTTCCGCTTCCGGACCTAAAACTCTTGCTTTTGGGAAGTTTGTAGGGCTTTTACCGTGCTCTCATTTTATGATAAAGGATCAGAGTAAGAAGAAGAGAACAAAGGGAATGAAAAGACCTGATCAAATAAAACGAAAGTAGCACAAGCTATCAAAACATTAGAAGTTGGAATGTTTAACATACTATGATAAATAATGCGAAATTCAAATTTCCTAGGTCTAACAGTTTTCAAGGCAAGGAAAATTGACTTTCTTTCGAAACCCGTGCTTGAGGAATTTTATTGGGGTAGAAATCAACATAAAGTGAAGTTGCCTCTGCCGCTGCACAAGAATAAGCTCGTGCCGGTGTGATCTTCTACAATGAAGATACCTGACGAACGAGAA

*Hemidesmus indicus* chloroplast genome contigs:

>Hemidesmus_indicus_chloroplast_contig1

CTTCGTTCACGTACTGACGGTGTTTTTTTGGTACCTTTTTTCACCGGAAAGGACCCGTAAAGTGATAATGATTATCATCTACATATCACAACGTGCGTGGAGGCCATCAAACCACGTCAAATAATCAATTATGACGCAGGTATCGTATTAATTGATCTGCATCAACTTAACGTAAAAACAACTTCAGACAATACAAATCAGCGACACTGAATACGGGGCAACCTCATGTCAACGAAGAACAGAACCCGCAGAACAACAACCCGCAACATCCGCTTTCCTAACCAAATGATTGAACAAATTAACATCGCTCTTGAGCAAAAAGGGTCCGGGAATTTCTCAGCCTGGGTCATTGAAGCCTGCCGTCGGAGACTAACGTCAGAAAAGAGCATATACATCAATTAAAAGTGATGAAGAATGAACATCCGCGTTCTTCCCTCCGAACAGGACGATATTGTAAATTCACTTAATTACGAGGGCATTGCAGTAATTGAGTTGCAGTTTTACCACTTTCCTGACAGTGACAGACTGCGTGTTGGCTCTGTCACAGACTAAATAGTTTGAATGATTAGCAGTTATGGTGATCAGTCAACCACCAGGAATAATCCTTCATATTATTATCGTGCTTCACCAACGCTGCCTCAATTGCTCTGAATGCTTCCAGAGACACCTTATGTTCTATACATGCAATTACAACATCAGGGTAACTCATAGAAATGGTGCTATTAAGCATATTTTTTACACGAATCAGATCCACGGAGGGATCATCAGCAGATTGTTCTTTATTCATTTTGTCGCTCCATGCGCTTGCTCTTCATCTAGCGGTTAAAATATTACTTCAAATCTTTCTGTATGAAGATTTGAGCACGTTAACCTTACATACATCTGTCGGTTGTATTTCCCTCCAGAATGCCAGCAGGACCGCACTTTGTTACGCAACCAATACTATTAAGTGAAAACATTCCTAATATTTGACATAAATCATCAACAAAACACAAGGAGGTCAGACCAGATTGAAACGATAAAAACGATAATGCAAACTACGCGCCCTCGTATCACATGGAAGGTTTTACCAATGGCTCAGGTTGCCATTTTTAAAGAAATATTCGATCAAGTGCGAAAAGATTTAGACTGTGAATTGTTTTATTCTGAACTAAAACGTCACAACGTCTCACATTATATTTACTATCTAGCCACAGATAATATTCACATCGTGTTAGAAAACGATAACACCGTGTTAATAAAAGGACTTAAAAGGTTGTAAATGTTAAATTCTCAAGAAACACGCATCTTATAGAAACGTCCTATGATAGGTTGAAATCAAGAGAAATCACATTTCAGCAATACAGGGAAAATCTTGCTAAAGCAGGAGTTTTCCGATGGGTTACAAATATCCATGAACATAAAAGATATTACTATACCTTTGATAATTCATTACTATTTACTGAGAGCATTCAGAACACTACACAAATCTTTCCACGCTAAATCATAACGTCCGGTTTCTTCCGTGTCAGCACCGGGGCGTTGGCATAATGCAATACGTGTACGCGCTAAACCCTGTGTGCATCGTTTTAATTATTCCCGGACACTCCCGCAGAGAAGTTCCCCGTCAGGGCTGTGGACATAGTTAATCCGGGAATACAATGACGATTCATCGCACCTGACATACATTAATAAATATTAACAATATGAAATTTCAACTCATTGTTTAGGGTTTGTTTAATTTTCTACACATACGATTCTGCGAACTTCAAAAGCATCGGGAATAACACCATGAAAAAATGCTACTCGCTACTGCGCTGGCCCTGCTTATTACAGGATGTGCTCAACAGACGTTTACTGTTCAAAACAAACCGGCAGCAGTAGCACCAAAGGAAACCATCACCCATCATTTCTTCGTTTCTGGAATTGGGCAGAAGAAAACTGTCGATGCAGCCAAAATTTGTGGCGGCGCAGAAAATGTTGTTAAAACAGAAACCCAGCAAACATTCGTAAATGGATTGCTCGGTTTTATTACTTTAGGCATTTATACTCCGCTGGAAGCGTGTGTATTGCTCACAATAATTGCATGAGTTGCCCATCGATATGGGCAACTCTATCTACACTGCTCATTAATATACTTCTGGGTTCCTTCCAGTTGTTTTTGCATAGTGATCAGCCTCTCTCTGAGGGTGAAATAATCCCGTTCAGCGGTGTCTGCCAGTCGGGGAGGCTGCATTATCCACGCCGGAGGCGGTGGTGGCTTCACGCACTGACTGACAGACTGCTTTGATGTGCAACCGACGACGACCAGCGGCAACATCATCACGCAGAGCATCATTTTCAGCTTTAGCATCAGCTAACTCCTTCGTGTATTTTGCATCGAGCGCAGCAACATCACGCTGACGCATCTGCATGTCAGTAATTGCCGCGTTCGCCAGCTTCAGTTCTCTGGCATTTTTGTCGCGCTGGGCTTTGTAGGTAATGGCGTTATCACGGTAATGATTAACAGCCCATGACAGGCAGACGATGATGCAGATAACCAGAGCGGAGATAATCGCGGTGACTCTGCTCATACATCAATCTCTCTGACCGTTCCGCCCGCTTCTTTGAATTTTGCAATCAGGCTGTCAGCCTTATGCTCGAACTGACCATAACCAGCGCCCGGCAGTGAAGCCCAGATATTGCTGCAACGGTCGATTGCCTGACGGATATCACCACGATCAATCATAGGTAAAGCGCCACGCTCCTTAATCTGCTGCAATGCCACAGCGTCCTGACTTTTCGGAGAGAAGTCTTTCAGGCCAAGCTGCTTGCGGTAGGCATCCCACCAACGGGAAAGAAGCTGGTAGCGTCCGGCGCCTGTTGATTTGAGTTTTGGGTTTAGCGTGACAAGTTTGCGAGGGTGATCGGGTAATCAGTAAATAGCTCTCCGCCTACAATGACGTCATAACCATGATTTCTGGTTTTCTGACGTCCGTTATCAGTTCCCTCCGACCACGCCAGCATATCGAGGAACGCCTTACGTTGATTATTGATTTCTACCATCTTCTACTCCGGCTTTTTAGCAGCGAAGCGTTTGATAAGCGAACCAATCGAGTCAGTACCGATGTAGCCGATAAACACGCTCGTTATATAAGCGAGATTGCTACTTAGTCCGGCGAAGTCGAGAAGGTCACGAATGAACTAGGCGATAATGGCGCACATCGTTGCGTCGATTACTGTTTTGTAAACGCACCGCCATTATATCTGCCGCGAAGGTACGCCATTGCAAACGCAAGGATTGCCCCGATGCCTTGTTCCTTTGCCGCGAGAATGGCGGCCAACAGGTCATGTTTTTCTGGCATCTTCATGTCTTACCCCAATAAGGGATTTGCTCTATTTAATTAGGAATAAGGTCGATTACTGATAGAACAAATCCAGGCTACTGTGTTTAGTAATCAGATTTGTTCGTGACCGATATGCACGGGCAAAACGGCAGGAGGTTGTTAGCGCAAAAAAAATTCAAAACTTCGTTCAGTTACGTATTGCTGCTTACGGTTCACTACTCACGACGATGTTTTTTGGTACCTTTTTTCACCGGAAAGGACCCGTAAAGTGATAATGATTATCATCTACATATCACAACGTGCGTGGAGGCCATCAAACCACGTCAAATAATCAATTATGACGCAGGTATCGTATTAATTGATCTGCATCAACTTAACGTAAAAACAACTTCAGACAATACAAATCAGCGACACTGAATACGGGGCAACCTCATGTCAACGAAGAACAGAACCCGCAGAACAACAACCCGCAACATCCGCTTTCCTAACCAAATGATTGAACAAATTAACATCGCTCTTGAGCAAAAAGGGTCCGGGAATTTCTCAGCCTGGGTCATTGAAGCCTGCCGTCGGAGACTAACGTCAGAAAAGAGAGCATATACATCAATTAAAAGTGATGAAGAATGAACATCCGCGTTCTTCCCTCCGAACAGGACGATATTGTAAATTCACTTAATTACGAGGGCATTGCAGTAATTGAGTTGCAGTTTTACCACTTTCCTGACAGTGACAGACTGCGTGTTGGCTCTGTCACAGACTAAATAGTTTGAATGATTAGCAGTTATGGTGATCAGTCAACCACCAGGAATAATCCTTCATATTATTATCGTGCTTCACCAACGCTGCCTCAATTGCTCTGAATGCTTCCAGAGACACCTTATGTTCTATACATGCAATTACAACATCAGGGTAACTCATAGAAATGGTGCTATTAAGCATATTTTTACACGAATCAGATCCACGGAGGGATCATCAGCAGATTGTTCTTTATTCATTTTGTCGCTCCATGCGCTTGCTCTTCATCTAGCGGTTAAAATATTACTTCAAATCTTTCTGTATGAAGATTTGAGCACGTTAACCTTACATACATCTGTCGGTTGTATTTCCCTCCAGAATGCCAGCAGGACCGCACTTTGTTACGCAACCAATACTATTAAGTGAAAACATTCCTAATATTTGACATAAATCATCAACAAAACACAAGGAGGTCAGACCAGATTGAAACGATAAAAACGATAATGCAAACTACGCGCCCTCGTATCACATGGAAGGTTTTACCAATGGCTCAGGTTGCCATTTTTAAAGAAATATTCGATCAAGTGCGAAAAGATTTAGACTGTGAATTGTTTTATTCTGAACTAAAACGTCACAACGTCTCACATTATATTTACTATCTAGCCACAGATAATATTCACATCGTGTTAGAAAACGATAACACCGTGTTAATAAAAGGACTTAAAAGGTTGTAAATGTTAAATTCTCAAGAAACACGCATCTTATAGAAACGTCCTATGATAGGTTGAAATCAAGAGAAATCACATTTCAGCAATACAGGGAAAATCTTGCTAAAGCAGGAGTTTTCCGATGGGTTACAAATATCCATGAACATAAAAGATATTACTATACCTTTGATAATTCATTACTATTTACTGAGAGCATTCAGAACACTACACAAATCTTTCCACGCTAAATCATAACGTCCGGTTTCTTCCGTGTCAGCACCGGGGCGTTGGCATAATGCAATACGTGTACGCGCTAAACCCTGTGTGCATCGTTTTAATTATTCCCGGACACTCCCGCAGAGAAGTTCCCCGTCAGGGCTGTGGACATAGTTAATCCGGGAATACAATGACGATTCATCGCACCTGACATACATTAATAAATATTAACAATATGAAATTTCAACTCATTGTTTAGGGTTTGTTTAATTTTCTACACATACGATTCTGCGAACTTCAAAAGCATCGGGAATAACACCATGAAAAAATGCTACTCGCTACTGCGCTGGCCCTGCTTATTACAGGATGTGCTCAACAGACGTTTACTGTTCAAAACAAACCGGCAGCAGTAGCACCAAAGGAAACCATCACCCATCATTTCTTCGTTTCTGGAATTGGGCAGAAGAAAACTGTCGATGCAGCCAAAATTTGTGGCGGCGCAGAAAATGTTGTTAAAACAGAAACCCAGCAAACATTCGTAAATGGATTGCTCGGTTTTATTACTTTAGGCATTTATACTCCGCTGGAAGCGTGTGTATTGCTCACAATAATTGCATGAGTTGCCCATCGATATGGGCAACTCTATCTGCACTGCTCATTAATATACTTCTGGGTTCCTTCCAGTTGTTTTTGCATAGTGATCAGCCTCTCTCTGAGGGTGAAATAATCCCGTTCAGCGGTGTCTGCCAGTCGGGGAGGCTGCATTATCCACGCCGGAGGCGGTGGTGGCTTCACGCACTGACTGACAGACTGCTTTGATGTGCAACCGACGACGACCAGCGGCAACATCATCACGCAGAGCATCATTTTCAGCTTTAGCATCAGCTAACTCCTTCGTGTATTTTGCATCGAGCGCAGCAACATCACGCTGACGCATCTGCATGTCAGTAATTGCCGCGTTCGCCAGCTTCAGTTCTCTGGCATTTTTGTCGCGCTGGGCTTTGTAGGTAATGGCGTTATCACGGTAATGATTAACAGCCCATGACAGGCAGACGATGATGCAGATAACCAGAGCGGAGATAATCGCGGTGACTCTGCTCATACATCAATCTCTCTGACCGTTCCGCCCGCTTCTTTGAATTTTGCAATCAGGCTGTCAGCCTTATGCTCGAACTGACCATAACCAGCGCCCGGCAGTGAAGCCCAGATATTGCTGCAACGGTCGATTGCCTGACGGATATCACCACGATCAATCATAGGTAAAGCGCCACGCTCCTTAATCTGCTGCAATGCCACAGCGTCCTGACTTTTCGGAGAGAAGTCTTTCAGGCCAAGCTGCTTGCGGTAGGCATCCCACCAACGGGAAAGAAGCTGGTAGCGTCCGGCGCCTGTTGATTTGAGTTTTGGGTTTAGCGTGACAAGTTTGCGAGGGTGATCGGGTAATCAGTAAATAGCTCTCCGCCTACAATGACGTCATAACCATGATTTCTGGTTTTCTGACGTCCGTTATCAGTTCCCTCCGACCACGCCAGCATATCGAGGAACGCCTTACGTTGATTATTGATTTCTACCATCTTCTACTCCGGCTTTTTAGCAGCGAAGCGTTTGATAAGCGAACCAATCGAGTCAGTACCGATGTAGCCGATAAACACGCTCGTTATATAAGCGAGATTGCTACTTAGTCCGGCGAAGTCGAGAAGGTCACGAATGAACTAGGCGATAATGGCGCACATCGTTGCGTCGATTACTGTTTTGTAAACGCACCGCCATTATATCTGCCGCGAAGGTACGCCATTGCAAACGCAAGGATTGCCCCGATGCCTTGTTCCTTTGCCGCGAGAATGGCGGCCAACAGGTCATGTTTTTCTGGCATCTTCATGTCTTACCCCCAATAAGGGATTTGCTCTATTTAATTAGGAATAAGGTCGATTACTGATAGAACAAATCCAGGCTACTGTGTTTAGTAATCAGATTTGTTCGTGACCGATATGCACGGGCAAAACGGCAGGAGGTTGTTAGCGCAAAAAAATTCCAAAAAATTCCAAAAAAAAGCGACTAACAAACACAATCTGATGGCTGCTTACGGTTCACTACTCACGACGATGTTTTTTGGTACCTTTTTTCACCGGAAAGGACCCGTAAAGTGATAATGATTATCATCTACATATCACAACGTGCGTGGAGGCCATCAAACCACGTCAAATAATCAATTATGACGCAGGTATCGTATTAATTGATCTGCATCAACTTAACGTAAAAACAACTTCAGACAATACAAATCAGCGACACTGAATACGGGGCAACCTCATGTCAACGAAGAACAGAACCCGCAGAACAACAACCCGCAACATCCGCTTTCCTAACCAAATGATTGAACAAATTAACATCGCTCTTGAGCAAAAAGGGTCCGGGAATTTCTCAGCCTGGGTCATTGAAGCCTGCCGTCGGAGACTAACGTCAGAAAAGAGAGCATATACATCAATTAAAAGTGATGAAGAATGAACATCCCGCGTTCTTCCCTCCGAACAGGACGATATTGTAAATTCACTTAATTACGAGGGCATTGCAGTAATTGAGTTGCAGTTTTACCACTTTCCTGACAGTGACAGACTGCGTGTTGGCTCTGTCACAGACTAAATAGTTTGAATGATTAGCAGTTATGGTGATCAGTCAACCACCAGGAATAATCCTTCATATTATTATCGTGCTTCACCAACGCTGCCTCAATTGCTCTGAATGCTTCCAGAGACACCTTATGTTCTATACATGCAATTACAACATCAGGGTAACTCATAGAAATGGTGCTATTAAGCATATTTTTTACACGAATCAGATCCACGGAGGGATCATCAGCAGATTGTTCTTTATTCATTTTGTCGCTCCATGCGCTTGCTCTTCATCTAGCGGTTAAAATATTACTTCAAATCTTTCTGTATGAAGATTTGAGCACGTTAACCTTACATACATCTGTCGGTTGTATTTCCCTCCAGAATGCCAGCAGGACCGCACTTTGTTACGCAACCAATACTATTAAGTGAAAACATTCCTAATATTTGACATAAATCATCAACAAAACACAAGGAGGTCAGACCAGATTGAAACGATAAAAACGATAATGCAAACTACGCGCCCTCGTATCACATGGAAGGTTTTACCAATGGCTCAGGTTGCCATTTTTAAAGAAATATTCGATCAAGTGCGAAAAGATTTAGACTGTGAATTGTTTTATTCTGAACTAAAACGTCACAACGTCTCACATTATATTTACTATCTAGCCACAGATAATATTCACATCGTGTTAGAAAACGATAACACCGTGTTAATAAAAGGACTTAAAAGGTTGTAAATGTTAAATTCTCAAGAAACACGCATCTTATAGAAACGTCCTATGATAGGTTGAAATCAAGAGAAATCACATTTCAGCAATACAGGGAAAATCTTGCTAAAGCAGGAGTTTTCCGATGGGTTACAAATATCCATGAACATAAAAGATATTACTATACCTTTGATAATTCATTACTATTTACTGAGAGCATTCAGAACACTACACAAATCTTTCCACGCTAAATCATAACGTCCGGTTTCTTCCGTGTCAGCACCGGGGCGTTGGCATAATGCAATACGTGTACGCGCTAAACCCTGTGTGCATCGTTTTAATTATTCCCGGACACTCCCGCAGAGAAGTTCCCCGTCAGGGCTGTGGACATAGTTAATCCGGGAATACAATGACGATTCATCGCACCTGACATACATTAATAAATATTAACAATATGAAATTTCAACTCATTGTTTAGGGTTTGTTTAATTTTCTACACATACGATTCTGCGAACTTCAAAAGCATCGGGAATAACACCATGAAAAAATGCTACTCGCTACTGCGCTGGCCCTGCTTATTACAGGATGTGCTCAACAGACGTTTACTGTTCAAAACAAACCGGCAGCAGTAGCACCAAAGGAAACCATCACCCATCATTTCTTCGTTTCTGGAATTGGGCAGAAGAAAACTGTCGATGCAGCCAAAATTTGTGGCGGCGCAGAAAATGTTGTTAAAACAGAAACCCAGCAAACATTCGTAAATGGATTGCTCGGTTTTATTACTTTAGGCATTTATACTCCGCTGGAAGCGTGTGTATTGCTCACAATAATTGCATGAGTTGCCCATCGATATGGGCAACTCTATCTGCACTGCTCATTAATATACTTCTGGGTTCCTTCCAGTTGTTTTTGCATAGTGATCAGCCTCTCTCTGAGGGTGAAATAATCCCGTTCAGCGGTGTCTGCCAGTCGGGGAGGCTGCATTATCCACGCCGGAGGCGGTGGTGGCTTCACGCACTGACTGACAGACTGCTTTGATGTGCAACCGACGACGACCAGCGGCAACATCATCACGCAGAGCATCATTTTCAGCTTTAGCATCAGCTAACTCCTTCGTGTATTTTGCATCGAGCGCAGCAACATCACGCTGACGCATCTGCATGTCAGTAATTGCCGCGTTCGCCAGCTTCAGTTCTCTGGCATTTTTGTCGCGCTGGGCTTTGTAGGTAATGGCGTTATCACGGTAATGATTAACAGCCCATGACAGGCAGACGATGATGCAGATAACCAGAGCGGAGATAATCGCGGTGACTCTGCTCATACATCAATCTCTCTGACCGTTCCGCCCGCTTCTTTGAATTTTGCAATCAGGCTGTCAGCCTTATGCTCGAACTGACCATAACCAGCGCCCGGCAGTGAAGCCCAGATATTGCTGCAACGGTCGATTGCCTGACGGATATCACCACGATCAATCATAGGTAAAGCGCCACGCTCCTTAATCTGCTGCAATGCCACAGCGTCCTGACTTTTCGGAGAGAAGTCTTTCAGGCCAAGCTGCTTGCGGTAGGCATCCCACCAACGGGAAAGAAGCTGGTAGCGTCCGGCGCCTGTTGATTTGAGTTTTGGGTTTAGCGTGACAAGTTTGCGAGGGTGATCGGGTAATCAGTAAATAGCTCTCCGCCTACAATGACGTCATAACCATGATTTCTGGTTTTCTGACGTCCGTTATCAGTTCCCTCCGACCACGCCAGCATATCGAGGAACGCCTTACGTTGATTATTGATTTCTACCATCTTCTACTCCGGCTTTTTAGCAGCGAAGCGTTTGATAAGCGAACCAATCGAGTCAGTACCGATGTAGCCGATAAACACGCTCGTTATATAAGCGAGATTGCTACTTAGTCCGGCGAAGTCGAGAAGGTCACGAATGAACTAGGCGATAATGGCGCACATCGTTGCGTCGATTACTGTTTTGTAAACGCACCGCCATTATATCTGCCGCGAAGGTACGCCATTGCAAACGCAAGGATTGCCCCGATGCCTTGTTCCTTTGCCGCGAGAATGGCGGCCAACAGGTCATGTTTTCTGGCATCTTCATGTCTTACCCCCAATAAGGGATTTGCTCTATTTAATTAGGAATAAGGTCGATTACTGATAGAACAAATCCAGGCTACTGTGTTTAGTAATCAGATTTGTTCGTGACCGATATGCACGGGCAAAACGGCAGGAGGTTGTTAGCGCAAAAAAAATTCCAAAAAAATTCCAAAAAAAAGCGACTAACAAACACAATCTGATGGCAGCAATACGTAACTGAACGAAGTAC

>Hemidesmus_indicus_chloroplast_contig2

GTAGTTACGTATTGCTGCCATCAGATTGTGTTTGTTAGTCGCTTTTTTTTGGAATTTTTTGGAATTTTTTTTGCGCTAACAACCTCCTGCCGTTTTGCCCGTGCATATCGGTCACGAACAAATCTGATTACTAAACACAGTAGCCTGGATTTGTTCTATCAGTAATCGACCTTATTCCTAATTAAATAGAGCAAATCCTTTATTGGGGGTAAGACATGAAGATGCCAGAAAACATGACCTGTTGGCCGCCATTCTCGCGGCAAAGGAACAAGGCATCGGGGCAATCCTTGCGTTTGCAATGGCGTACCTTCGCGGCAGATATAATGGCGGTGCGTTTACAAAACAGTAATCGACGCAACGATGTGCGCCATTATCGCCTAGTTCATTCGTGACCTTCTCGACTTCGCCGGACTAAGTAGCAATCTCGCTTATATAACGAGCGTGTTTATCGGCTACATCGGTACTGACTCGATTGGTTCGCTTATCAAACGCTTCGCTGCTAAAAAGCCGGAGTAGAAGATGGTAGAAATCAATAATCAACGTAAGGCGTTCCTCGATATGCTGGCGTGGTCGGAGGGAACTGATAACGGACGTCAGAAAACCAGAAATCATGGTTATGACGTCATTGTAGGCGGAGAGCTATTTACTGATTACTTGATCACCCTCGCAAACTTGTCACTGCCAAAACAAACTCAATCAAGATTCGCCTGAAGTCAGGACGCTGTGGCATTGCAGCAGATTAAGGAGCGTGGCGCTTTACCTATGATTGATCGTGGTGATATCCGTCAGGCAATCGACCGTTGCAGCAATATCTGGGCTTCACTGCCGGGCGCTGGTTATGGTCAGTTCGAGCATAAGGCTGACAGCCTGATTGCAAAATTCAAAGAAGCGGGCGGAACGGTCAGAGAGATTGATGTATGAGCAGAGTCACCGCGATTATCTCCGCTCTGGTTATCTGCATCATCGTCTGCCTGTCATGGGCTGTTAATCATTACCGTGATAACGCCATTACCTACAAAGCCCAGCGCGACAAAAATGCCAGAGAACTGAAGCTGGCGAACGCGGCAATTACTGACATGCAGATGCGTCAGCGTGATGTTGCTGCGCTCGATGCAAAATACACGAAGGAGTTAGCTGATGCTAAAGCTGAAAATGATGCTCTGCGTGATGATGTTGCCGCTGGTCGTCGTCGGTTGCACATCAAAGCAGTCTGTCAGTCAGTGCGTGAAGCCACCACGCCTCCGGCGTGGATAATGCAGCCTCCCCGACTGGCAGACACCGCTGAACGGGATTATTTCACCTCAGAGAGAGGCTGATCACTATGCAAAAACAACTGGAAGGAACCCAGAAGTATATTAATGAGCAGTGCAGATAGAGTTGCCCATATCGATGGGCAACTCATGCAATTATTGTGAGCAATACACACAGCTTCCAGCGGAGTATAAATGCCTAAAGTAATAAAACCGAGCAATCCATTTACGAATGTTTGCTGGGTTTCTGTTTTAACAACATTTTCTGCGCCGCCACAAATTTTGGCTGCATCGACAGTTTTCTTCTGCCCAATTCCAGAAACGAAGAAATGATGGGTGATGGTTTCCTTTGGTGCTACTGCTGCCGGTTTGTTTTGAACAGTAAACGTCTGTTGAGCACATCCTGTAATAAGCAGGGCCAGCGCAGTAGCGAGTAGCATTTTTTCATGGTGTTATTCCCGATGCTTTTTGAAGTTCGCAGAATCGTATGTGTAGAAAATTAAACAAACCCTAAACAATGAGTTGAAATTTCATATTGTTAATATTTATTAATGTATGTCAGGTGCGATGAATCGTCATTGTATTCCCGGATTAACTATGTCCACAGCCCTGACGGGGAACTTCTCTGCGGGAGTGTCCGGGAATAATTAAAACGATGCACACAGGGTTTAGCGCGTACACGTATTGCATTATGCCAACGCCCCGGTGCTGACACGGAAGAAACCGGACGTTATGATTTAGCGTGGAAAGATTTGTGTAGTGTTCTGAATGCTCTCAGTAAATAGTAATGAATTATCAAAGGTATAGTAATATCTTTTATGTTCATGGATATTTGTAACCCATCGGAAAACTCCTGCTTTAGCAAGATTTTCCCTGTATTGCTGAAATGTGATTTCTCTTGATTTCAACCTATCATAGGACGTTTCTATAAGATGCGTGTTTCTTGAGAATTTAACATTTACAACCTTTTAAGTCCTTTTATTAACACGGTGTTATCGTTTTCTAACACGATGTGAATATTATCTGTGGCTAGATAGTAAATATAATGTGAGACGTTGTGACGTTTTAGTTCAGAATAAAACAATTCACAGTCTAAATCTTTTCGCACTTGATCGAATATTTCTTTAAAAATGGCAACCTGAGCCATTGGTAAAACCTTCCATGTGATACGAGGGCGCGTAGTTTGCATTATCGTTTTTATCGTTTCAATCTGGTCTGACCTCCTTGTGTTTTGTTGATGATTTATGTCAAATATTAGGAATGTTTTCACTTAATAGTATTGGTTGCGTAACAAAGTGCGGTCCTGCTGGCATTCTGGAGGAAATACAACCAATTTTAACGTGCTAGATGAAGAGCAAGCGCATGGAGCGACAAAATGAATAAAGAACAATCTGCTGATGATCCCTCCGTGGATCTGATTCGTGTAAAAATATGCTTAATAGCACCATTTCTATGAGTTACCCTGATGTTGTAATTGCATGTATAGAACATAAGGTGTCTCTGGAAGCATTCAGAGCAATTGAGGCAGCGTTGGTGAAGCACGATAATAATATGAAGGATTATTCCTGGTGGTTGACTGATCACCATAACTGCTAATCATTCAAACTATTTAGTCTGTGACAGAGCCAACACGCAGTCTGTCACTGTCAGGAAAGTGGTAAAACTGCAACTCAATTACTGCAATGCCCTCGTAATTAAGTGAATTTACAATATCGTCCTGTTCGGAGGGAAGAACGCGGATGTTCATTCTTCATCACTTTTAATTGATGTATATGCTCTCTTTTCTGACGTTAGTCTCCGACGGCAGGCTTCAATGACCCAGGCTGAGAAATTCCCGGACCCTTTTGCTCAAGAGCGATGTTAATTTGTTCAATCATTTGGTTAGGAAAGCGGATGTTGCGGGTTGTTGTTCTGCGGGTTCTGTTCTTCGTTGACATGAGGTTGCCCCGTATTCAGTGTCGCTGATTTGTATTGTCTGAAGTTGTTTTTACGTTAAGTTGATGCAGATCAATTAATACGATACCTGCGTCATAATTGATTATTTGACGTGGTTTGATGGCCTCCACGCACGTTGTGATATGTAGATGATAATCATTATCACTTTACGGGTCCTTTCCGGTGAAAAAAGGATCAGTAACTTCGTTCAGTTACGTATTGCTGCCATCAGATTGTGTTTGTTAGTCGCTTTTTTTTTGGAATTTTTTTTGGAATTTTTTTGCGCTAACAACCTCCTGCCGTTTGCCCGTGCATATCGGTCACGAACAAATCTGATTACTAAACACAGTAGCCTGGATTTGTTCTATCAGTAATCGACCTTATTCCTAATTAAATAGAGCAAATCCCTTATTGGGGTAAGACATGAAGATGCCAGAAAAACATGACCTGTTGGCCGCCATTCTCGCGGCAAAGGAACAAGGCATCAGGGCAATCCTTGCGTTTGCAATGGCGTACCTTCGCGGCAGATATAATGGCGGTGCGTTTACAAAAACAGTAATCGACGCAACGATGTGCGCCATTATCGCCTAGTTCATTCGTGACCTTCTCGACTTCGCCGGACTAAGTAGCAATCTCGCTTATATAACGAGCGTGTTTATCGGCTACATCGGTACTGACTCGATTGGTTCGCTTATCAAACGCTTCGCTGCTAAAAAGCCGGAGTAGAAGATGGTAGAAATCAATAATCAACGTAAGGCGTTCCTCGATATGCTGGCGTGGTCGGAGGGAACTGATAACGGACGTCAGAAAACCAGAAATCATGGTTATGACGTCATTGTAGGCGGAGAGCTATTTACTGATTACTCCGATCACCCTCGCAAACTTGTCACGCTAAACCCAAAACTCAAATCAACAGGCGCCGGACGCTACCAGCTTCTTTCCCGTTGGTGGGATGCCTACCGCAAGCAGCTTGGCCTGAAAGACTTCTCTCCGAAAAGTCAGGACGCTGTGGCATTGCAGCAGATTAAGGAGCGTGGCGCTTTACCTATGATTGATCGTGGTGATATCCGTCAGGCAATCGACCGTTGCAGCAATATCTGGGCTTCACTGCCGGGCGCTGGTTATGGTCAGTTCGAGCATAAGGCTGACAGCCTGATTGCAAAATTCAAAGAAGCGGGCGGAACGGTCAGAGAGATTGATGTATGAGCAGAGTCACCGCGATTATCTCCGCTCTGGTTATCTGCATCATCGTCTGCCTGTCATGGGCTGTTAATCATTACCGTGATAACGCCATTACCTACAAAGCCCAGCGCGACAAAAATGCCAGAGAACTGAAGCTGGCGAACGCGGCAATTACTGACATGCAGATGCGTCAGCGTGATGTTGCTGCGCTCGATGCAAAATACACGAAGGAGTTAGCTGATGCTAAAGCTGAAAATGATGCTCTGCGTGATGATGTTGCCGCTGGTCGTCGTCGGTTGCACATCAAAGCAGTCTGTCAGTCAGTGCGTGAAGCCACCACCGCCTCCGGCGTGGATAATGCAGCCTCCCCGACTGGCAGACACCGCTGAACGGGATTATTTCACCCTCAGAGAGAGCTGATCACTATGCAAAAACAACTGGAAGGAACCCAGAAGTATATTAATGAGCAGTGCAGATAGAGTTGCCCATATCGATGGGCAACTCATGCAATATGTGAGCAATACACACGCGCTTCCAGCGGAGTATAAATGCCTAAAGTAATAAAACCGAGCAATCCATTTACGAATGTTTGCTGGGTTTCTGTTTTAACAACATTTTCTGCGCCGCCACAAATTTTGGCTGCATCGACAGTTTTCTTCTGCCCAATTCCAGAAACGAAGAAATGATGGGTGATGGTTTCCTTTGGTGCTACTGCTGCCGGTTTGTTTTGAACAGTAAACGTCTGTTGAGCACATCCTGTAATAAGCAGGGCCAGCGCAGTAGCGAGTAGCATTTTTTCATGGTGTTATTCCCGATGCTTTTGAAGTTCGCAGAATCGTATGTGTAGAAAATTAAACAAACCCTAAACAATGAGTTGAAATTTCATATTGTTAATATTTATTAATGTATGTCAGGTGCGATGAATCGTCATTGTATTCCCGGATTAACTATGTCCACAGCCCTGACGGGGAACTTCTCTGCAGGAGTGTCCGGGAATAATTAAAACGATGCACACAGGGTTTAGCGCGTACACGTATTGCATTATGCCAACGCCCCGGTGCTGACACGGAAGAAACCGGACGTTATGATTTAGCGTGGAAAGATTTGTGTAGTGTTCTGAATGCTCTCAGTAAATAGTAATGAATTATCAAAGGTATAGTAATATCTTTTATGTTCATGGATATTTGTAACCCATCGGAAAACTCCTGCTTTAGCAAGATTTTCCCTGTATTGCTGAAATGTGATTTCTCTTGATTTCAACCTATCATAGGACGTTTCTATAAGATGCGTGTTTCTTGAGAATTTAACATTTACAACCTTTTTAAGTCCTTTTATTAACACGGTGTTATCGTTTTCTAACACGATGTGAATATTATCTGTGGCTAGATAGTAAATATAATGTGAGACGTTGTGACGTTTTAGTTCAGAATAAAACAATTCACAGTCTAAATCTTTTCGCACTTGATCGAATATTTCTTTAAAAATGGCAACCTGAGCCATTGGTAAAACCTTCCATGTGATACGAGGGCGCGTAGTTTGCATTATCGTTTTTATCGTTTCAATCTGGTCTGACCTCCTTGTGTTTTGTTGATGATTTATGTCAAATATTAGGAATGTTTTCACTTAATAGTATTGGTTGCGTAACAAAGTGCGGTCCTGCTGGCATTCTGGAGGAAATACAACCGACAGATGTATGTAAGGCCAACGTGCTCAAATCTTCATACAGAAAGATTTGAAGTAATATTTTAACCGCTAGATGAAGAGCAAGCGCATGGAGCGACAAAATGAATAAAGAACAATCTGCTGATGATCCCTCCGTGGATCTGATTCGTGTAAAAATATGCTTAATAGCACCATTTCTATGAGTTACCCTGATGTTGTAATTGCATGTATAGAACATAAGGTGTCTCTGGAAGCATTCAGAGCAATTGAGGCAGCGTTGGTGAAGCACGATAATAATATGAAGGATTATTCCCTGGTGGTTGACTGATCACCATAACTGCTAATCATTCAAACTATTTAGTCTGTGACAGAGCCAACACGCAGTCTGTCACTGTCAGGAAAGTGGTAAAACTGCAACTCAATTACTGCAATGCCCTCGTAATTAAGTGAATTTACAATATCGTCCTGTTCGGAGGGAAGAACGCGGGATGTTCATTCTTCATCACTTTTAATTGATGTATATGCTCTCTTTTCTGACGTTAGTCTCCGACGGCAGGCTTCAATGACCCAGGCTGAAATTCCCGGACCCTTTTTGCTCAAGAGCGATGTTAATTTGTTCAATCATTTGGTTAGGAAAGCGGATGTTGCGGGTTGTTGTTCTGCGGGTTCTGTTCTTCGTTGACATGAGGTTGCCCCGTATTCAGTGTCGCTGATTTGTATTGTCTGAAGTTGTTTTTACGTTAAGTTGATGCAGATCAATTAATACGATACCTGCGTCATAATTGATTATTTGACGTGGTTTGATGGCCTCCACGCACGTTGTGATATGTAGATGATAATCATTATCACTTTACGGGTCCTTTCCGGTGAAAAAAAGGTACCAAAAAACATCGTCGTGAGTAGTGAACCGTAAGCAGCAATACGTAACTGAACGAAG
